# Burden of Central Nervous System Cancer in the United States, 1990-2021

**DOI:** 10.1001/jamaneurol.2025.4286

**Published:** 2025-11-03

**Authors:** GBD 2021 US CNS Cancer Collaborators, Hyun Jin Han, Yun Seo Kim, Seoyeon Park, Jae Il Shin, Min Seo Kim, Ju Hyung Moon, Yong Bae Kim, Hazim S. Ababneh, Ahmed Abu-Zaid, Demelash Areda, Santhosh Arul, Ahmed Y. Azzam, Mainak Bardhan, Mohammad Amin Bayat Tork, Babak Behnam, Gokce Belge Bilgin, Prarthna V. Bhardwaj, Soumitra S. Bhuyan, Nima Broomand Lomer, Meng Xuan Chen, Suma Sri Chennapragada, Xiaochen Dai, Frances E. Dean, Sindhura Deekonda, Xueting Ding, Ojas Prakashbhai Doshi, Abdel Rahman E’mar, Muhammed Elhadi, Jawad Fares, Patrick Fazeli, James L Fisher, Maryam Fotouhi, Ali Gholamrezanezhad, Fidelia Ida, Chidozie Declan Iwu, Mohamed Jalloh, Chinmay T. Jani, Rizwan Kalani, Samuel Berchi Kankam, Foad Kazemi, Ariz Keshwani, Atulya Aman Khosla, Stephen S. Lim, Riffat Mehboob, Tomislav Mestrovic, Ali H. Mokdad, Christopher J. L. Murray, Gurudatta Naik, Zuhair S. Natto, Dang Nguyen, Fred Nugen, Atakan Orscelik, Romil R. Parikh, Louise Penberthy, Richard G. Pestell, Disha Prabhu, Jagadeesh Puvvula, Shakthi Kumaran Ramasamy, Cameron John Sabet, Austin E. Schumacher, Yigit Can Senol, Sunder Sham, Samendra P. Sherchan, Gizeaddis Lamesgin Simegn, Jasvinder A. Singh, Ranjan Solanki, Bahadar S. Srichawla, Jabeen Taiba, Manoj Tanwar, Mike Tuffour Amirikah, Anjul Verma, Ismaeel Yunusa, David X. Zheng, Dong Keon Yon, Keun Young Park

**Affiliations:** 1 Department of Neurosurgery, Yonsei University, Seoul, South Korea; 2 Department of Medicine, Yonsei University, Seoul, South Korea; 3 Department of Biomedical Data Science, Stanford University, Stanford, California; 4 Department of Pediatrics, Yonsei University College of Medicine, Seoul, South Korea; 5 Cardiovascular Disease Initiative, Broad Institute of MIT and Harvard, Cambridge, Massachusetts; 6 Massachusetts General Hospital, Boston; 7 Severance Hospital, Yonsei University, Seoul, South Korea; 8 Department of Radiation Oncology, Massachusetts General Hospital, Boston; 9 Department of Biochemistry and Molecular Medicine, Alfaisal University, Riyadh, Saudi Arabia; 10 College of Graduate Health Sciences, University of Tennessee, Memphis; 11 College of Art and Science, Ottawa University, Surprise, Arizona; 12 School of Life Sciences, Arizona State University, Tempe; 13 Department of Neurological Surgery, University of California, San Francisco; 14 ASIDE Healthcare, Lewes, Delaware; 15 Faculty of Medicine, October 6 University, 6th of October City, Egypt; 16 Miller School of Medicine, University of Miami, Miami, Florida; 17 Biological Science Division, University of Chicago, Chicago, Illinois; 18 Avicenna Biotech Research, Germantown, Maryland; 19 Department of Regulatory Affairs, Amarex Clinical Research, Germantown, Maryland; 20 Department of Radiology, Mayo Clinic, Rochester, Minnesota; 21 Division of Hematology Oncology, University of Massachusetts Medical School, Springfield; 22 Department of Health Administration, Rutgers University, New Brunswick, New Jersey; 23 Department of Radiology, University of Pennsylvania, Philadelphia; 24 School of Dentistry, University of Michigan, Ann Arbor; 25 Department of Hematology-Oncology, LSU Health Shreveport, Shreveport, Louisiana; 26 Institute for Health Metrics and Evaluation, University of Washington, Seattle; 27 Department of Health Metrics Sciences, School of Medicine, University of Washington, Seattle; 28 Department of Mathematics, University of California, Berkeley; 29 Department of Pediatrics, Brookdale University Hospital Medical Center, Brooklyn, New York; 30 Joe C. Wen School of Population & Public Health, University of California, Irvine; 31 Independent Consultant, South Plainfield, New Jersey; 32 Department of Pediatrics, Cleveland Clinic, Cleveland, Ohio; 33 Faculty of Medicine, University of Tripoli, Tripoli, Libya; 34 Houston Methodist Hospital, Houston, Texas; 35 Department of Neurological Surgery, Northwestern University, Chicago, Illinois; 36 Department of Biology and Medicine, Brown University, Providence, Rhode Island; 37 James Cancer Hospital at The Ohio State University, Ohio State University, Columbus; 38 Department of Radiology, University of Southern California, Los Angeles; 39 Department of Medicine, Iran University of Medical Sciences, Tehran, Iran; 40 Pharmacoepidemiology Department, Sanofi, Cambridge, Massachusetts; 41 School of Health Systems and Public Health, University of Washington, Seattle; 42 Department of Neurosurgery, Medical College of Wisconsin, Milwaukee; 43 Sina Trauma and Surgery Research Center, Tehran University of Medical Sciences, Tehran, Iran; 44 Department of Internal Medicine, Harvard University, Cambridge, Massachusetts; 45 Department of Neurology, University of Washington, Seattle; 46 Harvard T.H. Chan School of Public Health, Harvard University, Boston, Massachusetts; 47 Department of Neurosurgery, Johns Hopkins University, Baltimore, Maryland; 48 Feinberg School of Medicine, Northwestern University, Chicago, Illinois; 49 Department of Internal Medicine, Corewell Health East William Beaumont University Hospital, Royal Oak, Michigan; 50 Department of Medical Oncology, Miami Cancer Institute, Miami, Florida; 51 National Heart, Lung, and Blood Institute, Bethesda, Maryland; 52 Research and Development Department, Lahore Medical Research Center, Lahore, Pakistan; 53 University Centre Varazdin, University North, Varazdin, Croatia; 54 Department of Health Services Research, University of Alabama at Birmingham; 55 Department of Dental Public Health, King Abdulaziz University, Jeddah, Saudi Arabia; 56 Department of Health Policy and Oral Epidemiology, Harvard University, Boston, Massachusetts; 57 Department of Medical Engineering, University of South Florida, Tampa; 58 School of Information, University of California, Berkeley; 59 Department of Neurosurgery, University of California, San Francisco; 60 Division of Health Policy and Management, University of Minnesota, Minneapolis; 61 Pennsylvania Cancer and Regenerative Medicine Center, Baruch S. Blumberg Institute, Doylestown; 62 Department of Medicine, Xavier University School of Medicine, Woodbury, New York; 63 Independent Consultant, San Diego, California; 64 Department of Biostatistics, Epidemiology, and Informatics, University of Pennsylvania, Philadelphia; 65 Department of Radiology, Stanford University, Stanford, California; 66 Department of Medicine, Georgetown University, Washington, DC; 67 Department of Pathology and Laboratory Medicine, Northwell Health, New York, New York; 68 Department of Biology, Morgan State University, Baltimore, Maryland; 69 Department of Environmental Health Sciences, Tulane University, New Orleans, Louisiana; 70 Radiology and Radiological Science, Johns Hopkins University, Baltimore, Maryland; 71 School of Medicine, Baylor College of Medicine, Houston, Texas; 72 Department of Medicine Service, US Department of Veterans Affairs, Houston, Texas; 73 Department of Systemic Pathology, Touro College of Osteopathic Medicine, Middletown, New York; 74 Department of Pathology, American University of the Caribbean School of Medicine, Cupecoy, Saint Martin; 75 Department of Neurology, University of Massachusetts Medical School, Worcester; 76 Department of Environmental, Agricultural and Occupational Health, University of Nebraska Medical Center, Omaha; 77 Sri Ramachandra Medical College and Research Institute, Chennai, India; 78 Department of Radiology, University of Alabama at Birmingham; 79 Department of Internal Medicine, Texas Tech University, Odessa; 80 Department of Clinical Pharmacy and Outcomes Sciences, University of South Carolina, Columbia; 81 Department of Medicine, Massachusetts General Hospital, Boston; 82 Department of Pediatrics, Kyung Hee University, Seoul, South Korea

## Abstract

**Importance:**

Primary brain and central nervous system cancer (collectively referred to as CNS cancer) comprises 2% of all human cancers and poses significant health and economic challenges in the United States.

**Objective:**

To analyze CNS cancer burden in the US, stratified by time, location (state and division), sex, age group, and Sociodemographic Index (SDI).

**Design, Setting, and Participants:**

This cross-sectional study involved a repeated analysis of Global Burden of Disease Study (GBD) 2021 data in 2024. Using data from 183 sources, CNS cancer metrics in the US were estimated across states and years. US CNS cancer metrics across all sexes and age groups were included in the GBD.

**Exposure:**

CNS cancer diagnosis.

**Main Outcomes and Measures:**

Overall and age-standardized estimates of the incidence, prevalence, mortality, disability-adjusted life-years (DALYs), years of life lost, and years lived with disability per 100 000 population, including 95% uncertainty intervals (UIs), and time trends.

**Results:**

In 2021, for all age groups and sexes across the US, there were 31 780 incident cases (95% UI, 29971.1 to 32843.9). Age-standardized incidence, DALYs, and mortality rates per 100 000 population were 6.91 (95% UI, 6.58 to 7.12), 134.38 (95% UI, 129.83 to 137.95), and 4.1 (95% UI, 3.87 to 4.22), respectively. Despite no significant change observed in the overall incidence between 1990 and 2021, DALY and mortality rates decreased by 15.77% (95% UI, −17.75% to −13.68%) and 8.41% (95% UI, −11.09% to −6.22%), respectively. Substantial geographic variability was noted. Mississippi, Alabama, Kentucky, and Kansas (West North Central and East South Central divisions) and West Virginia faced persistently high burdens over the past 30 years. Sex differences were evident; disease burden was consistently higher in males compared with females. Age-specific estimates showed a bimodal distribution: the youngest group (<5 years) showed a significant decrease in incidence rate (−34.42% to −11.56%), whereas older age groups (>70 years) experienced increasing trends. DALYs and mortality rates were negatively correlated with SDI (ρ = −0.6860 and ρ = −0.6391; *P* < .001).

**Conclusions and Relevance:**

These findings provide valuable insights into the CNS cancer burden across the US by age, sex, location, and SDI, enabling better public health status assessments, health care policy restructuring, and resource redistribution for improved care.

## Key Points

**Question:**

What were the trends of central nervous system (CNS) cancer burden in the US from 1990 to 2021?

**Findings:**

In this cross-sectional study, analysis of the Global Burden of Disease Study 2021 data on US CNS cancers revealed that although the incidence rate remained relatively stable, both disability-adjusted life-years and mortality rates declined. However, substantial disparities persisted across geographical location, age, sex, and sociodemographic profile.

**Meaning:**

The persistent disparity in CNS cancer burden highlights the urgent need to reevaluate public health policies and redistribute health care resources to better support marginalized and underserved populations.

## Teaser

This cross-sectional study analyzes data from the Global Burden of Disease Study to determine trends in central nervous system cancer over 3 decades in the United States.

## Introduction

Primary brain and central nervous system cancer (collectively referred to as CNS cancer) constitutes 2% of all human cancers and is a heterogeneous disease that consists of solid tumors originating in the brain, spinal cord, cranial nerves, or meninges.^1^ The outcome of CNS cancers is quite variable, depending on tumor location and histology. In addition to high mortality, patients frequently experience neurological sequelae that can impair daily functioning and impose substantial stress on caregivers.^2^ Furthermore, the cost of advanced diagnostic tools and treatment methods (eg, drugs or adjuvant radiotherapy) potentially represents major economic challenges,^3^ especially in the most aggressive tumors and highest-grade malignancies.^4,5^

Previous analyses of the Global Burden of Disease Study (GBD) reported that the global age-standardized incidence rate of CNS cancer, per 100 000 population, increased from 3.75 in 1990 to 4.28 in 2021,^6,7^ with the US among the countries with the highest incidence.^8^ In 2023, a US nationwide analysis estimated 24 810 incident cases and 18 990 deaths,^9^ and total health care expenditures on CNS cancers increased by 2.5-fold (from $2.72 billion to $6.85 billion) over a decade, signifying significant upward trends in per capita expenditures.^10^

The contemporary public health and clinical medicine landscape calls for an up-to-date comprehensive study of the CNS cancer burden. Previous studies have highlighted the high incidence and economic burden of CNS cancer in the US but lacked detailed analyses of temporal trends, regional variations, and disparities across sociodemographic groups. Such disparities, well documented for other cancers in the US, are likely pertinent for CNS cancer.^11^

This study aimed to provide updated epidemiological estimates of CNS cancer in the US from 1990 to 2021, further stratified by geographic location (state and division), age, sex, and sociodemographic profile. Investigating these subgroup-specific burdens is vital for identifying disproportionately affected populations and guiding targeted resource allocation and public health interventions to reduce health inequalities.

## Methods

### Overview of Study and Data

The GBD 2021 adhered to the Guidelines for Accurate and Transparent Health Estimates Reporting (eAppendix 1 in Supplement 1).^12^ Because this study used publicly available, deidentified, and aggregated data from the GBD 2021, informed consent and institutional review board approval were not required. This article was produced as part of the GBD Collaborator Network and adheres to the GBD Protocol.^13^

This study used data from the repeated cross-sectional GBD 2021, coordinated by the Institute for Health Metrics and Evaluation (IHME), which integrates multiple data sources, including vital registration, verbal autopsy, and cancer registry data, to develop the GBD cause of death database, containing cancer mortality data.^14,15^ Epidemiologic estimates, including incidence, prevalence, deaths, years lived with disability (YLDs), years of life lost (YLLs), and disability-adjusted life-years (DALYs), were obtained for CNS cancers in all US states and the District of Columbia.^14,15^ CNS cancer cases were identified using the *International Statistical Classification of Diseases and Related Health Problems, Tenth Revision*, with CNS cancer codes C70-C72.9 and C75.1-C75.3 (eAppendix 2 in Supplement 1).^14^ US CNS cancer metrics were estimated from 183 registries and health records (eAppendix 3 in Supplement 1).

### Statistical Analysis

The GBD estimation framework has been detailed previously.^14,15^ Briefly, mortality was derived using mortality to incidence ratios modeled with spatiotemporal gaussian process regression. Mortality estimates were calculated by multiplying the mortality to incidence ratios with cancer registry incidence data. Cause-specific mortality across states, years, and age groups was modeled using the cause of death ensemble model. YLLs were calculated by applying age-specific GBD life expectancy to the age-specific mortality estimates. YLDs were derived by combining prevalence estimates with the corresponding disability weights for various cancer survival stages. DALYs represents the sum of YLLs and YLDs, stratified by age, sex, location, and year, following methodologies described in the GBD capstone articles.^14,15^ The results were presented as mean values with 95% uncertainty intervals (UIs), which are used in the GBD study to reflect multiple sources of uncertainty (eg, sampling, modeling, and data sources) beyond what is captured by a traditional confidence interval. UIs were calculated from 500 draws from a log-normal distribution that incorporates this combined uncertainty. Significance was inferred if the UI excluded zero.

The Sociodemographic Index (SDI) is a composite metric of lag-distributed income per capita, average years of education for individuals aged 15 years and older (EDU15+), and the total fertility rate of those younger than 25 years (TFU25). The indicators were normalized (0-1), and their geometric mean was used for the final SDI. State-specific data extracted from the GBD 2021 demographics database were matched to each state-year.^16^ The division-level SDI was averaged from state-level values within each of the 9 geographic divisions defined by the US Census Bureau (eg, New England, Pacific), which group states into regions for analysis.^17^ As suitable data for lag-distributed income and EDU15+ were unavailable, the 2021 per capita income and high school graduation (or equivalency) rates for individuals 18 years and older were used as proxies.^18,19^ TFU25 data were sourced from the Centers for Disease Control and Prevention.^20,21^

Spearman rank-order correlation quantified the overall monotonic association between the SDI of US states and the age-standardized incidence, mortality, and DALY rates, while locally weighted scatterplot smoothing was used to visualize potential nonlinear associations between SDI and the age-standardized burden metrics at the division level. Statistical significance was defined as 2-sided *P* < .05. Secondary analyses were performed using Python version 3.10.4 (Python Software Foundation) and R version 4.2.1 (R Foundation).

## Results

### Overall Trends in the US

In 2021, there were estimated 31 780 incident cases (95% UI, 29 971.10 to 32 843.90) of CNS cancer in the US, corresponding to an age-standardized incidence rate of 6.91 (95% UI, 6.58 to 7.12) per 100 000 population (Table). The DALY rate per 100 000 population in 2021 was 134.38 (95% UI, 129.83 to 137.95), primarily from YLLs (131.78; 95% UI, 127.25 to 134.95) rather than YLDs (2.60; 95% UI, 1.92 to 3.40). Between 1990 and 2021, DALYs and mortality significantly decreased (15.77%; 95% UI, −17.75% to −13.68%; and 8.41%; 95% UI, −11.09% to −6.22%, respectively), whereas the incidence rate did not change significantly (−1.45%; 95% UI, −4.41% to 0.91%). The changes in the DALY rate were primarily driven by YLLs (−16.06%; 95% UI, −18.03% to −14.00%; YLDs, 2.03%; 95% UI, −2.10% to 6.25%).

**Table.** State-Level Incidence, DALYs, and Mortality of Central Nervous System Cancer (1990-2021)

Location	Incidence (95% UI)			DALYs (95% UI)			Deaths (95% UI)		
	Absolute No. (2021)	Age-standardized rate per 100 000 people (2021)	Percentage change, % (1990-2021)	Absolute No. (2021)	Age-standardized rate per 100 000 people (2021)	Percentage change, % (1990-2021)	Absolute No. (2021)	Age-standardized rate per 100 000 people (2021)	Percentage change, % (1990-2021)
United States	31 780.00 (29 971.10 to 32 843.90)	6.91 (6.58 to 7.12)	−1.45 (−4.41 to 0.91)	594 996.15 (571 294.69 to 610 278.40)	134.38 (129.83 to 137.95)	−15.77 (−17.75 to −13.68)	21 444.08 (20 045.85 to 22 166.97)	4.10 (3.87 to 4.22)	−8.41 (−11.09 to −6.22)
Alabama	577.20 (494.55 to 664.25)	8.15 (7.05 to 9.33)	8.51 (−7.36 to 25.85)	12 101.20 (10 421.29 to 13 949.93)	180.17 (157.11 to 205.96)	−2.28 (−14.59 to 12.56)	431.10 (369.87 to 497.72)	5.44 (4.69 to 6.26)	5.79 (−8.70 to 22.35)
Alaska	62.55 (54.14 to 73.83)	7.38 (6.29 to 9.24)	18.53 (−0.70 to 47.07)	1236.42 (1060.70 to 1443.72)	146.00 (124.29 to 176.99)	−0.07 (−15.75 to 22.19)	37.31 (32.51 to 42.78)	3.90 (3.38 to 4.51)	−4.80 (−17.40 to 10.28)
Arizona	689.13 (592.22 to 794.93)	6.97 (6.02 to 8.01)	8.88 (−6.21 to 26.03)	12 601.23 (10 792.16 to 14 520.14)	133.18 (115.31 to 152.62)	−8.63 (−20.92 to 4.66)	454.62 (390.36 to 525.31)	3.99 (3.45 to 4.60)	1.42 (−12.51 to 16.81)
Arkansas	329.31 (282.82 to 379.18)	8.02 (6.95 to 9.18)	3.75 (−11.37 to 19.38)	6852.66 (5916.10 to 7889.39)	176.27 (153.36 to 201.80)	−5.44 (−18.70 to 8.89)	239.37 (205.65 to 276.38)	5.17 (4.47 to 5.96)	0.64 (−13.70 to 16.89)
California	3222.04 (2821.36 to 3667.82)	6.15 (5.42 to 7.02)	−6.16 (−18.70 to 7.46)	61 314.24 (53 415.78 to 69 330.06)	119.18 (105.50 to 134.14)	−22.15 (−31.47 to −12.10)	2210.66 (1869.90 to 2503.29)	3.70 (3.17 to 4.19)	−12.56 (−24.18 to −1.20)
Colorado	546.88 (458.09 to 640.35)	7.00 (5.88 to 8.18)	−1.08 (−17.16 to 15.51)	9900.18 (8274.83 to 11 553.66)	128.77 (109.46 to 148.96)	−16.11 (−29.51 to −2.75)	348.79 (287.07 to 408.63)	3.96 (3.29 to 4.62)	−9.33 (−24.20 to 6.00)
Connecticut	441.28 (364.92 to 524.88)	8.30 (6.93 to 9.77)	−3.87 (−19.98 to 14.96)	6305.03 (5250.93 to 7410.65)	124.32 (103.84 to 144.44)	−18.07 (−31.23 to −3.83)	233.62 (195.62 to 275.14)	3.80 (3.17 to 4.46)	−11.33 (−25.37 to 4.96)
Delaware	93.57 (82.96 to 105.27)	6.56 (5.78 to 7.36)	1.63 (−10.61 to 14.82)	1772.88 (1569.10 to 1993.90)	130.07 (116.15 to 144.98)	−12.90 (−23.30 to −2.16)	63.60 (56.43 to 72.18)	3.85 (3.42 to 4.34)	−10.88 (−21.09 to 0.47)
Washington, DC	36.01 (30.73 to 41.41)	4.36 (3.76 to 5.02)	−23.70 (−36.04 to −10.90)	803.70 (688.50 to 927.22)	100.80 (87.13 to 116.03)	−43.24 (−51.77 to −33.88)	26.10 (22.39 to 30.10)	2.94 (2.53 to 3.39)	−34.07 (−44.23 to −22.97)
Florida	2014.68 (1705.77 to 2361.49)	6.25 (5.38 to 7.30)	−1.40 (−16.34 to 15.53)	41 025.10 (35 027.99 to 47 563.22)	134.83 (116.98 to 154.42)	−16.55 (−27.28 to −4.04)	1550.85 (1306.73 to 1804.19)	4.05 (3.45 to 4.68)	−9.00 (−21.41 to 4.78)
Georgia	944.64 (817.93 to 1088.00)	6.63 (5.75 to 7.60)	−0.94 (−13.40 to 15.11)	18 538.30 (16 038.82 to 21 346.26)	134.53 (117.65 to 153.81)	−17.99 (−27.91 to −6.08)	642.28 (552.39 to 742.02)	4.09 (3.54 to 4.70)	−10.12 (−21.77 to 3.63)
Hawaiʻi	98.41 (83.81 to 113.45)	4.70 (4.04 to 5.38)	0.44 (−15.36 to 16.65)	1765.70 (1500.81 to 2043.79)	88.92 (76.15 to 103.05)	−14.52 (−26.75 to −1.07)	64.48 (54.20 to 75.40)	2.65 (2.25 to 3.06)	−7.30 (−21.29 to 8.23)
Idaho	186.15 (155.07 to 215.44)	7.41 (6.22 to 8.51)	2.07 (−14.30 to 17.97)	3432.33 (2882.96 to 4000.04)	141.91 (120.67 to 164.02)	−11.89 (−25.48 to 2.71)	124.88 (104.84 to 145.33)	4.45 (3.73 to 5.18)	−3.44 (−18.46 to 12.87)
Illinois	1153.81 (983.09 to 1328.59)	6.61 (5.67 to 7.63)	−0.73 (−14.83 to 14.85)	21 395.86 (18 231.59 to 24 675.02)	126.94 (110.01 to 145.35)	−18.65 (−29.96 to −6.79)	763.47 (644.06 to 882.76)	3.84 (3.27 to 4.43)	−12.17 (−24.69 to 1.40)
Indiana	683.75 (590.63 to 785.55)	7.40 (6.43 to 8.46)	3.60 (−11.72 to 19.33)	13 277.60 (11 457.27 to 15 223.53)	149.46 (130.71 to 170.79)	−7.32 (−19.90 to 6.49)	473.13 (400.75 to 545.55)	4.53 (3.89 to 5.20)	0.10 (−13.45 to 15.30)
Iowa	369.42 (314.54 to 424.47)	8.10 (6.91 to 9.31)	10.10 (−7.45 to 30.95)	6470.76 (5462.96 to 7484.52)	151.68 (129.75 to 173.70)	−3.00 (−17.89 to 12.42)	238.12 (202.57 to 276.29)	4.60 (3.89 to 5.31)	3.00 (−13.12 to 19.52)
Kansas	325.60 (274.03 to 379.48)	8.21 (6.96 to 9.48)	8.55 (−8.11 to 27.71)	6065.12 (5093.86 to 7057.89)	159.20 (134.37 to 184.40)	−1.65 (−16.67 to 14.31)	217.02 (181.89 to 252.41)	4.85 (4.06 to 5.63)	6.28 (−10.57 to 23.74)
Kentucky	555.83 (476.87 to 643.56)	8.94 (7.69 to 10.30)	4.12 (−10.60 to 22.22)	9934.74 (8481.61 to 11386.97)	165.41 (141.72 to 189.20)	−2.87 (−16.54 to 11.71)	347.09 (296.82 to 398.74)	4.93 (4.22 to 5.64)	1.86 (−12.88 to 17.21)
Louisiana	493.28 (425.83 to 572.70)	8.00 (6.91 to 9.28)	11.74 (−3.52 to 29.91)	9368.70 (8129.14 to 10827.33)	157.90 (137.87 to 180.31)	−2.25 (−15.60 to 12.50)	318.33 (274.31 to 368.21)	4.65 (4.03 to 5.39)	4.59 (−9.95 to 21.43)
Maine	168.80 (143.45 to 196.71)	7.75 (6.62 to 9.06)	7.85 (−8.56 to 26.84)	3074.00 (2633.58 to 3581.83)	149.12 (128.42 to 173.16)	−2.03 (−17.38 to 14.16)	117.46 (100.96 to 136.39)	4.58 (3.95 to 5.32)	3.04 (−12.96 to 19.54)
Maryland	514.77 (437.04 to 601.98)	6.14 (5.25 to 7.18)	−10.53 (−24.45 to 5.36)	9925.91 (8409.08 to 11 597.29)	122.36 (104.67 to 142.28)	−23.64 (−35.50 to −11.19)	345.07 (290.35 to 401.34)	3.62 (3.09 to 4.21)	−20.78 (−32.67 to −7.05)
Massachusetts	759.53 (631.27 to 915.03)	7.16 (6.00 to 8.52)	−5.84 (−20.41 to 12.62)	12 399.13 (10 299.74 to 14 737.10)	121.38 (101.83 to 143.06)	−23.54 (−36.75 to −8.91)	487.27 (407.18 to 578.58)	4.05 (3.39 to 4.79)	−11.83 (−26.33 to 5.12)
Michigan	1087.91 (926.13 to 1269.72)	7.37 (6.33 to 8.53)	−1.13 (−16.19 to 14.79)	19 945.49 (17 186.55 to 23 066.13)	144.51 (125.37 to 164.88)	−14.85 (−26.33 to −2.02)	723.15 (618.15 to 837.42)	4.31 (3.72 to 4.97)	−8.83 (−21.45 to 5.63)
Minnesota	615.17 (512.29 to 719.75)	7.84 (6.63 to 9.13)	−1.20 (−18.05 to 16.59)	10 384.67 (8675.63 to 12 151.06)	135.87 (115.22 to 157.35)	−14.84 (−28.20 to −1.15)	376.87 (312.99 to 442.82)	4.14 (3.47 to 4.85)	−10.20 (−24.67 to 5.50)
Mississippi	329.45 (286.04 to 379.53)	8.27 (7.22 to 9.53)	15.31 (−0.51 to 34.73)	7219.27 (6234.69 to 8334.50)	190.99 (166.57 to 218.22)	4.85 (−8.82 to 20.26)	247.62 (211.82 to 286.37)	5.59 (4.83 to 6.43)	12.39 (−2.59 to 30.38)
Missouri	651.66 (557.40 to 752.73)	7.57 (6.52 to 8.74)	6.22 (−10.29 to 24.03)	12 559.74 (10 689.92 to 14 584.60)	152.51 (130.91 to 175.56)	−6.28 (−20.62 to 8.50)	449.74 (384.83 to 521.45)	4.57 (3.92 to 5.29)	0.46 (−14.45 to 16.53)
Montana	117.41 (101.33 to 135.59)	7.47 (6.47 to 8.59)	10.43 (−5.27 to 28.74)	2221.65 (1921.92 to 2575.68)	149.41 (131.00 to 172.50)	−1.20 (−14.66 to 14.87)	80.73 (68.94 to 93.86)	4.37 (3.77 to 5.05)	2.83 (−11.83 to 18.94)
Nebraska	225.00 (192.92 to 259.31)	8.48 (7.33 to 9.79)	6.40 (−9.96 to 23.21)	4010.40 (3422.24 to 4614.39)	157.97 (136.30 to 181.23)	−6.97 (−20.95 to 8.06)	145.54 (124.62 to 167.70)	4.83 (4.14 to 5.55)	0.43 (−14.31 to 16.03)
Nevada	258.69 (221.47 to 298.64)	6.14 (5.25 to 7.01)	−2.03 (−16.05 to 13.61)	5017.97 (4270.03 to 5791.08)	122.27 (105.48 to 140.03)	−17.23 (−29.16 to −4.55)	177.35 (150.53 to 205.76)	3.78 (3.21 to 4.36)	−9.48 (−22.91 to 4.97)
New Hampshire	155.97 (131.01 to 181.13)	7.32 (6.25 to 8.50)	4.67 (−11.56 to 22.24)	2721.21 (2316.43 to 3187.75)	131.15 (112.06 to 152.69)	−11.21 (−24.68 to 4.28)	102.97 (87.20 to 119.74)	4.13 (3.50 to 4.80)	−5.77 (−20.29 to 10.74)
New Jersey	1041.90 (886.76 to 1234.29)	7.86 (6.75 to 9.17)	−4.59 (−20.32 to 12.04)	15 117.62 (12 809.10 to 17 713.22)	115.83 (99.03 to 134.88)	−22.84 (−34.72 to −10.03)	553.34 (464.85 to 648.87)	3.63 (3.07 to 4.25)	−12.25 (−25.75 to 2.38)
New Mexico	187.84 (159.85 to 220.23)	6.49 (5.54 to 7.55)	20.20 (0.31 to 41.59)	3851.21 (3292.56 to 4506.07)	140.18 (120.51 to 162.98)	6.32 (−9.70 to 24.49)	131.70 (111.89 to 154.40)	3.99 (3.40 to 4.68)	12.96 (−4.28 to 32.98)
New York	1634.52 (1363.18 to 1896.40)	5.77 (4.90 to 6.61)	−3.95 (−17.61 to 11.62)	29 165.78 (24 989.81 to 33 942.49)	107.08 (92.70 to 123.05)	−25.71 (−35.88 to −14.22)	1064.05 (889.34 to 1243.64)	3.28 (2.78 to 3.81)	−16.71 (−28.68 to −2.53)
North Carolina	986.00 (841.10 to 1133.65)	6.75 (5.83 to 7.73)	−2.29 (−16.41 to 13.32)	19 069.23 (16 258.53 to 22 105.39)	136.16 (117.31 to 156.27)	−17.40 (−29.19 to −4.57)	686.44 (583.68 to 797.34)	4.14 (3.53 to 4.78)	−11.71 (−24.77 to 2.24)
North Dakota	77.91 (66.43 to 89.53)	7.63 (6.62 to 8.72)	0.22 (−14.58 to 17.22)	1448.59 (1250.01 to 1651.14)	149.37 (129.48 to 169.48)	−8.39 (−20.47 to 5.77)	49.93 (42.66 to 57.53)	4.24 (3.62 to 4.86)	−9.76 (−22.63 to 4.27)
Ohio	1233.82 (1057.31 to 1415.40)	7.28 (6.26 to 8.36)	4.62 (−10.38 to 20.75)	23 938.82 (20 681.47 to 27 426.39)	148.18 (129.22 to 168.38)	−6.58 (−18.03 to 6.62)	875.23 (752.41 to 1002.29)	4.53 (3.91 to 5.17)	1.92 (−11.44 to 16.96)
Oklahoma	384.58 (332.27 to 439.83)	7.23 (6.25 to 8.22)	1.98 (−12.44 to 17.19)	7986.77 (6975.08 to 9163.00)	157.67 (138.54 to 178.51)	−2.24 (−14.60 to 11.19)	277.78 (241.11 to 322.28)	4.70 (4.10 to 5.41)	2.38 (−11.06 to 17.15)
Oregon	477.20 (403.52 to 570.51)	7.63 (6.48 to 8.96)	−6.62 (−20.80 to 10.65)	8370.64 (7216.96 to 9925.13)	139.39 (120.78 to 163.47)	−19.94 (−31.20 to −5.79)	311.07 (263.81 to 368.72)	4.28 (3.67 to 5.05)	−14.28 (−26.75 to 1.14)
Pennsylvania	1332.71 (1132.28 to 1555.43)	6.99 (6.00 to 8.03)	−2.95 (−16.64 to 11.87)	24 365.97 (20 850.12 to 28 080.39)	134.86 (116.92 to 153.91)	−17.31 (−28.41 to −5.28)	910.51 (773.63 to 1056.32)	4.10 (3.52 to 4.73)	−11.06 (−23.71 to 2.53)
Rhode Island	111.80 (93.65 to 130.90)	6.83 (5.75 to 7.93)	−9.49 (−24.61 to 7.29)	1942.70 (1642.19 to 2285.10)	124.20 (104.76 to 145.85)	−24.70 (−37.61 to −10.80)	74.87 (63.27 to 88.05)	3.96 (3.37 to 4.65)	−18.17 (−31.43 to −3.07)
South Carolina	550.04 (466.96 to 624.86)	7.51 (6.42 to 8.55)	5.83 (−8.85 to 22.18)	11 109.62 (9461.01 to 12 826.51)	159.68 (137.93 to 182.62)	−11.16 (−22.98 to 2.92)	396.86 (336.80 to 455.84)	4.77 (4.08 to 5.46)	−5.08 (−19.19 to 9.53)
South Dakota	98.02 (85.47 to 111.68)	8.20 (7.20 to 9.28)	5.83 (−9.62 to 21.34)	1882.96 (1652.59 to 2151.85)	166.73 (147.66 to 188.62)	−4.01 (−16.42 to 9.54)	66.38 (57.98 to 76.28)	4.82 (4.24 to 5.51)	0.49 (−13.29 to 15.36)
Tennessee	749.94 (644.39 to 868.91)	7.82 (6.78 to 8.99)	4.05 (−10.38 to 19.87)	15 222.68 (13 134.04 to 17 647.11)	165.44 (144.36 to 190.32)	−8.01 (−20.46 to 5.89)	532.38 (457.98 to 619.03)	4.92 (4.25 to 5.68)	−3.73 (−17.06 to 11.01)
Texas	2391.76 (2093.89 to 2712.52)	6.46 (5.67 to 7.32)	−9.25 (−20.86 to 4.57)	45 797.20 (40 265.43 to 52 077.42)	125.75 (111.40 to 141.99)	−23.70 (−32.75 to −13.49)	1604.18 (1403.74 to 1822.03)	3.97 (3.49 to 4.50)	−15.48 (−25.61 to −3.85)
Utah	255.49 (219.38 to 295.14)	6.79 (5.85 to 7.84)	2.95 (−12.60 to 20.36)	4742.85 (4125.22 to 5436.38)	127.97 (111.60 to 145.88)	−13.54 (−24.83 to −0.90)	160.82 (138.23 to 186.08)	4.06 (3.50 to 4.69)	−2.74 (−16.17 to 11.84)
Vermont	70.10 (60.97 to 79.92)	7.21 (6.31 to 8.18)	7.34 (−6.71 to 22.79)	1248.30 (1102.58 to 1425.51)	134.51 (120.29 to 152.18)	−10.34 (−20.66 to 1.62)	46.74 (40.96 to 53.18)	4.04 (3.55 to 4.59)	−8.87 (−19.78 to 3.77)
Virginia	795.77 (675.35 to 932.51)	6.76 (5.79 to 7.90)	1.22 (−14.50 to 19.09)	14 572.70 (12 356.32 to 17 082.83)	127.22 (108.47 to 147.46)	−15.64 (−27.74 to −2.13)	520.14 (439.82 to 607.38)	3.89 (3.33 to 4.52)	−10.21 (−23.22 to 4.85)
Washington	783.06 (661.33 to 912.26)	7.15 (6.10 to 8.24)	−6.85 (−21.17 to 8.27)	14 588.27 (12 284.27 to 16 899.23)	139.19 (117.82 to 159.54)	−19.71 (−32.53 to −7.22)	528.58 (446.54 to 611.98)	4.30 (3.63 to 4.96)	−14.16 (−27.50 to 0.20)
West Virginia	208.19 (179.15 to 241.45)	7.81 (6.80 to 9.03)	24.47 (6.71 to 44.56)	4285.35 (3696.15 to 4948.00)	171.50 (148.81 to 197.70)	16.12 (0.32 to 34.27)	153.77 (132.29 to 177.74)	5.01 (4.34 to 5.79)	19.21 (2.23 to 38.80)
Wisconsin	644.43 (539.76 to 757.27)	7.73 (6.49 to 8.98)	−0.93 (−17.26 to 16.45)	11 553.83 (9837.41 to 13 515.33)	143.82 (123.71 to 166.72)	−11.52 (−24.62 to 3.03)	421.84 (357.27 to 493.42)	4.36 (3.71 to 5.11)	−5.96 (−19.87 to 11.32)
Wyoming	57.03 (49.90 to 64.12)	7.14 (6.25 to 8.03)	4.60 (−9.11 to 19.72)	1093.84 (973.53 to 1231.17)	142.15 (127.18 to 159.27)	−7.68 (−17.94 to 4.49)	38.84 (33.99 to 43.70)	4.30 (3.80 to 4.82)	−0.60 (−12.20 to 12.84)

Abbreviations: DALYs, disability-adjusted life-years; UI, uncertainty interval.

### Geographic Variation in CNS Cancer Burden

**Figure 1.**
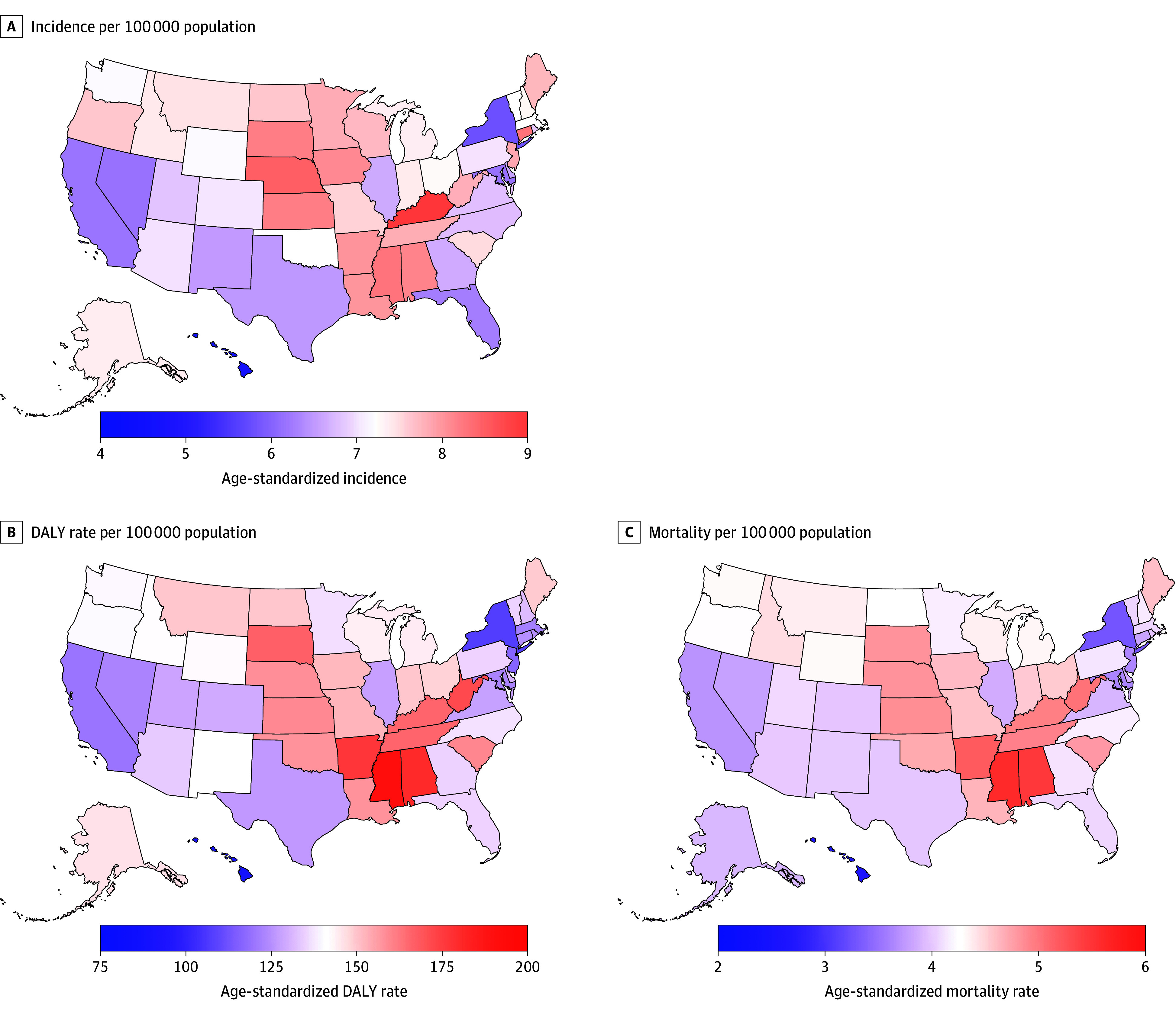
Central Nervous System Cancer Burden Across US States, 2021 Age-standardized rates for incidence (A), disability-adjusted life-years (DALYs) (B), and mortality (C) per 100 000 population of central nervous system cancer in the US, 2021.

Age-standardized incidence, DALYs, and mortality rates showed notable geographic variations. In 2021, age-standardized incidence rates per 100 000 population were generally higher in the West North Central (Nebraska, Kansas) and East South Central divisions (Kentucky, Mississippi) (Figure 1A), with the highest in Kentucky (8.94; 95% UI, 7.69 to 10.30). In contrast, coastal states generally had lower rates, with the lowest in Washington, DC (4.36 per 100 000 population; 95% UI, 3.76 to 5.02), followed by Hawaiʻi, New York, and Maryland. From 1990 to 2021, the change in incidence rates ranged from 24.47% (95% UI, 6.71% to 44.56%) in West Virginia to −23.70% (95% UI, −36.04% to −10.90%) in Washington, DC. However, no significant changes were observed in 48 of 50 states and Washington, DC.

In 2021, age-standardized DALYs resembled the incidence pattern, with higher rates in the East South Central division (Mississippi, Alabama) and lower rates in the coastal states (Figure 1B). Mississippi had the highest rate, at 190.99 per 100 000 population (95% UI, 166.57 to 218.22). Hawaiʻi recorded the lowest rate at 88.92 per 100 000 population (95% UI, 76.15 to 103.05), followed by New York, New Jersey, and California. Between 1990 and 2021, DALY rates significantly decreased in 24 states, and West Virginia was the only state that exhibited a significant increase of 16.12% (95% UI, 0.32% to 34.27%).

The age-standardized mortality rates per 100 000 population in 2021 mirrored the geographic trends in incidence and DALYs (Figure 1C) and ranged from 5.59 (Mississippi: 95% UI, 4.83 to 6.43) to 2.65 (Hawaiʻi: 95% UI, 2.25 to 3.06). Although most states showed a decreasing trend in deaths from 1990 to 2021, only 5 states/district (California; Washington, DC; Maryland; New York; Rhode Island) showed statistically significant decreases. West Virginia was the only state with a significant increase in the age-standardized mortality rate (19.21%; 95% UI, 2.23% to 38.80%).

### Sex-Specific CNS Cancer Burden

**Figure 2.**
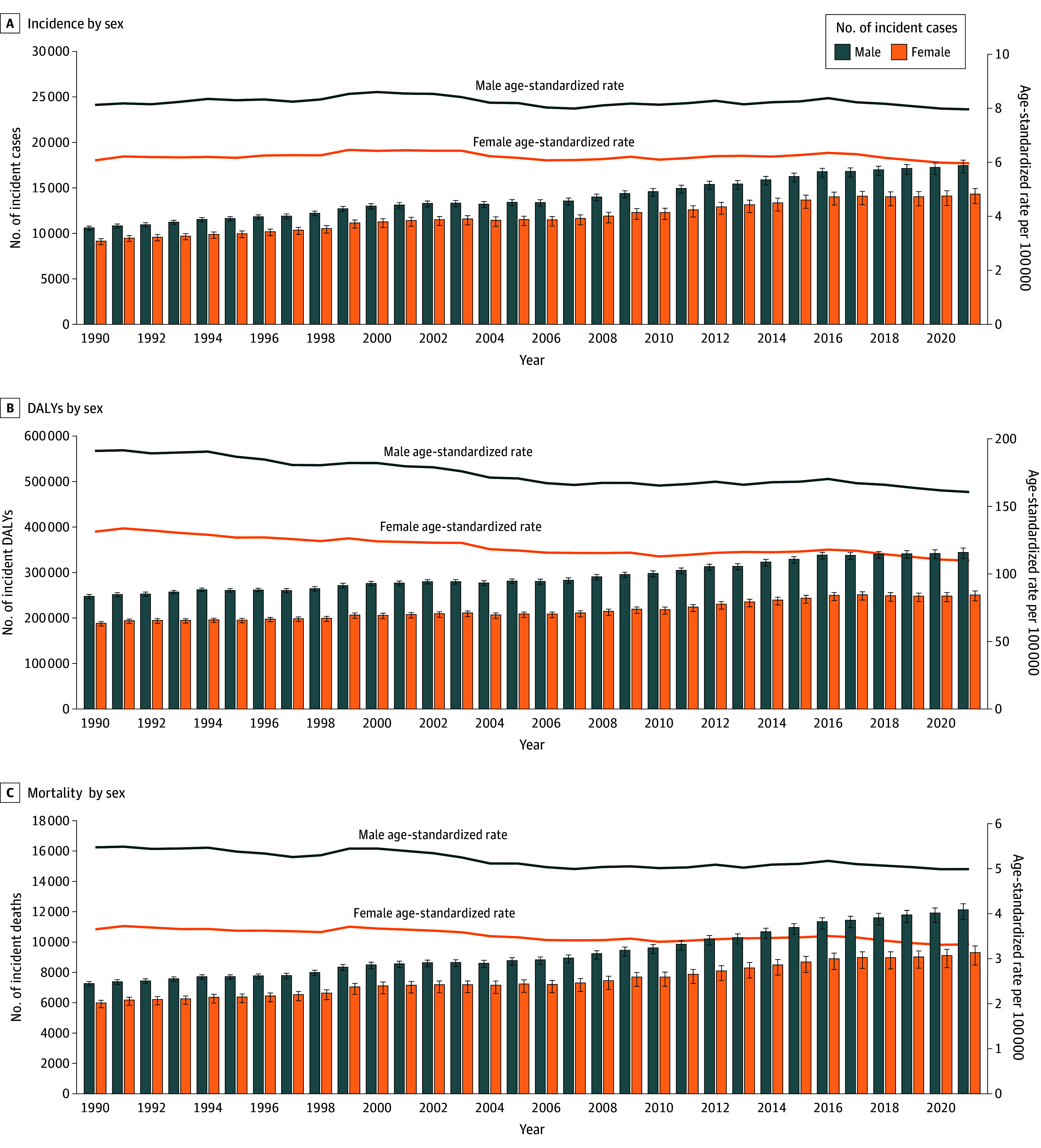
Sex-Stratified Trends of Central Nervous System Cancer Burden in the US Incidence (A), disability-adjusted life-years (DALYs) (B), death count (C), and age-standardized rates per 100 000 population by sex, 1990-2021. Error bars indicate 95% uncertainty intervals.

In 2021, males had higher CNS cancer incidence rates compared with females. Males accounted for 17 459.65 cases (95% UI, 16 653.75-18 044.82), with an age-standardized incidence rate of 7.96 per 100 000 population (95% UI, 7.64-8.22), whereas females had 14 320.35 cases (95% UI, 13 269.49-14 929.73), with a rate of 5.97 per 100 000 population (95% UI, 5.66-6.21) (Figure 2). Between 1990 and 2021, the incidence count increased significantly for both sexes, albeit without significant changes in the age-standardized incidence rates.

The DALYs and mortality count and rate were significantly higher in males compared with females in 2021 (Figure 2). For both sexes, the counts increased, but the age-standardized rates decreased between 1990 and 2021. The percentage changes in these measures were comparable between males and females.

### Age- and Sex-Group–Specific CNS Cancer Burden

**Figure 3.**
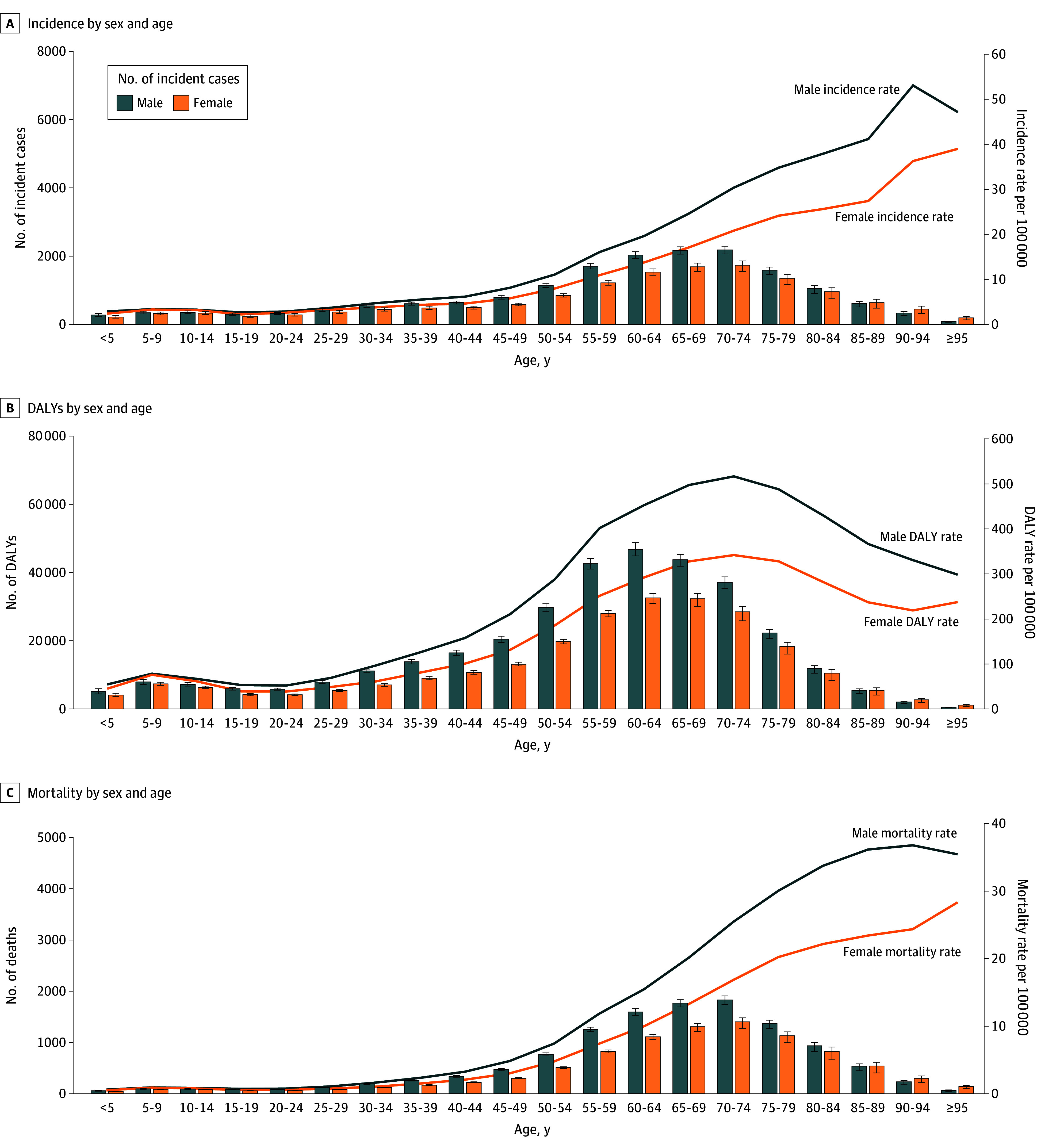
Age- and Sex-Stratified Trends of Central Nervous System Cancer Burden in the US Incidence (A),disability-adjusted life-years (DALYs) (B), death count (C), and rates per 100 000 population by sex and age, 2021. Error bars indicate 95% uncertainty intervals.

The incidence rate for CNS cancer generally increased with age, with a minor peak in the 5- to 9-year and 10- to 14-year age groups (Figure 3). No sex-based differences were noted in childhood; however, the incidence rates began to diverge after age 30 years, with males having significantly higher rates than females in all age groups from 30 to 89 years. The rates converged after age 90 years, with no significant differences observed in the age group 95 years and older.

A bimodal peak pattern was observed in age-specific DALY rates, with peaks in the 5- to 9-year and 70- to 74-year age group (Figure 3). The difference between male and female rates was insignificant up to the 10- to 14-year age group, after which sex differences widened with age. The highest DALY rate per 100 000 population was observed in males aged 70 to 74 years (516.96; 95% UI, 491.24-539.07), which significantly exceeded the corresponding rate in females (342.10; 95% UI, 310.44-361.34). Following this secondary peak, the rates for both sexes and the difference between them decreased with age.

The age-specific mortality trend mirrored that of incidence, with rates increasing with age. A slight increase was noted in the 5- to 9-year and 10- to 14-year age groups. Male mortality rates were significantly higher than female mortality rates from age 30 years to 95 years and older, after which the difference narrowed. (Figure 3).

The distribution of measures across the age groups changed over time. From 1990 to 2021, DALY and mortality rates decreased significantly across all age groups under 70 to 74 years. In older age groups, the rates were initially lower in 1990 and 1995 but increased over time, reflecting an evolving demographic impact on the disease burden.

### CNS Cancer Patterns With SDI

**Figure 4.**
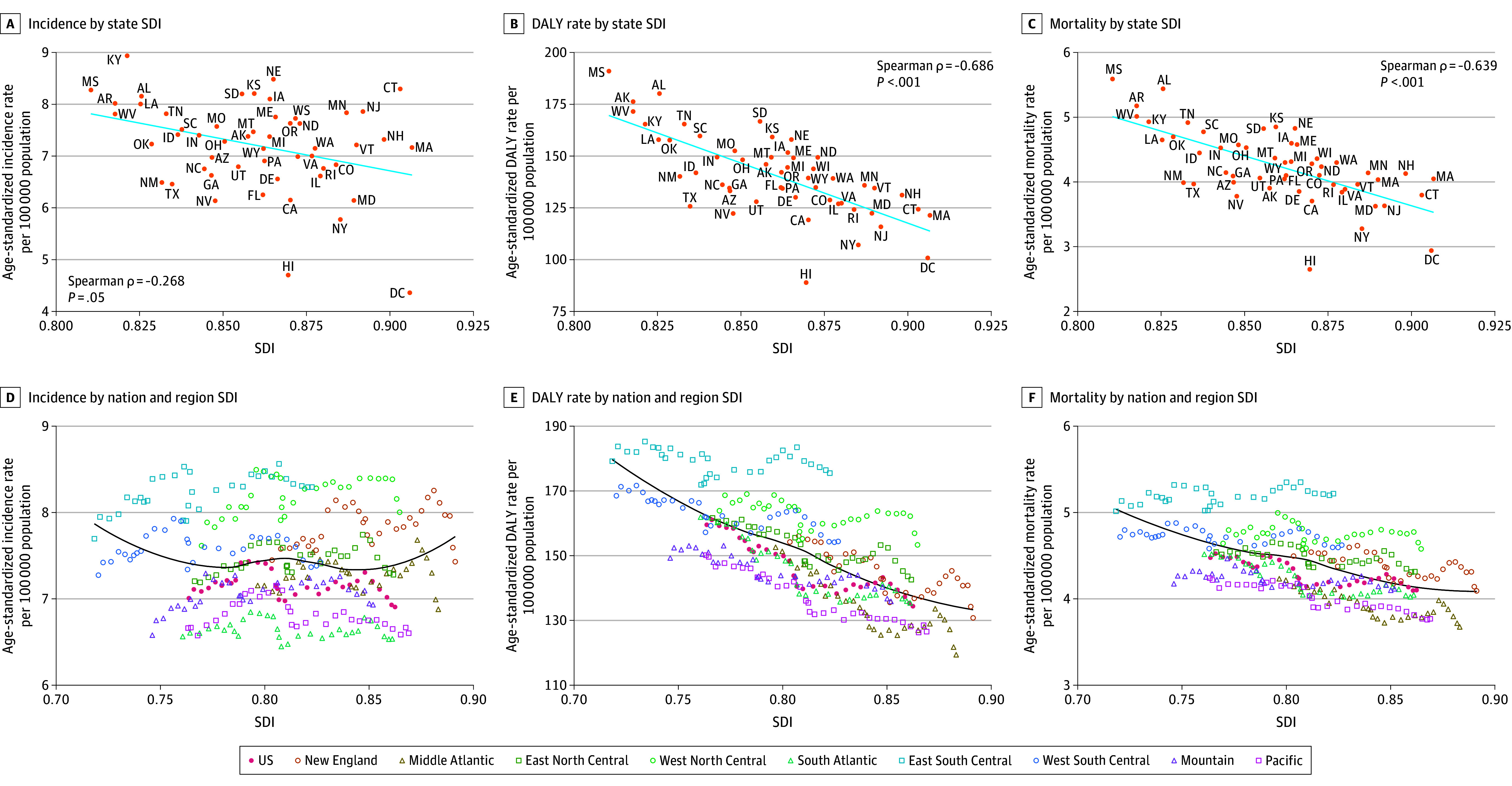
Correlation of Sociodemographic Index (SDI) With Age-Standardized Rates of Incidence, Disability-Adjusted Life-Years (DALYs), and Mortality per 100 000 Population of Central Nervous System Cancer in the US Incidence (A), DALYs (B), and death (C) by state SDI, 2021. Incidence (D), DALYs (E), and death (F) by nation and region SDI, 1990–2021. The yearly average SDI and DALY rates for each division from 1990 to 2021 were calculated. The year-wise notation of each datapoint has not been marked. Trendlines for panels A-C and D-F indicate linear and locally weighted scatterplot smoothing regression trendlines, respectively.

Epidemiological measures for 2021 were plotted against the SDI of the US and individual states to analyze the correlation. The incidence rates (ρ = −0.2683) approached significance (*P* = .05) (Figure 4A). Both DALY (ρ = −0.6860; *P* < .001) and mortality (ρ = −0.6391; *P* < .001) rates showed a significant negative correlation with SDI. The pattern for DALYs can be attributed to YLLs (ρ = −0.6929; *P* < .001) as opposed to YLDs (ρ = −0.0412; *P* = .77). States in the South Atlantic division, including Florida and Georgia, tended to have lower incidence, DALY, and mortality rates. Comparatively, states in the West North Central division, such as Nebraska and Kansas, had higher rates than states with similar SDI values.

Among SDI components, high school graduate or higher education status for individuals 18 years and older was negatively correlated with DALYs and death but positively correlated with prevalence. The TFU25 rate was positively correlated with all 3 measures, whereas income per capita was negatively correlated with all 3 measures. The East South Central division, including Mississippi, Alabama, and Tennessee, had higher rates than expected based on the general trend for academic achievement and income per capita.

Regional variations were observed when plotting by US division and SDI. Prevalence was higher in divisions with a higher SDI (Figure 4B); the South Atlantic division had lower prevalence rates than the general trend. Age-standardized DALY rates generally decreased as the SDI increased. The East South Central and West North Central divisions displayed higher DALY rates than expected for the corresponding SDI. The mortality rate showed a similar relationship with the SDI.

The CNS cancer estimates (incidence, DALYs, death, prevalence, YLDs, and YLLs) for the years 1990 to 2021 and corresponding figures are available in eAppendixes 4 and 5 in Supplement 1, as well as online through the GBD Results Tool.

## Discussion

In the GBD 2021, CNS cancers were newly defined to include malignant neoplasms of other endocrine glands and the associated structures. This study presents the first detailed analysis of epidemiologic trends in and disease burden of CNS cancer by state in the US under this revised definition. These findings could clarify public health challenges, guiding medical resource reallocation and health care policy for CNS cancer at the state level in the US.

The observations from this study regarding the geographic variations in the CNS cancer burden aligned with registry-based studies reporting on data from 2010 to 2014, with incidence and mortality rates per 100 000 population ranging from 4.86 to 8.0 and 2.93 to 5.64, respectively.^21^ Several studies suggest that biological factors, environmental carcinogens (such as ionizing radiation), and socioeconomic factors are associated with CNS cancer development.^22,23^ Genetic predisposition has been associated with many CNS tumors, including established genetic tumor syndromes and noncoding variants. Racial and ethnic disparities potentially exist, as the incidence rate of glioblastoma was twice as high in White people as it was in Black people and was much higher in Hispanic individuals from 2016 to 2020.^24^ These disparities may partially reflect sociodemographic differences and inequities in access to health care, which contribute to the detection of CNS cancer.^25,26^ Moreover, the accessibility of medical infrastructure further impacts diagnostic rates. The observed geographic diversity underscores the need for targeted public health policies that account for social, biological, and environmental factors.

To understand sex-specific differences, we found that males had a higher CNS cancer burden and incidence in all age groups from 30 to 34 years up to 85 to 89 years. This contrasts with US registry data showing a higher incidence rate in females,^27^ a discrepancy likely due to methodological differences such as age-standardization methods and data modeling, which may yield sex-specific variations in case attribution. Part of this discrepancy may also stem from how CNS cancers are defined across datasets (eAppendix 6 in Supplement 1). Although less common than nonmalignant types,^1^ malignant neoplasms are more lethal and have a higher incidence in males than females (8.06 vs 5.84 per 100 000, respectively) based on US data from 2017 to 2021.^27^ For instance, glioblastoma, which accounts for 51.5% of malignant CNS brain tumors, had a 1.6-fold higher incidence in males.^27^ However, females have a higher survival rate for glioblastoma.^27,28^ CNS cancers demonstrate measurable sex disparities in disease progression and therapeutic response, often attributed to differences in the timing of mutations, metabolic requirements, and immune landscape.^29,30^ Therefore, sex differences in CNS cancers should be noted in future studies.^31^ Sex-specific health policies, such as health checkups that include brain imaging or tumor marker screening, may be warranted in at-risk groups to aid early identification.

This study revealed differing temporal trends across age groups. CNS cancer is the most common tumor in children in the US.^24,32^ However, we observed a significant decrease in incidence rate among children younger than 5 years between 1990 and 2021 (−23.76%; 95% UI, −34.42% to −11.56%). Improved prenatal care and maternal health may be 1 of the major contributors to this decline.^33^ In international studies, pediatric CNS cancers were associated with prenatal alcohol use, advanced maternal age, and low body mass index.^34,35^ In the US, 88.1% of women received “at least adequate” prenatal care, as defined by the Adequacy of Prenatal Care Utilization Index, with a 0.8% increase in first trimester prenatal care from 1980 to 2016.^36^ However, the US has yet to establish tailored policies for populations with lower educational attainment or higher-order births, both of which are critical for sustaining the reduction of pediatric CNS cancer burden.^36,37^

In contrast to the younger groups, our study found that the older groups (>70 years) experienced increasing incidence, DALYs, and mortality rates.^38^ Over the past 6 decades, US life expectancy has increased by 9 years (from 69.9 to 78.9 years).^39^ As the population has aged, the overall incidence of CNS cancer has risen, and the majority of CNS cancer has shifted to glioblastoma, meningioma, and pituitary tumors.^24,32^ Another plausible explanation is the higher rate of medical utilization in older groups.^40^ The frequent use of neuroimaging, such as computed tomography scans for conditions like stroke or head trauma, likely increases the incidental detection of asymptomatic CNS tumors. This, combined with increased medical access for those older than 70 years, contributes to higher reported incidence rates and more accurate documentation of CNS cancer-related deaths.

This study also found a significant negative association between SDI and DALYs and mortality rates. Furthermore, subgroup analysis revealed that high education levels and income per capita were negatively correlated with DALYs and mortality rates, whereas higher education levels were positively correlated with prevalence. Population-based studies from the US and Europe have reported higher survival in patients with higher education levels and higher incidence rates of CNS cancer among those with higher socioeconomic status.^41,42,43^ The significant financial and logistical demands of CNS cancer treatment likely drive these disparities, as care requires a multidisciplinary approach with advanced and expensive technology.^44^ These characteristics therefore highlight the need for national- or state-level support, such as community-based cancer care programs with financial support.

Between 2016 and 2020, the incidence, DALYs, and mortality rates of CNS cancer have steadily declined. However, this trend shifted between 2020 and 2021, with incidence and DALYs decreasing by only 0.289% and 0.717%, respectively. Studies have shown that COVID-19 disrupted all standardized processes from diagnosis to treatment: tumor volumes increased at initial diagnosis and survival decreased, especially in malignant CNS cancers.^45,46^ In contrast, the monthly incidence declined, especially for nonmalignant CNS cancers.^47^ Only 2 years of the COVID-19 pandemic period were evaluated in this study; the significance and permanence of these effects requires further studies, considering potential changes in health care utilization and resource availability.

### Limitations

This study has several limitations. GBD data exhibit quality variations across states and divisions, which may introduce information bias, such as measurement error in reported cases or outcomes.^9^ This study relies on the epidemiological estimates from the GBD 2021, derived from a model using gathered data and published findings. The radiological diagnostic challenges in CNS cancer and the GBD classification by anatomical site prevent histology-specific disaggregation by tumor subtype, grade, or stage. Thus, our findings represent a population-level average across this heterogeneous disease. The heterogeneity of CNS cancers limits identifying precise risk factors and allows only broad associations with covariates. Additionally, the absence of subtype-specific data restricts conclusions regarding targeted treatment and prevention strategies for malignancies like glioblastoma or nonmalignant tumors such as meningioma. Moreover, GBD data lack granular demographics like race and ethnicity, precluding investigation into how these factors correlate with geographic or SDI-related disparities. Future research should prioritize improving data collection frameworks to reduce biases and enhance regional comparability.

## Conclusions

To our knowledge, this is the first comprehensive analysis to focus on newly defined CNS cancers across the US and provide age-, sex-, geographic-, and SDI-stratified estimates and 30-year trends, lending valuable insight into disease trends and risk factors. These findings may help assess the public health landscape and inform health policy and resources reallocation for CNS cancer in the US.

## Notes

Supplement 1. **eAppendix 1.** Supplementary methods **eAppendix 2.** Definition of CNS cancers **eAppendix 3.** US CNS cancer input sources **eAppendix 4.** Supplementary tables **eAppendix 5.** Supplementary figures **eAppendix 6.** Comparison of GBD 2021 estimate and national report methodologies with plausible reasons behind the discrepancies between them

Supplement 2. Data sharing statement
