# Supplementary material for: Burden of Central Nervous System Cancer in the United States, 1990-2021
Source: JAMA Neurol. 2025 Nov 3;83(1):35–48. doi: 10.1001/jamaneurol.2025.4286 (PMC12584065; doi:10.1001/jamaneurol.2025.4286)
Supplement: Supplement 1. — eAppendix 1. Supplementary methods eAppendix 2. Definition of CNS cancers eAppendix 3. US CNS cancer input sources eAppendix 4. Supplementary tables eAppendix 5. Supplementary figures eAppendix 6. Comparison of GBD 2021 estimate and national report methodologies with plausible reasons behind the discrepancies between them [file jamaneurol-e254286-s001.pdf]

Supplemental Online Content

GBD 2021 US CNS Cancer Collaborators. Burden of central nervous system cancer in the United States, 1990-2021. *JAMA Neurol*. Published online November 3, 2025. doi:10.1001/jamaneurol.2025.4286

eAppendix 1. Supplementary methods
eAppendix 2. Definition of CNS cancers
eAppendix 3. US CNS cancer input sources
eAppendix 4. Supplementary tables
eAppendix 5. Supplementary figures
eAppendix 6. Comparison of GBD 2021 estimate and national report methodologies with plausible reasons behind the discrepancies between them

This supplemental material has been provided by the authors to give readers additional information about their work.

© 2025 GBD 2021 US CNS Cancer Collaborators. *JAMA Neurology*.

List of Supplementary Tables and Figures

Appendix I: Supplementary methods 4

eTable 1: GATHER checklist 4

Appendix II: Definition of CNS cancers 6

Appendix III: US CNS cancer input sources 8

Appendix IV: Supplementary Tables 14

eTable 2a: Incidence count and age-standardized rate of Brain and central nervous system cancer by year and sex and percentage change from 1990 to 2021, United States 14

eTable 2b: DALY count and age-standardized rate of Brain and central nervous system cancer by year and sex, and percentage change from 1990 to 2021, United States 16

eTable 2c: Death count and age-standardized rate of Brain and central nervous system cancer by year and sex, and percentage change from 1990 to 2021, United States 18

eTable 2d: Prevalence count and age-standardized rate of Brain and central nervous system cancer by year and sex, and percentage change from 1990 to 2021, United States 20

eTable 2e: YLD count and age-standardized rate of Brain and central nervous system cancer by year and sex, and percentage change from 1990 to 2021, United States 22

eTable 2f: YLL count and age-standardized rate of Brain and central nervous system cancer by year and sex, and percentage change from 1990 to 2021, United States 24

eTable 3a: Incidence count and age-standardized rate of Brain and central nervous system cancer by state and sex, 2021, and percentage change from 1990 to 2021, United States 26

eTable 3b: DALY count and age-standardized rate of Brain and central nervous system cancer by state and sex, 2021, and percentage change from 1990 to 2021, United States 29

eTable 3c: Death count and age-standardized rate of Brain and central nervous system cancer by state and sex, 2021, and percentage change from 1990 to 2021, United States 32

eTable 3d: Prevalence count and age-standardized rate of Brain and central nervous system cancer by state and sex, 2021, and percentage change from 1990 to 2021, United States 35

eTable 3e: YLD count and age-standardized rate of Brain and central nervous system cancer by state and sex, 2021, and percentage change from 1990 to 2021, United States 38

eTable 3f: YLL count and age-standardized rate of Brain and central nervous system cancer by state and sex, 2021, and percentage change from 1990 to 2021, United States 41

eTable 4a: Incidence, DALYs rate of Brain and central nervous system cancer by age and sex, 1990-2021, United States 44

eTable 4b: Death, prevalence rate of Brain and central nervous system cancer by age and sex, 1990-2021, United States 46

eTable 4c: YLD, YLL rate of Brain and central nervous system cancer by age and sex, 1990-2021, United States 48

eTable 5a: Incidence, DALY, death rate of Brain and central nervous system cancer by age, both sexes, 1990-2021, United States 50

eTable 5b: Prevalence, YLD, YLL rate of Brain and central nervous system cancer by age, both sexes, 1990-2021, United States 52

eTable 6: Prevalence, YLD, YLL count and age-standardized rate of Brain and central nervous system cancer by state, 2021, and percentage change from 1990 to 2021, United States 54

eTable 7a: Correlation of Incidence, DALYs, death count and age-standardized rate of Brain and central nervous system cancer by state, 2021, and percentage change from 1990 to 2021 with SDI, United States 58

eTable 7b: Correlation of Prevalence, YLD, YLL count and age-standardized rate of Brain and central nervous system cancer by state, 2021, and percentage change from 1990 to 2021 with SDI, United States 62

eTable 8: Correlation of age-standardized incidence, DALYs, death, prevalence, YLD, YLL rate of Brain and central nervous system cancer by state with SDI related factors, 2021, United States 66

eTable 9a: Age-standardized incidence rate of Brain and central nervous system cancer by state and year, and percentage change from 1990 to 2021, United States 68

eTable 9b: Age-standardized DALY rate of Brain and central nervous system cancer by state and year, and percentage change from 1990 to 2021, United States 71

eTable 9c: Age-standardized death rate of Brain and central nervous system cancer by state and year, and percentage change from 1990 to 2021, United States 75

eTable 9d: Age-standardized prevalence rate of Brain and central nervous system cancer by state and year, and percentage change from 1990 to 2021, United States 78

© 2025 GBD 2021 US CNS Cancer Collaborators. *JAMA Neurology*.

eTable 9e: Age-standardized YLD rate of Brain and central nervous system cancer by state and year, and percentage change from 1990 to 2021, United States ... 81

eTable 9f: Age-standardized YLL rate of Brain and central nervous system cancer by state and year, and percentage change from 1990 to 2021, United States ... 84

Appendix V: Supplementary Figures ... 88

eFigure 1a: (A) incidence (B) DALYs (C) death (D) prevalence (E) YLDs (F) YLLs count and age-standardized rate of Brain and central nervous system cancer in the United States, 1990 to 2021 ... 89

eFigure 1b: Incidence count and age-standardized rate of Brain and central nervous system cancer in the United States by division, (A) New England, (B) Middle Atlantic, (C) East North Central, (D) West North Central, (E) South Atlantic, (F) East South Central, (G) West South Central, (H) Mountain, (I) Pacific division, 1990 to 2021 ... 96

eFigure 1c: DALYs count and age-standardized rate of Brain and central nervous system cancer in the United States by division, (A) New England, (B) Middle Atlantic, (C) East North Central, (D) West North Central, (E) South Atlantic, (F) East South Central, (G) West South Central, (H) Mountain, (I) Pacific division, 1990 to 2021 ... 103

eFigure 1d: Death count and age-standardized rate of Brain and central nervous system cancer in the United States by division, (A) New England, (B) Middle Atlantic, (C) East North Central, (D) West North Central, (E) South Atlantic, (F) East South Central, (G) West South Central, (H) Mountain, (I) Pacific division, 1990 to 2021 ... 110

eFigure 1e: Prevalence count and age-standardized rate of Brain and central nervous system cancer in the United States by division, (A) New England, (B) Middle Atlantic, (C) East North Central, (D) West North Central, (E) South Atlantic, (F) East South Central, (G) West South Central, (H) Mountain, (I) Pacific division, 1990 to 2021 ... 117

eFigure 1f: YLDs count and age-standardized rate of Brain and central nervous system cancer in the United States by division, (A) New England, (B) Middle Atlantic, (C) East North Central, (D) West North Central, (E) South Atlantic, (F) East South Central, (G) West South Central, (H) Mountain, (I) Pacific division, 1990 to 2021 ... 124

eFigure 1g: YLLs count and age-standardized rate of Brain and central nervous system cancer in the United States by division, (A) New England, (B) Middle Atlantic, (C) East North Central, (D) West North Central, (E) South Atlantic, (F) East South Central, (G) West South Central, (H) Mountain, (I) Pacific division, 1990 to 2021 ... 131

eFigure 2: (A) prevalence (B) YLDs (C) YLLs count and age-standardized rate of Brain and central nervous system cancer by sex, United States, 1990 to 2021 ... 132

eFigure 3: (A) Death (B) prevalence (C) YLDs (D) YLLs count and rate of Brain and central nervous system cancer by sex and age, United States, 2021 ... 133

eFigure 4: (A) incidence (B) DALYs (C) death (D) prevalence (E) YLDs (F) YLLs count and rate of Brain and central nervous system cancer by sex and age, United States, 1990 ... 135

eFigure 5: (A) incidence (B) DALYs (C) death (D) prevalence (E) YLDs (F) YLLs count and rate of Brain and central nervous system cancer by age, United States, 1990~2021 ... 137

eFigure 6: Age-standardized (A) Death (B) prevalence (C) YLDs (D) YLLs rate of Brain and central nervous system cancer in the United States, 2021 ... 138

eFigure 7: Change in age-standardized (A) incidence (B) DALYs (C) death (D) prevalence (E) YLDs (F) YLLs rate of Brain and central nervous system cancer in the United States, 1990-2021 ... 140

eFigure 8: Correlation between SDI and age-standardized (A) prevalence (B) YLDs (C) YLLs rate of Brain and central nervous system cancer in the United States by state, 2021 ... 142

eFigure 9: Correlation between Fertility rate below 25 and age-standardized (A) incidence (B) DALYs (C) death (D) prevalence (E) YLDs (F) YLLs rate of Brain and central nervous system cancer in the United States by state, 2021 ... 144

eFigure 10: Correlation between academic achievement and age-standardized (A) incidence (B) DALYs (C) death (D) prevalence (E) YLDs (F) YLLs rate of Brain and central nervous system cancer in the United States by state, 2021 ... 146

eFigure 11: Correlation between income per capita and age-standardized (A) incidence (B) DALYs (C) death (D) prevalence (E) YLDs (F) YLLs rate of Brain and central nervous system cancer in the United States by state, 2021 ... 148

eFigure 12: Correlation between SDI and age-standardized (A) prevalence (B) YLDs (C) YLLs rate of Brain and central nervous system cancer in the United States by division, 2021 ... 149

Appendix VI: Comparison of GBD 2021 estimate and national report methodologies with plausible reasons behind the discrepancies between them ... 148

© 2025 GBD 2021 US CNS Cancer Collaborators. *JAMA Neurology*.

eAppendix I: Supplementary methods

eTable 1: GATHER checklist

#	Checklist item	Section/paragraph/ interpretation
Objectives and funding		
1	Define the indicators, populations, and time periods for which estimates were made.	Methods / "Overview of study and data" sections
2	List the funding sources for the work.	No funding
Data Inputs		
For all data inputs from multiple sources that are synthesized as part of the study:		
3	Describe how the data were identified and how the data were accessed.	Methods /"Overview of study and data " section, the details have been published previously.
4	Specify the inclusion and exclusion criteria. Identify all ad-hoc exclusions.	Methods / "Overview of study and data" section
5	Provide information on all included data sources and their main characteristics. For each data source used, report reference information or contact name/institution, population represented, data collection method, year(s) of data collection, sex and age range, diagnostic criteria or measurement method, and sample size, as relevant.	Available via online data source tools (http://ghdx.healthdata.org/gbd-2021/data-input-sources).
6	Identify and describe any categories of input data that have potentially important biases (e.g., based on characteristics listed in item 5).	Methods / "Overview of study and data", the details have been published previously.
For data inputs that contribute to the analysis but were not synthesized as part of the study:		
7	Describe and give sources for any other data inputs.	Available via online data source tools (http://ghdx.healthdata.org/gbd-2021/data-input-sources).
For all data inputs:		
8	Provide all data inputs in a file format from which data can be efficiently extracted (e.g., a spreadsheet as opposed to a PDF), including all relevant meta-data listed in item 5. For any data inputs that cannot be shared due to ethical or legal reasons, such as third-party ownership, provide a contact name or the name of the institution that retains the right to the data.	Available via online data source tools (http://ghdx.healthdata.org/gbd-2021/data-input-sources); input data not available in tools will be made available upon request.
Data analysis		
9	Provide a conceptual overview of the data analysis method. A diagram may be helpful.	Flow diagrams of the overall methodological processes were available online (http://ghdx.healthdata.org/gbd-2021/code)
10	Provide a detailed description of all steps of the analysis, including mathematical formulae. This description should cover, as relevant, data cleaning, data pre-processing, data adjustments and weighting of data sources, and mathematical or statistical model(s).	Methods / "GBD estimation framework and metrics" and "SDI" sections, the details have been published previously.
11	Describe how candidate models were evaluated and how the final model(s) were selected.	Methods / "GBD estimation framework and metrics" section, the details have been published previously.
12	Provide the results of an evaluation of model performance, if done, as well as the results of any relevant sensitivity analysis.	Methods / "GBD estimation framework and metrics", "SDI", and " Statistical Analysis" sections, the details have been published previously.
13	Describe methods for calculating uncertainty of the estimates. State which sources of uncertainty were, and were not, accounted for in the uncertainty analysis.	Methods / "GBD estimation framework and metrics" and " Statistical Analysis" sections, the details have been published previously.
14	State how analytic or statistical source code used to generate estimates can be accessed.	Methods / "GBD estimation framework and metrics" and "SDI" sections
Results and Discussion		
15	Provide published estimates in a file format from which data can be efficiently extracted.	Results, and online data tools (data visualization tools, and data query tools, http://ghdx.healthdata.org/gbd-2021)
16	Report a quantitative measure of the uncertainty of the estimates (e.g. uncertainty intervals).	Results, and online data tools (data visualization tools, and data query tools, http://ghdx.healthdata.org/gbd-2021)
17	Interpret results in light of existing evidence. If updating a previous set of estimates, describe the reasons for changes in estimates.	Discussion

© 2025 GBD 2021 US CNS Cancer Collaborators. *JAMA Neurology*.

18	Discuss limitations of the estimates. Include a discussion of any modelling assumptions or data limitations that affect interpretation of the estimates.	Discussion

© 2025 GBD 2021 US CNS Cancer Collaborators. *JAMA Neurology*.

eAppendix II: Definition of CNS cancers

The International Classification of Diseases, Tenth Edition, Clinical Modification of CNS cancers (C70-C72.9 and C75.1-C75.3) were used to collect data sources.

**C70 Malignant neoplasm of meninges**	**C70.0 Cerebral meninges**<br>**C70.1 Spinal meninges**<br>**C70.9 Meninges, unspecified**
**C71 Malignant neoplasm of brain**	C71.0 Cerebrum, except lobes and ventricles<br>**Supratentorial NOS**<br>C71.1Frontal lobe<br>C71.2Temporal lobe<br>C71.3Parietal lobe<br>C71.4Occipital lobe<br>C71.5Cerebral ventricle<br>***Excl.:*** **fourth ventricle (C71.7)**<br>C71.6Cerebellum<br>C71.7Brain stem<br>**Fourth ventricle**<br>**Infratentorial NOS**<br>C71.8Overlapping lesion of brain<br>**[See note 5 at the beginning of this chapter]**<br>C71.9Brain, unspecified
**C72 Malignant neoplasm of spinal cord, cranial nerves and other parts of central nervous**	C72.0Spinal cord<br>C72.1Cauda equina<br>C72.2Olfactory nerve<br>**Olfactory bulb**<br>C72.3Optic nerve<br>C72.4Acoustic nerve<br>C72.5Other and unspecified cranial nerves<br>**Cranial nerve NOS**<br>C72.8Overlapping lesion of brain and other parts of central nervous system<br>**[See note 5 at the beginning of this chapter]**<br>**Malignant neoplasm of brain and other parts of central nervous system whose point of origin cannot be classified to any one of the categories C70-C72.5**

© 2025 GBD 2021 US CNS Cancer Collaborators. *JAMA Neurology*.

	C72.9Central nervous system, unspecified<br>**Nervous system NOS**
**C75 Malignant neoplasm of other endocrine glands and related structures**	C75.0Parathyroid gland<br><br>C75.1Pituitary gland<br><br>C75.2Craniopharyngeal duct<br><br>C75.3Pineal gland

Reference.
World Health Organization. (2004). ICD-10 : international statistical classification of diseases and related health problems : tenth revision, 2nd ed. World Health Organization. https://iris.who.int/handle/10665/42980

© 2025 GBD 2021 US CNS Cancer Collaborators. *JAMA Neurology*.

eAppendix III: US CNS cancer input sources

Institute for Health Metrics and Evaluation (IHME). IHME GBD Cancer Incidence and Mortality Estimates.
United States - Alabama Cancer Registry 1998-2002 - CI5. as it appears in Cancer Incidence in Five Continents Volume IX Periodic Data 1998-2002
United States - Alabama Cancer Registry 2003-2007 - CI5. as it appears in Cancer Incidence in Five Continents Volume X Summary Database 2003-2007
United States - Alabama Cancer Registry 2008-2012 - CI5.
United States - Alameda County Cancer Registry 1983-1987 - CI5. as it appears in Cancer Incidence in Five Continents Volumes I-VIII 1950-1997
United States - Arizona Cancer Registry 1998-2002 - CI5. as it appears in Cancer Incidence in Five Continents Volume IX Periodic Data 1998-2002
United States - Arizona Cancer Registry 2003-2007 - CI5. as it appears in Cancer Incidence in Five Continents Volume X Summary Database 2003-2007
United States - Arizona Cancer Registry 2008-2012 - CI5.
United States - Arkansas Cancer Registry 2003-2007 - CI5. as it appears in Cancer Incidence in Five Continents Volume X Summary Database 2003-2007
United States - Arkansas Cancer Registry 2008-2012 - CI5.
United States - California Cancer Registry 1998-2002 - CI5. as it appears in Cancer Incidence in Five Continents Volume IX Periodic Data 1998-2002
United States - California Cancer Registry 2003-2007 - CI5. as it appears in Cancer Incidence in Five Continents Volume X Summary Database 2003-2007
United States - California Cancer Registry 2008-2012 - CI5.
United States - Central Louisiana Cancer Registry 1988-1992 - CI5. as it appears in Cancer Incidence in Five Continents Volumes I-VIII 1950-1997
United States - Central Valley Cancer Registry 1988-1992 - CI5. as it appears in Cancer Incidence in Five Continents Volumes I-VIII 1950-1997
United States - Colorado Cancer Registry 1998-2002 - CI5. as it appears in Cancer Incidence in Five Continents Volume IX Periodic Data 1998-2002
United States - Colorado Cancer Registry 2003-2007 - CI5. as it appears in Cancer Incidence in Five Continents Volume X Summary Database 2003-2007
United States - Colorado Cancer Registry 2008-2012 - CI5.
United States - Delaware Cancer Registry 2003-2007 - CI5. as it appears in Cancer Incidence in Five Continents Volume X Summary Database 2003-2007
United States - Delaware Cancer Registry 2008-2012 - CI5.
United States - District of Columbia Cancer Registry 1998-2002 - CI5. as it appears in Cancer Incidence in Five Continents Volume IX Periodic Data 1998-2002
Florida Department of Health. United States - Florida Annual Cancer Report 1995. 1998.
Florida Department of Health. United States - Florida Annual Cancer Report 1996-1997. 1998.
Florida Department of Health. United States - Florida Annual Cancer Report 1998. 1998.
Florida Department of Health. United States - Florida Annual Cancer Report 1999. 1998.
United States - Florida Cancer Registry 1998-2002 - CI5. as it appears in Cancer Incidence in Five Continents Volume IX Periodic Data 1998-2002
United States - Florida Cancer Registry 2003-2007 - CI5. as it appears in Cancer Incidence in Five Continents Volume X Summary Database 2003-2007
United States - Florida Cancer Registry 2008-2012 - CI5.
Georgia Department of Public Health (United States). United States - Georgia Cancer Incidence 2011-2015.
Georgia Department of Public Health (United States). United States - Georgia Cancer Incidence 2012-2016.
United States - Georgia Cancer Registry 1998-2002 - CI5. as it appears in Cancer Incidence in Five Continents Volume IX Periodic Data 1998-2002
United States - Georgia Cancer Registry 2003-2007 - CI5. as it appears in Cancer Incidence in Five Continents Volume X Summary Database 2003-2007
United States - Georgia Cancer Registry 2008-2012 - CI5.
United States - Hawaii Cancer Registry 1968-1972 - CI5. as it appears in Cancer Incidence in Five Continents Volumes I-VIII 1950-1997
United States - Idaho Cancer Registry 1998-2002 - CI5. as it appears in Cancer Incidence in Five Continents Volume IX Periodic Data 1998-2002

© 2025 GBD 2021 US CNS Cancer Collaborators. *JAMA Neurology*.

United States - Idaho Cancer Registry 2003-2007 - CI5. as it appears in Cancer Incidence in Five Continents Volume X Summary Database 2003-2007
United States - Idaho Cancer Registry 2008-2012 - CI5.
United States - Illinois Cancer Registry 1998-2002 - CI5. as it appears in Cancer Incidence in Five Continents Volume IX Periodic Data 1998-2002
United States - Illinois Cancer Registry 2003-2007 - CI5. as it appears in Cancer Incidence in Five Continents Volume X Summary Database 2003-2007
United States - Illinois Cancer Registry 2008-2012 - CI5.
United States - Indiana Cancer Registry 1998-2002 - CI5. as it appears in Cancer Incidence in Five Continents Volume IX Periodic Data 1998-2002
United States - Indiana Cancer Registry 2003-2007 - CI5. as it appears in Cancer Incidence in Five Continents Volume X Summary Database 2003-2007
United States - Indiana Cancer Registry 2008-2012 - CI5.
United States - Los Angeles County Cancer Registry 1973 - CI5. as it appears in Cancer Incidence in Five Continents Time Trends Annual Dataset (Summary and Detailed Databases)
United States - Los Angeles County Cancer Registry 1974 - CI5. as it appears in Cancer Incidence in Five Continents Time Trends Annual Dataset (Summary and Detailed Databases)
United States - Los Angeles County Cancer Registry 1975 - CI5. as it appears in Cancer Incidence in Five Continents Time Trends Annual Dataset (Summary and Detailed Databases)
United States - Los Angeles County Cancer Registry 1976 - CI5. as it appears in Cancer Incidence in Five Continents Time Trends Annual Dataset (Summary and Detailed Databases)
United States - Los Angeles County Cancer Registry 1977 - CI5. as it appears in Cancer Incidence in Five Continents Time Trends Annual Dataset (Summary and Detailed Databases)
United States - Los Angeles County Cancer Registry 1978 - CI5. as it appears in Cancer Incidence in Five Continents Time Trends Annual Dataset (Summary and Detailed Databases)
United States - Los Angeles County Cancer Registry 1979 - CI5. as it appears in Cancer Incidence in Five Continents Time Trends Annual Dataset (Summary and Detailed Databases)
United States - Los Angeles County Cancer Registry 1980 - CI5. as it appears in Cancer Incidence in Five Continents Time Trends Annual Dataset (Summary and Detailed Databases)
United States - Los Angeles County Cancer Registry 1981 - CI5. as it appears in Cancer Incidence in Five Continents Time Trends Annual Dataset (Summary and Detailed Databases)
United States - Los Angeles County Cancer Registry 1982 - CI5. as it appears in Cancer Incidence in Five Continents Time Trends Annual Dataset (Summary and Detailed Databases)
United States - Los Angeles County Cancer Registry 1983 - CI5. as it appears in Cancer Incidence in Five Continents Time Trends Annual Dataset (Summary and Detailed Databases)
United States - Los Angeles County Cancer Registry 1984 - CI5. as it appears in Cancer Incidence in Five Continents Time Trends Annual Dataset (Summary and Detailed Databases)
United States - Los Angeles County Cancer Registry 1985 - CI5. as it appears in Cancer Incidence in Five Continents Time Trends Annual Dataset (Summary and Detailed Databases)
United States - Los Angeles County Cancer Registry 1986 - CI5. as it appears in Cancer Incidence in Five Continents Time Trends Annual Dataset (Summary and Detailed Databases)
United States - Los Angeles County Cancer Registry 1987 - CI5. as it appears in Cancer Incidence in Five Continents Time Trends Annual Dataset (Summary and Detailed Databases)
United States - Los Angeles County Cancer Registry 1988 - CI5. as it appears in Cancer Incidence in Five Continents Time Trends Annual Dataset (Summary and Detailed Databases)
United States - Los Angeles County Cancer Registry 1989 - CI5. as it appears in Cancer Incidence in Five Continents Time Trends Annual Dataset (Summary and Detailed Databases)
United States - Los Angeles County Cancer Registry 1990 - CI5. as it appears in Cancer Incidence in Five Continents Time Trends Annual Dataset (Summary and Detailed Databases)
United States - Los Angeles County Cancer Registry 1991 - CI5. as it appears in Cancer Incidence in Five Continents Time Trends Annual Dataset (Summary and Detailed Databases)
United States - Louisiana Cancer Registry 1993-1997 - CI5. as it appears in Cancer Incidence in Five Continents Volumes I-VIII 1950-1997
United States - Maine Cancer Registry 1998-2002 - CI5. as it appears in Cancer Incidence in Five Continents Volume IX Periodic Data 1998-2002
United States - Maine Cancer Registry 2003-2007 - CI5. as it appears in Cancer Incidence in Five Continents Volume X Summary Database 2003-2007
United States - Maine Cancer Registry 2008-2012 - CI5.
United States - Maryland Cancer Registry 2008-2012 - CI5.
United States - Massachusetts Cancer Registry 1998-2002 - CI5. as it appears in Cancer Incidence in Five Continents Volume IX Periodic Data 1998-2002

© 2025 GBD 2021 US CNS Cancer Collaborators. *JAMA Neurology*.

United States - Massachusetts Cancer Registry 2003-2007 - CI5. as it appears in Cancer Incidence in Five Continents Volume X Summary Database 2003-2007
United States - Massachusetts Cancer Registry 2008-2012 - CI5.
United States - Michigan Cancer Registry 1998-2002 - CI5. as it appears in Cancer Incidence in Five Continents Volume IX Periodic Data 1998-2002
United States - Michigan Cancer Registry 2003-2007 - CI5. as it appears in Cancer Incidence in Five Continents Volume X Summary Database 2003-2007
United States - Michigan Cancer Registry 2008-2012 - CI5.
United States - Minnesota Cancer Registry 2008-2012 - CI5.
United States - Mississippi Cancer Registry 2003-2007 - CI5. as it appears in Cancer Incidence in Five Continents Volume X Summary Database 2003-2007
United States - Mississippi Cancer Registry 2008-2012 - CI5.
United States - Missouri Cancer Registry 1998-2002 - CI5. as it appears in Cancer Incidence in Five Continents Volume IX Periodic Data 1998-2002
United States - Missouri Cancer Registry 2003-2007 - CI5. as it appears in Cancer Incidence in Five Continents Volume X Summary Database 2003-2007
United States - Missouri Cancer Registry 2008-2012 - CI5.
United States - Montana Cancer Registry 1998-2002 - CI5. as it appears in Cancer Incidence in Five Continents Volume IX Periodic Data 1998-2002
United States - Montana Cancer Registry 2003-2007 - CI5. as it appears in Cancer Incidence in Five Continents Volume X Summary Database 2003-2007
United States - Montana Cancer Registry 2008-2012 - CI5.
United States - Nebraska Cancer Registry 2003-2007 - CI5. as it appears in Cancer Incidence in Five Continents Volume X Summary Database 2003-2007
United States - Nebraska Cancer Registry 2008-2012 - CI5.
United States - Nevada Cancer Registry 2008-2012 - CI5.
United States - New Hampshire Cancer Registry 2003-2007 - CI5. as it appears in Cancer Incidence in Five Continents Volume X Summary Database 2003-2007
United States - New Hampshire Cancer Registry 2008-2012 - CI5.
United States - New Jersey Cancer Registry 1993-1997 - CI5. as it appears in Cancer Incidence in Five Continents Volumes I-VIII 1950-1997
United States - New Mexico Cancer Registry 1969-1972 - CI5. as it appears in Cancer Incidence in Five Continents Volume III
United States - New Orleans Cancer Registry 1983 - CI5. as it appears in Cancer Incidence in Five Continents Time Trends Annual Dataset (Summary and Detailed Databases)
United States - New Orleans Cancer Registry 1984 - CI5. as it appears in Cancer Incidence in Five Continents Time Trends Annual Dataset (Summary and Detailed Databases)
United States - New Orleans Cancer Registry 1985 - CI5. as it appears in Cancer Incidence in Five Continents Time Trends Annual Dataset (Summary and Detailed Databases)
United States - New Orleans Cancer Registry 1986 - CI5. as it appears in Cancer Incidence in Five Continents Time Trends Annual Dataset (Summary and Detailed Databases)
United States - New Orleans Cancer Registry 1987 - CI5. as it appears in Cancer Incidence in Five Continents Time Trends Annual Dataset (Summary and Detailed Databases)
United States - New Orleans Cancer Registry 1988 - CI5. as it appears in Cancer Incidence in Five Continents Time Trends Annual Dataset (Summary and Detailed Databases)
United States - New Orleans Cancer Registry 1989 - CI5. as it appears in Cancer Incidence in Five Continents Time Trends Annual Dataset (Summary and Detailed Databases)
United States - New Orleans Cancer Registry 1991 - CI5. as it appears in Cancer Incidence in Five Continents Time Trends Annual Dataset (Summary and Detailed Databases)
United States - New Orleans Cancer Registry 1992 - CI5. as it appears in Cancer Incidence in Five Continents Time Trends Annual Dataset (Summary and Detailed Databases)
United States - New Orleans Cancer Registry 1993 - CI5. as it appears in Cancer Incidence in Five Continents Time Trends Annual Dataset (Summary and Detailed Databases)
United States - New Orleans Cancer Registry 1994 - CI5. as it appears in Cancer Incidence in Five Continents Time Trends Annual Dataset (Summary and Detailed Databases)
United States - New Orleans Cancer Registry 1996 - CI5. as it appears in Cancer Incidence in Five Continents Time Trends Annual Dataset (Summary and Detailed Databases)
United States - New Orleans Cancer Registry 1997 - CI5. as it appears in Cancer Incidence in Five Continents Time Trends Annual Dataset (Summary and Detailed Databases)
United States - New Orleans Cancer Registry 1998 - CI5. as it appears in Cancer Incidence in Five Continents Time Trends Annual Dataset (Summary and Detailed Databases)

© 2025 GBD 2021 US CNS Cancer Collaborators. *JAMA Neurology*.

United States - New Orleans Cancer Registry 1999 - CI5. as it appears in Cancer Incidence in Five Continents Time Trends Annual Dataset (Summary and Detailed Databases)
United States - New York Cancer Registry 1993-1997 - CI5. as it appears in Cancer Incidence in Five Continents Volumes I-VIII 1950-1997
United States - New York Cancer Registry 1998-2002 - CI5. as it appears in Cancer Incidence in Five Continents Volume IX Periodic Data 1998-2002
United States - New York Cancer Registry 2003-2007 - CI5. as it appears in Cancer Incidence in Five Continents Volume X Summary Database 2003-2007
United States - New York Cancer Registry 2008-2012 - CI5.
United States - North Carolina Cancer Registry 2003-2007 - CI5. as it appears in Cancer Incidence in Five Continents Volume X Summary Database 2003-2007
United States - North Carolina Cancer Registry 2008-2012 - CI5.
United States - North Dakota Cancer Registry 2003-2007 - CI5. as it appears in Cancer Incidence in Five Continents Volume X Summary Database 2003-2007
United States - North Dakota Cancer Registry 2008-2012 - CI5.
United States - Ohio Cancer Registry 1998-2002 - CI5. as it appears in Cancer Incidence in Five Continents Volume IX Periodic Data 1998-2002
United States - Ohio Cancer Registry 2003-2007 - CI5. as it appears in Cancer Incidence in Five Continents Volume X Summary Database 2003-2007
United States - Ohio Cancer Registry 2008-2012 - CI5.
United States - Oklahoma Cancer Registry 1998-2002 - CI5. as it appears in Cancer Incidence in Five Continents Volume IX Periodic Data 1998-2002
United States - Oklahoma Cancer Registry 2003-2007 - CI5. as it appears in Cancer Incidence in Five Continents Volume X Summary Database 2003-2007
United States - Oklahoma Cancer Registry 2008-2012 - CI5.
United States - Oregon Cancer Registry 1998-2002 - CI5. as it appears in Cancer Incidence in Five Continents Volume IX Periodic Data 1998-2002
United States - Oregon Cancer Registry 2003-2007 - CI5. as it appears in Cancer Incidence in Five Continents Volume X Summary Database 2003-2007
United States - Oregon Cancer Registry 2008-2012 - CI5.
United States - Pennsylvania Cancer Registry 1998-2002 - CI5. as it appears in Cancer Incidence in Five Continents Volume IX Periodic Data 1998-2002
United States - Pennsylvania Cancer Registry 2003-2007 - CI5. as it appears in Cancer Incidence in Five Continents Volume X Summary Database 2003-2007
United States - Pennsylvania Cancer Registry 2008-2012 - CI5.
United States - Rhode Island Cancer Registry 1998-2002 - CI5. as it appears in Cancer Incidence in Five Continents Volume IX Periodic Data 1998-2002
United States - Rhode Island Cancer Registry 2003-2007 - CI5. as it appears in Cancer Incidence in Five Continents Volume X Summary Database 2003-2007
United States - Rhode Island Cancer Registry 2008-2012 - CI5.
United States - South Carolina Cancer Registry 1998-2002 - CI5. as it appears in Cancer Incidence in Five Continents Volume IX Periodic Data 1998-2002
United States - South Carolina Cancer Registry 2003-2007 - CI5. as it appears in Cancer Incidence in Five Continents Volume X Summary Database 2003-2007
United States - South Carolina Cancer Registry 2008-2012 - CI5.
United States - South Dakota Cancer Registry 2003-2007 - CI5. as it appears in Cancer Incidence in Five Continents Volume X Summary Database 2003-2007
United States - South Dakota Cancer Registry 2008-2012 - CI5.
United States - Tennessee Cancer Registry 2003-2007 - CI5. as it appears in Cancer Incidence in Five Continents Volume X Summary Database 2003-2007
United States - Tennessee Cancer Registry 2008-2012 - CI5.
United States - Texas Cancer Registry 1998-2002 - CI5. as it appears in Cancer Incidence in Five Continents Volume IX Periodic Data 1998-2002
United States - Texas Cancer Registry 2003-2007 - CI5. as it appears in Cancer Incidence in Five Continents Volume X Summary Database 2003-2007
United States - Texas Cancer Registry 2008-2012 - CI5.
United States - Vermont Cancer Registry 1998-2002 - CI5. as it appears in Cancer Incidence in Five Continents Volume IX Periodic Data 1998-2002

© 2025 GBD 2021 US CNS Cancer Collaborators. *JAMA Neurology*.

United States - Vermont Cancer Registry 2003-2007 - CI5. as it appears in Cancer Incidence in Five Continents Volume X Summary Database 2003-2007
United States - Vermont Cancer Registry 2008-2012 - CI5.
United States - Virginia Cancer Registry 2003-2007 - CI5. as it appears in Cancer Incidence in Five Continents Volume X Summary Database 2003-2007
United States - Virginia Cancer Registry 2008-2012 - CI5.
United States - Washington Cancer Registry 1998-2002 - CI5. as it appears in Cancer Incidence in Five Continents Volume IX Periodic Data 1998-2002
United States - Washington Cancer Registry 2003-2007 - CI5. as it appears in Cancer Incidence in Five Continents Volume X Summary Database 2003-2007
United States - Washington Cancer Registry 2008-2012 - CI5.
United States - West Virginia Cancer Registry 1998-2002 - CI5. as it appears in Cancer Incidence in Five Continents Volume IX Periodic Data 1998-2002
United States - West Virginia Cancer Registry 2003-2007 - CI5. as it appears in Cancer Incidence in Five Continents Volume X Summary Database 2003-2007
United States - West Virginia Cancer Registry 2008-2012 - CI5.
United States - Wisconsin Cancer Registry 1998-2002 - CI5. as it appears in Cancer Incidence in Five Continents Volume IX Periodic Data 1998-2002
United States - Wisconsin Cancer Registry 2003-2007 - CI5. as it appears in Cancer Incidence in Five Continents Volume X Summary Database 2003-2007
United States - Wisconsin Cancer Registry 2008-2012 - CI5.
United States - Wyoming Cancer Registry 2003-2007 - CI5. as it appears in Cancer Incidence in Five Continents Volume X Summary Database 2003-2007
United States - Wyoming Cancer Registry 2008-2012 - CI5.
National Center for Health Statistics, Centers for Disease Control and Prevention, U.S. Department of Defense. United States NVSS Mortality Data 1980 - NBER and United States Military Deaths 1980-2014.
National Center for Health Statistics, Centers for Disease Control and Prevention, U.S. Department of Defense. United States NVSS Mortality Data 1981 - NBER and United States Military Deaths 1980-2014.
National Center for Health Statistics, Centers for Disease Control and Prevention, U.S. Department of Defense. United States NVSS Mortality Data 1982 - NBER and United States Military Deaths 1980-2014.
National Center for Health Statistics, Centers for Disease Control and Prevention, U.S. Department of Defense. United States NVSS Mortality Data 1983 - NBER and United States Military Deaths 1980-2014.
National Center for Health Statistics, Centers for Disease Control and Prevention, U.S. Department of Defense. United States NVSS Mortality Data 1984 - NBER and United States Military Deaths 1980-2014.
National Center for Health Statistics, Centers for Disease Control and Prevention, U.S. Department of Defense. United States NVSS Mortality Data 1985 - NBER and United States Military Deaths 1980-2014.
National Center for Health Statistics, Centers for Disease Control and Prevention, U.S. Department of Defense. United States NVSS Mortality Data 1986 - NBER and United States Military Deaths 1980-2014.
National Center for Health Statistics, Centers for Disease Control and Prevention, U.S. Department of Defense. United States NVSS Mortality Data 1987 - NBER and United States Military Deaths 1980-2014.
National Center for Health Statistics, Centers for Disease Control and Prevention, U.S. Department of Defense. United States NVSS Mortality Data 1988 - NBER and United States Military Deaths 1980-2014.
National Center for Health Statistics, Centers for Disease Control and Prevention, U.S. Department of Defense. United States NVSS Mortality Data 1989 - NBER and United States Military Deaths 1980-2014.
National Center for Health Statistics, Centers for Disease Control and Prevention, U.S. Department of Defense. United States NVSS Mortality Data 1990 - NBER and United States Military Deaths 1980-2014.
National Center for Health Statistics, Centers for Disease Control and Prevention, U.S. Department of Defense. United States NVSS Mortality Data 1991 - NBER and United States Military Deaths 1980-2014.
National Center for Health Statistics, Centers for Disease Control and Prevention, U.S. Department of Defense. United States NVSS Mortality Data 1992 - NBER and United States Military Deaths 1980-2014.
National Center for Health Statistics, Centers for Disease Control and Prevention, U.S. Department of Defense. United States NVSS Mortality Data 1993 - NBER and United States Military Deaths 1980-2014.
National Center for Health Statistics, Centers for Disease Control and Prevention, U.S. Department of Defense. United States NVSS Mortality Data 1994 - NBER and United States Military Deaths 1980-2014.
National Center for Health Statistics, Centers for Disease Control and Prevention, U.S. Department of Defense. United States NVSS Mortality Data 1995 - NBER and United States Military Deaths 1980-2014.
National Center for Health Statistics, Centers for Disease Control and Prevention, U.S. Department of Defense. United States NVSS Mortality Data 1996 - NBER and United States Military Deaths 1980-2014.
National Center for Health Statistics, Centers for Disease Control and Prevention, U.S. Department of Defense. United States NVSS Mortality Data 1997 - NBER and United States Military Deaths 1980-2014.

© 2025 GBD 2021 US CNS Cancer Collaborators. *JAMA Neurology*.

National Center for Health Statistics, Centers for Disease Control and Prevention, U.S. Department of Defense. United States NVSS Mortality Data 1998 - NBER and United States Military Deaths 1980-2014.
National Center for Health Statistics, Centers for Disease Control and Prevention, U.S. Department of Defense. United States NVSS Mortality Data 1999 - NBER and United States Military Deaths 1980-2014.
National Center for Health Statistics, Centers for Disease Control and Prevention, U.S. Department of Defense. United States NVSS Mortality Data 2000 - NBER and United States Military Deaths 1980-2014.
National Center for Health Statistics, Centers for Disease Control and Prevention, U.S. Department of Defense. United States NVSS Mortality Data 2001 - NBER and United States Military Deaths 1980-2014.
National Center for Health Statistics, Centers for Disease Control and Prevention, U.S. Department of Defense. United States NVSS Mortality Data 2002 - NBER and United States Military Deaths 1980-2014.
National Center for Health Statistics, Centers for Disease Control and Prevention, U.S. Department of Defense. United States NVSS Mortality Data 2003 - NBER and United States Military Deaths 1980-2014.
National Center for Health Statistics, Centers for Disease Control and Prevention, U.S. Department of Defense. United States NVSS Mortality Data 2004 - NBER and United States Military Deaths 1980-2014.
Surveillance, Epidemiology, and End Results (SEER) Program (www.seer.cancer.gov) Research Data (1973-2008), National Cancer Institute, DCCPS, Surveillance Research Program, Cancer Statistics Branch, released April 2011, based on the November 2010 submission.
Surveillance, Epidemiology, and End Results (SEER) Program (www.seer.cancer.gov) Research Data (1973-2008), National Cancer Institute, DCCPS, Surveillance Research Program, Cancer Statistics Branch, released April 2011, based on the November 2010 submission.
Surveillance, Epidemiology, and End Results (SEER) Program (www.seer.cancer.gov) SEER*Stat Database: Incidence - SEER 18 Regs Research Data + Hurricane Katrina Impacted Louisiana Cases, Nov 2013 Sub (1973-2011 varying) - Linked To County Attributes - Total U.S., 1969-2012 Counties, National Cancer Institute, DCCPS, Surveillance Research Program, Surveillance Systems Branch, released April 2014 (updated 5/7/2014), based on the November 2013 submission.
Surveillance, Epidemiology, and End Results (SEER) Program (www.seer.cancer.gov) SEER*Stat Database: Incidence - SEER 18 Regs Research Data, Nov 2015 Sub (1973-2013) - Linked To County Attributes - Total U.S., 1969-2014 Counties, National Cancer Institute, DCCPS, Surveillance Research Program, Surveillance Systems Branch, released April 2016, based on the November 2015 submission.
Surveillance, Epidemiology, and End Results (SEER) Program (www.seer.cancer.gov) SEER*Stat Database: Incidence - SEER 18 Regs Research Data, Nov 2018 Sub (1975-2016) <Katrina/Rita Population Adjustment> - Linked To County Attributes - Total U.S., 1969-2017 Counties, National Cancer Institute, DCCPS, Surveillance Research Program, released April 2019, based on the November 2018 submission.

© 2025 GBD 2021 US CNS Cancer Collaborators. *JAMA Neurology*.

eAppendix IV: Supplementary Tables

eTable 2a: Incidence count and age-standardized rate of Brain and central nervous system cancer by year and sex and percentage change from 1990 to 2021, United States

	Both		Male		Female	
	Absolute number	Age-standardized rate, per 100 000 people	Absolute number	Age-standardized rate, per 100 000 people	Absolute number	Age-standardized rate, per 100 000 people
**1990**	19755.84 (19189.37 to 20173.19)	7.01 (6.83 to 7.14)	10604 (10347.42 to 10796.58)	8.13 (7.93 to 8.28)	9151.84 (8773.75 to 9420.04)	6.07 (5.89 to 6.23)
**1991**	20307.11 (19732.75 to 20722.9)	7.11 (6.93 to 7.25)	10822.02 (10584.17 to 11022.98)	8.18 (8 to 8.33)	9485.09 (9106.08 to 9752.65)	6.22 (6.02 to 6.38)
**1992**	20536.71 (19964.22 to 20971.8)	7.09 (6.91 to 7.23)	10954.9 (10717.92 to 11167.3)	8.15 (7.97 to 8.31)	9581.81 (9177.59 to 9873.84)	6.19 (6 to 6.36)
**1993**	20933.45 (20329.46 to 21329.61)	7.13 (6.95 to 7.25)	11235.01 (11005.7 to 11440.37)	8.24 (8.07 to 8.39)	9698.44 (9292.95 to 9979.62)	6.18 (5.99 to 6.34)
**1994**	21422.49 (20829.69 to 21849.39)	7.19 (7.02 to 7.32)	11541.34 (11312.68 to 11747.61)	8.35 (8.18 to 8.49)	9881.15 (9452.99 to 10160.89)	6.2 (5.99 to 6.35)
**1995**	21599.79 (20965.32 to 22024.41)	7.15 (6.98 to 7.28)	11636.31 (11396.09 to 11845.69)	8.3 (8.13 to 8.45)	9963.48 (9538.21 to 10260.71)	6.17 (5.96 to 6.32)
**1996**	22013.56 (21371.76 to 22432.73)	7.21 (7.03 to 7.34)	11826.59 (11577.18 to 12036.08)	8.33 (8.14 to 8.48)	10186.97 (9743.85 to 10471.71)	6.25 (6.05 to 6.4)
**1997**	22263.39 (21574.81 to 22737.14)	7.18 (6.99 to 7.31)	11903.93 (11626.67 to 12134.45)	8.24 (8.05 to 8.41)	10359.45 (9891.21 to 10662.8)	6.27 (6.05 to 6.42)
**1998**	22751.56 (22026.98 to 23234.45)	7.22 (7.01 to 7.35)	12213.94 (11938.01 to 12450.75)	8.33 (8.12 to 8.49)	10537.62 (10036.21 to 10851.73)	6.26 (6.05 to 6.42)
**1999**	23846.4 (22991.79 to 24353.85)	7.42 (7.2 to 7.56)	12705.17 (12401.24 to 12957.51)	8.53 (8.33 to 8.71)	11141.23 (10576.91 to 11475.87)	6.46 (6.21 to 6.62)
**2000**	24257.72 (23298.09 to 24819.8)	7.43 (7.18 to 7.59)	12995.46 (12678.24 to 13263.8)	8.6 (8.38 to 8.78)	11262.27 (10642.6 to 11609.22)	6.42 (6.17 to 6.59)
**2001**	24546.33 (23589.39 to 25077.25)	7.42 (7.17 to 7.57)	13122.82 (12775.51 to 13395.03)	8.55 (8.32 to 8.73)	11423.51 (10785.57 to 11776.87)	6.45 (6.18 to 6.62)
**2002**	24799.98 (23795.77 to 25336)	7.41 (7.16 to 7.56)	13287.77 (12918.55 to 13559.21)	8.53 (8.3 to 8.71)	11512.22 (10879.1 to 11857.48)	6.43 (6.18 to 6.59)
**2003**	24910.73 (23868.47 to 25442.22)	7.35 (7.1 to 7.49)	13321.68 (12935.69 to 13603.16)	8.41 (8.18 to 8.59)	11589.05 (10936.61 to 11944.92)	6.43 (6.16 to 6.59)
**2004**	24656.61 (23573.81 to 25247.44)	7.16 (6.9 to 7.31)	13210.55 (12838.15 to 13501.29)	8.21 (7.97 to 8.38)	11446.06 (10760.57 to 11814.45)	6.23 (5.96 to 6.38)
**2005**	24946.18 (23827.46 to 25526.49)	7.12 (6.87 to 7.26)	13422.41 (13016.36 to 13707.13)	8.19 (7.94 to 8.37)	11523.77 (10864.06 to 11890.51)	6.16 (5.9 to 6.33)
**2006**	24875.73 (23769.57 to 25459.26)	6.99 (6.76 to 7.13)	13390.29 (12978.21 to 13692.56)	8.03 (7.78 to 8.21)	11485.44 (10804.92 to 11848.36)	6.07 (5.82 to 6.24)
**2007**	25209.16 (24132.45 to 25849.98)	6.98 (6.74 to 7.14)	13566.27 (13123 to 13874.24)	7.99 (7.74 to 8.17)	11642.9 (10942.19 to 12033.86)	6.08 (5.82 to 6.25)
**2008**	25914.71 (24735.26 to 26546.91)	7.05 (6.81 to 7.2)	14012.02 (13577.85 to 14312.26)	8.11 (7.86 to 8.28)	11902.69 (11142.91 to 12313.15)	6.12 (5.83 to 6.29)
**2009**	26675.25 (25453.88 to 27346.38)	7.13 (6.88 to 7.29)	14381.55 (13913.66 to 14683.01)	8.17 (7.93 to 8.35)	12293.7 (11541.67 to 12721.25)	6.21 (5.92 to 6.38)

© 2025 GBD 2021 US CNS Cancer Collaborators. *JAMA Neurology*.

**2010**	26896.66<br>(25654.33 to 27620.17)	7.06<br>(6.81 to 7.22)	14595.15<br>(14076.01 to 14925.26)	8.13<br>(7.87 to 8.32)	12301.52<br>(11525.91 to 12757.93)	6.1<br>(5.8 to 6.28)
**2011**	27536.58<br>(26270.36 to 28274.1)	7.12<br>(6.86 to 7.28)	14942.47<br>(14428.86 to 15287.64)	8.19<br>(7.92 to 8.37)	12594.11<br>(11800.81 to 13041.43)	6.16<br>(5.87 to 6.34)
**2012**	28306.46<br>(27022.67 to 29047.57)	7.2<br>(6.94 to 7.36)	15380.97<br>(14863.38 to 15734.86)	8.28<br>(8.02 to 8.46)	12925.49<br>(12098.38 to 13405.8)	6.23<br>(5.94 to 6.42)
**2013**	28584.93<br>(27297.01 to 29361.99)	7.14<br>(6.88 to 7.3)	15428.08<br>(14856.32 to 15796.09)	8.15<br>(7.87 to 8.33)	13156.85<br>(12289.76 to 13647.88)	6.24<br>(5.95 to 6.43)
**2014**	29250.1<br>(27935.04 to 30042.44)	7.16<br>(6.89 to 7.33)	15888.42<br>(15323.87 to 16260.45)	8.22<br>(7.94 to 8.42)	13361.69<br>(12454.06 to 13869.29)	6.21<br>(5.93 to 6.4)
**2015**	29919.69<br>(28515.09 to 30744.18)	7.21<br>(6.95 to 7.37)	16251.93<br>(15666.82 to 16615.85)	8.26<br>(7.98 to 8.45)	13667.75<br>(12755.56 to 14198.5)	6.27<br>(5.99 to 6.47)
**2016**	30793.51<br>(29320.61 to 31625.38)	7.31<br>(7.06 to 7.47)	16779<br>(16161.05 to 17155.22)	8.38<br>(8.11 to 8.56)	14014.51<br>(13112.39 to 14538.75)	6.35<br>(6.07 to 6.55)
**2017**	30904.68<br>(29353.13 to 31738.05)	7.2<br>(6.93 to 7.37)	16807.14<br>(16186.37 to 17194.15)	8.22<br>(7.96 to 8.42)	14097.54<br>(13149.8 to 14630.44)	6.3<br>(6.02 to 6.49)
**2018**	31008.45<br>(29483.3 to 31868.2)	7.11<br>(6.86 to 7.27)	16983.88<br>(16377.46 to 17374.5)	8.17<br>(7.91 to 8.36)	14024.56<br>(13024.64 to 14569)	6.17<br>(5.87 to 6.36)
**2019**	31173.9<br>(29593.43 to 32042.78)	7.02<br>(6.76 to 7.18)	17133.52<br>(16484.82 to 17559.27)	8.08<br>(7.8 to 8.27)	14040.38<br>(13020.1 to 14601.4)	6.08<br>(5.79 to 6.27)
**2020**	31355.06<br>(29606.89 to 32290.84)	6.93<br>(6.64 to 7.1)	17249.45<br>(16482.31 to 17739.77)	7.99<br>(7.69 to 8.2)	14105.61<br>(13067.1 to 14686.25)	5.99<br>(5.69 to 6.2)
**2021**	31780<br>(29971.1 to 32843.9)	6.91<br>(6.58 to 7.12)	17459.65<br>(16653.75 to 18044.82)	7.96<br>(7.64 to 8.22)	14320.35<br>(13269.49 to 14929.73)	5.97<br>(5.66 to 6.21)
**Percentage Change, 1990-2021**	60.86%<br>(54.87% to 65.22%)	-1.45%<br>(-4.41% to 0.91%)	64.65%<br>(58.71% to 70.79%)	-2.02%<br>(-5.3% to 1.38%)	56.48%<br>(49.8% to 62.12%)	-1.75%<br>(-5.32% to 1.71%)
**Statsitical Analysis**	ρ = 0.9974<br>p-value < 0.0001	ρ = -0.2254<br>p-value = 0.2139	ρ = 0.9934<br>p-value < 0.0001	ρ = -0.3816<br>p-value = 0.0319	ρ = 0.9941<br>p-value < 0.0001	ρ = -0.2144<br>p-value = 0.2376

Wilcoxon rank sum test between between sex (count): p-value = 0.0003
Wilcoxon rank sum test between sex (rate): p-value < 0.0001

© 2025 GBD 2021 US CNS Cancer Collaborators. *JAMA Neurology*.

eTable 2b: DALY count and age-standardized rate of Brain and central nervous system cancer by year and sex, and percentage change from 1990 to 2021, United States

	Both		Male		Female	
	Absolute number	Age-standardized rate, per 100 000 people	Absolute number	Age-standardized rate, per 100 000 people	Absolute number	Age-standardized rate, per 100 000 people
**1990**	435838.76 (427480.15 to 442708.68)	159.54 (156.74 to 161.86)	247563.28 (242741.64 to 251793.25)	191.11 (187.38 to 194.32)	188275.48 (182458.12 to 191918.8)	131.3 (128.18 to 133.45)
**1991**	445734.55 (436383.78 to 452091.3)	161.07 (158.28 to 163.15)	251585.51 (247064.84 to 255755.93)	191.57 (188.15 to 194.76)	194149.04 (188201.66 to 197712.23)	133.72 (130.68 to 135.78)
**1992**	447329.35 (438303.03 to 454244.79)	159.23 (156.33 to 161.39)	252677.82 (247749.05 to 257022.14)	189.31 (185.79 to 192.42)	194651.53 (188416.28 to 198583.25)	132.22 (129.16 to 134.55)
**1993**	452064.16 (442887.25 to 458311.25)	158.66 (155.84 to 160.61)	257307.74 (253598.75 to 260864.1)	189.92 (187.13 to 192.64)	194756.42 (188921.52 to 198348.82)	130.39 (127.23 to 132.33)
**1994**	458104.84 (448972.94 to 464297.45)	158.41 (155.6 to 160.32)	262115.17 (258198.5 to 265798.17)	190.6 (187.7 to 193.3)	195989.67 (190304.37 to 199632.4)	129.04 (125.89 to 131.01)
**1995**	456494.53 (446950.01 to 462518.69)	155.52 (152.79 to 157.4)	260883.39 (257172.11 to 264563.84)	186.8 (184.19 to 189.47)	195611.14 (189509 to 199310.26)	126.93 (123.77 to 128.9)
**1996**	459164.61 (449601.59 to 465546.11)	154.61 (151.92 to 156.53)	261574.06 (257704.53 to 265174.62)	184.77 (182.11 to 187.39)	197590.55 (191586.55 to 201243.79)	127.07 (124.04 to 129.04)
**1997**	458786.98 (448903.18 to 465819.88)	151.98 (149.24 to 154.15)	260342.65 (255810.14 to 264797.89)	180.66 (177.56 to 183.8)	198444.33 (192365.12 to 202262.56)	125.81 (122.76 to 127.81)
**1998**	464113.32 (453078.63 to 471620.23)	151.16 (148.1 to 153.44)	264545.13 (259661.75 to 269001.37)	180.53 (177.25 to 183.44)	199568.18 (192322.55 to 203737.38)	124.34 (121.05 to 126.56)
**1999**	478080.33 (466812.17 to 485900.23)	152.95 (149.87 to 155.27)	271467.53 (266553 to 276430.66)	182.16 (178.86 to 185.52)	206612.81 (199262.13 to 210955.34)	126.36 (123.17 to 128.54)
**2000**	482009.03 (470029.71 to 490096.42)	151.87 (148.77 to 154.2)	275821.57 (270750 to 280520.14)	182.14 (178.79 to 185.39)	206187.45 (198455.09 to 210636.25)	124.15 (120.63 to 126.31)
**2001**	484575.74 (472471.61 to 492248.47)	150.47 (147.4 to 152.66)	276817.84 (271669.33 to 281156.53)	179.71 (176.38 to 182.68)	207757.9 (200149.55 to 212064.65)	123.72 (120.33 to 125.85)
**2002**	489297.46 (476697.87 to 496938.47)	149.9 (146.72 to 152.06)	279774.06 (274925.4 to 284114.19)	178.98 (175.89 to 181.83)	209523.4 (201662.65 to 213769.39)	123.15 (119.57 to 125.27)
**2003**	491107.11 (477993.38 to 498745)	148.38 (145.04 to 150.51)	279909.51 (274695.93 to 284496.68)	176.01 (172.85 to 178.9)	211197.59 (203043.85 to 215497.21)	123.03 (119.53 to 125.12)
**2004**	484017.87 (471028.01 to 492281.15)	143.82 (140.71 to 146.06)	277070.79 (271145.27 to 281869.06)	171.34 (167.67 to 174.34)	206947.08 (198706.61 to 211411.76)	118.32 (114.9 to 120.44)
**2005**	489993.85 (476147.18 to 498213.1)	143.04 (139.81 to 145.18)	281093.12 (275219.13 to 285797.86)	170.73 (167.23 to 173.49)	208900.72 (201032.63 to 213217.13)	117.37 (114.07 to 119.36)
**2006**	489152.61 (475223.08 to 497642.32)	140.51 (137.22 to 142.71)	280310.58 (274243.42 to 285304.3)	167.26 (163.76 to 170.14)	208842.03 (200691.52 to 213260.71)	115.8 (112.46 to 117.8)
**2007**	494243.09 (479554.97 to 503150.94)	139.76 (136.26 to 142.08)	282998.95 (276804.01 to 288205.48)	165.9 (162.45 to 168.91)	211244.14 (202446.42 to 216132.73)	115.57 (112.07 to 117.8)
**2008**	505568.62 (490245.44 to 514383.32)	140.5 (136.97 to 142.69)	290691.3 (283869.74 to 295713.22)	167.46 (163.79 to 170.33)	214877.31 (205604.72 to 219722.06)	115.52 (111.78 to 117.72)
**2009**	515115.38 (498744.32 to 524447.77)	140.63 (137.01 to 142.87)	295644.87 (289005.93 to 300815.37)	167.4 (163.78 to 170.35)	219470.51 (210205.5 to 224361.34)	115.73 (112.26 to 117.99)
**2010**	516408.23 (499831.62 to 526011.12)	138.27 (134.76 to 140.55)	297998.63 (290528.49 to 303572.42)	165.45 (161.73 to 168.49)	218409.6 (208433.89 to 223872.95)	112.98 (109.1 to 115.24)

© 2025 GBD 2021 US CNS Cancer Collaborators. *JAMA Neurology*.

**2011**	528800.52 (512152.34 to 538693.68)	139.41 (135.97 to 141.73)	304754.62 (297563.84 to 309944.89)	166.55 (162.96 to 169.3)	224045.91 (214225.41 to 229449.96)	114.09 (110.45 to 116.24)
**2012**	543279.98 (525107.11 to 552706.05)	141.07 (137.38 to 143.18)	312918.48 (305257.21 to 318327.13)	168.33 (164.49 to 171.38)	230361.5 (219517.27 to 236158.32)	115.65 (111.82 to 117.99)
**2013**	549255.83 (531254.04 to 559776.44)	140.3 (136.57 to 142.7)	313758.04 (305992.93 to 319357.23)	166.05 (162.19 to 169.02)	235497.79 (224733.04 to 241241)	116.3 (112.59 to 118.67)
**2014**	562510.96 (543140.92 to 573148.93)	141.11 (137.43 to 143.56)	322946.63 (313858.49 to 328718.19)	167.91 (163.68 to 170.95)	239564.33 (228855.63 to 245682.13)	116.08 (112.54 to 118.53)
**2015**	572941.82 (553203.89 to 583684.05)	141.6 (137.86 to 143.88)	329225.19 (320191.89 to 334843.87)	168.34 (164.18 to 171.09)	243716.63 (232628.45 to 249961.28)	116.61 (112.94 to 118.93)
**2016**	588212.41 (566799.24 to 599184.65)	143.29 (139.59 to 145.55)	338433.1 (329717.59 to 344165.12)	170.35 (166.44 to 173.22)	249779.31 (238409.47 to 256101.42)	118 (114.37 to 120.38)
**2017**	588992.5 (567635.15 to 599987.56)	141.29 (137.63 to 143.51)	337819.34 (328924.11 to 343804.05)	167.21 (163.31 to 170.05)	251173.17 (239548.41 to 257606.89)	117.16 (113.59 to 119.5)
**2018**	589125.51 (569277.14 to 600824.69)	139.44 (135.96 to 141.83)	340066.31 (330830.93 to 346252.68)	166.07 (161.81 to 168.95)	249059.2 (236842.17 to 255813.17)	114.6 (110.99 to 117)
**2019**	589228.38 (569118.29 to 601942.38)	137.29 (133.74 to 139.76)	341154.25 (331188.33 to 348178.18)	163.83 (159.37 to 167.04)	248074.13 (236177.29 to 254898.32)	112.59 (109.02 to 115.07)
**2020**	590320.86 (566859.86 to 604381.86)	135.35 (131.37 to 138.17)	342032.02 (330505.49 to 350207.63)	161.87 (156.99 to 165.48)	248288.84 (235823.09 to 256185.67)	110.65 (106.89 to 113.58)
**2021**	594996.15 (571294.69 to 610278.4)	134.38 (129.83 to 137.95)	344129.03 (330881.91 to 353859.18)	160.7 (155.05 to 165.07)	250867.11 (237587.77 to 259574.57)	109.9 (105.59 to 113.56)
**Percentage Change, 1990-2021**	36.52% (32.71% to 39.72%)	-15.77% (-17.75% to -13.68%)	39.01% (34.61% to 43.09%)	-15.91% (-18.61% to -13.18%)	33.24% (29.12% to 36.5%)	-16.3% (-18.59% to -14.3%)
**Statsitical Analysis**	ρ = 0.9934 p-value < 0.0001	ρ = -0.9007 p-value < 0.0001	ρ = 0.9949 p-value < 0.0001	ρ = -0.9117 p-value < 0.0001	ρ = 0.9839 p-value < 0.0001	ρ = -0.8985 p-value < 0.0001

Wilcoxon rank sum test between between sex (count): p-value < 0.0001
Wilcoxon rank sum test between sex (rate): p-value < 0.0001

© 2025 GBD 2021 US CNS Cancer Collaborators. *JAMA Neurology*.

eTable 2c: Death count and age-standardized rate of Brain and central nervous system cancer by year and sex, and percentage change from 1990 to 2021, United States

	Both		Male		Female	
	Absolute number	Age-standardized rate, per 100 000 people	Absolute number	Age-standardized rate, per 100 000 people	Absolute number	Age-standardized rate, per 100 000 people
**1990**	13246.78 (12787.55 to 13529.49)	4.48 (4.34 to 4.56)	7264.24 (7090.33 to 7392.02)	5.47 (5.34 to 5.57)	5982.54 (5664.34 to 6160.29)	3.65 (3.5 to 3.74)
**1991**	13560.57 (13084.11 to 13840.02)	4.52 (4.39 to 4.6)	7384.17 (7208.83 to 7515.22)	5.49 (5.35 to 5.59)	6176.4 (5824.72 to 6363.58)	3.72 (3.56 to 3.81)
**1992**	13655.64 (13154.65 to 13944.38)	4.48 (4.34 to 4.57)	7442.44 (7261.59 to 7579.57)	5.44 (5.3 to 5.54)	6213.2 (5859.7 to 6407.75)	3.69 (3.53 to 3.79)
**1993**	13837.22 (13292.81 to 14114.77)	4.47 (4.32 to 4.55)	7578.62 (7400.74 to 7705.18)	5.45 (5.32 to 5.54)	6258.59 (5894.4 to 6442.46)	3.66 (3.5 to 3.74)
**1994**	14078.13 (13531.51 to 14375.16)	4.49 (4.34 to 4.57)	7717.29 (7544.24 to 7842.61)	5.46 (5.34 to 5.55)	6360.84 (5979.2 to 6550.73)	3.66 (3.5 to 3.75)
**1995**	14096.4 (13541.43 to 14389)	4.43 (4.28 to 4.5)	7717.69 (7542.97 to 7837.98)	5.38 (5.26 to 5.46)	6378.72 (6003.75 to 6577.7)	3.62 (3.46 to 3.71)
**1996**	14218.85 (13658.97 to 14519.24)	4.41 (4.26 to 4.49)	7771.82 (7587.14 to 7901.29)	5.34 (5.21 to 5.42)	6447.02 (6060.34 to 6642.65)	3.62 (3.46 to 3.71)
**1997**	14330.87 (13733.07 to 14646.09)	4.36 (4.21 to 4.45)	7795.9 (7600.79 to 7941.51)	5.26 (5.12 to 5.35)	6534.97 (6130.02 to 6742.78)	3.61 (3.44 to 3.7)
**1998**	14627.47 (13999.99 to 14964.73)	4.37 (4.21 to 4.46)	7992.82 (7787.64 to 8144.29)	5.3 (5.16 to 5.4)	6634.66 (6204.86 to 6852.9)	3.59 (3.41 to 3.68)
**1999**	15392.6 (14711.85 to 15757.27)	4.5 (4.34 to 4.6)	8346.83 (8127.72 to 8518.47)	5.44 (5.3 to 5.56)	7045.77 (6560.27 to 7283.77)	3.71 (3.52 to 3.81)
**2000**	15604.99 (14867.34 to 16000.77)	4.48 (4.31 to 4.58)	8494.16 (8257.19 to 8666.27)	5.45 (5.29 to 5.56)	7110.83 (6587.21 to 7361.73)	3.67 (3.46 to 3.77)
**2001**	15722.05 (14955.25 to 16117.39)	4.45 (4.27 to 4.55)	8567.55 (8315.33 to 8741.59)	5.39 (5.23 to 5.51)	7154.5 (6643.09 to 7403.12)	3.65 (3.45 to 3.74)
**2002**	15824.5 (15032.65 to 16214.09)	4.41 (4.23 to 4.51)	8639.71 (8376.63 to 8810.26)	5.34 (5.18 to 5.45)	7184.79 (6654.73 to 7431.73)	3.62 (3.42 to 3.72)
**2003**	15842.98 (15025.91 to 16225.92)	4.35 (4.16 to 4.44)	8658.33 (8380.52 to 8834.16)	5.25 (5.08 to 5.35)	7184.66 (6660.6 to 7435.02)	3.59 (3.39 to 3.68)
**2004**	15766.11 (14946.97 to 16189.76)	4.25 (4.06 to 4.35)	8605.34 (8321.02 to 8798.57)	5.11 (4.95 to 5.23)	7160.76 (6617.08 to 7428.99)	3.5 (3.29 to 3.6)
**2005**	16015.38 (15152.19 to 16446.05)	4.24 (4.04 to 4.33)	8781.03 (8493.22 to 8965.66)	5.11 (4.95 to 5.22)	7234.35 (6685.67 to 7506.08)	3.47 (3.28 to 3.57)
**2006**	16032.25 (15159.51 to 16468.37)	4.16 (3.97 to 4.26)	8824.58 (8519.04 to 9018.32)	5.03 (4.86 to 5.14)	7207.66 (6662.09 to 7474.43)	3.41 (3.22 to 3.51)
**2007**	16251.08 (15380.49 to 16717.94)	4.14 (3.95 to 4.25)	8945.26 (8624.05 to 9151.36)	4.99 (4.81 to 5.11)	7305.82 (6743.89 to 7592.64)	3.41 (3.21 to 3.51)
**2008**	16689.33 (15764.98 to 17142.93)	4.17 (3.97 to 4.27)	9231.35 (8886.02 to 9429.26)	5.04 (4.85 to 5.15)	7457.98 (6879.3 to 7746.3)	3.41 (3.21 to 3.51)
**2009**	17151.79 (16165.99 to 17634.36)	4.19 (3.99 to 4.29)	9458.29 (9097.9 to 9668.95)	5.05 (4.86 to 5.16)	7693.5 (7083.73 to 7993.06)	3.45 (3.23 to 3.55)
**2010**	17308.52 (16338.64 to 17825.4)	4.14 (3.94 to 4.25)	9616.53 (9251.47 to 9834.65)	5.01 (4.83 to 5.13)	7691.99 (7075.41 to 8015.48)	3.38 (3.16 to 3.49)

© 2025 GBD 2021 US CNS Cancer Collaborators. *JAMA Neurology*.

**2011**	17732.39<br>(16744.1 to 18248.36)	4.16<br>(3.96 to 4.26)	9856.48<br>(9471.86 to 10082.05)	5.03<br>(4.84 to 5.14)	7875.92<br>(7268.39 to 8198.93)	3.4<br>(3.19 to 3.51)
**2012**	18301.45<br>(17270.39 to 18838.15)	4.21<br>(4 to 4.31)	10200.9<br>(9808.39 to 10433.52)	5.09<br>(4.9 to 5.2)	8100.55<br>(7462.09 to 8438)	3.43<br>(3.22 to 3.54)
**2013**	18596.44<br>(17534.12 to 19157.42)	4.18<br>(3.98 to 4.29)	10301.26<br>(9881.35 to 10537.36)	5.02<br>(4.82 to 5.13)	8295.18<br>(7629.52 to 8649.23)	3.45<br>(3.23 to 3.57)
**2014**	19166.98<br>(18064.7 to 19732.76)	4.22<br>(4.02 to 4.33)	10677.13<br>(10233.22 to 10916.8)	5.09<br>(4.89 to 5.2)	8489.85<br>(7821.89 to 8850.66)	3.46<br>(3.25 to 3.58)
**2015**	19643.88<br>(18516.19 to 20228.93)	4.24<br>(4.03 to 4.35)	10958.68<br>(10496.6 to 11216.81)	5.11<br>(4.9 to 5.22)	8685.2<br>(7995.7 to 9060.61)	3.47<br>(3.26 to 3.59)
**2016**	20239.39<br>(19088.05 to 20831.36)	4.28<br>(4.08 to 4.39)	11342.39<br>(10858.24 to 11599.16)	5.17<br>(4.97 to 5.29)	8897<br>(8195.28 to 9274.74)	3.5<br>(3.29 to 3.62)
**2017**	20419.31<br>(19251.05 to 21008.77)	4.23<br>(4.03 to 4.34)	11436.96<br>(10944.75 to 11699.7)	5.1<br>(4.89 to 5.21)	8982.34<br>(8268.74 to 9357.33)	3.47<br>(3.26 to 3.58)
**2018**	20582.7<br>(19377.8 to 21200.24)	4.18<br>(3.98 to 4.29)	11604.61<br>(11139.95 to 11893.36)	5.07<br>(4.87 to 5.19)	8978.09<br>(8226.12 to 9359.72)	3.4<br>(3.18 to 3.52)
**2019**	20810.23<br>(19563.36 to 21442.24)	4.14<br>(3.94 to 4.25)	11791.29<br>(11279.37 to 12093.49)	5.03<br>(4.82 to 5.16)	9018.93<br>(8274.92 to 9417.34)	3.35<br>(3.13 to 3.46)
**2020**	21031.62<br>(19690.83 to 21714.74)	4.1<br>(3.88 to 4.21)	11920.87<br>(11298.36 to 12251.97)	4.99<br>(4.74 to 5.12)	9110.75<br>(8310.06 to 9522.92)	3.31<br>(3.09 to 3.43)
**2021**	21444.08<br>(20045.85 to 22166.97)	4.1<br>(3.87 to 4.22)	12132.27<br>(11497.15 to 12524.76)	4.99<br>(4.76 to 5.14)	9311.81<br>(8488.03 to 9745.27)	3.32<br>(3.09 to 3.45)
**Percentage Change, 1990-2021**	61.88%<br>(56.18% to 66.11%)	-8.41%<br>(-11.09% to -6.22%)	67.01%<br>(61.28% to 72.54%)	-8.84%<br>(-11.72% to -5.95%)	55.65%<br>(49.65% to 59.98%)	-9.18%<br>(-12.11% to -6.85%)
**Statsitical Analysis**	ρ = 0.9995<br>p-value < 0.0001	ρ = -0.5326<br>p-value = 0.0003	ρ = 0.9994<br>p-value < 0.0001	ρ = -0.6538<br>p-value < 0.0001	ρ = 0.9989<br>p-value < 0.0001	ρ = -0.4996<br>p-value = 0.0009

Wilcoxon rank sum test between between sex (count): p-value < 0.0001
Wilcoxon rank sum test between sex (rate): p-value < 0.0001

© 2025 GBD 2021 US CNS Cancer Collaborators. *JAMA Neurology*.

eTable 2d: Prevalence count and age-standardized rate of Brain and central nervous system cancer by year and sex, and percentage change from 1990 to 2021, United States

	Both		Male		Female	
	Absolute number	Age-standardized rate, per 100 000 people	Absolute number	Age-standardized rate, per 100 000 people	Absolute number	Age-standardized rate, per 100 000 people
**1990**	55754.27 (54576.36 to 56910.79)	21.59 (21.12 to 22.04)	29455.36 (28712.28 to 30110.39)	23.4 (22.83 to 23.96)	26298.91 (25440.66 to 27118.74)	19.98 (19.37 to 20.65)
**1991**	57629.52 (56386.85 to 58772.49)	22.03 (21.58 to 22.5)	30196.31 (29483.4 to 30894.01)	23.69 (23.1 to 24.26)	27433.21 (26573.17 to 28290.14)	20.58 (19.98 to 21.22)
**1992**	58634.89 (57352.35 to 59804.69)	22.14 (21.65 to 22.59)	30753.29 (30032.35 to 31493.64)	23.79 (23.23 to 24.38)	27881.6 (27041.45 to 28728)	20.67 (20.09 to 21.32)
**1993**	60271.17 (58983.67 to 61461.97)	22.53 (22.05 to 22.97)	31890.83 (31145.98 to 32602.14)	24.42 (23.83 to 25)	28380.34 (27580.23 to 29219)	20.83 (20.26 to 21.42)
**1994**	61892.03 (60664.49 to 63041.12)	22.86 (22.39 to 23.3)	33063.83 (32321.05 to 33757.56)	25.02 (24.4 to 25.59)	28828.19 (28000.28 to 29625.21)	20.85 (20.27 to 21.43)
**1995**	62488.06 (61204.89 to 63634.15)	22.83 (22.37 to 23.26)	33506.14 (32765.18 to 34222.46)	25.1 (24.49 to 25.67)	28981.92 (28136.84 to 29780.58)	20.71 (20.14 to 21.29)
**1996**	64161.64 (62913.85 to 65320.97)	23.28 (22.82 to 23.73)	34268.03 (33517.27 to 35026.58)	25.46 (24.85 to 26.08)	29893.61 (28995.71 to 30702.74)	21.24 (20.62 to 21.81)
**1997**	64920.54 (63636.8 to 66202.15)	23.27 (22.79 to 23.77)	34565.13 (33730.03 to 35401.76)	25.33 (24.69 to 25.98)	30355.41 (29443.77 to 31172.44)	21.36 (20.73 to 21.97)
**1998**	66418.81 (65029.43 to 67691.73)	23.5 (23.01 to 23.95)	35508.18 (34669.12 to 36352.64)	25.69 (25.06 to 26.34)	30910.63 (29953.28 to 31771.89)	21.46 (20.84 to 22.09)
**1999**	69326.69 (67869.02 to 70663.94)	24.19 (23.7 to 24.65)	36778.74 (35921.32 to 37696.03)	26.27 (25.62 to 26.96)	32547.94 (31510.89 to 33474.8)	22.28 (21.65 to 22.92)
**2000**	71627.01 (69964.01 to 73039.11)	24.73 (24.2 to 25.22)	38337.48 (37438.09 to 39296.15)	27.09 (26.44 to 27.81)	33289.53 (32173.82 to 34251.94)	22.53 (21.84 to 23.2)
**2001**	73598.54 (71983.71 to 74988.74)	25.11 (24.58 to 25.59)	39109.87 (38219.96 to 40112.76)	27.27 (26.59 to 27.99)	34488.67 (33405 to 35414.41)	23.11 (22.42 to 23.72)
**2002**	75207.1 (73559.63 to 76644.65)	25.44 (24.91 to 25.94)	40128.81 (39212.97 to 41044.72)	27.76 (27.08 to 28.44)	35078.29 (33967.02 to 36096.21)	23.26 (22.56 to 23.92)
**2003**	76118.21 (74413.93 to 77495.52)	25.54 (25 to 26)	40315.08 (39329.33 to 41281.84)	27.58 (26.87 to 28.27)	35803.13 (34615.52 to 36815.86)	23.65 (22.94 to 24.33)
**2004**	74605.9 (72755.03 to 76006.97)	24.75 (24.23 to 25.23)	39825.28 (38841.97 to 40768.45)	26.95 (26.24 to 27.63)	34780.62 (33614.32 to 35761.39)	22.67 (22.02 to 23.32)
**2005**	74764.75 (73091.93 to 76091.74)	24.5 (23.97 to 24.92)	40104.42 (39101.87 to 41065.92)	26.82 (26.11 to 27.49)	34660.33 (33482.32 to 35584.65)	22.3 (21.66 to 22.9)
**2006**	73952.92 (72144.83 to 75434.15)	23.99 (23.46 to 24.46)	39438.17 (38438.6 to 40421.66)	26.07 (25.38 to 26.76)	34514.75 (33396.37 to 35516.05)	22.04 (21.44 to 22.65)
**2007**	74725.12 (72938.9 to 76210.2)	24.02 (23.49 to 24.5)	39813.74 (38814.16 to 40788.96)	26.02 (25.32 to 26.7)	34911.38 (33783.02 to 35994.86)	22.16 (21.54 to 22.82)
**2008**	76677.02 (74802.83 to 78259.53)	24.38 (23.84 to 24.9)	41132.35 (40088.57 to 42121.37)	26.59 (25.9 to 27.29)	35544.67 (34364.17 to 36591.99)	22.3 (21.65 to 22.98)
**2009**	78754.35 (76865.26 to 80398.85)	24.73 (24.2 to 25.26)	42196.99 (41076.8 to 43247.89)	26.96 (26.2 to 27.69)	36557.36 (35315.8 to 37652.23)	22.64 (21.94 to 23.28)

© 2025 GBD 2021 US CNS Cancer Collaborators. *JAMA Neurology*.

**2010**	78909.75 (76999.02 to 80705.27)	24.49 (23.95 to 25.04)	42550.95 (41522.76 to 43608.93)	26.87 (26.15 to 27.61)	36358.8 (34987.18 to 37523.35)	22.25 (21.55 to 22.95)
**2011**	80810.42 (78794.95 to 82683.35)	24.87 (24.31 to 25.42)	43617.13 (42621.51 to 44703.38)	27.31 (26.6 to 28.03)	37193.29 (35797.05 to 38362.45)	22.55 (21.85 to 23.3)
**2012**	82828.16 (80587.35 to 84670.51)	25.27 (24.69 to 25.83)	44688.93 (43570.38 to 45746.38)	27.72 (26.97 to 28.44)	38139.23 (36655.55 to 39337.67)	22.95 (22.24 to 23.69)
**2013**	82929.66 (80678.23 to 84769.17)	25 (24.42 to 25.53)	44432.52 (43263.66 to 45613.94)	27.24 (26.44 to 27.98)	38497.15 (36926.27 to 39759.72)	22.9 (22.15 to 23.65)
**2014**	83565.06 (81068.23 to 85534.92)	24.85 (24.25 to 25.41)	45164.16 (43867.4 to 46421.22)	27.31 (26.55 to 28.15)	38400.89 (36805.99 to 39683.55)	22.51 (21.74 to 23.28)
**2015**	84509.95 (81976.53 to 86344.83)	24.92 (24.3 to 25.47)	45532.82 (44284.93 to 46686.72)	27.23 (26.51 to 28)	38977.12 (37448.12 to 40300.78)	22.74 (21.95 to 23.5)
**2016**	86337.13 (83984.15 to 88075.06)	25.25 (24.64 to 25.77)	46540.94 (45274.46 to 47702.61)	27.57 (26.8 to 28.36)	39796.19 (38250.71 to 41112.45)	23.05 (22.24 to 23.84)
**2017**	85750.74 (83273.6 to 87508.78)	24.8 (24.21 to 25.32)	45964.38 (44666.08 to 47197.69)	26.84 (26.11 to 27.57)	39786.37 (38235.3 to 41087.62)	22.9 (22.07 to 23.64)
**2018**	85450.59 (82734.28 to 87395.68)	24.48 (23.91 to 25.02)	46160.57 (44893.93 to 47298.37)	26.69 (25.96 to 27.39)	39290.02 (37724.48 to 40568.72)	22.42 (21.56 to 23.17)
**2019**	84924.24 (82264.76 to 86868.45)	24.1 (23.5 to 24.66)	45847.06 (44429.43 to 47058.8)	26.2 (25.39 to 26.93)	39077.18 (37448.8 to 40447.22)	22.15 (21.33 to 22.88)
**2020**	84364.71 (81474.34 to 86701.62)	23.64 (22.96 to 24.29)	45644.77 (44091.77 to 47048.92)	25.78 (24.88 to 26.62)	38719.94 (37035.72 to 40197.68)	21.64 (20.76 to 22.48)
**2021**	84402.47 (80958.69 to 87130.86)	23.38 (22.43 to 24.28)	45640.22 (43801.73 to 47232.28)	25.51 (24.32 to 26.53)	38762.25 (36889.46 to 40440.67)	21.39 (20.33 to 22.52)
**Percentage Change, 1990-2021**	51.38% (45.74% to 56.31%)	8.28% (4.11% to 12.56%)	54.95% (48.69% to 61.56%)	9.02% (3.72% to 14.23%)	47.39% (40.15% to 54.09%)	7.05% (1.63% to 12.52%)
**Statsitical Analysis**	ρ = 0.9729 p-value < 0.0001	ρ = 0.5293 p-value = 0.0021	ρ = 0.9754 p-value < 0.0001	ρ = 0.5209 p-value = 0.0026	ρ = 0.9707 p-value < 0.0001	ρ = 0.5110 p-value = 0.0032

Wilcoxon rank sum test between between sex (count): p-value < 0.0001
Wilcoxon rank sum test between sex (rate): p-value < 0.0001

© 2025 GBD 2021 US CNS Cancer Collaborators. *JAMA Neurology*.

eTable 2e: YLD count and age-standardized rate of Brain and central nervous system cancer by year and sex, and percentage change from 1990 to 2021, United States

	Both		Male		Female	
	Absolute number	Age-standardized rate, per 100 000 people	Absolute number	Age-standardized rate, per 100 000 people	Absolute number	Age-standardized rate, per 100 000 people
**1990**	6974.17 (5073.55 to 9059.88)	2.55 (1.86 to 3.32)	3731.32 (2718.77 to 4834.48)	2.89 (2.1 to 3.75)	3242.85 (2350 to 4225.4)	2.25 (1.63 to 2.94)
**1991**	7176.2 (5197.41 to 9288.65)	2.59 (1.88 to 3.37)	3810.34 (2757.88 to 4912.36)	2.91 (2.12 to 3.76)	3365.86 (2441.61 to 4392.03)	2.31 (1.68 to 3.01)
**1992**	7286.92 (5274.85 to 9481.39)	2.59 (1.89 to 3.38)	3874.81 (2803.67 to 4967.36)	2.92 (2.12 to 3.76)	3412.11 (2466.7 to 4477.92)	2.31 (1.68 to 3.03)
**1993**	7458.2 (5382.42 to 9701.29)	2.62 (1.91 to 3.41)	3995.19 (2896.33 to 5143.65)	2.97 (2.15 to 3.83)	3463.01 (2518.19 to 4534.21)	2.31 (1.68 to 3.02)
**1994**	7636.83 (5538.55 to 9891.97)	2.65 (1.94 to 3.45)	4115.52 (2989.99 to 5311.73)	3.02 (2.21 to 3.91)	3521.31 (2541.12 to 4597.04)	2.32 (1.69 to 3.03)
**1995**	7711.06 (5569.94 to 9976.08)	2.64 (1.92 to 3.43)	4161.2 (3005 to 5354.21)	3.01 (2.19 to 3.88)	3549.85 (2547.66 to 4624.66)	2.31 (1.68 to 3.01)
**1996**	7873.12 (5725.58 to 10193.19)	2.67 (1.96 to 3.49)	4234.24 (3090.94 to 5428.38)	3.03 (2.22 to 3.91)	3638.88 (2641.6 to 4754.82)	2.35 (1.72 to 3.08)
**1997**	7960.39 (5813.1 to 10345.45)	2.67 (1.96 to 3.47)	4269.71 (3098.97 to 5522.07)	3.01 (2.2 to 3.91)	3690.68 (2691.18 to 4818.83)	2.35 (1.73 to 3.08)
**1998**	8132.77 (5956.65 to 10560.55)	2.68 (1.97 to 3.49)	4390.06 (3202.35 to 5690.37)	3.05 (2.24 to 3.95)	3742.71 (2734.19 to 4873.98)	2.35 (1.74 to 3.06)
**1999**	8466.47 (6215.65 to 10970.9)	2.75 (2.03 to 3.59)	4553.72 (3350 to 5911.46)	3.12 (2.29 to 4.05)	3912.74 (2863.05 to 5076.88)	2.42 (1.78 to 3.15)
**2000**	8686.39 (6349.39 to 11313.79)	2.78 (2.05 to 3.65)	4702.4 (3472.73 to 6149.55)	3.18 (2.35 to 4.16)	3983.98 (2895.41 to 5174.94)	2.43 (1.78 to 3.18)
**2001**	8853.28 (6502.47 to 11510.37)	2.8 (2.07 to 3.67)	4781.15 (3531.09 to 6222.31)	3.18 (2.35 to 4.15)	4072.13 (2959.25 to 5284.25)	2.46 (1.81 to 3.21)
**2002**	9003.4 (6548.7 to 11721.82)	2.81 (2.07 to 3.69)	4869.99 (3541.25 to 6341.52)	3.2 (2.34 to 4.17)	4133.41 (3014.39 to 5408.12)	2.47 (1.81 to 3.23)
**2003**	9087.73 (6661.04 to 11894.89)	2.81 (2.07 to 3.69)	4900.6 (3598.4 to 6394.63)	3.17 (2.34 to 4.16)	4187.13 (3058.76 to 5519.29)	2.48 (1.82 to 3.26)
**2004**	8969.74 (6571.12 to 11779.1)	2.73 (2.02 to 3.6)	4861.19 (3568.19 to 6429.51)	3.1 (2.3 to 4.1)	4108.55 (2994.13 to 5401.47)	2.39 (1.76 to 3.15)
**2005**	9048.35 (6681.33 to 11829.22)	2.71 (2 to 3.57)	4927.71 (3633.73 to 6433.17)	3.09 (2.29 to 4.05)	4120.64 (3028.8 to 5396.32)	2.36 (1.73 to 3.11)
**2006**	9005 (6620.09 to 11717.43)	2.66 (1.98 to 3.49)	4887.62 (3563.2 to 6362.75)	3.02 (2.22 to 3.95)	4117.37 (3039.62 to 5367.58)	2.34 (1.74 to 3.08)
**2007**	9110.85 (6713.42 to 11853.56)	2.66 (1.98 to 3.49)	4945.86 (3651.36 to 6459.98)	3 (2.24 to 3.94)	4164.99 (3057.65 to 5423.02)	2.34 (1.73 to 3.07)
**2008**	9363.42 (6936.57 to 12161.28)	2.69 (2 to 3.51)	5112.17 (3780.28 to 6627.96)	3.06 (2.27 to 3.98)	4251.25 (3152.45 to 5513.93)	2.35 (1.73 to 3.09)
**2009**	9617.54 (7075.75 to 12503.41)	2.72 (2.02 to 3.54)	5237.92 (3877.04 to 6806.73)	3.08 (2.3 to 4)	4379.62 (3232.62 to 5724.3)	2.38 (1.77 to 3.14)
**2010**	9696.87 (7175.58 to 12630.28)	2.69 (2.02 to 3.53)	5308.17 (3941.54 to 6928.63)	3.07 (2.29 to 4.01)	4388.71 (3209.05 to 5712.74)	2.35 (1.73 to 3.09)

© 2025 GBD 2021 US CNS Cancer Collaborators. *JAMA Neurology*.

**2011**	9934.28 (7360.85 to 12969.64)	2.72 (2.02 to 3.57)	5434.78 (4030.29 to 7080.58)	3.1 (2.31 to 4.04)	4499.5 (3313.43 to 5823.69)	2.38 (1.76 to 3.12)
**2012**	10190.66 (7552.27 to 13246.81)	2.76 (2.06 to 3.59)	5577.13 (4123.78 to 7250.87)	3.13 (2.32 to 4.08)	4613.53 (3401.56 to 5983.29)	2.41 (1.8 to 3.15)
**2013**	10253.22 (7507.57 to 13326.11)	2.73 (2.02 to 3.57)	5576.7 (4078.72 to 7210.01)	3.08 (2.28 to 4)	4676.52 (3421.25 to 6069.98)	2.4 (1.77 to 3.15)
**2014**	10431.88 (7647.23 to 13619.39)	2.73 (2.03 to 3.58)	5710.26 (4153 to 7495.93)	3.1 (2.3 to 4.07)	4721.62 (3473.04 to 6154.37)	2.38 (1.78 to 3.13)
**2015**	10611.96 (7809.33 to 13810)	2.74 (2.03 to 3.57)	5804.77 (4268.79 to 7486.27)	3.1 (2.3 to 4.04)	4807.19 (3512.53 to 6246.86)	2.4 (1.78 to 3.16)
**2016**	10879.85 (7961.01 to 14196.07)	2.77 (2.06 to 3.62)	5956.76 (4347.29 to 7732.36)	3.14 (2.32 to 4.11)	4923.1 (3652.23 to 6378.65)	2.43 (1.79 to 3.18)
**2017**	10886.8 (7947.62 to 14178.94)	2.73 (2.02 to 3.57)	5943.28 (4340.05 to 7714.69)	3.08 (2.28 to 4.01)	4943.51 (3616.72 to 6430.81)	2.41 (1.77 to 3.15)
**2018**	10894.46 (7906.1 to 14160.87)	2.7 (1.99 to 3.53)	5991.22 (4321.17 to 7793.24)	3.06 (2.23 to 3.99)	4903.24 (3596.44 to 6362.49)	2.36 (1.75 to 3.08)
**2019**	10902.62 (7972.41 to 14267.24)	2.66 (1.98 to 3.49)	5996.37 (4355.77 to 7853.7)	3.01 (2.23 to 3.95)	4906.26 (3605.74 to 6307.9)	2.33 (1.72 to 3.05)
**2020**	10915.34 (7962.8 to 14118.66)	2.62 (1.93 to 3.44)	6012.24 (4353.59 to 7787.63)	2.97 (2.17 to 3.89)	4903.1 (3573.81 to 6334.93)	2.29 (1.69 to 3.01)
**2021**	11009.32 (7989.38 to 14200)	2.6 (1.92 to 3.4)	6059.23 (4397.19 to 7872.68)	2.95 (2.17 to 3.89)	4950.09 (3597.8 to 6426.81)	2.27 (1.68 to 2.97)
**Percentage Change, 1990-2021**	57.86% (50.98% to 64.23%)	2.03% (-2.1% to 6.25%)	62.39% (52.9% to 72.5%)	2.25% (-3.3% to 8.51%)	52.65% (44.99% to 60.51%)	1.15% (-3.95% to 6.62%)
**Statsitical Analysis**	ρ = 0.9967 p-value < 0.0001	ρ = 0.2742 p-value = 0.1287	ρ = 0.9967 p-value < 0.0001	ρ = 0.2372 p-value = 0.1905	ρ = 0.9861 p-value < 0.0001	ρ = 0.2155 p-value = 0.2351

Wilcoxon rank sum test between between sex (count): p-value < 0.0001
Wilcoxon rank sum test between sex (rate): p-value < 0.0001

© 2025 GBD 2021 US CNS Cancer Collaborators. *JAMA Neurology*.

eTable 2f: YLL count and age-standardized rate of Brain and central nervous system cancer by year and sex, and percentage change from 1990 to 2021, United States

	Both		Male		Female	
	Absolute number	Age-standardized rate, per 100 000 people	Absolute number	Age-standardized rate, per 100 000 people	Absolute number	Age-standardized rate, per 100 000 people
**1990**	428864.59 (419828.74 to 435253.38)	156.99 (154.18 to 159.12)	243831.96 (239141.4 to 247589.72)	188.22 (184.7 to 191.06)	185032.63 (179784.82 to 188407.82)	129.06 (126.19 to 131.12)
**1991**	438558.35 (429855.75 to 444313.04)	158.48 (155.74 to 160.34)	247775.18 (243309.56 to 251501.93)	188.66 (185.28 to 191.45)	190783.17 (184965.75 to 194242.86)	131.41 (128.29 to 133.37)
**1992**	440042.42 (431567.26 to 446255.62)	156.64 (153.86 to 158.67)	248803 (244002.47 to 252822.03)	186.39 (182.86 to 189.33)	191239.42 (185301.35 to 195144.51)	129.92 (126.89 to 132.17)
**1993**	444605.96 (435682.57 to 450173.47)	156.04 (153.44 to 157.74)	253312.55 (249484.9 to 256706.04)	186.95 (184.24 to 189.43)	191293.41 (185435.26 to 194635.55)	128.08 (125.12 to 129.91)
**1994**	450468.01 (441653.25 to 456213.15)	155.76 (153.25 to 157.52)	257999.65 (254168.36 to 261398.48)	187.58 (184.87 to 190.02)	192468.36 (186856.43 to 196092.18)	126.73 (123.9 to 128.67)
**1995**	448783.48 (439796.86 to 454059.5)	152.87 (150.32 to 154.53)	256722.19 (253027.74 to 260113.03)	183.79 (181.24 to 186.23)	192061.29 (186315.06 to 195650.42)	124.63 (121.7 to 126.48)
**1996**	451291.49 (442426.18 to 457036.39)	151.94 (149.51 to 153.67)	257339.82 (253700.6 to 260748.13)	181.73 (179.19 to 184.13)	193951.67 (187970.88 to 197491.17)	124.72 (121.85 to 126.69)
**1997**	450826.59 (441345.86 to 457084.95)	149.32 (146.7 to 151.3)	256072.94 (251778.11 to 260008.73)	177.65 (174.74 to 180.42)	194753.65 (188594.43 to 198515.72)	123.45 (120.54 to 125.38)
**1998**	455980.55 (445730.38 to 462549.55)	148.48 (145.8 to 150.42)	260155.08 (255666.85 to 264302.38)	177.48 (174.44 to 180.23)	195825.47 (189008.27 to 199814.05)	121.99 (118.82 to 124.12)
**1999**	469613.87 (458912.8 to 476603)	150.2 (147.26 to 152.27)	266913.81 (262491.3 to 271123.56)	179.04 (176.07 to 181.88)	202700.06 (195456.19 to 206693.18)	123.94 (120.83 to 125.97)
**2000**	473322.64 (462015.8 to 480827.83)	149.09 (146.1 to 151.25)	271119.17 (266422.15 to 275220.8)	178.96 (175.79 to 181.78)	202203.47 (194407.61 to 206513.19)	121.72 (118.35 to 123.75)
**2001**	475722.46 (464331.14 to 482835.07)	147.67 (144.7 to 149.6)	272036.68 (267321.26 to 276181.18)	176.54 (173.4 to 179.25)	203685.77 (196169.78 to 207679)	121.26 (118.12 to 123.19)
**2002**	480294.06 (468023.36 to 487415.54)	147.08 (144.06 to 149.02)	274904.07 (269970.76 to 278902.23)	175.79 (172.84 to 178.3)	205389.99 (197660.14 to 209484.72)	120.68 (117.39 to 122.67)
**2003**	482019.38 (469302.31 to 489080.92)	145.57 (142.38 to 147.44)	275008.91 (270253.3 to 279312.46)	172.84 (169.86 to 175.45)	207010.46 (199136.39 to 211072.34)	120.54 (117.24 to 122.49)
**2004**	475048.13 (462189.14 to 483153.63)	141.09 (138.09 to 143.18)	272209.6 (266927.03 to 276906.83)	168.24 (165.19 to 171.07)	202838.53 (194594.36 to 207011.58)	115.93 (112.5 to 117.87)
**2005**	480945.5 (467626.27 to 488532.76)	140.33 (137.17 to 142.31)	276165.41 (270729.42 to 280734.97)	167.64 (164.54 to 170.28)	204780.09 (196867.81 to 208908.81)	115.01 (111.83 to 116.85)
**2006**	480147.61 (466680.14 to 488050.82)	137.85 (134.78 to 139.86)	275422.95 (269813.21 to 280005.1)	164.24 (161.03 to 166.97)	204724.66 (196777.06 to 208962.26)	113.47 (110.25 to 115.37)
**2007**	485132.24 (471200.54 to 493749.44)	137.1 (133.92 to 139.25)	278053.09 (271981.69 to 282886.94)	162.9 (159.47 to 165.62)	207079.15 (198719.79 to 211834.39)	113.24 (109.93 to 115.21)
**2008**	496205.2 (481850.1 to 504528.24)	137.82 (134.62 to 139.82)	285579.13 (279003.65 to 290348.18)	164.4 (160.9 to 167.05)	210626.06 (201867.02 to 215288.27)	113.16 (109.56 to 115.11)
**2009**	505497.84 (490284.97 to 514085.28)	137.91 (134.59 to 139.97)	290406.95 (283885.86 to 295236.53)	164.32 (160.98 to 167.05)	215090.88 (206147.77 to 219765.69)	113.35 (109.86 to 115.26)
**2010**	506711.36 (491110.16 to 515921.12)	135.58 (132.32 to 137.8)	292690.46 (285674.58 to 297824.03)	162.38 (159.03 to 165.25)	214020.9 (204613.39 to 219173.38)	110.63 (107.05 to 112.85)

© 2025 GBD 2021 US CNS Cancer Collaborators. *JAMA Neurology*.

**2011**	518866.24 (502748.76 to 527781.71)	136.69 (133.36 to 138.74)	299319.84 (292478.04 to 304238.87)	163.45 (160.02 to 166.12)	219546.4 (210304.59 to 224709.93)	111.71 (108.15 to 113.74)
**2012**	533089.32 (516142.31 to 542283.45)	138.31 (134.88 to 140.43)	307341.35 (299816.93 to 312489.36)	165.2 (161.44 to 167.95)	225747.97 (215342.24 to 231050.08)	113.24 (109.54 to 115.41)
**2013**	539002.61 (521661.5 to 548735)	137.57 (134.05 to 139.73)	308181.34 (300253.93 to 313261.33)	162.97 (158.98 to 165.59)	230821.27 (219993.92 to 236516.03)	113.9 (110.1 to 116.13)
**2014**	552079.08 (533940.61 to 562420.5)	138.39 (134.81 to 140.6)	317236.37 (308718.66 to 322626.71)	164.81 (160.64 to 167.53)	234842.71 (224031.72 to 240791.19)	113.69 (109.96 to 116.12)
**2015**	562329.86 (543860.94 to 572646.53)	138.86 (135.39 to 140.96)	323420.42 (314531.09 to 328749.37)	165.23 (161.01 to 167.85)	238909.44 (227792.27 to 244903)	114.21 (110.56 to 116.47)
**2016**	577332.56 (557086.57 to 587654.1)	140.52 (136.85 to 142.59)	332476.34 (323863.27 to 338167.11)	167.21 (163.53 to 170)	244856.22 (233818.17 to 250765.03)	115.57 (112.18 to 117.75)
**2017**	578105.71 (558004.11 to 588575.34)	138.56 (134.99 to 140.65)	331876.05 (323024.97 to 337682.75)	164.13 (160.33 to 166.88)	246229.65 (234799.46 to 252164.46)	114.74 (111.11 to 116.84)
**2018**	578231.05 (559077.75 to 589477.23)	136.74 (133.45 to 138.97)	334075.09 (325734.1 to 340577.08)	163.01 (159.11 to 166.04)	244155.96 (232319.15 to 250611.94)	112.24 (108.71 to 114.48)
**2019**	578325.76 (559259.89 to 590022.49)	134.63 (131.29 to 136.87)	335157.88 (325963.51 to 342304.84)	160.82 (156.9 to 164.05)	243167.87 (231325.3 to 249714.82)	110.26 (106.66 to 112.59)
**2020**	579405.52 (556934.82 to 592929.21)	132.74 (128.82 to 135.47)	336019.78 (325197.94 to 343828.2)	158.9 (154.27 to 162.34)	243385.74 (230914.24 to 250768.44)	108.36 (104.65 to 111.04)
**2021**	583986.83 (560205.4 to 599074.57)	131.78 (127.25 to 134.95)	338069.81 (325335.24 to 347969.68)	157.74 (152.17 to 162.34)	245917.02 (232317.57 to 254689.71)	107.62 (103.35 to 111.17)
**Percentage Change, 1990-2021**	36.17% (32.34% to 39.4%)	-16.06% (-18.03% to -14%)	38.65% (34.21% to 42.72%)	-16.19% (-18.88% to -13.52%)	32.9% (28.74% to 36.09%)	-16.61% (-18.9% to -14.57%)
**Statsitical Analysis**	ρ = 0.9971 p-value < 0.0001	ρ = -0.8853 p-value < 0.0001	ρ = 0.9966 p-value < 0.0001	ρ = -0.9005 p-value < 0.0001	ρ = 0.9929 p-value < 0.0001	ρ = -0.8888 p-value < 0.0001

Wilcoxon rank sum test between between sex (count): p-value < 0.001
Wilcoxon rank sum test between sex (rate): p-value < 0.001

© 2025 GBD 2021 US CNS Cancer Collaborators. *JAMA Neurology*.

eTable 3a: Incidence count and age-standardized rate of Brain and central nervous system cancer by state and sex, 2021, and percentage change from 1990 to 2021, United States

	Male			Female		
	Absolute number, 2021	Age-standardized rate, per 100 000 people, 2021	Percentage Change, 1990-2021	Absolute number, 2021	Age-standardized rate, per 100 000 people, 2021	Percentage Change, 1990-2021
**United States of America**	17459.65 (16653.75 to 18044.82)	7.96 (7.64 to 8.22)	-2.02% (-5.3% to 1.38%)	14320.35 (13269.49 to 14929.73)	5.97 (5.66 to 6.21)	-1.75% (-5.32% to 1.71%)
**Alabama**	311.25 (250.58 to 379.05)	9.36 (7.65 to 11.29)	6.63% (-15% to 29.32%)	265.95 (218.69 to 318.61)	7.08 (5.78 to 8.45)	9.43% (-13.29% to 33.35%)
**Alaska**	37.44 (29.74 to 46.95)	8.53 (6.56 to 11.68)	17.42% (-10.98% to 59.85%)	25.1 (21.75 to 29.19)	6.21 (5.28 to 7.26)	20.44% (-0.08% to 45.19%)
**Arizona**	384.97 (314.81 to 469.32)	8.1 (6.71 to 9.7)	10.11% (-10.31% to 35.04%)	304.17 (245.05 to 369.45)	5.94 (4.75 to 7.18)	6.93% (-14.29% to 30.56%)
**Arkansas**	176.78 (140.44 to 212.5)	8.98 (7.31 to 10.71)	5.12% (-14.38% to 26.95%)	152.53 (122.56 to 186.08)	7.14 (5.8 to 8.71)	1.16% (-20.1% to 25.2%)
**California**	1834.93 (1473.78 to 2231.39)	7.21 (5.88 to 8.71)	-4.94% (-22.92% to 15.79%)	1387.11 (1150.96 to 1675.88)	5.2 (4.38 to 6.22)	-7.93% (-22.89% to 11.42%)
**Colorado**	302.28 (236.94 to 374.32)	8.11 (6.45 to 9.91)	-0.83% (-20.7% to 21.61%)	244.6 (194.28 to 298.1)	5.96 (4.77 to 7.33)	-2.07% (-21.74% to 19.68%)
**Connecticut**	238.75 (187.41 to 304.8)	9.61 (7.71 to 12.14)	-2.99% (-23.51% to 23.59%)	202.53 (163.14 to 250.99)	7.12 (5.71 to 8.69)	-5.95% (-26.8% to 17.48%)
**Delaware**	52.92 (44.16 to 62.5)	7.94 (6.66 to 9.35)	5.85% (-12.03% to 28.22%)	40.65 (34.92 to 46.47)	5.32 (4.58 to 6.13)	-5.31% (-21.12% to 10.64%)
**District of Columbia**	19.69 (15.55 to 24.62)	5.03 (4.01 to 6.28)	-29.58% (-46.36% to -12.06%)	16.32 (13.65 to 19.55)	3.78 (3.14 to 4.52)	-16.89% (-31.83% to 0.13%)
**Florida**	1141.13 (922.97 to 1397.41)	7.35 (6.01 to 8.87)	-1.22% (-20% to 21.69%)	873.55 (712.98 to 1051.2)	5.27 (4.41 to 6.32)	-2.48% (-19.18% to 19.17%)
**Georgia**	492.75 (399.96 to 601)	7.4 (6.06 to 8.88)	-3.06% (-21.72% to 18.79%)	451.89 (367.82 to 543.95)	5.96 (4.86 to 7.17)	0.65% (-16.89% to 22.64%)
**Hawaii**	55.99 (44.18 to 69)	5.51 (4.41 to 6.72)	5.53% (-17.78% to 32.05%)	42.43 (35.08 to 50.46)	3.94 (3.3 to 4.63)	-5.94% (-22.72% to 14.05%)
**Idaho**	110.16 (88.69 to 137.15)	9.04 (7.35 to 11.1)	3.82% (-17.22% to 30.27%)	75.99 (63.91 to 89.99)	5.86 (4.89 to 6.93)	-1.51% (-18.15% to 17.71%)
**Illinois**	650.42 (518.14 to 808.41)	7.86 (6.31 to 9.61)	0.25% (-19.83% to 22.05%)	503.39 (402.69 to 618.25)	5.52 (4.53 to 6.69)	-2.98% (-19.97% to 19.18%)
**Indiana**	370.91 (297.65 to 447.03)	8.35 (6.8 to 10)	0.14% (-18.88% to 21.68%)	312.85 (256.19 to 383.16)	6.59 (5.39 to 7.99)	6.45% (-14.42% to 32.16%)
**Iowa**	202.08 (159.87 to 246.61)	9.14 (7.24 to 11.15)	13.08% (-10.81% to 39.51%)	167.34 (135.87 to 203.65)	7.19 (5.83 to 8.81)	5.79% (-16.61% to 33.44%)
**Kansas**	179.41 (140.6 to 222.35)	9.41 (7.46 to 11.6)	5.32% (-15.84% to 30.67%)	146.19 (117.9 to 176.61)	7.12 (5.75 to 8.63)	11.34% (-10.9% to 39.43%)
**Kentucky**	292.24 (235.17 to 356.73)	9.83 (7.9 to 11.91)	0.84% (-19.4% to 23.12%)	263.59 (212.91 to 323.93)	8.14 (6.67 to 9.98)	7.28% (-13.63% to 33.4%)
**Louisiana**	272.99 (218.09 to 330.45)	9.25 (7.48 to 11.19)	8.53% (-13.86% to 32.46%)	220.29 (179.88 to 266.41)	6.9 (5.64 to 8.4)	13.64% (-8.33% to 40.43%)

© 2025 GBD 2021 US CNS Cancer Collaborators. *JAMA Neurology*.

**Maine**	97.35<br>(76.86 to 124.45)	9.48<br>(7.52 to 11.83)	11.52%<br>(-12.98% to 41.62%)	71.45<br>(57.52 to 85.86)	6.14<br>(4.98 to 7.48)	0.59%<br>(-19.69% to 23.63%)
**Maryland**	282.09<br>(222.59 to 346.02)	7.18<br>(5.72 to 8.76)	-11.63%<br>(-30.53% to 7.46%)	232.68<br>(186.67 to 290.9)	5.24<br>(4.26 to 6.52)	-9.77%<br>(-29.63% to 15.44%)
**Massachusetts**	415.61<br>(321.88 to 538.59)	8.49<br>(6.71 to 10.9)	-4.57%<br>(-25.54% to 22.76%)	343.93<br>(267.94 to 422.09)	6.02<br>(4.78 to 7.5)	-8.85%<br>(-28.36% to 12.9%)
**Michigan**	550.24<br>(437.95 to 668.97)	7.88<br>(6.4 to 9.49)	-3.05%<br>(-23.45% to 17.26%)	537.66<br>(430.5 to 653.25)	6.96<br>(5.61 to 8.51)	-0.08%<br>(-19.51% to 23.45%)
**Minnesota**	345.19<br>(271.48 to 430.23)	9.11<br>(7.25 to 11.35)	-2.19%<br>(-22.56% to 21.37%)	269.98<br>(214.85 to 335.31)	6.67<br>(5.4 to 8.25)	-0.94%<br>(-22.9% to 25.99%)
**Mississippi**	169.08<br>(137.18 to 205.69)	8.98<br>(7.42 to 10.89)	14.08%<br>(-7.61% to 40.21%)	160.37<br>(132.68 to 192.51)	7.68<br>(6.34 to 9.24)	15.81%<br>(-5.21% to 41.88%)
**Missouri**	360.29<br>(292.9 to 440.4)	8.81<br>(7.13 to 10.63)	6.07%<br>(-15.55% to 30.81%)	291.37<br>(235.34 to 350.78)	6.44<br>(5.22 to 7.71)	5.39%<br>(-15.28% to 29.08%)
**Montana**	65.75<br>(51.79 to 80.93)	8.32<br>(6.63 to 10.22)	9.63%<br>(-13.24% to 37.98%)	51.66<br>(43.5 to 60.78)	6.65<br>(5.67 to 7.81)	10.03%<br>(-7.91% to 32.78%)
**Nebraska**	118.88<br>(94.24 to 146.3)	9.29<br>(7.36 to 11.37)	2.54%<br>(-20.23% to 29.16%)	106.13<br>(89.28 to 124.32)	7.76<br>(6.52 to 9.07)	9.14%<br>(-8.84% to 31.15%)
**Nevada**	149.17<br>(118.24 to 182.9)	7.24<br>(5.83 to 8.76)	-1.21%<br>(-21.13% to 21.15%)	109.52<br>(92.41 to 128.19)	5.09<br>(4.31 to 5.99)	-2.87%<br>(-18.65% to 18.05%)
**New Hampshire**	90.04<br>(70.28 to 113.7)	8.86<br>(6.95 to 11.26)	3.53%<br>(-20.03% to 31.57%)	65.93<br>(53.62 to 78.7)	5.91<br>(4.76 to 7.04)	4.38%<br>(-16.41% to 25.84%)
**New Jersey**	566.22<br>(449.97 to 707.94)	9.24<br>(7.43 to 11.46)	-3.56%<br>(-23.55% to 20.83%)	475.68<br>(368.61 to 587.4)	6.65<br>(5.24 to 8.23)	-6.68%<br>(-27.01% to 17.71%)
**New Mexico**	105.55<br>(84.03 to 129.89)	7.56<br>(6.1 to 9.24)	20.57%<br>(-5% to 50.39%)	82.29<br>(66.4 to 100.97)	5.48<br>(4.45 to 6.77)	18.78%<br>(-5.23% to 49.39%)
**New York**	891.31<br>(710.2 to 1104.54)	6.78<br>(5.45 to 8.29)	-5.52%<br>(-25.3% to 16.33%)	743.21<br>(595.84 to 895.42)	4.89<br>(3.97 to 5.82)	-3.5%<br>(-22.07% to 18.16%)
**North Carolina**	537<br>(429.84 to 664.37)	7.82<br>(6.33 to 9.56)	-0.19%<br>(-19.81% to 23.26%)	449.01<br>(361 to 537.35)	5.85<br>(4.73 to 7.01)	-5.32%<br>(-23.55% to 15.75%)
**North Dakota**	42.88<br>(35.07 to 52.08)	8.45<br>(7 to 10.2)	-1.84%<br>(-21.5% to 22.21%)	35.03<br>(29.71 to 42.12)	6.84<br>(5.81 to 8.14)	1.23%<br>(-15.45% to 22.72%)
**Ohio**	676.87<br>(541.06 to 820.2)	8.45<br>(6.82 to 10.15)	2.89%<br>(-16.67% to 25.25%)	556.95<br>(453.39 to 667.99)	6.24<br>(5.08 to 7.45)	5.29%<br>(-13.96% to 27.65%)
**Oklahoma**	206.99<br>(167.58 to 249.59)	8.09<br>(6.6 to 9.66)	-0.54%<br>(-19.86% to 21.54%)	177.59<br>(144.77 to 212.84)	6.46<br>(5.3 to 7.73)	3.81%<br>(-15.13% to 26.37%)
**Oregon**	277.31<br>(219.15 to 349.25)	9.17<br>(7.34 to 11.36)	-3.91%<br>(-23.43% to 19.09%)	199.88<br>(161.23 to 247.57)	6.21<br>(5.09 to 7.61)	-11.04%<br>(-28.1% to 10.91%)
**Pennsylvania**	747.69<br>(600.72 to 926.02)	8.34<br>(6.87 to 10.21)	-1.58%<br>(-20.97% to 20.57%)	585.01<br>(467.32 to 719.97)	5.76<br>(4.73 to 7.05)	-6.27%<br>(-24.16% to 16.72%)
**Rhode Island**	60.72<br>(46.5 to 76.34)	7.96<br>(6.14 to 10)	-10.53%<br>(-32.33% to 15.5%)	51.08<br>(40.86 to 61.22)	5.87<br>(4.84 to 7.01)	-9.68%<br>(-27.97% to 10.59%)
**South Carolina**	297.47<br>(240.49 to 360.39)	8.6<br>(6.99 to 10.43)	2.72%<br>(-16.91% to 25.94%)	252.57<br>(202.39 to 307.7)	6.55<br>(5.29 to 7.92)	7.76%<br>(-13.79% to 32.2%)
**South Dakota**	55.35<br>(45.31 to 66.29)	9.46<br>(7.83 to 11.36)	7.61%<br>(-12.73% to 32.52%)	42.67<br>(35.62 to 49.21)	7<br>(5.99 to 8.07)	2.01%<br>(-15.67% to 22.53%)
**Tennessee**	410.14<br>(331.7 to 499)	9.07<br>(7.36 to 10.87)	0.96%<br>(-18.73% to 22.62%)	339.8<br>(274.98 to 412.09)	6.71<br>(5.44 to 8.14)	6.94%<br>(-14.82% to 31.72%)

© 2025 GBD 2021 US CNS Cancer Collaborators. *JAMA Neurology*.

**Texas**	1301.96 (1064.53 to 1546.19)	7.36 (6.12 to 8.7)	-9.39% (-25.46% to 8.77%)	1089.8 (893.72 to 1291.43)	5.69 (4.68 to 6.74)	-9.27% (-26.59% to 7.69%)
**Utah**	142.79 (113.92 to 175.96)	7.82 (6.25 to 9.59)	2.24% (-18.87% to 27.48%)	112.7 (95.65 to 133.29)	5.84 (4.97 to 6.9)	3.2% (-14.19% to 23.82%)
**Vermont**	40.25 (33.16 to 48.39)	8.53 (7.08 to 10.2)	7.05% (-12.72% to 28.38%)	29.85 (25.2 to 35.04)	5.99 (5.07 to 6.99)	5.48% (-13.59% to 25.38%)
**Virginia**	437.51 (347.07 to 540.48)	7.81 (6.29 to 9.55)	-1.18% (-20.35% to 23.16%)	358.26 (284.34 to 440.24)	5.85 (4.7 to 7.12)	3.05% (-17.82% to 28.98%)
**Washington**	392.71 (316.06 to 473.99)	7.43 (6.06 to 8.98)	-13.62% (-30.75% to 5.3%)	390.36 (314.48 to 480.43)	6.89 (5.58 to 8.43)	0.51% (-20.02% to 24.03%)
**West Virginia**	113.81 (90.34 to 139.27)	8.78 (7.06 to 10.54)	21.6% (-3.63% to 46.78%)	94.37 (77.14 to 115.05)	6.89 (5.62 to 8.42)	24.81% (-0.49% to 53.57%)
**Wisconsin**	350.97 (273.05 to 433.34)	8.64 (6.88 to 10.55)	-3.13% (-24.51% to 20.13%)	293.46 (231.66 to 357.19)	6.91 (5.55 to 8.5)	0.64% (-20.26% to 25.3%)
**Wyoming**	33.37 (27.89 to 40.15)	8.28 (6.93 to 9.81)	6.53% (-12.25% to 28.25%)	23.66 (20.13 to 27.61)	5.99 (5.1 to 6.97)	-0.09% (-15.82% to 18.46%)

© 2025 GBD 2021 US CNS Cancer Collaborators. *JAMA Neurology*.

eTable 3b: DALY count and age-standardized rate of Brain and central nervous system cancer by state and sex, 2021, and percentage change from 1990 to 2021, United States

	Male			Female		
	Absolute number, 2021	Age-standardized rate, per 100 000 people, 2021	Percentage Change, 1990-2021	Absolute number, 2021	Age-standardized rate, per 100 000 people, 2021	Percentage Change, 1990-2021
**United States of America**	344129.03 (330881.91 to 353859.18)	160.7 (155.05 to 165.07)	-15.91% (-18.61% to -13.18%)	250867.11 (237587.77 to 259574.57)	109.9 (105.59 to 113.56)	-16.3% (-18.59% to -14.3%)
**Alabama**	6856.92 (5522.15 to 8255.23)	213.71 (174.31 to 254.57)	-3.61% (-22.8% to 16.35%)	5244.28 (4272.1 to 6298.85)	149.28 (123.05 to 177.3)	-1.71% (-20.25% to 17.59%)
**Alaska**	772.23 (624.99 to 964.42)	174.74 (137.37 to 231.6)	-0.19% (-22.64% to 31.52%)	464.18 (411.5 to 531.89)	115.42 (102.53 to 131.15)	0.54% (-12.06% to 15.64%)
**Arizona**	7390.05 (6003.5 to 8922.64)	161.11 (133.09 to 191.94)	-7.03% (-22.84% to 12.65%)	5211.18 (4258.67 to 6266.66)	106.89 (88.15 to 127.58)	-11.04% (-26.36% to 6.27%)
**Arkansas**	3849.76 (3064.01 to 4659.69)	203.75 (165.02 to 244.83)	-4.03% (-21.92% to 16.91%)	3002.91 (2455.45 to 3593.34)	150.48 (124.69 to 179.18)	-7.96% (-24.88% to 10.97%)
**California**	36238.94 (29388.36 to 43990.99)	143.37 (117.22 to 173.68)	-21.2% (-35.69% to -5.46%)	25075.3 (20716.47 to 30041.79)	96.55 (81.19 to 114.21)	-23.46% (-34.97% to -9.14%)
**Colorado**	5772.64 (4535.16 to 7153.8)	155.64 (125.15 to 191.09)	-15.61% (-32.05% to 3.11%)	4127.54 (3312.3 to 4977.65)	102.7 (83.14 to 123.51)	-17.32% (-33.33% to 0.24%)
**Connecticut**	3624.71 (2872.61 to 4600.86)	149.05 (119.47 to 186.8)	-18.64% (-36.25% to 1.16%)	2680.32 (2166.03 to 3311.74)	101.37 (82.76 to 123.92)	-17.95% (-33.77% to 0.57%)
**Delaware**	1054.28 (891.13 to 1256.66)	164 (138.59 to 193.77)	-7.26% (-23.42% to 11.13%)	718.6 (626.85 to 813.62)	99.11 (86.73 to 111.55)	-21.16% (-31.83% to -9.49%)
**District of Columbia**	459.92 (361.04 to 570.25)	118.92 (94.09 to 145.8)	-47.21% (-59.23% to -33.96%)	343.78 (291.93 to 409.15)	84.62 (72.9 to 98.87)	-38.04% (-47.17% to -26.38%)
**Florida**	23883.02 (19287.97 to 29067.28)	162.44 (133.28 to 195.06)	-15.98% (-31.87% to 2.61%)	17142.08 (14178.86 to 20682.14)	109.32 (92.36 to 130.56)	-17.97% (-31.21% to -2.66%)
**Georgia**	10262.87 (8347.09 to 12537.87)	157.44 (130.06 to 190.46)	-18.41% (-33.91% to -0.96%)	8275.44 (6763.42 to 9953.65)	114.07 (94.89 to 135.37)	-17.89% (-31.94% to -1.91%)
**Hawaii**	1061.86 (841.28 to 1303.08)	108.42 (87 to 133.01)	-9.41% (-27.97% to 12.79%)	703.85 (592.38 to 819.22)	69.74 (59.49 to 80.38)	-21.64% (-32.73% to -8.52%)
**Idaho**	2114.07 (1687.35 to 2620.7)	178.67 (144.23 to 218.5)	-10.14% (-28.47% to 12.05%)	1318.26 (1117.9 to 1531.16)	105.9 (90.31 to 122.89)	-15.23% (-28.01% to -0.98%)
**Illinois**	12641.73 (10102.05 to 15670.9)	155.58 (126.17 to 189.98)	-17.32% (-33.4% to 0.16%)	8754.13 (7169.81 to 10504.87)	100.43 (83.7 to 119.06)	-21.3% (-34.88% to -6.2%)
**Indiana**	7515.86 (6119.82 to 9158.04)	173.18 (142.32 to 208.78)	-10.23% (-26.94% to 9.24%)	5761.74 (4708.27 to 6993.54)	127.8 (105.37 to 153.55)	-4.48% (-21.55% to 13.27%)
**Iowa**	3812.55 (3038.43 to 4655.13)	180.24 (144.8 to 218.22)	-4.04% (-23.57% to 19.58%)	2658.21 (2163.87 to 3209.12)	124.4 (101.37 to 149)	-2.89% (-21.35% to 18.72%)
**Kansas**	3500.01 (2757.41 to 4324.77)	188.17 (149.72 to 230.85)	-4.38% (-23.6% to 17.17%)	2565.12 (2085.22 to 3102.85)	131.63 (109.23 to 159.24)	1.15% (-17.99% to 22.97%)
**Kentucky**	5585.09 (4464 to 6793.5)	189.9 (153.66 to 228.77)	-4.92% (-23.43% to 16.43%)	4349.64 (3576.31 to 5372.6)	142.64 (118.62 to 175.15)	-1.06% (-18.69% to 20.81%)
**Louisiana**	5502.48 (4469.94 to 6709.12)	190.52 (155.89 to 231.05)	-2.5% (-21.38% to 17.49%)	3866.22 (3204.46 to 4642.3)	127.28 (107.28 to 151.82)	-3.68% (-19.7% to 14.29%)

© 2025 GBD 2021 US CNS Cancer Collaborators. *JAMA Neurology*.

**Maine**	1873.36 (1463.99 to 2334.73)	190.32 (151.8 to 235.2)	2.74% (-19.54% to 27.25%)	1200.64 (989.8 to 1415.5)	109.46 (90.69 to 129.86)	-10.71% (-26.66% to 5.65%)
**Maryland**	5698.85 (4447.91 to 6984.94)	147.61 (117.34 to 179.68)	-24.46% (-40.21% to -7.26%)	4227.06 (3431.93 to 5198.39)	99.59 (82.27 to 121.04)	-22.74% (-37.56% to -4.58%)
**Massachusetts**	7273.03 (5621.32 to 9456.86)	150.43 (117.74 to 194.22)	-21.35% (-38.16% to 1.46%)	5126.1 (4079.5 to 6221.78)	95.2 (77.39 to 114.32)	-27.22% (-40.63% to -11.45%)
**Michigan**	11207.19 (9072.65 to 13452.08)	169.92 (139.51 to 202.86)	-15.71% (-32.56% to 1.19%)	8738.3 (7046.38 to 10557.2)	120.74 (99.48 to 144.73)	-14.76% (-29.47% to 2.07%)
**Minnesota**	6111.89 (4776.51 to 7562.7)	162.96 (130.42 to 199.57)	-15.88% (-33.47% to 2.19%)	4272.78 (3465.93 to 5194.76)	109.84 (90.26 to 132.97)	-14.14% (-30.32% to 4.57%)
**Mississippi**	3864.02 (3125.52 to 4699.59)	212.86 (175.39 to 256.64)	3.81% (-16.07% to 27.54%)	3355.25 (2757.76 to 4008.07)	171.5 (143.24 to 203.44)	5.26% (-11.51% to 26.62%)
**Missouri**	7282.83 (5896.6 to 8904.88)	183.82 (149.51 to 221.77)	-6.39% (-24.97% to 13.86%)	5276.91 (4248.58 to 6358.8)	122.92 (99.97 to 147.36)	-7.11% (-23.82% to 11.13%)
**Montana**	1293.32 (1020.81 to 1608.07)	172.28 (138.53 to 212.08)	-0.92% (-22.46% to 23.87%)	928.32 (808.25 to 1071)	126.57 (111.7 to 144.01)	-2.57% (-16.16% to 13.27%)
**Nebraska**	2239.32 (1746.76 to 2753.2)	179.39 (142.14 to 218.58)	-9.16% (-29.45% to 13.8%)	1771.08 (1491.1 to 2062.22)	137.44 (117.74 to 158.17)	-5.48% (-19.65% to 10.48%)
**Nevada**	3013.44 (2404.33 to 3672.98)	148.71 (120.86 to 179.85)	-16.27% (-32.46% to 2.28%)	2004.53 (1731.03 to 2331.58)	96.46 (84.25 to 111.83)	-17.97% (-28.75% to -4.71%)
**New Hampshire**	1649.78 (1285.41 to 2086.24)	164.98 (129.62 to 205.4)	-10.84% (-31.05% to 13.31%)	1071.42 (890.13 to 1275.67)	98.8 (83.74 to 116.13)	-13.03% (-27.32% to 3.37%)
**New Jersey**	8715.14 (6826.26 to 10741.95)	140.92 (112.09 to 172.17)	-22.28% (-38.89% to -3.76%)	6402.48 (5078.43 to 7789.38)	92.94 (74.54 to 112.13)	-24.32% (-39.06% to -6.83%)
**New Mexico**	2269.4 (1796.75 to 2777.64)	169.84 (136.9 to 207.9)	7.58% (-14.75% to 31.74%)	1581.81 (1294.41 to 1923.32)	111.41 (91.67 to 135.5)	3.67% (-15.77% to 27.84%)
**New York**	16838 (13448.92 to 20689.4)	130.41 (105.69 to 157.64)	-26.26% (-40.18% to -9.74%)	12327.78 (10027.78 to 14761.57)	85.81 (71.21 to 101.14)	-25.95% (-38.49% to -12.14%)
**North Carolina**	10862.15 (8707.7 to 13257.28)	162.66 (131.99 to 196.54)	-15.32% (-31.48% to 4.91%)	8207.09 (6637.88 to 9762.51)	112.65 (93.1 to 133.11)	-20.28% (-34.26% to -4.77%)
**North Dakota**	843.73 (692.6 to 1017.42)	171.63 (142.03 to 204.71)	-9.21% (-26.03% to 9.48%)	604.87 (522.97 to 709.15)	126.49 (109.86 to 146.66)	-8.92% (-21.83% to 5.28%)
**Ohio**	13779.62 (11005.18 to 16815.17)	177.67 (144.29 to 214.57)	-7.87% (-26.25% to 12.18%)	10159.2 (8459.4 to 12020.65)	120.72 (100.92 to 142.09)	-6.18% (-22.08% to 11.28%)
**Oklahoma**	4475.32 (3654.55 to 5452.77)	180.95 (148.9 to 218.32)	-4.99% (-22.14% to 15.33%)	3511.46 (2931.76 to 4242.25)	135.56 (113.93 to 162.19)	0.43% (-16.78% to 20.55%)
**Oregon**	5098 (4092.28 to 6331.82)	173.76 (141.28 to 213.65)	-17.2% (-33.21% to 1.84%)	3272.65 (2641.81 to 4062.32)	106.69 (87.34 to 130.42)	-24.4% (-38.34% to -8.01%)
**Pennsylvania**	14466.69 (11528.72 to 17709.21)	166.84 (135.15 to 202.64)	-15.74% (-31.99% to 3.19%)	9899.28 (8001.79 to 11761.58)	104.58 (86.85 to 124.02)	-20.94% (-34.32% to -6.08%)
**Rhode Island**	1119.88 (866.68 to 1398.23)	149.91 (116.61 to 187.2)	-24.58% (-42.67% to -2.85%)	822.81 (677.99 to 988.55)	101.08 (84.26 to 120.46)	-25.86% (-38.74% to -11.53%)
**South Carolina**	6276.85 (5020.55 to 7686.01)	188.56 (152.65 to 228.42)	-12.73% (-29.12% to 5.63%)	4832.76 (3958.41 to 5829.45)	133.25 (109.97 to 158.68)	-10.42% (-25.91% to 8.31%)
**South Dakota**	1109.55 (916.62 to 1347.81)	198.03 (165.42 to 237.77)	-1.88% (-18.76% to 20.61%)	773.41 (663.58 to 898.03)	135.51 (116.89 to 156.1)	-8.54% (-21.84% to 6.99%)
**Tennessee**	8708.51 (6990.1 to 10736.96)	198.55 (161.79 to 241.18)	-10.37% (-27.02% to 9.31%)	6514.17 (5386.91 to 7893.73)	135.1 (111.26 to 163.12)	-5.65% (-22.94% to 14.94%)

© 2025 GBD 2021 US CNS Cancer Collaborators. *JAMA Neurology*.

**Texas**	25982.97 (21402.56 to 31093.63)	147.06 (122.63 to 174.49)	-24.04% (-37% to -9.67%)	19814.22 (16502.92 to 23298.55)	106.35 (89.16 to 124.17)	-23.34% (-35.48% to -10.66%)
**Utah**	2773.84 (2231.26 to 3387.48)	152.3 (123.73 to 185.24)	-13.68% (-30.32% to 6.65%)	1969.01 (1705.08 to 2296.11)	104.54 (91.23 to 121.34)	-13.82% (-26.24% to 1.66%)
**Vermont**	757.08 (626.25 to 909.64)	166.04 (138.13 to 197.96)	-8.87% (-25.3% to 9.88%)	491.22 (423.97 to 570.28)	104.31 (90.15 to 119.46)	-13.93% (-26.48% to -0.59%)
**Virginia**	8399.51 (6678.57 to 10369.81)	151.92 (123.33 to 185.96)	-17.2% (-33.28% to 1.66%)	6173.19 (5054.99 to 7453.46)	104.44 (86.51 to 125.01)	-14.23% (-29.41% to 3.86%)
**Washington**	8493.53 (6806.1 to 10251.44)	165.66 (134.16 to 197.56)	-21.41% (-36.95% to -6.09%)	6094.74 (4996.79 to 7511.88)	113.68 (94.69 to 138.8)	-17.62% (-31.68% to 1.2%)
**West Virginia**	2466.24 (1973.6 to 3036.11)	200.58 (163.14 to 243.59)	15.31% (-7.15% to 39.58%)	1819.11 (1480.16 to 2196.73)	143.04 (116.93 to 173.03)	14.29% (-6.8% to 39.6%)
**Wisconsin**	6617.86 (5169.69 to 8215.37)	166.45 (132.2 to 202.22)	-13.24% (-31.52% to 7.09%)	4935.97 (4009.59 to 5936.59)	122.09 (100.45 to 146.29)	-10.21% (-26.37% to 9.17%)
**Wyoming**	669.16 (563.72 to 801.22)	170.63 (144.56 to 203.14)	-5.21% (-21.26% to 12.15%)	424.68 (366.33 to 486.17)	112.22 (98.28 to 129.35)	-13.08% (-24.28% to 0.09%)

© 2025 GBD 2021 US CNS Cancer Collaborators. *JAMA Neurology*.

eTable 3c: Death count and age-standardized rate of Brain and central nervous system cancer by state and sex, 2021, and percentage change from 1990 to 2021, United States

	Male			Female		
	Absolute number, 2021	Age-standardized rate, per 100 000 people, 2021	Percentage Change, 1990-2021	Absolute number, 2021	Age-standardized rate, per 100 000 people, 2021	Percentage Change, 1990-2021
**United States of America**	12132.27 (11497.15 to 12524.76)	4.99 (4.76 to 5.14)	-8.84% (-11.72% to -5.95%)	9311.81 (8488.03 to 9745.27)	3.32 (3.09 to 3.45)	-9.18% (-12.11% to -6.85%)
**Alabama**	236.71 (190.01 to 285.23)	6.5 (5.25 to 7.8)	3.64% (-17.12% to 26.42%)	194.39 (157.97 to 232.05)	4.51 (3.69 to 5.4)	6.84% (-13.18% to 27.98%)
**Alaska**	23.23 (19.17 to 28.21)	4.74 (3.85 to 5.8)	-4.57% (-23.99% to 18.01%)	14.09 (12.3 to 16.24)	3.05 (2.66 to 3.51)	-5.37% (-17.7% to 10.07%)
**Arizona**	261.35 (212.24 to 318.33)	4.89 (4 to 5.91)	2.9% (-15.99% to 26.54%)	193.27 (157.33 to 233.48)	3.19 (2.61 to 3.82)	-1.03% (-18.43% to 19.84%)
**Arkansas**	131.34 (104.56 to 160.14)	6.07 (4.85 to 7.34)	1.93% (-18.68% to 24.85%)	108.03 (87.21 to 130.25)	4.37 (3.57 to 5.22)	-2.41% (-21.3% to 17.23%)
**California**	1281.19 (1031.43 to 1554.64)	4.57 (3.69 to 5.53)	-10.78% (-27.81% to 7.7%)	929.47 (761.98 to 1128.63)	2.93 (2.43 to 3.53)	-15.44% (-29.73% to 1.26%)
**Colorado**	196.66 (154 to 245.65)	4.76 (3.76 to 5.93)	-9.35% (-27.33% to 11.56%)	152.13 (120.91 to 182.31)	3.22 (2.59 to 3.85)	-10.07% (-27.25% to 9.23%)
**Connecticut**	129.78 (101.81 to 163.87)	4.61 (3.65 to 5.82)	-12.3% (-31.44% to 9.9%)	103.84 (84.46 to 127.46)	3.09 (2.51 to 3.79)	-11.36% (-28.68% to 10.01%)
**Delaware**	36.57 (30.65 to 43.63)	4.87 (4.12 to 5.79)	-8.18% (-24.16% to 10.49%)	27.03 (23.28 to 30.83)	2.96 (2.58 to 3.35)	-16.53% (-27.15% to -4.39%)
**District of Columbia**	14.55 (11.41 to 18.13)	3.52 (2.78 to 4.38)	-38.34% (-52.98% to -22.71%)	11.55 (9.57 to 13.75)	2.43 (2.05 to 2.87)	-29.71% (-40.71% to -15.26%)
**Florida**	887.57 (704 to 1083.14)	4.97 (4.03 to 6.04)	-8.82% (-26.59% to 12.51%)	663.29 (536.86 to 797.79)	3.23 (2.67 to 3.88)	-10.2% (-25.41% to 7.58%)
**Georgia**	346.39 (279.58 to 428.63)	4.83 (3.93 to 5.95)	-11.83% (-29.5% to 8.22%)	295.89 (239.15 to 356.54)	3.46 (2.83 to 4.15)	-9.12% (-25.8% to 9.49%)
**Hawaii**	37.75 (29.76 to 46.83)	3.27 (2.59 to 4.03)	-3.07% (-23.82% to 21.25%)	26.74 (21.96 to 31.61)	2.07 (1.73 to 2.42)	-13.02% (-26.58% to 2.08%)
**Idaho**	75.38 (60.14 to 94.36)	5.6 (4.49 to 6.95)	-2.63% (-23.87% to 23.18%)	49.5 (41.66 to 57.66)	3.37 (2.84 to 3.91)	-6.07% (-20.48% to 9.04%)
**Illinois**	441.35 (353.29 to 550.23)	4.81 (3.87 to 5.95)	-10.81% (-28.12% to 8.74%)	322.12 (256.46 to 385.86)	3 (2.44 to 3.58)	-15.33% (-30.02% to 1.72%)
**Indiana**	265.49 (212.62 to 325.38)	5.43 (4.39 to 6.59)	-3.14% (-21.65% to 18.56%)	207.64 (168.99 to 252.42)	3.75 (3.06 to 4.56)	2.41% (-16.17% to 22.9%)
**Iowa**	138 (109.1 to 169.08)	5.64 (4.49 to 6.86)	2.55% (-19.14% to 28.31%)	100.12 (81.13 to 122.21)	3.66 (2.97 to 4.46)	1.44% (-18.55% to 25.13%)
**Kansas**	122.58 (96.43 to 151.94)	5.84 (4.61 to 7.19)	2.57% (-18.34% to 25.85%)	94.44 (75.66 to 114.85)	3.97 (3.22 to 4.81)	9.39% (-13.03% to 32.47%)
**Kentucky**	191.4 (154.65 to 233.69)	5.79 (4.69 to 7.02)	0.82% (-19.23% to 23.26%)	155.69 (127.5 to 191.09)	4.16 (3.43 to 5.13)	1.83% (-16.62% to 24.33%)
**Louisiana**	182.82 (147.47 to 223.14)	5.71 (4.64 to 6.95)	4.04% (-16.25% to 27.96%)	135.51 (110.29 to 162.67)	3.71 (3.07 to 4.44)	2.79% (-15.86% to 23.82%)

© 2025 GBD 2021 US CNS Cancer Collaborators. *JAMA Neurology*.

**Maine**	68.83 (53.86 to 86.46)	5.79 (4.58 to 7.19)	4.75% (-18.85% to 31.62%)	48.63 (40.1 to 56.84)	3.46 (2.85 to 4.06)	-2.48% (-19.41% to 14.85%)
**Maryland**	193.82 (150.58 to 237.35)	4.47 (3.49 to 5.44)	-21.04% (-38.21% to -2.6%)	151.25 (122.02 to 185.59)	2.91 (2.36 to 3.57)	-21.17% (-36.33% to -3.06%)
**Massachusetts**	274.66 (213.7 to 353.39)	5.07 (3.97 to 6.52)	-10.67% (-30.22% to 15.54%)	212.61 (166.53 to 257.41)	3.17 (2.52 to 3.83)	-15.54% (-31.54% to 2.69%)
**Michigan**	397.2 (315.95 to 476.76)	5.13 (4.15 to 6.12)	-11.57% (-29.83% to 7.1%)	325.95 (260.53 to 390.9)	3.6 (2.9 to 4.34)	-6.68% (-23.59% to 13.13%)
**Minnesota**	217.89 (171.45 to 271.24)	5.06 (4 to 6.23)	-11.08% (-29.92% to 9.25%)	158.98 (126.97 to 192.5)	3.31 (2.66 to 4.02)	-10.36% (-27.01% to 9.71%)
**Mississippi**	130.2 (105.89 to 159.87)	6.37 (5.25 to 7.77)	11.26% (-11.08% to 36.19%)	117.42 (96.96 to 140.29)	4.93 (4.06 to 5.88)	12.57% (-5.63% to 35.97%)
**Missouri**	254.06 (204.68 to 312.89)	5.56 (4.51 to 6.81)	0.62% (-20.19% to 23.72%)	195.69 (157.61 to 231.78)	3.69 (2.97 to 4.41)	-1.09% (-19.84% to 17.77%)
**Montana**	46.7 (36.46 to 58.04)	5.16 (4.07 to 6.4)	3.7% (-19.34% to 30.4%)	34.03 (28.82 to 39.65)	3.61 (3.13 to 4.14)	-0.46% (-15.07% to 15.91%)
**Nebraska**	79.21 (62.34 to 97.08)	5.58 (4.41 to 6.79)	-3.7% (-25.37% to 20.7%)	66.33 (55.37 to 78.28)	4.15 (3.47 to 4.85)	3.23% (-12.31% to 21.24%)
**Nevada**	105 (82.79 to 129.42)	4.65 (3.72 to 5.68)	-7.99% (-27.23% to 12.7%)	72.35 (61.9 to 84.16)	2.97 (2.56 to 3.44)	-11.42% (-23.51% to 3.49%)
**New Hampshire**	60.79 (47.51 to 76.18)	5.21 (4.1 to 6.53)	-6.17% (-26.25% to 18.79%)	42.17 (34.65 to 50.17)	3.15 (2.61 to 3.73)	-7.8% (-23.4% to 9.71%)
**New Jersey**	309.37 (241.8 to 380.79)	4.47 (3.53 to 5.47)	-13.05% (-31.53% to 7.07%)	243.98 (194.95 to 295.77)	2.91 (2.32 to 3.52)	-12.57% (-30.17% to 6.8%)
**New Mexico**	75.49 (58.53 to 91.73)	4.85 (3.84 to 5.92)	14.01% (-10.37% to 40.91%)	56.2 (45.15 to 68.35)	3.19 (2.61 to 3.88)	10.33% (-9.98% to 36.56%)
**New York**	592.59 (471.41 to 727.89)	4.03 (3.23 to 4.91)	-18.46% (-34.63% to 0.2%)	471.46 (382.81 to 568.05)	2.63 (2.14 to 3.14)	-16.04% (-30.38% to 0.34%)
**North Carolina**	384.48 (305.09 to 472.04)	5.07 (4.06 to 6.19)	-9.15% (-27.33% to 12.01%)	301.97 (243.7 to 360.91)	3.36 (2.72 to 4.01)	-15.69% (-31.19% to 0.58%)
**North Dakota**	28.29 (23.15 to 34.31)	4.95 (4.07 to 5.99)	-11.58% (-28.75% to 8.34%)	21.65 (18.31 to 25.6)	3.54 (3.05 to 4.19)	-9.33% (-23.01% to 5.82%)
**Ohio**	490.28 (390.83 to 598.24)	5.5 (4.41 to 6.68)	-0.7% (-21% to 21.87%)	384.95 (317.04 to 456.55)	3.68 (3.05 to 4.36)	3.6% (-13.7% to 22.2%)
**Oklahoma**	152.79 (124.59 to 186.86)	5.48 (4.48 to 6.66)	-0.17% (-19.21% to 23.21%)	124.99 (102.93 to 150.66)	4 (3.31 to 4.82)	3.74% (-14.58% to 24.66%)
**Oregon**	186.08 (147.92 to 234.61)	5.42 (4.34 to 6.75)	-11.71% (-30% to 8.86%)	124.99 (100.67 to 155.34)	3.24 (2.61 to 4.01)	-19.07% (-34.97% to -0.37%)
**Pennsylvania**	520.36 (409.95 to 648.15)	5.11 (4.07 to 6.28)	-10.05% (-28.65% to 10.57%)	390.15 (310.09 to 462.19)	3.2 (2.58 to 3.79)	-14.4% (-29.69% to 2.35%)
**Rhode Island**	41.68 (32.57 to 51.69)	4.88 (3.84 to 6.04)	-18.82% (-38.22% to 3.78%)	33.19 (26.9 to 39.71)	3.19 (2.62 to 3.81)	-19.35% (-34.24% to -3.12%)
**South Carolina**	219.11 (174.68 to 269.38)	5.71 (4.6 to 6.96)	-8.39% (-26.49% to 12.8%)	177.76 (145.99 to 215.14)	3.96 (3.26 to 4.75)	-2.74% (-20.31% to 18.51%)
**South Dakota**	38.36 (31.31 to 47.13)	5.82 (4.79 to 7.1)	0.08% (-17.06% to 24.7%)	28.03 (23.62 to 32.84)	3.89 (3.33 to 4.52)	-0.98% (-15.22% to 15.77%)
**Tennessee**	298.28 (237.3 to 369.5)	5.98 (4.79 to 7.36)	-6.3% (-25.04% to 14.5%)	234.1 (193.52 to 283.7)	3.99 (3.31 to 4.84)	-1.44% (-19.03% to 21.19%)

© 2025 GBD 2021 US CNS Cancer Collaborators. *JAMA Neurology*.

**Texas**	903.12<br>(741.79 to 1083.21)	4.79<br>(3.96 to 5.72)	-14.47%<br>(-29.73% to 2.34%)	701.06<br>(579.06 to 829.68)	3.26<br>(2.71 to 3.86)	-17.12%<br>(-31.67% to -3.09%)
**Utah**	92.64<br>(73.98 to 113.51)	4.9<br>(3.93 to 5.99)	-2.61%<br>(-22.3% to 20.64%)	68.18<br>(57.82 to 79.98)	3.29<br>(2.8 to 3.85)	-3.92%<br>(-17.39% to 12.1%)
**Vermont**	27.55<br>(22.66 to 33.03)	5.04<br>(4.17 to 6.02)	-8.7%<br>(-25.27% to 10.94%)	19.2<br>(16.38 to 22.35)	3.11<br>(2.69 to 3.61)	-11.79%<br>(-24.85% to 2.71%)
**Virginia**	294.32<br>(233.72 to 364.66)	4.74<br>(3.8 to 5.82)	-12.01%<br>(-29.92% to 8.98%)	225.82<br>(183.38 to 273.12)	3.14<br>(2.56 to 3.8)	-9.28%<br>(-25.85% to 10.4%)
**Washington**	302.68<br>(241.77 to 368.87)	5.2<br>(4.18 to 6.29)	-15.59%<br>(-32.85% to 1.72%)	225.9<br>(182.66 to 279.74)	3.48<br>(2.84 to 4.29)	-13.04%<br>(-28.28% to 6.7%)
**West Virginia**	85.93<br>(68.23 to 105.98)	5.9<br>(4.73 to 7.22)	15.97%<br>(-7.71% to 42.01%)	67.84<br>(55.75 to 81.37)	4.17<br>(3.42 to 5.04)	19.24%<br>(-3.03% to 45.26%)
**Wisconsin**	237.18<br>(185.84 to 294.32)	5.16<br>(4.08 to 6.35)	-8.01%<br>(-26.74% to 14.41%)	184.66<br>(148.54 to 222.06)	3.63<br>(2.92 to 4.36)	-4.92%<br>(-22.34% to 17.2%)
**Wyoming**	23.23<br>(19.48 to 27.93)	5.18<br>(4.37 to 6.18)	0.32%<br>(-17.46% to 19.74%)	15.62<br>(13.35 to 18)	3.41<br>(2.94 to 3.92)	-4.88%<br>(-17.58% to 10.39%)

© 2025 GBD 2021 US CNS Cancer Collaborators. *JAMA Neurology*.

eTable 3d: Prevalence count and age-standardized rate of Brain and central nervous system cancer by state and sex, 2021, and percentage change from 1990 to 2021, United States

	Male			Female		
	Absolute number, 2021	Age-standardized rate, per 100 000 people, 2021	Percentage Change, 1990-2021	Absolute number, 2021	Age-standardized rate, per 100 000 people, 2021	Percentage Change, 1990-2021
**United States of America**	45640.22 (43801.73 to 47232.28)	25.51 (24.32 to 26.53)	9.02% (3.72% to 14.23%)	38762.25 (36889.46 to 40440.67)	21.39 (20.33 to 22.52)	7.05% (1.63% to 12.52%)
**Alabama**	686.78 (550.93 to 825.18)	25.61 (20.7 to 30.63)	12.09% (-10.75% to 37.97%)	593.93 (472.12 to 713.62)	21.32 (17.03 to 25.76)	11.43% (-12.88% to 41.48%)
**Alaska**	119.44 (89.36 to 175.08)	32.14 (23 to 52.02)	56.06% (8.22% to 142.84%)	86.9 (71.71 to 103.6)	25.61 (20.8 to 30.99)	56.7% (21.75% to 97.45%)
**Arizona**	1030.77 (848.88 to 1249.93)	26.99 (22.41 to 32.46)	21.23% (-2.49% to 51.15%)	848.81 (668.62 to 1038.84)	22.08 (17.33 to 27.42)	14.17% (-12.67% to 50.34%)
**Arkansas**	407.73 (329.97 to 487.89)	25.51 (20.69 to 30.6)	10.58% (-11.15% to 36.59%)	365.03 (287.39 to 445.92)	22.87 (18.13 to 27.99)	4.26% (-21.17% to 33.83%)
**California**	5376.32 (4263.53 to 6578)	25.28 (20.65 to 30.99)	5.41% (-16.9% to 30.88%)	4295.18 (3517.44 to 5241.68)	21.02 (17.09 to 25.62)	1.16% (-19.13% to 27.96%)
**Colorado**	866.55 (696.06 to 1084.81)	27.88 (22.75 to 34.58)	11.51% (-11.16% to 42.43%)	699.88 (540.32 to 865.43)	21.71 (16.96 to 26.86)	6.06% (-19.05% to 39.95%)
**Connecticut**	784.56 (611.54 to 1003.74)	36.93 (29.15 to 47.42)	2.84% (-20.22% to 33.76%)	639.72 (499 to 810.9)	28.76 (22.31 to 36.36)	0.45% (-25.08% to 31.82%)
**Delaware**	136.35 (112.35 to 161.97)	25.74 (21.23 to 30.66)	31.02% (6.79% to 62.57%)	104.81 (87.8 to 123.71)	19.06 (15.6 to 22.94)	9.31% (-12.86% to 37.41%)
**District of Columbia**	45.8 (35.58 to 57.07)	13.49 (10.69 to 16.75)	-12.88% (-31.92% to 11.11%)	39.2 (32.34 to 48.24)	11.35 (9.17 to 13.96)	5.7% (-17.1% to 33.07%)
**Florida**	2541.68 (2074.88 to 3084.21)	23.82 (19.44 to 28.89)	7.54% (-12.6% to 35.81%)	2131.73 (1746.97 to 2584.73)	19.88 (16.25 to 24.23)	4.03% (-18.47% to 31.08%)
**Georgia**	1282.12 (1043.79 to 1559.16)	22.14 (18.13 to 26.64)	23.29% (-1.33% to 52.03%)	1101.14 (876.74 to 1363.09)	19.13 (15.42 to 23.57)	9.1% (-13.05% to 38.28%)
**Hawaii**	133.43 (104.99 to 165.7)	17.2 (13.49 to 21.36)	11.8% (-15.38% to 43.21%)	119.15 (97.93 to 143.86)	15.18 (12.45 to 18.46)	0.25% (-20.37% to 28.23%)
**Idaho**	292.93 (231.85 to 358.48)	28.98 (23 to 35.68)	12.37% (-13.14% to 43.25%)	201.79 (163.96 to 242.77)	19.59 (15.95 to 23.73)	-0.16% (-22.56% to 26.69%)
**Illinois**	1732.67 (1389.7 to 2123.28)	25.61 (20.64 to 30.94)	17.74% (-6.69% to 43.46%)	1386.4 (1098.31 to 1733.73)	20.33 (16.3 to 25.36)	14.32% (-10.24% to 47.99%)
**Indiana**	907.86 (735.23 to 1095.05)	25.05 (20.45 to 30.2)	5.31% (-15.91% to 30.87%)	825.68 (661.72 to 1015.61)	23.02 (18.36 to 28.47)	10.42% (-16.2% to 47.84%)
**Iowa**	396.67 (314.56 to 483.66)	21.95 (17.39 to 26.73)	21.62% (-5.86% to 53.88%)	395.53 (312.77 to 491.74)	22.96 (18.08 to 28.71)	9.84% (-18.98% to 44.41%)
**Kansas**	474.93 (378.93 to 584.71)	30.08 (24.07 to 36.62)	9.58% (-15.15% to 38.46%)	397.48 (313.57 to 495.64)	25.24 (19.95 to 31.25)	11.4% (-15.93% to 46.89%)
**Kentucky**	798.54 (632.5 to 975.98)	31.92 (25.54 to 38.72)	4.06% (-19.51% to 31.96%)	655.4 (518.82 to 837.71)	25.51 (20.4 to 32.35)	8.91% (-16.24% to 42.86%)
**Louisiana**	603.32 (484.97 to 738.23)	23.98 (19.35 to 29.22)	14.36% (-9.61% to 41.4%)	492.03 (398.03 to 606.75)	20.38 (16.41 to 25.21)	19.14% (-6.86% to 54.05%)

© 2025 GBD 2021 US CNS Cancer Collaborators. *JAMA Neurology*.

**Maine**	234.14 (182.36 to 295.53)	30.53 (24.39 to 38.05)	20.63% (-6.67% to 57.65%)	168.73 (136.28 to 208.76)	20.99 (16.87 to 26.02)	0.04% (-24.22% to 28.21%)
**Maryland**	744.67 (597.56 to 931.81)	23.03 (18.71 to 28.35)	6.01% (-14.74% to 32.81%)	627.73 (498.2 to 791.33)	18.81 (14.76 to 23.72)	6.53% (-19.76% to 40.49%)
**Massachusetts**	1120.01 (897.25 to 1460.52)	27.77 (22.36 to 35.47)	2.28% (-20.65% to 34.15%)	944.92 (725.59 to 1200.93)	22.29 (17.44 to 28.02)	-3.39% (-26.29% to 25.31%)
**Michigan**	1234.8 (1001.8 to 1503.11)	21.63 (17.85 to 26.23)	6.39% (-14.67% to 31.57%)	1328.13 (1045.88 to 1675.21)	21.28 (16.74 to 26.41)	14.54% (-11.25% to 48.15%)
**Minnesota**	1035.56 (838.17 to 1318.87)	33.75 (27.42 to 42.56)	12.12% (-12.22% to 44.11%)	835.39 (651.23 to 1051.91)	26.96 (21.23 to 33.64)	10.37% (-16.87% to 45.65%)
**Mississippi**	368.49 (303.64 to 447.71)	23.79 (19.77 to 29.1)	18.71% (-5% to 48.33%)	365.95 (295.98 to 451.55)	23.16 (18.55 to 28.24)	18.33% (-7.58% to 50.55%)
**Missouri**	902.79 (733.32 to 1109.2)	27.65 (22.46 to 33.73)	14.78% (-8.94% to 45.46%)	741 (596.58 to 890.92)	22.11 (17.74 to 26.66)	12.64% (-13.52% to 42.73%)
**Montana**	160.47 (128.26 to 199.65)	26.59 (21.52 to 33.01)	16.84% (-8.24% to 47.53%)	136.83 (114.61 to 164.13)	24.62 (20.48 to 29.72)	23.52% (-2.58% to 57.25%)
**Nebraska**	326.7 (259.52 to 401.56)	30.97 (24.87 to 37.82)	11.22% (-14.73% to 41.02%)	301.13 (249 to 360.09)	28.83 (23.74 to 35.04)	13.4% (-11.31% to 43.21%)
**Nevada**	375.44 (303.82 to 447.73)	22.11 (18.03 to 26.47)	10.16% (-12.44% to 36.74%)	288.51 (239.87 to 345.02)	17.12 (13.98 to 20.69)	8.22% (-14.62% to 34.87%)
**New Hampshire**	232.46 (180.3 to 292.47)	29.71 (23.56 to 37.78)	16.75% (-10.44% to 51.3%)	173.38 (136.55 to 211.71)	21.65 (16.84 to 26.39)	18.59% (-9.41% to 51.08%)
**New Jersey**	2256.13 (1767 to 2858.12)	42.2 (33.33 to 52.41)	9.56% (-16.18% to 41.21%)	1919.77 (1459.83 to 2390.16)	32.13 (25.09 to 40.07)	0.47% (-23.69% to 30.85%)
**New Mexico**	297.96 (238.32 to 364.87)	26.01 (21.01 to 31.52)	26.66% (-1.61% to 60.52%)	224.75 (178.35 to 282.27)	19.33 (15.27 to 24.29)	29.34% (0.93% to 70.99%)
**New York**	2444.46 (1966.06 to 3004.2)	22.96 (18.42 to 27.84)	16.58% (-8.48% to 44.93%)	2024.16 (1608.7 to 2495.1)	18.08 (14.37 to 22.21)	11.92% (-13.16% to 44.36%)
**North Carolina**	1309.56 (1057.29 to 1607.03)	23.57 (19.37 to 28.84)	16.58% (-8.19% to 46.06%)	1152.66 (912.42 to 1409.54)	20.18 (16.16 to 24.89)	9.71% (-16.33% to 42.1%)
**North Dakota**	122.05 (100.51 to 147.74)	29.45 (24.11 to 35.96)	14.35% (-9.54% to 45.03%)	103.77 (86.9 to 127.14)	26.74 (22.09 to 33.07)	14.62% (-11.08% to 46.63%)
**Ohio**	1600.23 (1287.34 to 1932.98)	25.15 (20.51 to 30.29)	8.06% (-13.48% to 33.61%)	1348.04 (1076.61 to 1633.44)	20.69 (16.82 to 25.13)	4.7% (-18.13% to 32.53%)
**Oklahoma**	485.34 (399.46 to 579.32)	22.92 (18.83 to 27.23)	-1.33% (-21.09% to 23.98%)	425.66 (340.86 to 524.71)	20.12 (15.92 to 24.76)	2.4% (-21.1% to 30.93%)
**Oregon**	741.85 (591.42 to 907.09)	31.22 (25.11 to 37.78)	8.07% (-15.55% to 37.44%)	561.4 (449.5 to 692.52)	23.76 (19.22 to 29.28)	-1.64% (-23.42% to 26.64%)
**Pennsylvania**	1886.23 (1506.07 to 2320.33)	27.13 (22.05 to 33.15)	12.59% (-10.13% to 41.41%)	1474.74 (1189.39 to 1804.88)	20.58 (16.85 to 25)	4.74% (-18.32% to 34.52%)
**Rhode Island**	155.05 (119.18 to 199.69)	25.66 (19.78 to 33.28)	2.87% (-22.1% to 35.46%)	131.98 (104.43 to 162.37)	21.48 (17.17 to 26.19)	3.18% (-20.98% to 33.54%)
**South Carolina**	694.5 (566.3 to 842.09)	25.12 (20.38 to 30.19)	24.63% (1.98% to 55.59%)	601.61 (477.32 to 734.4)	21.22 (16.87 to 26.13)	21.36% (-6.58% to 54.46%)
**South Dakota**	144.83 (119.6 to 173.91)	30.99 (25.25 to 37.05)	19.14% (-5.69% to 50.77%)	114.36 (95.3 to 134.01)	25.1 (20.55 to 29.85)	2.7% (-19.07% to 30.11%)
**Tennessee**	974.06 (794.18 to 1175.35)	26.55 (22.02 to 31.72)	13.63% (-8.11% to 38.84%)	840.48 (657.71 to 1039.86)	22.01 (17.33 to 27.35)	17.19% (-10.17% to 51.29%)

© 2025 GBD 2021 US CNS Cancer Collaborators. *JAMA Neurology*.

**Texas**	3397.02<br>(2798.67 to 4080.18)	21.96<br>(18.2 to 25.88)	-1.12%<br>(-20.12% to 21.57%)	3066.99<br>(2446.7 to 3761.45)	19.74<br>(15.91 to 24.3)	1.31%<br>(-20.12% to 27.88%)
**Utah**	374.41<br>(301.64 to 461.71)	21.85<br>(17.69 to 26.85)	2.38%<br>(-20.7% to 28.01%)	389.26<br>(323.36 to 469.96)	22.13<br>(18.3 to 26.47)	10.69%<br>(-11.33% to 40.01%)
**Vermont**	102.69<br>(85.25 to 123.36)	28.68<br>(23.93 to 34.8)	34.31%<br>(7.25% to 65.63%)	78.87<br>(64.61 to 93.61)	22.94<br>(18.26 to 27.87)	27.74%<br>(-2.5% to 61.83%)
**Virginia**	1185.19<br>(954.4 to 1489.77)	25.7<br>(20.79 to 32.25)	17.55%<br>(-6.08% to 49.7%)	1010.25<br>(786.34 to 1269.61)	21.77<br>(17.1 to 27.11)	19.24%<br>(-8.48% to 56.12%)
**Washington**	853.27<br>(687.19 to 1028.4)	19.84<br>(16.17 to 23.77)	-5.91%<br>(-25.77% to 15.48%)	905.32<br>(726.25 to 1123.52)	21.21<br>(17.18 to 26.15)	15.79%<br>(-8.72% to 49.71%)
**West Virginia**	245.55<br>(199.21 to 294.52)	24.64<br>(20.34 to 29.77)	29.53%<br>(3.06% to 59.96%)	210.45<br>(165.38 to 261.64)	21.83<br>(17.04 to 27.19)	29.39%<br>(-4.33% to 68.23%)
**Wisconsin**	930.98<br>(732.54 to 1136.17)	28.91<br>(23.34 to 35.25)	3.82%<br>(-19.2% to 31.35%)	824.76<br>(650.88 to 1014.24)	26.41<br>(20.82 to 32.84)	6.66%<br>(-19.83% to 41.16%)
**Wyoming**	84.85<br>(70.08 to 101.23)	25.94<br>(21.51 to 31.07)	13.86%<br>(-11.42% to 39.47%)	61.51<br>(50.7 to 72.58)	20.4<br>(16.79 to 24.4)	1.96%<br>(-19.16% to 29.3%)

© 2025 GBD 2021 US CNS Cancer Collaborators. *JAMA Neurology*.

eTable 3e: YLD count and age-standardized rate of Brain and central nervous system cancer by state and sex, 2021, and percentage change from 1990 to 2021, United States

	Male			Female		
	Absolute number, 2021	Age-standardized rate, per 100 000 people, 2021	Percentage Change, 1990-2021	Absolute number, 2021	Age-standardized rate, per 100 000 people, 2021	Percentage Change, 1990-2021
**United States of America**	6059.23 (4397.19 to 7872.68)	2.95 (2.17 to 3.89)	2.25% (-3.3% to 8.51%)	4950.09 (3597.8 to 6426.81)	2.27 (1.68 to 2.97)	1.15% (-3.95% to 6.62%)
**Alabama**	99.33 (65.49 to 139.98)	3.2 (2.18 to 4.44)	8.14% (-18.81% to 39.06%)	82.85 (57.77 to 113.77)	2.45 (1.72 to 3.39)	7.33% (-18.55% to 36.84%)
**Alaska**	14.04 (9.72 to 21.52)	3.36 (2.22 to 5.65)	32.33% (-5.71% to 103.34%)	9.53 (6.57 to 13.28)	2.5 (1.71 to 3.56)	33.82% (1.88% to 72.82%)
**Arizona**	135.37 (90.68 to 185.47)	3.07 (2.05 to 4.21)	13.1% (-12.12% to 50.15%)	105.21 (71.55 to 146.99)	2.28 (1.53 to 3.19)	8.67% (-17.7% to 41.52%)
**Arkansas**	57.54 (38.06 to 78.68)	3.13 (2.08 to 4.2)	6.28% (-18.04% to 43.38%)	48.83 (31.29 to 66.72)	2.54 (1.66 to 3.48)	0.38% (-25.23% to 29.15%)
**California**	670.71 (460.12 to 906.9)	2.79 (1.93 to 3.75)	-2.57% (-25.93% to 26.2%)	509.84 (345.29 to 692.52)	2.1 (1.43 to 2.89)	-4.56% (-25.76% to 20.99%)
**Colorado**	109.6 (72.37 to 149.63)	3.11 (2.09 to 4.26)	5.23% (-20.27% to 38.43%)	86.55 (56.16 to 120.38)	2.29 (1.51 to 3.25)	1.88% (-23.09% to 34.46%)
**Connecticut**	93.81 (63.57 to 138.8)	3.97 (2.66 to 5.76)	-1.23% (-27.59% to 30.07%)	75.58 (49.99 to 104.65)	2.9 (1.94 to 4.07)	-4.11% (-27.12% to 27.13%)
**Delaware**	18.53 (12.95 to 25.29)	3 (2.09 to 4.13)	17.38% (-8.49% to 50.6%)	13.99 (9.65 to 18.87)	2.03 (1.38 to 2.78)	0.15% (-20.33% to 28.13%)
**District of Columbia**	6.82 (4.54 to 9.34)	1.8 (1.22 to 2.48)	-22.4% (-40.77% to 0.13%)	5.53 (3.79 to 7.34)	1.36 (0.94 to 1.88)	-8.13% (-27.88% to 13.09%)
**Florida**	357.03 (231.73 to 511.31)	2.66 (1.76 to 3.85)	1.16% (-23.06% to 32.47%)	291.58 (192.07 to 392.69)	2.05 (1.36 to 2.85)	0.74% (-24.05% to 29.85%)
**Georgia**	174.08 (116.47 to 248.22)	2.71 (1.81 to 3.79)	6.5% (-17.88% to 39.02%)	148.16 (98.16 to 207.3)	2.14 (1.42 to 3.02)	2.12% (-19.92% to 29.07%)
**Hawaii**	19.34 (13.13 to 27.17)	2.06 (1.44 to 2.95)	7.77% (-18.78% to 40.16%)	15.26 (10.56 to 20.47)	1.58 (1.08 to 2.17)	-2.38% (-25.82% to 26.86%)
**Idaho**	37.85 (24.97 to 54.06)	3.32 (2.23 to 4.63)	7.5% (-22.16% to 43.55%)	25.91 (18.15 to 35.28)	2.17 (1.49 to 2.94)	-1.72% (-23.93% to 25.86%)
**Illinois**	228.12 (146.93 to 327.59)	2.94 (1.97 to 4.18)	7.35% (-18.27% to 39.67%)	178.72 (122.47 to 245.91)	2.15 (1.48 to 2.96)	4.55% (-18.39% to 32.53%)
**Indiana**	123.96 (81.84 to 175.39)	2.98 (2.02 to 4.16)	1.6% (-22.52% to 31.29%)	105.55 (71.66 to 142.39)	2.45 (1.64 to 3.4)	5.71% (-20.86% to 37.64%)
**Iowa**	61.69 (38.97 to 91.19)	2.97 (1.96 to 4.39)	14.17% (-17.27% to 50.44%)	54.3 (37.47 to 76.38)	2.58 (1.78 to 3.63)	5.37% (-21.91% to 40.84%)
**Kansas**	62 (42.18 to 84.75)	3.47 (2.35 to 4.73)	6.86% (-19.82% to 41.92%)	49.59 (33.55 to 70.73)	2.67 (1.79 to 3.8)	9.51% (-17.19% to 41.85%)
**Kentucky**	104 (69.21 to 142.15)	3.71 (2.49 to 4.97)	2.26% (-22.76% to 32.03%)	86.78 (57.68 to 125.27)	2.9 (1.96 to 4.15)	6.41% (-20.22% to 42.63%)
**Louisiana**	87.05 (56.95 to 122.94)	3.11 (2.05 to 4.32)	9.03% (-18.21% to 42.79%)	69.49 (47.76 to 97.77)	2.39 (1.64 to 3.42)	14.05% (-12.56% to 50.76%)

© 2025 GBD 2021 US CNS Cancer Collaborators. *JAMA Neurology*.

**Maine**	32.97 (21.45 to 47.53)	3.55 (2.37 to 5.08)	16.84% (-15.52% to 54.7%)	23.47 (15.88 to 32.87)	2.29 (1.58 to 3.2)	0.29% (-24.9% to 30.61%)
**Maryland**	99.15 (66.25 to 141.63)	2.68 (1.8 to 3.85)	-3.59% (-27.96% to 24.5%)	81.13 (55.21 to 114.7)	2.01 (1.35 to 2.85)	-2.85% (-27.56% to 30.26%)
**Massachusetts**	148.87 (93.47 to 218.17)	3.24 (2.11 to 4.66)	0.86% (-25.65% to 35.16%)	119.12 (77.33 to 169.49)	2.33 (1.56 to 3.36)	-7.45% (-29.88% to 19.79%)
**Michigan**	181.44 (119.7 to 260.42)	2.75 (1.88 to 3.94)	0.6% (-24.89% to 30.46%)	180.34 (122.35 to 257.25)	2.5 (1.71 to 3.57)	3.11% (-20.64% to 33.84%)
**Minnesota**	127.66 (82.69 to 179.68)	3.62 (2.36 to 5.15)	4.36% (-22.76% to 37.93%)	98.93 (65.66 to 138.19)	2.69 (1.78 to 3.8)	3.47% (-22.82% to 35.33%)
**Mississippi**	53.76 (33.6 to 76.7)	3.04 (1.94 to 4.25)	14.27% (-15.98% to 52.34%)	50.91 (34.04 to 69.21)	2.69 (1.8 to 3.65)	14.76% (-11.12% to 47.23%)
**Missouri**	122.1 (83.25 to 171.55)	3.22 (2.24 to 4.45)	8.41% (-18.4% to 39.68%)	97.4 (67.77 to 132.46)	2.39 (1.66 to 3.26)	7.3% (-17.82% to 39.19%)
**Montana**	22.19 (14.82 to 31.55)	3.08 (2.09 to 4.3)	13.19% (-14.02% to 50.76%)	17.68 (12.22 to 24.15)	2.56 (1.75 to 3.47)	14.75% (-11.23% to 46.98%)
**Nebraska**	41.9 (28.07 to 58.73)	3.51 (2.33 to 4.85)	7.05% (-20.24% to 43.53%)	36.56 (24.86 to 50.25)	2.97 (2.07 to 4.09)	10.69% (-14.18% to 43.22%)
**Nevada**	51.59 (33.8 to 70.64)	2.65 (1.77 to 3.61)	3.62% (-20.71% to 35.37%)	38.32 (26.19 to 51.33)	1.92 (1.31 to 2.61)	2.06% (-18.97% to 30.13%)
**New Hampshire**	31.51 (19.53 to 44.45)	3.38 (2.13 to 4.77)	10.76% (-21.28% to 44.31%)	22.74 (14.86 to 30.74)	2.28 (1.5 to 3.1)	9.72% (-17.3% to 39.88%)
**New Jersey**	246.77 (165.02 to 342.59)	4.2 (2.82 to 5.8)	4.66% (-21.97% to 37.36%)	203.34 (134.68 to 292.32)	3.02 (2.03 to 4.34)	-2.92% (-27.3% to 28.97%)
**New Mexico**	38.6 (25.47 to 53.39)	2.96 (1.93 to 4.07)	23.1% (-6.92% to 58.83%)	29.43 (19.64 to 41.46)	2.11 (1.42 to 3.01)	25.09% (-3.96% to 61.95%)
**New York**	317.11 (218.42 to 439.28)	2.58 (1.78 to 3.58)	2.25% (-20.57% to 33%)	264.15 (177.79 to 367.5)	1.92 (1.28 to 2.65)	3.86% (-20.73% to 33.14%)
**North Carolina**	180.35 (119.98 to 263.06)	2.82 (1.89 to 3.98)	5.98% (-19.62% to 38.1%)	150.92 (101.46 to 211.69)	2.18 (1.47 to 3.07)	-0.32% (-23.07% to 29.01%)
**North Dakota**	15.31 (9.87 to 20.92)	3.23 (2.08 to 4.45)	6.18% (-20.65% to 39.7%)	12.28 (8.52 to 16.8)	2.67 (1.83 to 3.62)	4.59% (-17.46% to 36.16%)
**Ohio**	224.66 (149.31 to 315.56)	3.03 (2.09 to 4.25)	5.09% (-21.57% to 36.71%)	182.65 (124.29 to 253.34)	2.28 (1.58 to 3.18)	3.57% (-19.77% to 32.24%)
**Oklahoma**	67.88 (45.21 to 98.14)	2.82 (1.92 to 3.98)	-1.31% (-24.44% to 30.94%)	58.59 (39.02 to 80.3)	2.32 (1.55 to 3.18)	2.46% (-21.61% to 31.56%)
**Oregon**	97.85 (62.65 to 140.84)	3.52 (2.32 to 5.04)	3.01% (-22.86% to 37.14%)	70.46 (47.5 to 101.08)	2.44 (1.64 to 3.5)	-6.1% (-27.64% to 23.51%)
**Pennsylvania**	255.19 (164.53 to 364.55)	3.1 (2.03 to 4.39)	4.75% (-22.34% to 36.6%)	195.29 (126.81 to 269.74)	2.18 (1.43 to 3.06)	-3.55% (-25.35% to 26.76%)
**Rhode Island**	21.08 (13.52 to 30.87)	2.97 (1.96 to 4.36)	-3.87% (-33.02% to 30.54%)	17.32 (11.54 to 24.75)	2.24 (1.53 to 3.15)	-4.54% (-26.08% to 24.48%)
**South Carolina**	98.14 (66.28 to 139.03)	3.05 (2.09 to 4.29)	11.28% (-16.42% to 42.69%)	82.91 (55.12 to 116.32)	2.39 (1.61 to 3.4)	12.24% (-15.27% to 46.31%)
**South Dakota**	19 (13.09 to 26.43)	3.52 (2.44 to 4.91)	11.82% (-15.13% to 46.83%)	14.43 (9.77 to 18.85)	2.65 (1.79 to 3.45)	2.18% (-19.97% to 32.08%)
**Tennessee**	134.98 (91.04 to 187.37)	3.21 (2.22 to 4.44)	6.25% (-20.58% to 39.29%)	112.87 (77.12 to 156.28)	2.46 (1.66 to 3.49)	10.58% (-16.47% to 43.05%)

© 2025 GBD 2021 US CNS Cancer Collaborators. *JAMA Neurology*.

**Texas**	451.26 (303.51 to 636.91)	2.65 (1.8 to 3.72)	-6.03% (-26.75% to 19.68%)	380.73 (260.16 to 510.55)	2.13 (1.46 to 2.92)	-5.8% (-27.41% to 21.37%)
**Utah**	49.12 (33.16 to 70.11)	2.74 (1.86 to 3.89)	2.32% (-22.63% to 33.02%)	42.97 (29.2 to 60.26)	2.3 (1.59 to 3.2)	6.05% (-18.7% to 35.34%)
**Vermont**	13.95 (9.29 to 18.89)	3.25 (2.19 to 4.47)	19.57% (-8.53% to 54.87%)	10.18 (7.09 to 14.14)	2.34 (1.63 to 3.25)	13.78% (-13.4% to 49.61%)
**Virginia**	155.76 (105.12 to 216.94)	2.96 (1.97 to 4.04)	6.77% (-17.87% to 38.29%)	126.63 (86.72 to 173.85)	2.27 (1.55 to 3.18)	9.14% (-16.3% to 43.93%)
**Washington**	127.19 (81.72 to 176.43)	2.56 (1.65 to 3.58)	-11.96% (-34.41% to 14.87%)	124.6 (82.92 to 179.06)	2.43 (1.61 to 3.39)	4.37% (-20.13% to 35.7%)
**West Virginia**	36.62 (23.63 to 50.21)	3.09 (2.05 to 4.2)	24.54% (-6.88% to 59.41%)	30.07 (20.74 to 41.71)	2.47 (1.74 to 3.42)	24.96% (-3.28% to 62.52%)
**Wisconsin**	122.87 (80.6 to 173.17)	3.27 (2.15 to 4.58)	0.27% (-25.43% to 30.81%)	102.41 (68.64 to 142.07)	2.69 (1.79 to 3.78)	2.07% (-22.11% to 33.5%)
**Wyoming**	11.51 (7.59 to 15.88)	3.06 (2.07 to 4.17)	11.09% (-14.04% to 40.96%)	8 (5.54 to 10.93)	2.22 (1.54 to 3.01)	0.57% (-20.97% to 27.17%)

© 2025 GBD 2021 US CNS Cancer Collaborators. *JAMA Neurology*.

eTable 3f: YLL count and age-standardized rate of Brain and central nervous system cancer by state and sex, 2021, and percentage change from 1990 to 2021, United States

	Male			Female		
	Absolute number, 2021	Age-standardized rate, per 100 000 people, 2021	Percentage Change, 1990-2021	Absolute number, 2021	Age-standardized rate, per 100 000 people, 2021	Percentage Change, 1990-2021
**United States of America**	338069.81 (325335.24 to 347969.68)	157.74 (152.17 to 162.34)	-16.19% (-18.88% to -13.52%)	245917.02 (232317.57 to 254689.71)	107.62 (103.35 to 111.17)	-16.61% (-18.9% to -14.57%)
**Alabama**	6757.59 (5454.89 to 8142.28)	210.51 (172.15 to 250.9)	-3.76% (-22.79% to 16.01%)	5161.43 (4208.99 to 6188.6)	146.82 (120.93 to 174.43)	-1.85% (-20.34% to 17.45%)
**Alaska**	758.19 (614.13 to 945.38)	171.38 (135.25 to 226.99)	-0.67% (-23.09% to 30.99%)	454.65 (402.28 to 520.21)	112.92 (100.42 to 128.32)	-0.01% (-12.67% to 15.04%)
**Arizona**	7254.68 (5907.8 to 8771.46)	158.04 (130.64 to 188.57)	-7.35% (-23.07% to 12.12%)	5105.98 (4175.82 to 6130.74)	104.61 (86.42 to 124.49)	-11.39% (-26.67% to 6.16%)
**Arkansas**	3792.22 (3021.01 to 4594.98)	200.63 (162.62 to 241.3)	-4.17% (-22.04% to 16.82%)	2954.07 (2420.98 to 3525.04)	147.94 (122.23 to 176.05)	-8.09% (-24.94% to 10.76%)
**California**	35568.23 (28779.11 to 43203.01)	140.57 (114.77 to 170.47)	-21.5% (-35.96% to -5.82%)	24565.45 (20293.82 to 29469.98)	94.45 (79.35 to 111.85)	-23.8% (-35.41% to -9.48%)
**Colorado**	5663.04 (4447.21 to 7016.83)	152.53 (122.72 to 187.26)	-15.95% (-32.4% to 2.83%)	4040.99 (3250.49 to 4860.65)	100.41 (81.47 to 120.87)	-17.68% (-33.68% to -0.1%)
**Connecticut**	3530.89 (2786.89 to 4469.38)	145.08 (116.02 to 182.17)	-19.03% (-36.55% to 0.87%)	2604.74 (2105.36 to 3205.26)	98.47 (80.36 to 119.91)	-18.29% (-33.94% to -0.21%)
**Delaware**	1035.74 (875.3 to 1233.49)	160.99 (136.4 to 189.95)	-7.62% (-23.67% to 10.63%)	704.61 (613.81 to 797.35)	97.08 (85.18 to 109.53)	-21.51% (-32.12% to -9.8%)
**District of Columbia**	453.1 (355.79 to 562.82)	117.11 (92.71 to 143.82)	-47.47% (-59.46% to -34.21%)	338.24 (287.25 to 402.8)	83.25 (71.67 to 97.35)	-38.36% (-47.54% to -26.7%)
**Florida**	23525.99 (18930.99 to 28609.27)	159.78 (130.9 to 191.64)	-16.21% (-32.22% to 2.22%)	16850.5 (13939.37 to 20337.97)	107.27 (90.54 to 128.24)	-18.26% (-31.52% to -3.01%)
**Georgia**	10088.79 (8206.59 to 12344.43)	154.73 (127.78 to 187.28)	-18.75% (-34.29% to -1.58%)	8127.28 (6646.9 to 9779.08)	111.93 (93.11 to 132.64)	-18.2% (-32.22% to -2.12%)
**Hawaii**	1042.51 (825.42 to 1281.77)	106.36 (85.5 to 130.85)	-9.69% (-28.37% to 12.53%)	688.59 (581.07 to 803.16)	68.17 (58.36 to 78.45)	-21.99% (-33.04% to -8.89%)
**Idaho**	2076.22 (1659.61 to 2575.73)	175.35 (141.55 to 214.51)	-10.42% (-28.7% to 11.67%)	1292.34 (1093.49 to 1501.16)	103.73 (88.42 to 120.16)	-15.47% (-28.19% to -1.27%)
**Illinois**	12413.61 (9941.6 to 15382.61)	152.64 (123.49 to 186.49)	-17.69% (-33.6% to -0.01%)	8575.41 (7008.02 to 10307.87)	98.28 (81.68 to 116.77)	-21.72% (-35.32% to -6.68%)
**Indiana**	7391.9 (6008.36 to 9005.33)	170.2 (139.74 to 205.16)	-10.41% (-27.08% to 9.16%)	5656.19 (4615.93 to 6882.36)	125.35 (103.25 to 150.98)	-4.66% (-21.74% to 13.25%)
**Iowa**	3750.86 (2987.15 to 4589.14)	177.27 (142.46 to 214.82)	-4.29% (-23.86% to 19.37%)	2603.91 (2118.76 to 3156.67)	121.81 (98.88 to 145.84)	-3.05% (-21.75% to 18.46%)
**Kansas**	3438.01 (2703.34 to 4244.68)	184.69 (146.84 to 226.28)	-4.56% (-23.8% to 16.79%)	2515.53 (2047.25 to 3042.32)	128.95 (106.74 to 155.65)	0.99% (-18.2% to 22.78%)
**Kentucky**	5481.09 (4381.98 to 6693.67)	186.2 (150.45 to 225.12)	-5.05% (-23.51% to 16.15%)	4262.86 (3501.54 to 5258)	139.73 (116.39 to 171.25)	-1.2% (-18.78% to 20.38%)
**Louisiana**	5415.43 (4394.28 to 6597.28)	187.42 (152.85 to 227.09)	-2.67% (-21.49% to 17.3%)	3796.73 (3151.97 to 4560.26)	124.89 (105.39 to 149.11)	-3.96% (-19.96% to 14.09%)
**Maine**	1840.39 (1437.41 to 2304.49)	186.77 (149.14 to 231.19)	2.5% (-19.59% to 27.07%)	1177.17 (970.02 to 1388.91)	107.17 (88.93 to 127.02)	-10.92% (-26.78% to 5.54%)

© 2025 GBD 2021 US CNS Cancer Collaborators. *JAMA Neurology*.

**Maryland**	5599.7 (4371.43 to 6861.66)	144.93 (115.21 to 176.1)	-24.76% (-40.57% to -7.63%)	4145.93 (3359.71 to 5099.11)	97.58 (80.37 to 118.45)	-23.06% (-37.81% to -5.18%)
**Massachusetts**	7124.16 (5497.91 to 9285.6)	147.19 (115.54 to 189.93)	-21.73% (-38.53% to 0.99%)	5006.98 (3991.02 to 6070.31)	92.87 (75.39 to 111.88)	-27.61% (-41.15% to -12.09%)
**Michigan**	11025.75 (8898.7 to 13222.8)	167.17 (137.04 to 199.3)	-15.94% (-32.68% to 0.99%)	8557.96 (6901.79 to 10379.16)	118.24 (97.43 to 141.86)	-15.07% (-29.7% to 1.66%)
**Minnesota**	5984.23 (4681.72 to 7400.95)	159.34 (127.98 to 195.33)	-16.25% (-33.64% to 1.82%)	4173.85 (3374.54 to 5075.94)	107.14 (87.87 to 129.63)	-14.51% (-30.6% to 4%)
**Mississippi**	3810.26 (3088.02 to 4637.03)	209.83 (172.79 to 252.65)	3.67% (-16.37% to 27.24%)	3304.33 (2725.53 to 3949.46)	168.81 (141.05 to 200.39)	5.13% (-11.61% to 26.38%)
**Missouri**	7160.72 (5774.03 to 8762.61)	180.6 (146.75 to 217.54)	-6.62% (-25.21% to 13.59%)	5179.51 (4156.81 to 6222.62)	120.53 (97.94 to 144.66)	-7.35% (-24.09% to 10.88%)
**Montana**	1271.13 (1003.35 to 1586.19)	169.2 (135.82 to 208.05)	-1.15% (-22.7% to 23.62%)	910.64 (791.09 to 1048.44)	124.01 (109.28 to 141.44)	-2.87% (-16.47% to 12.67%)
**Nebraska**	2197.42 (1716.95 to 2694.56)	175.88 (139.5 to 214.19)	-9.43% (-29.72% to 13.29%)	1734.53 (1459.48 to 2020.53)	134.46 (115.21 to 155.16)	-5.78% (-19.68% to 10.16%)
**Nevada**	2961.85 (2368.94 to 3616.07)	146.07 (118.64 to 176.77)	-16.56% (-32.71% to 1.9%)	1966.21 (1695.32 to 2278.95)	94.54 (82.41 to 109.47)	-18.29% (-29.1% to -5.01%)
**New Hampshire**	1618.27 (1262.6 to 2051.49)	161.6 (127.32 to 201.96)	-11.2% (-31.32% to 12.87%)	1048.69 (870.51 to 1252.55)	96.52 (81.61 to 113.67)	-13.45% (-27.63% to 2.78%)
**New Jersey**	8468.37 (6615.5 to 10467.58)	136.72 (107.95 to 167.36)	-22.89% (-39.53% to -4.32%)	6199.14 (4927.72 to 7523.33)	89.92 (72.29 to 108.65)	-24.88% (-39.48% to -7.76%)
**New Mexico**	2230.8 (1764.84 to 2728.74)	166.88 (134.38 to 203.96)	7.34% (-15% to 31.41%)	1552.38 (1268.53 to 1887.9)	109.3 (90.08 to 132.93)	3.33% (-15.9% to 27.38%)
**New York**	16520.89 (13173.97 to 20210.8)	127.83 (103.52 to 154.75)	-26.68% (-40.55% to -10.27%)	12063.63 (9834.42 to 14444.01)	83.9 (69.77 to 98.76)	-26.43% (-38.78% to -12.57%)
**North Carolina**	10681.8 (8538.1 to 13042.93)	159.84 (129.32 to 192.82)	-15.62% (-31.73% to 4.49%)	8056.17 (6519.48 to 9582.71)	110.47 (91.34 to 130.7)	-20.6% (-34.6% to -5.2%)
**North Dakota**	828.41 (679.85 to 998.14)	168.4 (139.4 to 200.86)	-9.46% (-26.2% to 9.15%)	592.59 (512.07 to 695.89)	123.82 (107.86 to 143.6)	-9.17% (-22.22% to 4.85%)
**Ohio**	13554.96 (10840.12 to 16570.12)	174.65 (141.92 to 211.17)	-8.06% (-26.43% to 12.03%)	9976.55 (8322.24 to 11797.16)	118.44 (99.14 to 139.51)	-6.35% (-22.44% to 10.87%)
**Oklahoma**	4407.44 (3596.89 to 5376.55)	178.12 (146.47 to 215.23)	-5.04% (-22.15% to 15.15%)	3452.86 (2879.32 to 4166.91)	133.24 (112.32 to 159.36)	0.4% (-16.92% to 20.6%)
**Oregon**	5000.15 (4007.91 to 6231.7)	170.24 (137.85 to 209.49)	-17.53% (-33.71% to 1.52%)	3202.19 (2579.39 to 3963.75)	104.24 (85.2 to 126.92)	-24.75% (-38.58% to -8.2%)
**Pennsylvania**	14211.5 (11335.03 to 17386.41)	163.74 (132.85 to 198.86)	-16.05% (-32.34% to 2.88%)	9703.99 (7834.1 to 11528.75)	102.4 (84.88 to 121.44)	-21.24% (-34.58% to -6.67%)
**Rhode Island**	1098.8 (851.99 to 1368.27)	146.94 (114.7 to 183.42)	-24.91% (-42.92% to -3.22%)	805.49 (663.77 to 968.81)	98.84 (82.51 to 117.43)	-26.24% (-39.2% to -12.06%)
**South Carolina**	6178.72 (4937.16 to 7570.29)	185.51 (149.82 to 225.18)	-13.04% (-29.44% to 5.31%)	4749.86 (3891.02 to 5726.62)	130.86 (108.16 to 155.71)	-10.74% (-26.24% to 7.97%)
**South Dakota**	1090.55 (900.64 to 1323.95)	194.51 (162.4 to 233.58)	-2.1% (-18.9% to 20.34%)	758.98 (650.82 to 879.7)	132.86 (114.83 to 152.83)	-8.73% (-21.95% to 6.89%)
**Tennessee**	8573.53 (6883.73 to 10587.66)	195.35 (159.04 to 238.16)	-10.6% (-27.29% to 9.05%)	6401.3 (5289.74 to 7745.85)	132.64 (109.24 to 160.03)	-5.91% (-23.04% to 14.68%)
**Texas**	25531.71 (21061.82 to 30522.31)	144.41 (120.37 to 171.46)	-24.3% (-37.31% to -10%)	19433.49 (16149.98 to 22878.6)	104.21 (87.61 to 121.88)	-23.63% (-35.72% to -11%)

© 2025 GBD 2021 US CNS Cancer Collaborators. *JAMA Neurology*.

**Utah**	2724.72 (2194.41 to 3330.69)	149.56 (121.5 to 181.94)	-13.93% (-30.58% to 6.48%)	1926.04 (1666.62 to 2242.25)	102.24 (89.26 to 118.46)	-14.19% (-26.45% to 1.17%)
**Vermont**	743.13 (613.05 to 894.65)	162.79 (135.65 to 194.54)	-9.3% (-25.72% to 9.57%)	481.05 (415.19 to 558.37)	101.97 (87.96 to 116.7)	-14.41% (-26.88% to -1.07%)
**Virginia**	8243.75 (6538.82 to 10185.92)	148.97 (120.78 to 182.2)	-17.56% (-33.49% to 1.14%)	6046.57 (4944.14 to 7313.79)	102.18 (84.8 to 122.05)	-14.63% (-29.74% to 3.47%)
**Washington**	8366.34 (6718.62 to 10113.37)	163.1 (132.02 to 194.61)	-21.54% (-37.03% to -6.19%)	5970.14 (4877.32 to 7359.05)	111.26 (92.42 to 135.73)	-18% (-31.87% to 0.63%)
**West Virginia**	2429.62 (1945.68 to 2979.08)	197.49 (160.73 to 239.66)	15.18% (-7.15% to 39.47%)	1789.04 (1458.02 to 2160.76)	140.56 (114.82 to 170.3)	14.12% (-6.94% to 39.49%)
**Wisconsin**	6494.99 (5079.67 to 8074.08)	163.17 (129.68 to 198.63)	-13.48% (-31.67% to 6.89%)	4833.55 (3909.64 to 5811.51)	119.4 (97.87 to 143.22)	-10.45% (-26.58% to 9.19%)
**Wyoming**	657.65 (555.11 to 786.01)	167.57 (142 to 199.97)	-5.46% (-21.38% to 11.98%)	416.68 (359.55 to 476.64)	110 (96.49 to 126.82)	-13.31% (-24.48% to -0.11%)

© 2025 GBD 2021 US CNS Cancer Collaborators. *JAMA Neurology*.

eTable 4a: Incidence, DALYs rate of Brain and central nervous system cancer by age and sex, 1990-2021, United States

	Incidence (95% UI)						DALYs (95% UI)					
	1990		2021		Percentage Change, 1990-2021		1990		2021		Percentage Change, 1990-2021	
	Male	Female	Male	Female	Male	Female	Male	Female	Male	Female	Male	Female
**<5 years**	3.49 (3.17 to 3.84)	3.43 (3.08 to 3.87)	2.88 (2.41 to 3.34)	2.39 (2.02 to 2.8)	-17.39% (-32.11% to -2.07%)	-30.52% (-42.87% to -14.46%)	85.36 (82.16 to 88.39)	80.58 (77.84 to 83.46)	54.76 (46.96 to 62.79)	45.22 (40.26 to 50.71)	-35.86% (-45.1% to -26.75%)	-43.89% (-50.16% to -36.76%)
**5-9 years**	3.69 (3.4 to 4.01)	3.77 (3.44 to 4.13)	3.38 (3.01 to 3.83)	3.3 (2.89 to 3.71)	-8.42% (-21.53% to 6.57%)	-12.53% (-26.4% to 2.47%)	106.84 (103.09 to 111.25)	106.56 (102.66 to 110.03)	78.64 (71.57 to 85.95)	76.21 (71.83 to 81.28)	-26.4% (-33.21% to -17.56%)	-28.48% (-33.24% to -22.73%)
**10-14 years**	3.19 (2.94 to 3.51)	2.93 (2.64 to 3.24)	3.27 (2.93 to 3.67)	3.17 (2.81 to 3.59)	2.46% (-11.2% to 17.1%)	8.4% (-7.97% to 27.51%)	82.23 (79.5 to 85.71)	70.26 (67.58 to 73.12)	67.09 (62.4 to 72.25)	61.58 (58.05 to 65.24)	-18.41% (-24.72% to -11.51%)	-12.36% (-17.92% to -6.34%)
**15-19 years**	2.49 (2.28 to 2.74)	2.19 (1.98 to 2.43)	2.65 (2.38 to 2.96)	2.22 (1.92 to 2.57)	6.47% (-8.37% to 23.84%)	1.48% (-14.92% to 22.62%)	63.06 (60.58 to 65.45)	47.56 (45.87 to 49.12)	53.23 (49.63 to 56.79)	39.2 (36.45 to 42.65)	-15.58% (-22.58% to -8.17%)	-17.56% (-23.91% to -8.69%)
**20-24 years**	2.68 (2.45 to 2.94)	2.51 (2.26 to 2.83)	2.86 (2.6 to 3.16)	2.61 (2.27 to 3.01)	6.73% (-6.28% to 21.45%)	4.08% (-12.34% to 21.94%)	61.74 (59.17 to 64.54)	46.75 (44.89 to 48.87)	52.32 (49.61 to 55.02)	38.97 (36.82 to 41.1)	-15.26% (-20.36% to -9.67%)	-16.65% (-21.66% to -11.29%)
**25-29 years**	3.65 (3.37 to 4)	3.24 (2.91 to 3.57)	3.69 (3.36 to 4.08)	3.25 (2.87 to 3.69)	1.02% (-11.05% to 16.18%)	0.25% (-13.77% to 17.71%)	84.96 (81.93 to 88.54)	59.97 (57.71 to 62.32)	69.35 (65.52 to 73.29)	49.05 (46.39 to 51.81)	-18.37% (-24.08% to -12.5%)	-18.2% (-22.81% to -13.12%)
**30-34 years**	4.9 (4.57 to 5.24)	4.13 (3.74 to 4.54)	4.71 (4.33 to 5.16)	3.78 (3.38 to 4.23)	-3.86% (-14.37% to 6.74%)	-8.37% (-20.91% to 6.15%)	121.8 (117.94 to 126.23)	81.27 (78.86 to 83.77)	96.82 (92.43 to 101.77)	61.48 (58.13 to 64.97)	-20.51% (-24.95% to -15.73%)	-24.35% (-28.71% to -19.83%)
**35-39 years**	6.15 (5.84 to 6.51)	4.56 (4.21 to 4.95)	5.5 (5.1 to 5.93)	4.34 (3.94 to 4.81)	-10.59% (-18.4% to -2.38%)	-4.9% (-15.49% to 7.56%)	165.67 (161.12 to 170.44)	100.94 (98.35 to 104.21)	126.38 (120.45 to 132.4)	81.2 (77.09 to 86.56)	-23.72% (-27.81% to -19.12%)	-19.56% (-23.69% to -14.39%)
**40-44 years**	7.66 (7.26 to 8.07)	5.16 (4.81 to 5.54)	6.16 (5.73 to 6.6)	4.63 (4.22 to 5.03)	-19.58% (-25.83% to -13.02%)	-10.24% (-19.15% to -1.4%)	222.7 (214.35 to 230.34)	128.76 (124.54 to 133.25)	158.23 (150.31 to 165.85)	101.11 (96.08 to 106.57)	-28.95% (-32.34% to -25.18%)	-21.48% (-25.46% to -17.3%)
**45-49 years**	9.61 (9.17 to 10.06)	6.57 (6.18 to 6.99)	8.14 (7.61 to 8.63)	5.79 (5.42 to 6.23)	-15.33% (-21.21% to -8.89%)	-11.91% (-19.83% to -4.12%)	278.15 (268.5 to 286.36)	168.66 (163.51 to 174.01)	210.85 (200.95 to 219.83)	131.51 (126.43 to 137.39)	-24.2% (-28.2% to -20.35%)	-22.03% (-25.65% to -18.23%)
**50-54 years**	13.3 (12.73 to 13.87)	8.88 (8.47 to 9.29)	11.07 (10.46 to 11.65)	7.97 (7.54 to 8.45)	-16.74% (-22.58% to -11.29%)	-10.25% (-16.27% to -2.8%)	375.25 (362.08 to 387.28)	228.55 (223.11 to 234.37)	288.42 (276.12 to 298.63)	185.93 (179.34 to 191.9)	-23.14% (-27.07% to -19.3%)	-18.65% (-22.19% to -15.14%)
**55-59 years**	17.65 (16.99 to 18.28)	12.01 (11.48 to 12.47)	16.06 (15.3 to 16.88)	10.94 (10.32 to 11.6)	-9% (-13.92% to -3.14%)	-8.94% (-15.6% to -2.88%)	467.69 (454.71 to 480.23)	301.41 (292.06 to 309.11)	401.99 (387.13 to 416.36)	251.62 (243.06 to 260.21)	-14.05% (-17.67% to -10.03%)	-16.52% (-19.65% to -13.23%)
**60-64 years**	21.5 (20.78 to 22.18)	14.84 (14.08 to 15.53)	19.66 (18.74 to 20.64)	13.78 (12.93 to 14.59)	-8.57% (-13.61% to -3.35%)	-7.16% (-13.33% to -1.25%)	520.24 (504.95 to 534.02)	337.54 (324.48 to 347.78)	453.27 (434.97 to 472.84)	292.61 (278.12 to 304.07)	-12.87% (-16.76% to -8.47%)	-13.31% (-16.92% to -9.52%)
**65-69 years**	26.36 (25.4 to 27.31)	17.79 (16.82 to 18.64)	24.65 (23.41 to 25.84)	17.12 (15.75 to 18.24)	-6.48% (-11.27% to -0.77%)	-3.79% (-9.93% to 2.9%)	552.27 (535.15 to 568.42)	364.69 (346.35 to 377.23)	497.84 (476.02 to 515.7)	328.08 (304.41 to 343.92)	-9.86% (-13.53% to -5.5%)	-10.04% (-14.21% to -5.99%)

© 2025 GBD 2021 US CNS Cancer Collaborators. *JAMA Neurology*.

**70-74 years**	29.09 (27.66 to 30.21)	20.12 (18.57 to 21.11)	30.38 (28.68 to 31.88)	20.82 (18.62 to 22.28)	4.44% (-1.27% to 10.28%)	3.48% (-2.97% to 10.69%)	521.59 (500.82 to 538.11)	356.29 (334.22 to 371.17)	516.96 (491.24 to 539.07)	342.1 (310.44 to 361.34)	-0.89% (-5.36% to 3.81%)	-3.98% (-8.63% to 0.92%)
**75-79 years**	30.13 (28.59 to 31.44)	20.99 (18.88 to 22.34)	34.8 (32.04 to 36.87)	24.14 (20.88 to 26.08)	15.49% (9.22% to 22.86%)	15.03% (7.4% to 22.83%)	452.33 (430.87 to 469.62)	307.46 (278.71 to 323.59)	488.34 (452.62 to 512.13)	328.23 (288.37 to 349.6)	7.96% (2.45% to 13.74%)	6.76% (1.27% to 11.7%)
**80-84 years**	27.62 (25.18 to 29.19)	19.05 (15.66 to 20.87)	37.94 (32.65 to 41.01)	25.64 (20.13 to 28.76)	37.38% (27.44% to 47.05%)	34.6% (25.2% to 45.21%)	339.2 (311.52 to 356.44)	228.9 (190.83 to 248.21)	430.01 (378.25 to 459.36)	282.04 (225.87 to 311.55)	26.77% (19.73% to 34.22%)	23.22% (17.2% to 29.75%)
**85-89 years**	22.94 (19.82 to 24.88)	16.5 (12.74 to 18.48)	41.17 (34.24 to 45.44)	27.41 (20.51 to 31.76)	79.47% (66.21% to 94.26%)	66.1% (51.49% to 81.33%)	226.96 (196.92 to 244.3)	158.74 (123.48 to 177.23)	366.86 (306.5 to 399.52)	237.25 (177.14 to 268.22)	61.64% (51.33% to 72.69%)	49.46% (39.31% to 57.97%)
**90-94 years**	25.53 (20.76 to 28.45)	16.67 (12.61 to 19.18)	53.07 (41.85 to 60.01)	36.27 (25.84 to 43.28)	107.86% (87.48% to 129.24%)	117.59% (94.23% to 138.39%)	184.21 (151.08 to 200.92)	117.86 (88.86 to 133.24)	330.92 (263.83 to 366.29)	219.17 (157.19 to 251.45)	79.64% (69.6% to 89.06%)	85.96% (75.24% to 94.45%)
**95+ years**	23.62 (17.54 to 27.45)	13.13 (9.18 to 15.45)	47.15 (34.3 to 55.16)	38.99 (26.69 to 47.17)	99.59% (78.6% to 122.32%)	196.98% (166.94% to 229.79%)	174.74 (129.82 to 198.68)	94.03 (66.32 to 108.6)	298.6 (217.3 to 345.09)	237.68 (165.3 to 275.73)	70.88% (59.17% to 82.54%)	152.77% (138.61% to 166.66%)

© 2025 GBD 2021 US CNS Cancer Collaborators. *JAMA Neurology*.

eTable 4b: Death, prevalence rate of Brain and central nervous system cancer by age and sex, 1990-2021, United States

	Deaths (95% UI)						Prevalence (95% UI)					
	1990		2021		Percentage Change, 1990-2021		1990		2021		Percentage Change, 1990-2021	
	Male	Female	Male	Female	Male	Female	Male	Female	Male	Female	Male	Female
**<5 years**	0.95 (0.92 to 0.98)	0.9 (0.86 to 0.93)	0.6 (0.52 to 0.69)	0.5 (0.44 to 0.56)	-36.32% (-45.53% to -27.25%)	-44.23% (-50.52% to -37.12%)	23.71 (21.51 to 26.23)	23.21 (20.64 to 26.45)	21.36 (17.9 to 24.92)	17.53 (14.79 to 20.71)	-9.91% (-26.41% to 6.4%)	-24.49% (-37.93% to -8.26%)
**5-9 years**	1.27 (1.23 to 1.32)	1.27 (1.22 to 1.3)	0.93 (0.85 to 1.02)	0.9 (0.85 to 0.96)	-26.73% (-33.62% to -17.83%)	-28.82% (-33.45% to -23.01%)	21.17 (19.3 to 23.27)	21.48 (19.34 to 23.82)	21.63 (19.22 to 24.86)	21.34 (18.5 to 24.45)	2.17% (-13.85% to 19.81%)	-0.64% (-16.8% to 19.53%)
**10-14 years**	1.04 (1.01 to 1.08)	0.89 (0.86 to 0.92)	0.85 (0.79 to 0.91)	0.78 (0.73 to 0.82)	-18.77% (-25.17% to -11.94%)	-12.78% (-18.17% to -6.86%)	19.83 (18.2 to 22.02)	18.47 (16.56 to 20.77)	22.52 (20.03 to 25.45)	22.48 (19.9 to 25.65)	13.56% (-1.42% to 30.85%)	21.74% (2.28% to 44.3%)
**15-19 years**	0.86 (0.82 to 0.89)	0.64 (0.62 to 0.66)	0.72 (0.67 to 0.76)	0.53 (0.49 to 0.57)	-16.1% (-23.04% to -8.88%)	-18.08% (-24.44% to -9.47%)	14.73 (13.39 to 16.24)	13.55 (12.06 to 15.4)	17.82 (15.94 to 19.93)	15.35 (13.11 to 17.83)	20.95% (2.67% to 42.7%)	13.27% (-6.98% to 38.41%)
**20-24 years**	0.89 (0.86 to 0.93)	0.67 (0.65 to 0.7)	0.75 (0.71 to 0.79)	0.56 (0.53 to 0.59)	-15.78% (-20.87% to -10.2%)	-17.25% (-22.39% to -12.03%)	15.41 (13.79 to 17.15)	15.53 (13.87 to 17.69)	18.71 (16.86 to 20.87)	17.93 (15.56 to 20.68)	21.39% (5.19% to 40.33%)	15.5% (-3.91% to 36.68%)
**25-29 years**	1.33 (1.28 to 1.38)	0.93 (0.9 to 0.97)	1.08 (1.02 to 1.13)	0.76 (0.72 to 0.8)	-18.93% (-24.58% to -13.21%)	-18.86% (-23.5% to -13.82%)	20.22 (18.48 to 22.28)	19.46 (17.2 to 21.86)	22.89 (20.56 to 25.44)	21.33 (18.75 to 24.47)	13.17% (-2.23% to 31.05%)	9.57% (-6.11% to 30.66%)
**30-34 years**	2.07 (2 to 2.14)	1.38 (1.34 to 1.42)	1.64 (1.56 to 1.72)	1.03 (0.98 to 1.09)	-21.01% (-25.4% to -16.3%)	-24.86% (-29.42% to -20.34%)	23.81 (22.17 to 25.92)	21.6 (19.32 to 24.21)	25.69 (23.29 to 28.23)	21.77 (19.22 to 24.75)	7.9% (-5.52% to 22.11%)	0.81% (-13.5% to 18.76%)
**35-39 years**	3.09 (3 to 3.18)	1.88 (1.83 to 1.94)	2.35 (2.24 to 2.46)	1.5 (1.42 to 1.6)	-24% (-28.08% to -19.55%)	-19.95% (-24.06% to -14.57%)	23.85 (22.31 to 25.62)	20.12 (18.21 to 22.23)	25.22 (23.14 to 27.71)	22.08 (19.65 to 24.93)	5.75% (-4.86% to 18.88%)	9.78% (-6.17% to 29.2%)
**40-44 years**	4.59 (4.42 to 4.74)	2.65 (2.56 to 2.74)	3.25 (3.09 to 3.41)	2.07 (1.97 to 2.19)	-29.13% (-32.53% to -25.38%)	-21.78% (-25.78% to -17.59%)	21.97 (20.17 to 23.84)	17.32 (15.57 to 19.34)	22.11 (20.14 to 24.16)	18.94 (16.79 to 21.31)	0.65% (-11.91% to 13.92%)	9.35% (-7.57% to 28.2%)
**45-49 years**	6.39 (6.17 to 6.57)	3.87 (3.76 to 4)	4.84 (4.62 to 5.04)	3.01 (2.9 to 3.14)	-24.28% (-28.27% to -20.43%)	-22.19% (-25.79% to -18.42%)	23.53 (21.93 to 25.2)	18.17 (16.41 to 20.27)	24.54 (22.56 to 26.7)	19.6 (17.56 to 22.08)	4.31% (-6.12% to 15.97%)	7.87% (-7.09% to 24.81%)
**50-54 years**	9.71 (9.37 to 10.02)	5.91 (5.76 to 6.06)	7.44 (7.13 to 7.72)	4.79 (4.62 to 4.95)	-23.33% (-27.34% to -19.53%)	-18.92% (-22.48% to -15.45%)	28.42 (26.75 to 30.43)	20.3 (18.73 to 22.07)	27.57 (25.58 to 29.77)	21.71 (19.59 to 23.98)	-2.98% (-12.01% to 7.42%)	6.91% (-6.15% to 22.33%)
**55-59 years**	13.81 (13.43 to 14.18)	8.9 (8.64 to 9.15)	11.84 (11.42 to 12.26)	7.41 (7.15 to 7.67)	-14.27% (-17.93% to -10.23%)	-16.77% (-19.81% to -13.51%)	34.17 (32.38 to 36.08)	23.73 (22.16 to 25.7)	35.36 (33.29 to 37.93)	25.42 (23.34 to 27.96)	3.51% (-4.83% to 12.66%)	7.11% (-5.58% to 21.13%)
**60-64 years**	17.82 (17.28 to 18.31)	11.56 (11.12 to 11.93)	15.46 (14.83 to 16.1)	9.98 (9.48 to 10.37)	-13.24% (-17.12% to -8.84%)	-13.69% (-17.27% to -9.9%)	35.65 (34.01 to 37.5)	25.34 (23.45 to 27.36)	37.11 (34.8 to 39.83)	26.65 (24.31 to 29.37)	4.11% (-3.88% to 13.36%)	5.16% (-6.8% to 17.24%)
**65-69 years**	22.43 (21.72 to 23.1)	14.81 (14.09 to 15.35)	20.14 (19.25 to 20.87)	13.27 (12.31 to 13.91)	-10.2% (-13.96% to -5.72%)	-10.38% (-14.54% to -6.34%)	39.88 (37.71 to 42.04)	26.79 (24.78 to 29.14)	41.81 (38.78 to 44.92)	28.94 (26.03 to 32.13)	4.84% (-3.42% to 14.15%)	8.03% (-3.93% to 22.49%)

© 2025 GBD 2021 US CNS Cancer Collaborators. *JAMA Neurology*.

**70-74 years**	25.69 (24.67 to 26.48)	17.57 (16.48 to 18.32)	25.47 (24.23 to 26.55)	16.86 (15.32 to 17.79)	-0.86% (-5.29% to 3.77%)	-4.03% (-8.63% to 0.86%)	39.72 (37.29 to 42.65)	27.24 (24.94 to 29.91)	48.5 (45.08 to 52.49)	31.77 (27.94 to 35.84)	22.09% (11.85% to 34.82%)	16.6% (1.79% to 33.37%)
**75-79 years**	27.83 (26.5 to 28.91)	18.95 (17.18 to 19.94)	30.04 (27.91 to 31.48)	20.23 (17.78 to 21.56)	7.95% (2.5% to 13.6%)	6.73% (1.36% to 11.72%)	31.63 (29.43 to 33.83)	23.86 (21.11 to 26.85)	44.84 (40.32 to 49.64)	31.56 (27.3 to 35.7)	41.77% (27.7% to 57.33%)	32.31% (12.64% to 53.06%)
**80-84 years**	26.59 (24.41 to 27.97)	17.99 (15.02 to 19.52)	33.76 (29.72 to 36)	22.16 (17.75 to 24.49)	26.95% (19.83% to 34.2%)	23.2% (17.16% to 29.62%)	23.19 (20.88 to 25.3)	17.91 (14.46 to 20.53)	39.93 (34.31 to 45.67)	28.46 (21.48 to 33.83)	72.19% (53.24% to 97.03%)	58.96% (36.58% to 87.5%)
**85-89 years**	22.3 (19.44 to 24)	15.64 (12.13 to 17.47)	36.15 (30.18 to 39.21)	23.41 (17.45 to 26.47)	62.07% (51.97% to 72.45%)	49.74% (39.65% to 57.96%)	18.03 (15.07 to 20.05)	15.06 (11.31 to 17.92)	43.44 (35.75 to 50.62)	31.33 (22.23 to 40.1)	140.88% (107.44% to 178.23%)	108.01% (68.3% to 155.19%)
**90-94 years**	20.65 (16.94 to 22.5)	13.14 (9.84 to 14.81)	36.78 (29.4 to 40.6)	24.36 (17.47 to 27.88)	78.11% (68.58% to 87.02%)	85.42% (75.08% to 93.81%)	25.46 (20.15 to 29.79)	19.05 (13.59 to 23.64)	78.8 (59.67 to 95.85)	54.5 (36.95 to 70.5)	209.52% (155.5% to 271.9%)	186.11% (122.32% to 269.82%)
**95+ years**	20.85 (15.41 to 23.74)	11.24 (7.88 to 12.96)	35.44 (25.78 to 40.91)	28.33 (19.81 to 32.89)	69.99% (58.63% to 81.68%)	152% (137.68% to 165.35%)	13.09 (9.73 to 15.27)	7.32 (5.07 to 8.76)	29.97 (22 to 35.37)	24.38 (16.31 to 30.27)	128.97% (100.33% to 160.15%)	233.23% (185.37% to 285.31%)

© 2025 GBD 2021 US CNS Cancer Collaborators. *JAMA Neurology*.

eTable 4c: YLD, YLL rate of Brain and central nervous system cancer by age and sex, 1990-2021, United States

	YLDs (95% UI)						YLLs (95% UI)					
	1990		2021		Percentage Change, 1990-2021		1990		2021		Percentage Change, 1990-2021	
	Male	Female	Male	Female	Male	Female	Male	Female	Male	Female	Male	Female
**<5 years**	1.72 (1.19 to 2.38)	1.68 (1.13 to 2.32)	1.49 (1 to 2.1)	1.23 (0.84 to 1.77)	-13.16% (-31.05% to 5.25%)	-27.27% (-41.55% to -8.83%)	83.64 (80.64 to 86.46)	78.9 (76.16 to 81.71)	53.26 (45.54 to 60.93)	43.99 (39.07 to 49.47)	-36.32% (-45.54% to -27.25%)	-44.24% (-50.5% to -37.19%)
**5-9 years**	1.67 (1.1 to 2.27)	1.69 (1.16 to 2.35)	1.63 (1.11 to 2.35)	1.59 (1.05 to 2.26)	-2.48% (-24.91% to 22.91%)	-5.9% (-27.1% to 21.46%)	105.18 (101.75 to 109.51)	104.87 (101.18 to 108.11)	77.01 (70.23 to 84.32)	74.62 (70.47 to 79.69)	-26.78% (-33.67% to -17.88%)	-28.85% (-33.47% to -23.04%)
**10-14 years**	1.48 (1.02 to 2.07)	1.37 (0.92 to 1.99)	1.59 (1.08 to 2.25)	1.58 (1.07 to 2.31)	8.09% (-18.78% to 43.93%)	14.93% (-10.46% to 55.08%)	80.75 (78.15 to 83.89)	68.89 (66.48 to 71.49)	65.49 (60.73 to 70.58)	60 (56.71 to 63.67)	-18.9% (-25.29% to -12.09%)	-12.9% (-18.28% to -6.99%)
**15-19 years**	1.14 (0.76 to 1.61)	1.01 (0.69 to 1.42)	1.28 (0.88 to 1.81)	1.09 (0.75 to 1.57)	12.68% (-14.88% to 51.05%)	7.33% (-19.31% to 45.37%)	61.92 (59.54 to 64.28)	46.54 (44.85 to 48)	51.95 (48.4 to 55.33)	38.12 (35.54 to 41.42)	-16.1% (-23.04% to -8.88%)	-18.1% (-24.47% to -9.5%)
**20-24 years**	1.29 (0.89 to 1.8)	1.19 (0.79 to 1.69)	1.48 (1.03 to 2.07)	1.33 (0.86 to 1.89)	14.5% (-11.42% to 47.74%)	10.94% (-14.05% to 46.8%)	60.45 (57.91 to 63.1)	45.55 (43.83 to 47.56)	50.84 (48.31 to 53.46)	37.64 (35.68 to 39.67)	-15.9% (-20.97% to -10.32%)	-17.37% (-22.5% to -12.16%)
**25-29 years**	1.75 (1.24 to 2.37)	1.55 (1.05 to 2.13)	1.89 (1.28 to 2.58)	1.64 (1.12 to 2.29)	7.77% (-14.3% to 34.77%)	5.9% (-15.21% to 35.3%)	83.21 (80.15 to 86.62)	58.42 (56.35 to 60.57)	67.46 (63.85 to 71.09)	47.41 (44.93 to 50.09)	-18.92% (-24.57% to -13.2%)	-18.84% (-23.49% to -13.8%)
**30-34 years**	2.22 (1.54 to 3.07)	1.83 (1.29 to 2.49)	2.28 (1.63 to 3.16)	1.76 (1.22 to 2.43)	2.84% (-13.93% to 27.34%)	-4.03% (-23.2% to 18.82%)	119.58 (115.74 to 123.81)	79.44 (77.12 to 81.78)	94.54 (90.36 to 99.66)	59.72 (56.42 to 63.05)	-20.94% (-25.34% to -16.23%)	-24.82% (-29.39% to -20.31%)
**35-39 years**	2.51 (1.8 to 3.3)	1.9 (1.32 to 2.54)	2.44 (1.69 to 3.24)	1.94 (1.35 to 2.6)	-2.83% (-15.07% to 14.15%)	2.18% (-16.36% to 27.46%)	163.16 (158.74 to 167.75)	99.04 (96.48 to 102.22)	123.94 (118.16 to 129.83)	79.26 (75.19 to 84.22)	-24.04% (-28.11% to -19.59%)	-19.97% (-24.08% to -14.6%)
**40-44 years**	2.77 (1.98 to 3.69)	1.92 (1.36 to 2.54)	2.46 (1.72 to 3.27)	1.89 (1.35 to 2.56)	-11.13% (-23.59% to 2.3%)	-1.42% (-16.91% to 17.99%)	219.93 (211.61 to 227.36)	126.84 (122.57 to 131.33)	155.77 (148.08 to 163.22)	99.22 (94.38 to 104.57)	-29.17% (-32.57% to -25.42%)	-21.78% (-25.78% to -17.59%)
**45-49 years**	3.39 (2.42 to 4.4)	2.34 (1.66 to 3.1)	3.15 (2.24 to 4.16)	2.25 (1.63 to 2.98)	-7.06% (-17.7% to 3.7%)	-3.95% (-17.03% to 10.95%)	274.76 (265.21 to 282.62)	166.32 (161.33 to 171.62)	207.69 (198.16 to 216.51)	129.26 (124.42 to 134.82)	-24.41% (-28.39% to -20.56%)	-22.28% (-25.88% to -18.52%)
**50-54 years**	4.56 (3.27 to 6.04)	3.04 (2.16 to 3.96)	4.05 (2.94 to 5.28)	2.93 (2.1 to 3.78)	-11.25% (-19.67% to -1.08%)	-3.53% (-14.06% to 10.16%)	370.69 (357.81 to 382.52)	225.51 (219.97 to 231.3)	284.38 (272.32 to 294.72)	183 (176.43 to 188.96)	-23.28% (-27.29% to -19.47%)	-18.85% (-22.41% to -15.38%)
**55-59 years**	6.07 (4.35 to 7.89)	3.98 (2.86 to 5.21)	5.85 (4.17 to 7.63)	3.86 (2.7 to 5.06)	-3.58% (-11.32% to 4.24%)	-3.18% (-13.46% to 7.06%)	461.63 (448.85 to 473.8)	297.43 (288.57 to 305.58)	396.14 (382.05 to 409.99)	247.77 (239.06 to 256.37)	-14.19% (-17.84% to -10.14%)	-16.7% (-19.73% to -13.43%)
**60-64 years**	6.92 (4.8 to 9.18)	4.78 (3.39 to 6.31)	6.79 (4.8 to 8.75)	4.66 (3.3 to 6.14)	-1.97% (-13.46% to 10.23%)	-2.52% (-11.23% to 6.68%)	513.32 (497.88 to 527.27)	332.76 (319.99 to 343.34)	446.48 (428.26 to 464.79)	287.95 (273.68 to 299.22)	-13.02% (-16.91% to -8.62%)	-13.47% (-17.06% to -9.66%)
**65-69 years**	7.8 (5.53 to 10.25)	5.51 (3.82 to 7.23)	7.64 (5.38 to 10.18)	5.52 (3.81 to 7.28)	-2.1% (-22.22% to 25.07%)	0.09% (-8.79% to 9.93%)	544.47 (527.33 to 560.85)	359.17 (341.69 to 372.18)	490.2 (468.58 to 508.06)	322.56 (299.16 to 338.14)	-9.97% (-13.74% to -5.47%)	-10.19% (-14.37% to -6.15%)

© 2025 GBD 2021 US CNS Cancer Collaborators. *JAMA Neurology*.

**70-74 years**	8.18 (5.86 to 11.19)	5.96 (4.17 to 7.77)	8.99 (6.21 to 12.14)	6.38 (4.52 to 8.42)	9.86% (-16.65% to 36.73%)	7.13% (-4.31% to 20.13%)	513.41 (492.97 to 529.17)	350.34 (328.7 to 365.32)	507.97 (483.2 to 529.58)	335.72 (305.15 to 354.2)	-1.06% (-5.49% to 3.56%)	-4.17% (-8.77% to 0.71%)
**75-79 years**	7.56 (5.16 to 10.42)	5.84 (4.09 to 7.65)	9.25 (6.4 to 12.29)	6.44 (4.56 to 8.86)	22.31% (-5.9% to 54.53%)	10.33% (-7.52% to 31.98%)	444.78 (423.57 to 462.06)	301.62 (273.37 to 317.32)	479.09 (445.07 to 501.97)	321.79 (282.91 to 343.04)	7.72% (2.27% to 13.35%)	6.69% (1.31% to 11.67%)
**80-84 years**	6.35 (4.33 to 8.47)	5.07 (3.28 to 6.9)	8.79 (6.07 to 11.64)	6.32 (4.3 to 8.47)	38.5% (9.12% to 73.03%)	24.52% (6.63% to 45.61%)	332.85 (305.53 to 350.09)	223.83 (186.81 to 242.87)	421.22 (370.85 to 449.26)	275.72 (220.84 to 304.71)	26.55% (19.45% to 33.76%)	23.19% (17.14% to 29.61%)
**85-89 years**	5.55 (3.86 to 7.39)	4.43 (2.85 to 6.03)	9.51 (6.28 to 12.74)	6.67 (4.02 to 9.18)	71.16% (38.48% to 108%)	50.35% (24.09% to 82.67%)	221.41 (193.01 to 238.21)	154.31 (119.73 to 172.39)	357.35 (298.3 to 387.57)	230.58 (171.88 to 260.71)	61.4% (51.34% to 71.74%)	49.43% (39.37% to 57.63%)
**90-94 years**	6.29 (4.32 to 8.64)	4.77 (2.86 to 6.51)	14.19 (9.38 to 19.41)	9.59 (6.23 to 13.1)	125.55% (83.92% to 175.7%)	101.17% (63.44% to 145.39%)	177.92 (145.92 to 193.84)	113.09 (84.75 to 127.47)	316.74 (253.22 to 349.65)	209.59 (150.36 to 239.87)	78.02% (68.5% to 86.93%)	85.32% (74.99% to 93.71%)
**95+ years**	5.19 (3.31 to 7.17)	3.19 (1.89 to 4.53)	9.44 (5.79 to 13)	7.72 (4.5 to 11.18)	81.91% (48.66% to 120.63%)	142.27% (91.79% to 198.28%)	169.55 (125.34 to 193.03)	90.84 (63.69 to 104.75)	289.16 (210.35 to 333.76)	229.96 (160.79 to 266.92)	70.55% (59.15% to 82.27%)	153.14% (138.77% to 166.58%)

© 2025 GBD 2021 US CNS Cancer Collaborators. *JAMA Neurology*.

eTable 5a: Incidence, DALY, death rate of Brain and central nervous system cancer by age, both sexes, 1990-2021, United States

	Incidence (95% UI)			DALYs (95% UI)			Deaths (95% UI)		
	**1990**	**2021**	**Percentage Change, 1990-2021**	**1990**	**2021**	**Percentage Change, 1990-2021**	**1990**	**2021**	**Percentage Change, 1990-2021**
**<5 years**	3.46 (3.2 to 3.76)	2.64 (2.28 to 3.03)	-23.76% (-34.42% to -11.56%)	83.03 (80.31 to 85.83)	50.09 (44.23 to 56.34)	-39.67% (-47.13% to -31.94%)	0.92 (0.89 to 0.95)	0.55 (0.49 to 0.62)	-40.07% (-47.45% to -32.39%)
**5-9 years**	3.73 (3.48 to 3.97)	3.34 (3.02 to 3.69)	-10.45% (-20.47% to 0.83%)	106.7 (103.87 to 109.49)	77.45 (72.49 to 82.98)	-27.42% (-32.35% to -21.22%)	1.27 (1.24 to 1.3)	0.92 (0.86 to 0.98)	-27.75% (-32.6% to -21.61%)
**10-14 years**	3.06 (2.85 to 3.29)	3.22 (2.94 to 3.51)	5.22% (-5.61% to 18.12%)	76.39 (74.44 to 78.66)	64.39 (60.96 to 68.2)	-15.71% (-20.44% to -10.61%)	0.97 (0.94 to 1)	0.81 (0.77 to 0.86)	-16.1% (-20.82% to -10.94%)
**15-19 years**	2.34 (2.18 to 2.49)	2.44 (2.23 to 2.68)	4.16% (-7.16% to 17.4%)	55.51 (53.85 to 57.01)	46.38 (43.53 to 49.25)	-16.45% (-22.43% to -10.52%)	0.75 (0.73 to 0.77)	0.62 (0.59 to 0.66)	-16.97% (-22.93% to -11.03%)
**20-24 years**	2.6 (2.41 to 2.82)	2.74 (2.52 to 2.98)	5.48% (-5.2% to 16.22%)	54.38 (52.53 to 56.3)	45.77 (43.92 to 47.52)	-15.84% (-19.63% to -12.07%)	0.79 (0.76 to 0.81)	0.66 (0.63 to 0.68)	-16.39% (-20.1% to -12.79%)
**25-29 years**	3.45 (3.23 to 3.67)	3.47 (3.22 to 3.76)	0.7% (-8.28% to 11.63%)	72.52 (70.65 to 74.69)	59.31 (56.86 to 61.69)	-18.21% (-22.14% to -14.49%)	1.13 (1.1 to 1.16)	0.92 (0.88 to 0.95)	-18.81% (-22.61% to -15.14%)
**30-34 years**	4.51 (4.26 to 4.78)	4.25 (3.95 to 4.55)	-5.89% (-12.96% to 2.85%)	101.48 (99.08 to 104.39)	79.18 (76.28 to 82.32)	-21.98% (-25.21% to -18.61%)	1.72 (1.68 to 1.77)	1.34 (1.29 to 1.39)	-22.48% (-25.64% to -19.11%)
**35-39 years**	5.35 (5.12 to 5.6)	4.92 (4.61 to 5.22)	-8.13% (-14.94% to -0.82%)	133.07 (130.38 to 136.33)	103.65 (99.75 to 107.94)	-22.11% (-25.66% to -18.5%)	2.48 (2.43 to 2.53)	1.92 (1.85 to 2)	-22.44% (-25.85% to -18.83%)
**40-44 years**	6.39 (6.13 to 6.69)	5.39 (5.07 to 5.7)	-15.76% (-20.84% to -10.64%)	175.17 (170.39 to 179.83)	129.37 (124.48 to 134.93)	-26.15% (-29.02% to -23.23%)	3.61 (3.51 to 3.7)	2.66 (2.56 to 2.77)	-26.38% (-29.21% to -23.44%)
**45-49 years**	8.06 (7.74 to 8.38)	6.95 (6.61 to 7.28)	-13.85% (-18.36% to -8.81%)	222.41 (216.57 to 227.23)	170.64 (165.05 to 176.01)	-23.28% (-26.08% to -20.53%)	5.11 (4.97 to 5.22)	3.91 (3.79 to 4.04)	-23.4% (-26.18% to -20.64%)
**50-54 years**	11.03 (10.68 to 11.39)	9.5 (9.12 to 9.89)	-13.87% (-18.12% to -9.21%)	299.87 (292.04 to 307.01)	236.43 (229.47 to 242.72)	-21.16% (-24.02% to -18.25%)	7.76 (7.56 to 7.94)	6.1 (5.92 to 6.27)	-21.38% (-24.27% to -18.51%)
**55-59 years**	14.71 (14.27 to 15.1)	13.44 (12.95 to 13.98)	-8.63% (-12.71% to -4.5%)	380.94 (372.97 to 388.86)	325 (315.87 to 334.2)	-14.68% (-17.38% to -11.91%)	11.25 (11.01 to 11.49)	9.57 (9.3 to 9.86)	-14.92% (-17.58% to -12.18%)
**60-64 years**	17.95 (17.38 to 18.44)	16.6 (15.88 to 17.26)	-7.48% (-11.53% to -3.22%)	422.79 (411.61 to 432.13)	369.9 (355.53 to 381.92)	-12.51% (-15.28% to -9.22%)	14.48 (14.11 to 14.8)	12.62 (12.11 to 13.02)	-12.88% (-15.62% to -9.66%)
**65-69 years**	21.64 (20.85 to 22.31)	20.67 (19.48 to 21.52)	-4.47% (-8.99% to 0.27%)	448.86 (433.24 to 460.65)	408.13 (386.26 to 421.49)	-9.07% (-12.25% to -5.94%)	18.23 (17.59 to 18.7)	16.51 (15.61 to 17.04)	-9.42% (-12.66% to -6.21%)

© 2025 GBD 2021 US CNS Cancer Collaborators. *JAMA Neurology*.

**70-74 years**	23.96 (22.61 to 24.87)	25.25 (23.53 to 26.42)	5.38% (0.69% to 10.06%)	427.01 (407.08 to 439.57)	423.06 (395.84 to 440.6)	-0.93% (-4.55% to 2.8%)	21.04 (20.13 to 21.65)	20.85 (19.48 to 21.69)	-0.93% (-4.42% to 2.84%)
**75-79 years**	24.58 (22.67 to 25.68)	28.93 (25.96 to 30.59)	17.67% (12.05% to 23.29%)	364.42 (339.06 to 378.82)	400.12 (362.88 to 419.89)	9.79% (5.53% to 14.08%)	22.44 (20.95 to 23.29)	24.63 (22.37 to 25.87)	9.77% (5.61% to 14.04%)
**80-84 years**	22.04 (18.99 to 23.67)	30.88 (25.67 to 33.74)	40.16% (32.54% to 48.25%)	267.33 (232.43 to 285.69)	345.12 (291.73 to 372.15)	29.1% (23.6% to 34.41%)	20.99 (18.27 to 22.38)	27.11 (22.84 to 29.2)	29.16% (23.74% to 34.48%)
**85-89 years**	18.42 (15.03 to 20.3)	32.78 (25.85 to 36.57)	77.94% (67.14% to 89.4%)	179.1 (146.67 to 196.07)	287.84 (227.27 to 318.84)	60.72% (53.27% to 68.15%)	17.63 (14.43 to 19.28)	28.39 (22.39 to 31.31)	61.04% (53.5% to 68.66%)
**90-94 years**	18.87 (14.91 to 21.33)	41.93 (31.47 to 48.22)	122.26% (103.77% to 139.21%)	134.3 (104.31 to 149.49)	256.83 (193.1 to 288.95)	91.23% (82.33% to 98.8%)	15 (11.62 to 16.68)	28.54 (21.45 to 32.2)	90.31% (81.49% to 97.55%)
**95+ years**	15.4 (11.04 to 17.91)	41.16 (28.73 to 48.69)	167.32% (144.8% to 190.26%)	111.48 (79.95 to 128.05)	253.89 (178.09 to 294.01)	127.75% (116.82% to 139.67%)	13.32 (9.49 to 15.26)	30.22 (21.27 to 34.96)	126.91% (116.05% to 138.56%)

© 2025 GBD 2021 US CNS Cancer Collaborators. *JAMA Neurology*.

eTable 5b: Prevalence, YLD, YLL rate of Brain and central nervous system cancer by age, both sexes, 1990-2021, United States

	Prevalence (95% UI)			YLDs (95% UI)			YLLs (95% UI)		
	1990	2021	Percentage Change, 1990-2021	1990	2021	Percentage Change, 1990-2021	1990	2021	Percentage Change, 1990-2021
**<5 years**	23.46 (21.68 to 25.51)	19.48 (16.83 to 22.52)	-16.96% (-28.56% to -3.86%)	1.7 (1.16 to 2.35)	1.36 (0.94 to 1.91)	-19.98% (-33% to -4.15%)	81.33 (78.78 to 83.72)	48.73 (43 to 54.98)	-40.08% (-47.45% to -32.44%)
**5-9 years**	21.32 (19.92 to 22.86)	21.49 (19.31 to 23.83)	0.79% (-11.28% to 14.77%)	1.68 (1.14 to 2.3)	1.61 (1.09 to 2.27)	-4.16% (-22.34% to 14.57%)	105.03 (102.28 to 107.55)	75.84 (71.15 to 81.41)	-27.79% (-32.63% to -21.65%)
**10-14 years**	19.17 (17.9 to 20.73)	22.5 (20.48 to 24.73)	17.4% (4.42% to 31.5%)	1.42 (0.99 to 1.98)	1.59 (1.13 to 2.22)	11.3% (-9.3% to 34.19%)	74.97 (73.1 to 77.13)	62.81 (59.41 to 66.46)	-16.22% (-20.94% to -11.07%)
**15-19 years**	14.16 (13.09 to 15.2)	16.61 (15.18 to 18.29)	17.34% (3.63% to 33.22%)	1.08 (0.74 to 1.46)	1.19 (0.83 to 1.65)	10.2% (-9.91% to 34.4%)	54.43 (52.87 to 55.88)	45.19 (42.46 to 47.95)	-16.98% (-22.94% to -11.04%)
**20-24 years**	15.47 (14.27 to 17.02)	18.33 (16.83 to 20.19)	18.49% (4.21% to 31.81%)	1.25 (0.89 to 1.71)	1.41 (0.98 to 1.92)	12.83% (-5.52% to 35.76%)	53.14 (51.43 to 54.91)	44.37 (42.73 to 45.97)	-16.51% (-20.21% to -12.91%)
**25-29 years**	19.84 (18.46 to 21.21)	22.11 (20.4 to 24.06)	11.44% (-0.08% to 24.51%)	1.65 (1.18 to 2.24)	1.77 (1.21 to 2.41)	6.95% (-8.42% to 28.24%)	70.87 (68.97 to 72.97)	57.55 (55.25 to 59.81)	-18.8% (-22.6% to -15.13%)
**30-34 years**	22.7 (21.16 to 24.18)	23.74 (21.94 to 25.49)	4.56% (-4.69% to 15.78%)	2.03 (1.45 to 2.72)	2.02 (1.45 to 2.75)	-0.22% (-12.96% to 16.19%)	99.45 (97.04 to 102.17)	77.16 (74.33 to 80.13)	-22.42% (-25.59% to -19.06%)
**35-39 years**	21.97 (20.67 to 23.35)	23.64 (21.88 to 25.64)	7.61% (-2.47% to 18.87%)	2.2 (1.58 to 2.87)	2.19 (1.57 to 2.84)	-0.64% (-12.97% to 13.9%)	130.87 (128.37 to 133.88)	101.47 (97.75 to 105.55)	-22.47% (-25.88% to -18.86%)
**40-44 years**	19.62 (18.29 to 21)	20.51 (19.03 to 22.18)	4.55% (-5.76% to 15.49%)	2.34 (1.69 to 3.09)	2.17 (1.55 to 2.89)	-7.09% (-17.01% to 3.65%)	172.83 (167.97 to 177.25)	127.2 (122.44 to 132.6)	-26.4% (-29.24% to -23.47%)
**45-49 years**	20.8 (19.59 to 22.05)	22.03 (20.51 to 23.51)	5.95% (-2.94% to 15.45%)	2.86 (2.04 to 3.72)	2.7 (1.93 to 3.56)	-5.69% (-14% to 3.83%)	219.55 (213.7 to 224.3)	167.94 (162.59 to 173.38)	-23.51% (-26.29% to -20.75%)
**50-54 years**	24.25 (23.07 to 25.62)	24.6 (23.13 to 26.22)	1.43% (-5.95% to 9.48%)	3.78 (2.7 to 4.92)	3.48 (2.53 to 4.53)	-7.87% (-14.75% to 1.01%)	296.09 (288.52 to 303.24)	232.95 (225.97 to 239.4)	-21.33% (-24.21% to -18.44%)
**55-59 years**	28.72 (27.47 to 29.95)	30.27 (28.66 to 32.04)	5.4% (-2.1% to 13.39%)	4.98 (3.56 to 6.48)	4.83 (3.42 to 6.35)	-3.02% (-8.9% to 4.2%)	375.96 (367.81 to 383.96)	320.18 (311.02 to 329.83)	-14.84% (-17.5% to -12.09%)
**60-64 years**	30.15 (28.86 to 31.47)	31.68 (29.73 to 33.68)	5.08% (-2.09% to 12.42%)	5.78 (4.04 to 7.66)	5.68 (3.99 to 7.34)	-1.68% (-9.68% to 6.56%)	417.01 (406.37 to 426.07)	364.21 (349.57 to 375.97)	-12.66% (-15.41% to -9.43%)
**65-69 years**	32.66 (31.08 to 34.29)	35.01 (32.83 to 37.24)	7.18% (-0.08% to 15.57%)	6.54 (4.62 to 8.52)	6.52 (4.57 to 8.55)	-0.34% (-12.64% to 14.79%)	442.32 (426.8 to 453.85)	401.61 (379.58 to 414.51)	-9.2% (-12.46% to -5.99%)
**70-74 years**	32.58 (30.62 to 34.45)	39.51 (36.1 to 42.61)	21.27% (12.47% to 32.23%)	6.91 (4.97 to 9.02)	7.59 (5.32 to 9.9)	9.84% (-6.59% to 25.77%)	420.11 (401.84 to 432.32)	415.47 (388.16 to 432.29)	-1.1% (-4.58% to 2.66%)

© 2025 GBD 2021 US CNS Cancer Collaborators. *JAMA Neurology*.

**75-79 years**	26.91 (24.85 to 28.93)	37.52 (33.87 to 40.72)	39.43% (25.91% to 52.33%)	6.52 (4.55 to 8.69)	7.7 (5.4 to 10.19)	18.19% (1.09% to 37.5%)	357.91 (334.06 to 371.42)	392.42 (356.43 to 412.17)	9.64% (5.49% to 13.91%)
**80-84 years**	19.75 (16.84 to 21.68)	33.35 (27.45 to 37.54)	68.9% (52.43% to 87.29%)	5.52 (3.72 to 7.42)	7.37 (5.08 to 9.8)	33.62% (16.47% to 54.76%)	261.81 (227.99 to 279.22)	337.75 (284.62 to 363.9)	29.01% (23.58% to 34.33%)
**85-89 years**	15.95 (12.53 to 18.19)	36.06 (27.82 to 42.76)	126.08% (96.35% to 161.32%)	4.77 (3.13 to 6.42)	7.78 (5.06 to 10.37)	63.07% (40.97% to 86.58%)	174.33 (142.72 to 190.64)	280.07 (220.98 to 308.93)	60.65% (53.12% to 68.26%)
**90-94 years**	20.64 (15.24 to 24.59)	62.69 (46.1 to 74.96)	203.77% (155.42% to 259.71%)	5.14 (3.25 to 7.04)	11.14 (7.31 to 14.92)	116.53% (88.36% to 149.51%)	129.16 (100.08 to 143.66)	245.69 (184.64 to 277.14)	90.23% (81.41% to 97.46%)
**95+ years**	8.56 (6.13 to 10.06)	25.86 (18.08 to 31.25)	202.04% (171.33% to 239.76%)	3.62 (2.18 to 5.11)	8.18 (4.93 to 11.53)	125.93% (88.12% to 164.44%)	107.86 (76.89 to 123.58)	245.71 (172.95 to 284.21)	127.81% (116.96% to 139.49%)

© 2025 GBD 2021 US CNS Cancer Collaborators. *JAMA Neurology*.

eTable 6: Prevalence, YLD, YLL count and age-standardized rate of Brain and central nervous system cancer by state, 2021, and percentage change from 1990 to 2021, United States

	Prevalence (95% UI)			YLDs (95% UI)			YLLs (95% UI)		
	Absolute number, 2021	Age-standardized rate, per 100 000 people, 2021	Percentage Change, 1990-2021	Absolute number, 2021	Age-standardized rate, per 100 000 people, 2021	Percentage Change, 1990-2021	Absolute number, 2021	Age-standardized rate, per 100 000 people, 2021	Percentage Change, 1990-2021
**United States of America**	84402.47 (80958.69 to 87130.86)	23.38 (22.43 to 24.28)	8.28% (4.11% to 12.56%)	11009.32 (7989.38 to 14200)	2.6 (1.92 to 3.4)	2.03% (-2.1% to 6.25%)	583986.83 (560205.4 to 599074.57)	131.78 (127.25 to 134.95)	-16.06% (-18.03% to -14%)
**Alabama**	1280.71 (1090.8 to 1467.92)	23.4 (20.08 to 26.85)	12.11% (-6.5% to 32.03%)	182.18 (125.17 to 247.38)	2.81 (1.96 to 3.84)	8.07% (-10.76% to 27.78%)	11919.02 (10286.49 to 13754.68)	177.36 (154.7 to 202.81)	-2.43% (-14.76% to 12.41%)
**Alaska**	206.34 (170.98 to 265.27)	28.9 (23.25 to 39.3)	55.88% (22.17% to 110.4%)	23.57 (16.8 to 32.52)	2.94 (2.07 to 4.36)	32.65% (6.52% to 72.42%)	1212.85 (1040.75 to 1417.68)	143.06 (121.65 to 173.3)	-0.58% (-16.15% to 21.73%)
**Arizona**	1879.58 (1614.77 to 2182.12)	24.48 (21.07 to 28.54)	18.03% (-1.02% to 41.36%)	240.58 (167.56 to 321.35)	2.66 (1.86 to 3.61)	11.28% (-7.73% to 35.78%)	12360.65 (10600.42 to 14236.6)	130.52 (113.17 to 149.8)	-8.96% (-21.22% to 4.29%)
**Arkansas**	772.76 (666.96 to 889.85)	24.13 (20.68 to 27.98)	7.64% (-10.2% to 27.54%)	106.37 (75.53 to 142.11)	2.82 (2 to 3.71)	3.7% (-14.58% to 25.31%)	6746.29 (5809.6 to 7776.21)	173.45 (150.62 to 198.45)	-5.57% (-18.77% to 8.88%)
**California**	9671.5 (8396.9 to 11279.52)	23.05 (19.86 to 26.87)	3.4% (-12.7% to 22.61%)	1180.55 (837.39 to 1540.8)	2.43 (1.73 to 3.23)	-3.36% (-19.04% to 15.27%)	60133.68 (52356.23 to 68113.03)	116.75 (103.33 to 131.44)	-22.46% (-31.81% to -12.43%)
**Colorado**	1566.44 (1308.11 to 1837.03)	24.77 (20.81 to 28.87)	9.2% (-9.41% to 30.08%)	196.16 (134.59 to 267.2)	2.69 (1.86 to 3.64)	3.88% (-15.4% to 28.55%)	9704.03 (8109.27 to 11317.12)	126.08 (107.21 to 145.48)	-16.46% (-29.8% to -3.14%)
**Connecticut**	1424.28 (1186.67 to 1723.56)	32.69 (27.42 to 38.97)	2% (-15.81% to 22.94%)	169.4 (116.14 to 228.6)	3.41 (2.36 to 4.52)	-2.12% (-21.08% to 20.37%)	6135.63 (5111.75 to 7220.7)	120.91 (100.82 to 140.72)	-18.44% (-31.61% to -4.41%)
**Delaware**	241.16 (209.75 to 274.82)	22.28 (19.4 to 25.45)	20.94% (2.46% to 42.31%)	32.52 (23.31 to 42.83)	2.5 (1.76 to 3.3)	9.92% (-8.32% to 31.58%)	1740.36 (1542.34 to 1954.64)	127.57 (114.14 to 142.32)	-13.26% (-23.64% to -2.57%)
**District of Columbia**	85 (72.26 to 98.78)	12.33 (10.39 to 14.37)	-4.8% (-20.68% to 12.93%)	12.35 (8.56 to 16.31)	1.57 (1.1 to 2.07)	-15.94% (-30.08% to 0.61%)	791.34 (676.76 to 912.28)	99.24 (85.67 to 113.89)	-43.53% (-52.01% to -34.23%)
**Florida**	4673.41 (4001.93 to 5420.73)	21.81 (18.65 to 25.3)	6.13% (-11.45% to 26.42%)	648.62 (422.72 to 891.9)	2.34 (1.59 to 3.23)	1.32% (-15.38% to 24.07%)	40376.49 (34475.39 to 46747.59)	132.49 (114.99 to 152.14)	-16.81% (-27.56% to -4.4%)
**Georgia**	2383.26 (2043.61 to 2779.76)	20.46 (17.57 to 23.94)	16.15% (-1.56% to 36.79%)	322.24 (219.91 to 435.15)	2.4 (1.66 to 3.22)	4.6% (-13.94% to 24.72%)	18216.07 (15784.37 to 20945.98)	132.13 (115.67 to 150.71)	-18.31% (-28.21% to -6.46%)
**Hawaii**	252.58 (214.96 to 289.48)	16.18 (13.74 to 18.72)	6.41% (-12.46% to 27.71%)	34.6 (24.46 to 46.16)	1.82 (1.3 to 2.42)	3.33% (-14.51% to 24.78%)	1731.1 (1468.56 to 2002.99)	87.1 (74.67 to 100.93)	-14.83% (-27.08% to -1.4%)

© 2025 GBD 2021 US CNS Cancer Collaborators. *JAMA Neurology*.

**Idaho**	494.72 (413.81 to 569.02)	24.29 (20.45 to 27.87)	7.15% (-11.81% to 27.08%)	63.77 (44.36 to 86.68)	2.74 (1.88 to 3.68)	3.82% (-17.09% to 25.43%)	3368.57 (2826.13 to 3936.39)	139.17 (118.2 to 161.06)	-12.15% (-25.63% to 2.49%)
**Illinois**	3119.08 (2686.51 to 3649.48)	22.87 (19.69 to 26.58)	16.31% (-1.06% to 36.44%)	406.84 (280.02 to 565.24)	2.53 (1.77 to 3.46)	6.44% (-11.24% to 27.96%)	20989.02 (17867.07 to 24218.27)	124.41 (107.63 to 142.16)	-19.04% (-30.29% to -7.11%)
**Indiana**	1733.55 (1487.55 to 1992.48)	23.95 (20.44 to 27.44)	7.97% (-10.41% to 26.92%)	229.51 (157.95 to 304.58)	2.7 (1.87 to 3.58)	3.82% (-14.39% to 24.18%)	13048.09 (11251.07 to 14990.81)	146.76 (128.19 to 167.25)	-7.5% (-20.09% to 6.31%)
**Iowa**	792.19 (668.39 to 926.68)	22.38 (18.79 to 26.27)	15.1% (-5.95% to 39.72%)	115.99 (80.23 to 162.79)	2.76 (1.93 to 3.85)	9.93% (-11.33% to 34.85%)	6354.77 (5368 to 7361.79)	148.91 (127.37 to 170.85)	-3.21% (-18.11% to 12.21%)
**Kansas**	872.41 (741.15 to 1015.93)	27.6 (23.55 to 32)	10.63% (-7.43% to 32.17%)	111.59 (78.44 to 146.82)	3.06 (2.15 to 4.01)	8.33% (-13.78% to 32.23%)	5953.54 (4987.99 to 6927.6)	156.14 (131.75 to 181.04)	-1.82% (-16.85% to 14.15%)
**Kentucky**	1453.94 (1231.46 to 1703.69)	28.67 (24.41 to 33.52)	6.43% (-12.38% to 28.36%)	190.79 (131.93 to 252.79)	3.29 (2.28 to 4.37)	4.36% (-14.89% to 27.62%)	9743.95 (8332.69 to 11164.82)	162.11 (139.09 to 184.79)	-3.01% (-16.67% to 11.64%)
**Louisiana**	1095.35 (944.84 to 1278.79)	22.07 (19.1 to 25.86)	17.19% (-0.65% to 39.28%)	156.54 (108.95 to 213.49)	2.73 (1.91 to 3.72)	11.97% (-7.89% to 35.88%)	9212.16 (7998.32 to 10650.81)	155.17 (135.26 to 177.27)	-2.46% (-15.76% to 12.28%)
**Maine**	402.87 (340.7 to 475.68)	25.74 (21.91 to 30.13)	11.8% (-6.81% to 35.2%)	56.44 (39.54 to 78.59)	2.91 (2.03 to 4.04)	10.26% (-10.75% to 35.13%)	3017.56 (2587.54 to 3516.03)	146.21 (126.07 to 169.77)	-2.25% (-17.62% to 13.82%)
**Maryland**	1372.39 (1175.91 to 1601.73)	20.83 (17.82 to 24.34)	6.34% (-11.37% to 27.43%)	180.28 (124.53 to 251.53)	2.33 (1.63 to 3.25)	-3.15% (-20.97% to 17.02%)	9745.63 (8219.68 to 11425.14)	120.03 (102.5 to 139.57)	-23.95% (-35.68% to -11.53%)
**Massachusetts**	2064.93 (1702.53 to 2506.22)	24.88 (20.88 to 29.62)	-0.3% (-17.44% to 20.76%)	267.99 (182.48 to 365.1)	2.75 (1.89 to 3.75)	-2.78% (-21.04% to 19.04%)	12131.14 (10084.34 to 14395.89)	118.63 (99.57 to 140.31)	-23.91% (-37% to -9.5%)
**Michigan**	2562.93 (2167.09 to 3016.29)	21.45 (18.35 to 25.16)	10.44% (-7.01% to 30.23%)	361.77 (253.23 to 493.8)	2.62 (1.85 to 3.57)	1.92% (-17.34% to 22.68%)	19583.72 (16892.98 to 22620.33)	141.89 (123.39 to 161.69)	-15.1% (-26.6% to -2.31%)
**Minnesota**	1870.96 (1576.67 to 2200.58)	30.3 (25.48 to 35.4)	11.51% (-9.57% to 33.11%)	226.59 (155.83 to 300.81)	3.14 (2.15 to 4.22)	4.19% (-16.7% to 26.99%)	10158.08 (8496.26 to 11852.83)	132.72 (112.72 to 153.84)	-15.21% (-28.5% to -1.4%)
**Mississippi**	734.43 (624.49 to 843.29)	23.41 (20.12 to 26.93)	18.71% (0.25% to 40.94%)	104.67 (69.08 to 139.5)	2.85 (1.93 to 3.79)	14.69% (-5.57% to 39.78%)	7114.6 (6165.26 to 8208.85)	188.15 (163.68 to 215.11)	4.71% (-8.86% to 20.14%)
**Missouri**	1643.8 (1402.97 to 1924.55)	24.83 (21.34 to 28.81)	14.07% (-3.85% to 36.18%)	219.51 (151.45 to 287.24)	2.79 (1.98 to 3.7)	8.17% (-10.81% to 33.33%)	12340.24 (10519.82 to 14322.49)	149.72 (128.69 to 172.19)	-6.51% (-20.91% to 8.29%)
**Montana**	297.3 (255.84 to 343.8)	25.58 (22.2 to 29.61)	20.08% (0.7% to 40.9%)	39.88 (28.38 to 53.7)	2.82 (2.03 to 3.89)	14.16% (-4.15% to 36.54%)	2181.77 (1883.12 to 2528.63)	146.59 (128.49 to 168.97)	-1.46% (-14.92% to 14.45%)
**Nebraska**	627.83 (534.99 to 726.83)	29.85 (25.56 to 34.51)	12.55% (-6.65% to 35.13%)	78.46 (55.4 to 105.49)	3.23 (2.27 to 4.33)	9.18% (-9.66% to 32.68%)	3931.94 (3355.24 to 4518.18)	154.74 (133.5 to 177.77)	-7.26% (-21.2% to 7.79%)

© 2025 GBD 2021 US CNS Cancer Collaborators. *JAMA Neurology*.

**Nevada**	663.96 (571.39 to 760.45)	19.6 (16.79 to 22.59)	9.1% (-7.98% to 28.21%)	89.91 (62.68 to 118.33)	2.28 (1.6 to 2.98)	2.73% (-13.06% to 22.71%)	4928.06 (4199.28 to 5701.43)	120 (103.48 to 137.53)	-17.54% (-29.4% to -4.84%)
**New Hampshire**	405.84 (340.41 to 471.04)	25.61 (21.68 to 29.76)	17.75% (-2.04% to 40.53%)	54.25 (37.11 to 72.9)	2.81 (1.94 to 3.78)	10.71% (-8.63% to 32.66%)	2666.96 (2271.69 to 3115.01)	128.33 (109.68 to 149.24)	-11.6% (-25.06% to 3.81%)
**New Jersey**	4175.9 (3505.97 to 4905.06)	37 (31.61 to 43.37)	5.32% (-13.62% to 25.46%)	450.11 (312.91 to 616.36)	3.58 (2.52 to 4.95)	1.35% (-19.03% to 24.06%)	14667.51 (12435.62 to 17263.5)	112.24 (96.03 to 130.73)	-23.43% (-35.3% to -10.77%)
**New Mexico**	522.7 (441.4 to 611.27)	22.66 (19.42 to 26.7)	28.31% (5.04% to 54.72%)	68.03 (47.42 to 91.48)	2.53 (1.73 to 3.41)	24.6% (0.78% to 49.03%)	3783.18 (3234.44 to 4419.67)	137.65 (118.21 to 160.04)	6.04% (-9.97% to 24.14%)
**New York**	4468.61 (3852.92 to 5232.51)	20.43 (17.56 to 23.61)	14.81% (-1.98% to 36.17%)	581.26 (418.56 to 775.69)	2.23 (1.59 to 3)	3.7% (-14.2% to 25.39%)	28584.52 (24442.5 to 33250.81)	104.85 (90.95 to 120.5)	-26.16% (-36.25% to -14.75%)
**North Carolina**	2462.21 (2112.97 to 2845.11)	21.77 (18.81 to 24.9)	13.36% (-3.95% to 33.86%)	331.26 (233.53 to 444.09)	2.48 (1.75 to 3.32)	3.07% (-14.54% to 23.6%)	18737.97 (15995.71 to 21676.21)	133.68 (115.11 to 153.31)	-17.7% (-29.55% to -4.93%)
**North Dakota**	225.83 (196.15 to 261.5)	28.1 (24.21 to 32.88)	14.82% (-3.32% to 37.09%)	27.59 (18.78 to 36.24)	2.95 (2.02 to 3.91)	5.91% (-14.74% to 29.79%)	1421 (1227.1 to 1621.02)	146.42 (127.34 to 166.15)	-8.64% (-20.74% to 5.47%)
**Ohio**	2948.27 (2531.56 to 3403.96)	22.85 (19.7 to 26.29)	6.87% (-9.3% to 26.73%)	407.32 (289.98 to 543.8)	2.64 (1.86 to 3.51)	4.83% (-12.61% to 24.92%)	23531.51 (20303.29 to 26956.72)	145.54 (126.82 to 165.44)	-6.76% (-18.2% to 6.36%)
**Oklahoma**	911.01 (792.05 to 1041.48)	21.48 (18.61 to 24.43)	0.54% (-15.65% to 18.48%)	126.47 (87.78 to 173.35)	2.56 (1.77 to 3.46)	0.64% (-17.47% to 20.73%)	7860.3 (6849.26 to 9000.76)	155.1 (136.31 to 175.83)	-2.29% (-14.56% to 11.18%)
**Oregon**	1303.25 (1108.22 to 1548.32)	27.43 (23.32 to 32.18)	3.76% (-14.01% to 24.44%)	168.31 (118.6 to 234.9)	2.97 (2.08 to 4.13)	-0.79% (-17.66% to 23.31%)	8202.34 (7070.87 to 9685.88)	136.43 (118.49 to 159.74)	-20.28% (-31.48% to -6.26%)
**Pennsylvania**	3360.97 (2888.35 to 3856.81)	23.79 (20.76 to 27.26)	9.38% (-7.91% to 28.8%)	450.47 (305.63 to 621.34)	2.62 (1.79 to 3.68)	1.48% (-16.41% to 23.23%)	23915.49 (20452.68 to 27583.01)	132.23 (114.69 to 150.83)	-17.61% (-28.67% to -5.54%)
**Rhode Island**	287.03 (238.14 to 340.92)	23.44 (19.54 to 27.85)	3.17% (-15.35% to 22.12%)	38.41 (27 to 53.36)	2.58 (1.84 to 3.58)	-3.81% (-22.38% to 19.44%)	1904.29 (1609.2 to 2238.97)	121.62 (102.61 to 142.53)	-25.04% (-37.98% to -11.11%)
**South Carolina**	1296.11 (1101.8 to 1482.11)	23.1 (19.9 to 26.22)	23.68% (2.93% to 45.27%)	181.04 (125.08 to 245.41)	2.7 (1.89 to 3.66)	12.35% (-8.84% to 34.03%)	10928.57 (9323.01 to 12602.18)	156.98 (135.9 to 179.07)	-11.48% (-23.33% to 2.65%)
**South Dakota**	259.19 (226.2 to 294.17)	28.05 (24.25 to 31.89)	11.62% (-6.93% to 31.79%)	33.43 (24.12 to 44.15)	3.08 (2.24 to 4.05)	7.95% (-10.8% to 29.26%)	1849.53 (1625.29 to 2114.59)	163.65 (145.07 to 185.17)	-4.21% (-16.52% to 9.39%)
**Tennessee**	1814.54 (1566.9 to 2121.42)	24.2 (20.9 to 28.07)	15.6% (-2.41% to 36.07%)	247.85 (177.79 to 331.14)	2.81 (2.02 to 3.74)	8.54% (-10.86% to 30.17%)	14974.83 (12904.03 to 17356.79)	162.63 (142.02 to 187)	-8.25% (-20.72% to 5.71%)
**Texas**	6464.01 (5636.98 to 7368.23)	20.77 (18.18 to 23.68)	0.02% (-13.63% to 16.95%)	831.99 (587.66 to 1108.53)	2.38 (1.68 to 3.14)	-5.99% (-21.89% to 12.6%)	44965.2 (39596.46 to 51181.17)	123.37 (109.4 to 139.19)	-23.98% (-32.97% to -13.77%)

© 2025 GBD 2021 US CNS Cancer Collaborators. *JAMA Neurology*.

**Utah**	763.66 (661.9 to 879.88)	22.01 (19.09 to 25.41)	6.54% (-10.82% to 25.99%)	92.09 (65.03 to 123.28)	2.52 (1.77 to 3.37)	4.11% (-13.78% to 26.37%)	4650.76 (4047.62 to 5343.19)	125.45 (109.61 to 143.62)	-13.83% (-25.19% to -1.2%)
**Vermont**	181.55 (158.82 to 207.76)	25.75 (22.34 to 29.52)	31.79% (12.52% to 54.41%)	24.12 (17.5 to 31.72)	2.78 (1.95 to 3.79)	17.56% (-2.97% to 40.76%)	1224.18 (1082.89 to 1401.83)	131.73 (118.06 to 148.91)	-10.79% (-20.94% to 1.07%)
**Virginia**	2195.44 (1846.9 to 2572.26)	23.65 (20.02 to 27.78)	18.55% (-1.14% to 41.72%)	282.39 (200.2 to 382.49)	2.59 (1.82 to 3.51)	8.13% (-10.68% to 31.3%)	14290.32 (12145.34 to 16707.33)	124.63 (106.23 to 144.3)	-16.03% (-28.06% to -2.45%)
**Washington**	1758.59 (1500.56 to 2045.82)	20.51 (17.69 to 23.73)	4.49% (-11.54% to 22.72%)	251.79 (170.44 to 337.03)	2.49 (1.7 to 3.34)	-4.32% (-21.5% to 16.23%)	14336.48 (12039.22 to 16602.81)	136.7 (115.29 to 156.65)	-19.95% (-32.71% to -7.46%)
**West Virginia**	456 (394.07 to 531.9)	23.21 (20 to 26.84)	30.14% (9.96% to 54.12%)	66.69 (46.97 to 89.93)	2.78 (1.95 to 3.71)	25.79% (4.48% to 53.39%)	4218.67 (3650.56 to 4876.82)	168.72 (146.5 to 194.48)	15.97% (0.13% to 34.18%)
**Wisconsin**	1755.74 (1458.28 to 2034.49)	27.6 (23.3 to 31.87)	5.35% (-13.71% to 25.77%)	225.29 (159.99 to 303.78)	2.97 (2.11 to 4.08)	1.36% (-18.51% to 21.69%)	11328.55 (9639.44 to 13294.57)	140.85 (121.09 to 162.61)	-11.76% (-24.77% to 2.97%)
**Wyoming**	146.36 (126.85 to 165.11)	23.24 (20.06 to 26.35)	8.84% (-8.87% to 27.17%)	19.52 (13.82 to 25.87)	2.65 (1.87 to 3.55)	7.03% (-10.37% to 26.07%)	1074.33 (954.83 to 1210.49)	139.5 (124.67 to 156.52)	-7.92% (-18.12% to 4.33%)

© 2025 GBD 2021 US CNS Cancer Collaborators. *JAMA Neurology*.

eTable 7a: Correlation of Incidence, DALYs, death count and age-standardized rate of Brain and central nervous system cancer by state, 2021, and percentage change from 1990 to 2021 with SDI, United States

	Incidence (95% UI)			DALYs (95% UI)			Deaths (95% UI)			
	Absolute number, 2021	Age-standardized rate, per 100 000 people, 2021	Percentage Change, 1990-2021	Absolute number, 2021	Age-standardized rate, per 100 000 people, 2021	Percentage Change, 1990-2021	Absolute number, 2021	Age-standardized rate, per 100 000 people, 2021	Percentage Change, 1990-2021	SDI
**United States of America**	31780 (29971.1 to 32843.9)	6.91 (6.58 to 7.12)	-1.45% (-4.41% to 0.91%)	594996.15 (571294.69 to 610278.4)	134.38 (129.83 to 137.95)	-15.77% (-17.75% to -13.68%)	21444.08 (20045.85 to 22166.97)	4.1 (3.87 to 4.22)	-8.41% (-11.09% to -6.22%)	0.859
**Alabama**	577.2 (494.55 to 664.25)	8.15 (7.05 to 9.33)	8.51% (-7.36% to 25.85%)	12101.2 (10421.29 to 13949.93)	180.17 (157.11 to 205.96)	-2.28% (-14.59% to 12.56%)	431.1 (369.87 to 497.72)	5.44 (4.69 to 6.26)	5.79% (-8.7% to 22.35%)	0.819
**Alaska**	62.55 (54.14 to 73.83)	7.38 (6.29 to 9.24)	18.53% (-0.7% to 47.07%)	1236.42 (1060.7 to 1443.72)	146 (124.29 to 176.99)	-0.07% (-15.75% to 22.19%)	37.31 (32.51 to 42.78)	3.9 (3.38 to 4.51)	-4.8% (-17.4% to 10.28%)	0.851
**Arizona**	689.13 (592.22 to 794.93)	6.97 (6.02 to 8.01)	8.88% (-6.21% to 26.03%)	12601.23 (10792.16 to 14520.14)	133.18 (115.31 to 152.62)	-8.63% (-20.92% to 4.66%)	454.62 (390.36 to 525.31)	3.99 (3.45 to 4.6)	1.42% (-12.51% to 16.81%)	0.842
**Arkansas**	329.31 (282.82 to 379.18)	8.02 (6.95 to 9.18)	3.75% (-11.37% to 19.38%)	6852.66 (5916.1 to 7889.39)	176.27 (153.36 to 201.8)	-5.44% (-18.7% to 8.89%)	239.37 (205.65 to 276.38)	5.17 (4.47 to 5.96)	0.64% (-13.7% to 16.89%)	0.811
**California**	3222.04 (2821.36 to 3667.82)	6.15 (5.42 to 7.02)	-6.16% (-18.7% to 7.46%)	61314.24 (53415.78 to 69330.06)	119.18 (105.5 to 134.14)	-22.15% (-31.47% to -12.1%)	2210.66 (1869.9 to 2503.29)	3.7 (3.17 to 4.19)	-12.56% (-24.18% to -1.2%)	0.87
**Colorado**	546.88 (458.09 to 640.35)	7 (5.88 to 8.18)	-1.08% (-17.16% to 15.51%)	9900.18 (8274.83 to 11553.66)	128.77 (109.46 to 148.96)	-16.11% (-29.51% to -2.75%)	348.79 (287.07 to 408.63)	3.96 (3.29 to 4.62)	-9.33% (-24.2% to 6%)	0.877
**Connecticut**	441.28 (364.92 to 524.88)	8.3 (6.93 to 9.77)	-3.87% (-19.98% to 14.96%)	6305.03 (5250.93 to 7410.65)	124.32 (103.84 to 144.44)	-18.07% (-31.23% to -3.83%)	233.62 (195.62 to 275.14)	3.8 (3.17 to 4.46)	-11.33% (-25.37% to 4.96%)	0.902
**Delaware**	93.57 (82.96 to 105.27)	6.56 (5.78 to 7.36)	1.63% (-10.61% to 14.82%)	1772.88 (1569.1 to 1993.9)	130.07 (116.15 to 144.98)	-12.9% (-23.3% to -2.16%)	63.6 (56.43 to 72.18)	3.85 (3.42 to 4.34)	-10.88% (-21.09% to 0.47%)	0.863
**District of Columbia**	36.01 (30.73 to 41.41)	4.36 (3.76 to 5.02)	-23.7% (-36.04% to -10.9%)	803.7 (688.5 to 927.22)	100.8 (87.13 to 116.03)	-43.24% (-51.77% to -33.88%)	26.1 (22.39 to 30.1)	2.94 (2.53 to 3.39)	-34.07% (-44.23% to -22.97%)	0.886
**Florida**	2014.68 (1705.77 to 2361.49)	6.25 (5.38 to 7.3)	-1.4% (-16.34% to 15.53%)	41025.1 (35027.99 to 47563.22)	134.83 (116.98 to 154.42)	-16.55% (-27.28% to -4.04%)	1550.85 (1306.73 to 1804.19)	4.05 (3.45 to 4.68)	-9% (-21.41% to 4.78%)	0.856
**Georgia**	944.64 (817.93 to 1088)	6.63 (5.75 to 7.6)	-0.94% (-13.4% to 15.11%)	18538.3 (16038.82 to 21346.26)	134.53 (117.65 to 153.81)	-17.99% (-27.91% to -6.08%)	642.28 (552.39 to 742.02)	4.09 (3.54 to 4.7)	-10.12% (-21.77% to 3.63%)	0.841
**Hawaii**	98.41 (83.81 to 113.45)	4.7 (4.04 to 5.38)	0.44% (-15.36% to 16.65%)	1765.7 (1500.81 to 2043.79)	88.92 (76.15 to 103.05)	-14.52% (-26.75% to -1.07%)	64.48 (54.2 to 75.4)	2.65 (2.25 to 3.06)	-7.3% (-21.29% to 8.23%)	0.86

© 2025 GBD 2021 US CNS Cancer Collaborators. *JAMA Neurology*.

**Idaho**	186.15 (155.07 to 215.44)	7.41 (6.22 to 8.51)	2.07% (-14.3% to 17.97%)	3432.33 (2882.96 to 4000.04)	141.91 (120.67 to 164.02)	-11.89% (-25.48% to 2.71%)	124.88 (104.84 to 145.33)	4.45 (3.73 to 5.18)	-3.44% (-18.46% to 12.87%)	0.827
**Illinois**	1153.81 (983.09 to 1328.59)	6.61 (5.67 to 7.63)	-0.73% (-14.83% to 14.85%)	21395.86 (18231.59 to 24675.02)	126.94 (110.01 to 145.35)	-18.65% (-29.96% to -6.79%)	763.47 (644.06 to 882.76)	3.84 (3.27 to 4.43)	-12.17% (-24.69% to 1.4%)	0.872
**Indiana**	683.75 (590.63 to 785.55)	7.4 (6.43 to 8.46)	3.6% (-11.72% to 19.33%)	13277.6 (11457.27 to 15223.53)	149.46 (130.71 to 170.79)	-7.32% (-19.9% to 6.49%)	473.13 (400.75 to 545.55)	4.53 (3.89 to 5.2)	0.1% (-13.45% to 15.3%)	0.838
**Iowa**	369.42 (314.54 to 424.47)	8.1 (6.91 to 9.31)	10.1% (-7.45% to 30.95%)	6470.76 (5462.96 to 7484.52)	151.68 (129.75 to 173.7)	-3% (-17.89% to 12.42%)	238.12 (202.57 to 276.29)	4.6 (3.89 to 5.31)	3% (-13.12% to 19.52%)	0.864
**Kansas**	325.6 (274.03 to 379.48)	8.21 (6.96 to 9.48)	8.55% (-8.11% to 27.71%)	6065.12 (5093.86 to 7057.89)	159.2 (134.37 to 184.4)	-1.65% (-16.67% to 14.31%)	217.02 (181.89 to 252.41)	4.85 (4.06 to 5.63)	6.28% (-10.57% to 23.74%)	0.858
**Kentucky**	555.83 (476.87 to 643.56)	8.94 (7.69 to 10.3)	4.12% (-10.6% to 22.22%)	9934.74 (8481.61 to 11386.97)	165.41 (141.72 to 189.2)	-2.87% (-16.54% to 11.71%)	347.09 (296.82 to 398.74)	4.93 (4.22 to 5.64)	1.86% (-12.88% to 17.21%)	0.815
**Louisiana**	493.28 (425.83 to 572.7)	8 (6.91 to 9.28)	11.74% (-3.52% to 29.91%)	9368.7 (8129.14 to 10827.33)	157.9 (137.87 to 180.31)	-2.25% (-15.6% to 12.5%)	318.33 (274.31 to 368.21)	4.65 (4.03 to 5.39)	4.59% (-9.95% to 21.43%)	0.823
**Maine**	168.8 (143.45 to 196.71)	7.75 (6.62 to 9.06)	7.85% (-8.56% to 26.84%)	3074 (2633.58 to 3581.83)	149.12 (128.42 to 173.16)	-2.03% (-17.38% to 14.16%)	117.46 (100.96 to 136.39)	4.58 (3.95 to 5.32)	3.04% (-12.96% to 19.54%)	0.862
**Maryland**	514.77 (437.04 to 601.98)	6.14 (5.25 to 7.18)	-10.53% (-24.45% to 5.36%)	9925.91 (8409.08 to 11597.29)	122.36 (104.67 to 142.28)	-23.64% (-35.5% to -11.19%)	345.07 (290.35 to 401.34)	3.62 (3.09 to 4.21)	-20.78% (-32.67% to -7.05%)	0.887
**Massachusetts**	759.53 (631.27 to 915.03)	7.16 (6 to 8.52)	-5.84% (-20.41% to 12.62%)	12399.13 (10299.74 to 14737.1)	121.38 (101.83 to 143.06)	-23.54% (-36.75% to -8.91%)	487.27 (407.18 to 578.58)	4.05 (3.39 to 4.79)	-11.83% (-26.33% to 5.12%)	0.907
**Michigan**	1087.91 (926.13 to 1269.72)	7.37 (6.33 to 8.53)	-1.13% (-16.19% to 14.79%)	19945.49 (17186.55 to 23066.13)	144.51 (125.37 to 164.88)	-14.85% (-26.33% to -2.02%)	723.15 (618.15 to 837.42)	4.31 (3.72 to 4.97)	-8.83% (-21.45% to 5.63%)	0.863
**Minnesota**	615.17 (512.29 to 719.75)	7.84 (6.63 to 9.13)	-1.2% (-18.05% to 16.59%)	10384.67 (8675.63 to 12151.06)	135.87 (115.22 to 157.35)	-14.84% (-28.2% to -1.15%)	376.87 (312.99 to 442.82)	4.14 (3.47 to 4.85)	-10.2% (-24.67% to 5.5%)	0.886
**Mississippi**	329.45 (286.04 to 379.53)	8.27 (7.22 to 9.53)	15.31% (-0.51% to 34.73%)	7219.27 (6234.69 to 8334.5)	190.99 (166.57 to 218.22)	4.85% (-8.82% to 20.26%)	247.62 (211.82 to 286.37)	5.59 (4.83 to 6.43)	12.39% (-2.59% to 30.38%)	0.805
**Missouri**	651.66 (557.4 to 752.73)	7.57 (6.52 to 8.74)	6.22% (-10.29% to 24.03%)	12559.74 (10689.92 to 14584.6)	152.51 (130.91 to 175.56)	-6.28% (-20.62% to 8.5%)	449.74 (384.83 to 521.45)	4.57 (3.92 to 5.29)	0.46% (-14.45% to 16.53%)	0.844
**Montana**	117.41 (101.33 to 135.59)	7.47 (6.47 to 8.59)	10.43% (-5.27% to 28.74%)	2221.65 (1921.92 to 2575.68)	149.41 (131 to 172.5)	-1.2% (-14.66% to 14.87%)	80.73 (68.94 to 93.86)	4.37 (3.77 to 5.05)	2.83% (-11.83% to 18.94%)	0.856
**Nebraska**	225 (192.92 to 259.31)	8.48 (7.33 to 9.79)	6.4% (-9.96% to 23.21%)	4010.4 (3422.24 to 4614.39)	157.97 (136.3 to 181.23)	-6.97% (-20.95% to 8.06%)	145.54 (124.62 to 167.7)	4.83 (4.14 to 5.55)	0.43% (-14.31% to 16.03%)	0.862

© 2025 GBD 2021 US CNS Cancer Collaborators. *JAMA Neurology*.

**Nevada**	258.69 (221.47 to 298.64)	6.14 (5.25 to 7.01)	-2.03% (-16.05% to 13.61%)	5017.97 (4270.03 to 5791.08)	122.27 (105.48 to 140.03)	-17.23% (-29.16% to -4.55%)	177.35 (150.53 to 205.76)	3.78 (3.21 to 4.36)	-9.48% (-22.91% to 4.97%)	0.835
**New Hampshire**	155.97 (131.01 to 181.13)	7.32 (6.25 to 8.5)	4.67% (-11.56% to 22.24%)	2721.21 (2316.43 to 3187.75)	131.15 (112.06 to 152.69)	-11.21% (-24.68% to 4.28%)	102.97 (87.2 to 119.74)	4.13 (3.5 to 4.8)	-5.77% (-20.29% to 10.74%)	0.898
**New Jersey**	1041.9 (886.76 to 1234.29)	7.86 (6.75 to 9.17)	-4.59% (-20.32% to 12.04%)	15117.62 (12809.1 to 17713.22)	115.83 (99.03 to 134.88)	-22.84% (-34.72% to -10.03%)	553.34 (464.85 to 648.87)	3.63 (3.07 to 4.25)	-12.25% (-25.75% to 2.38%)	0.892
**New Mexico**	187.84 (159.85 to 220.23)	6.49 (5.54 to 7.55)	20.2% (0.31% to 41.59%)	3851.21 (3292.56 to 4506.07)	140.18 (120.51 to 162.98)	6.32% (-9.7% to 24.49%)	131.7 (111.89 to 154.4)	3.99 (3.4 to 4.68)	12.96% (-4.28% to 32.98%)	0.826
**New York**	1634.52 (1363.18 to 1896.4)	5.77 (4.9 to 6.61)	-3.95% (-17.61% to 11.62%)	29165.78 (24989.81 to 33942.49)	107.08 (92.7 to 123.05)	-25.71% (-35.88% to -14.22%)	1064.05 (889.34 to 1243.64)	3.28 (2.78 to 3.81)	-16.71% (-28.68% to -2.53%)	0.884
**North Carolina**	986 (841.1 to 1133.65)	6.75 (5.83 to 7.73)	-2.29% (-16.41% to 13.32%)	19069.23 (16258.53 to 22105.39)	136.16 (117.31 to 156.27)	-17.4% (-29.19% to -4.57%)	686.44 (583.68 to 797.34)	4.14 (3.53 to 4.78)	-11.71% (-24.77% to 2.24%)	0.838
**North Dakota**	77.91 (66.43 to 89.53)	7.63 (6.62 to 8.72)	0.22% (-14.58% to 17.22%)	1448.59 (1250.01 to 1651.14)	149.37 (129.48 to 169.48)	-8.39% (-20.47% to 5.77%)	49.93 (42.66 to 57.53)	4.24 (3.62 to 4.86)	-9.76% (-22.63% to 4.27%)	0.876
**Ohio**	1233.82 (1057.31 to 1415.4)	7.28 (6.26 to 8.36)	4.62% (-10.38% to 20.75%)	23938.82 (20681.47 to 27426.39)	148.18 (129.22 to 168.38)	-6.58% (-18.03% to 6.62%)	875.23 (752.41 to 1002.29)	4.53 (3.91 to 5.17)	1.92% (-11.44% to 16.96%)	0.846
**Oklahoma**	384.58 (332.27 to 439.83)	7.23 (6.25 to 8.22)	1.98% (-12.44% to 17.19%)	7986.77 (6975.08 to 9163)	157.67 (138.54 to 178.51)	-2.24% (-14.6% to 11.19%)	277.78 (241.11 to 322.28)	4.7 (4.1 to 5.41)	2.38% (-11.06% to 17.15%)	0.827
**Oregon**	477.2 (403.52 to 570.51)	7.63 (6.48 to 8.96)	-6.62% (-20.8% to 10.65%)	8370.64 (7216.96 to 9925.13)	139.39 (120.78 to 163.47)	-19.94% (-31.2% to -5.79%)	311.07 (263.81 to 368.72)	4.28 (3.67 to 5.05)	-14.28% (-26.75% to 1.14%)	0.868
**Pennsylvania**	1332.71 (1132.28 to 1555.43)	6.99 (6 to 8.03)	-2.95% (-16.64% to 11.87%)	24365.97 (20850.12 to 28080.39)	134.86 (116.92 to 153.91)	-17.31% (-28.41% to -5.28%)	910.51 (773.63 to 1056.32)	4.1 (3.52 to 4.73)	-11.06% (-23.71% to 2.53%)	0.87
**Rhode Island**	111.8 (93.65 to 130.9)	6.83 (5.75 to 7.93)	-9.49% (-24.61% to 7.29%)	1942.7 (1642.19 to 2285.1)	124.2 (104.76 to 145.85)	-24.7% (-37.61% to -10.8%)	74.87 (63.27 to 88.05)	3.96 (3.37 to 4.65)	-18.17% (-31.43% to -3.07%)	0.882
**South Carolina**	550.04 (466.96 to 624.86)	7.51 (6.42 to 8.55)	5.83% (-8.85% to 22.18%)	11109.62 (9461.01 to 12826.51)	159.68 (137.93 to 182.62)	-11.16% (-22.98% to 2.92%)	396.86 (336.8 to 455.84)	4.77 (4.08 to 5.46)	-5.08% (-19.19% to 9.53%)	0.832
**South Dakota**	98.02 (85.47 to 111.68)	8.2 (7.2 to 9.28)	5.83% (-9.62% to 21.34%)	1882.96 (1652.59 to 2151.85)	166.73 (147.66 to 188.62)	-4.01% (-16.42% to 9.54%)	66.38 (57.98 to 76.28)	4.82 (4.24 to 5.51)	0.49% (-13.29% to 15.36%)	0.85
**Tennessee**	749.94 (644.39 to 868.91)	7.82 (6.78 to 8.99)	4.05% (-10.38% to 19.87%)	15222.68 (13134.04 to 17647.11)	165.44 (144.36 to 190.32)	-8.01% (-20.46% to 5.89%)	532.38 (457.98 to 619.03)	4.92 (4.25 to 5.68)	-3.73% (-17.06% to 11.01%)	0.827
**Texas**	2391.76 (2093.89 to 2712.52)	6.46 (5.67 to 7.32)	-9.25% (-20.86% to 4.57%)	45797.2 (40265.43 to 52077.42)	125.75 (111.4 to 141.99)	-23.7% (-32.75% to -13.49%)	1604.18 (1403.74 to 1822.03)	3.97 (3.49 to 4.5)	-15.48% (-25.61% to -3.85%)	0.835

© 2025 GBD 2021 US CNS Cancer Collaborators. *JAMA Neurology*.

**Utah**	255.49 (219.38 to 295.14)	6.79 (5.85 to 7.84)	2.95% (-12.6% to 20.36%)	4742.85 (4125.22 to 5436.38)	127.97 (111.6 to 145.88)	-13.54% (-24.83% to -0.9%)	160.82 (138.23 to 186.08)	4.06 (3.5 to 4.69)	-2.74% (-16.17% to 11.84%)	0.851
**Vermont**	70.1 (60.97 to 79.92)	7.21 (6.31 to 8.18)	7.34% (-6.71% to 22.79%)	1248.3 (1102.58 to 1425.51)	134.51 (120.29 to 152.18)	-10.34% (-20.66% to 1.62%)	46.74 (40.96 to 53.18)	4.04 (3.55 to 4.59)	-8.87% (-19.78% to 3.77%)	0.889
**Virginia**	795.77 (675.35 to 932.51)	6.76 (5.79 to 7.9)	1.22% (-14.5% to 19.09%)	14572.7 (12356.32 to 17082.83)	127.22 (108.47 to 147.46)	-15.64% (-27.74% to -2.13%)	520.14 (439.82 to 607.38)	3.89 (3.33 to 4.52)	-10.21% (-23.22% to 4.85%)	0.877
**Washington**	783.06 (661.33 to 912.26)	7.15 (6.1 to 8.24)	-6.85% (-21.17% to 8.27%)	14588.27 (12284.27 to 16899.23)	139.19 (117.82 to 159.54)	-19.71% (-32.53% to -7.22%)	528.58 (446.54 to 611.98)	4.3 (3.63 to 4.96)	-14.16% (-27.5% to 0.2%)	0.876
**West Virginia**	208.19 (179.15 to 241.45)	7.81 (6.8 to 9.03)	24.47% (6.71% to 44.56%)	4285.35 (3696.15 to 4948)	171.5 (148.81 to 197.7)	16.12% (0.32% to 34.27%)	153.77 (132.29 to 177.74)	5.01 (4.34 to 5.79)	19.21% (2.23% to 38.8%)	0.812
**Wisconsin**	644.43 (539.76 to 757.27)	7.73 (6.49 to 8.98)	-0.93% (-17.26% to 16.45%)	11553.83 (9837.41 to 13515.33)	143.82 (123.71 to 166.72)	-11.52% (-24.62% to 3.03%)	421.84 (357.27 to 493.42)	4.36 (3.71 to 5.11)	-5.96% (-19.87% to 11.32%)	0.869
**Wyoming**	57.03 (49.9 to 64.12)	7.14 (6.25 to 8.03)	4.6% (-9.11% to 19.72%)	1093.84 (973.53 to 1231.17)	142.15 (127.18 to 159.27)	-7.68% (-17.94% to 4.49%)	38.84 (33.99 to 43.7)	4.3 (3.8 to 4.82)	-0.6% (-12.2% to 12.84%)	0.856
**Statistical Test for SDI**	Spearman's r: ρ = -0.0108 p-value = 0.9397	Spearman's r: ρ = -0.2683 p-value = 0.0547	Spearman's r: ρ = -0.5546 p-value < 0.0001	Spearman's r: ρ = -0.0680 p-value = 0.6312	Spearman's r: ρ = -0.6860 p-value < 0.0001	Spearman's r: ρ = -0.6282 p-value < 0.0001	Spearman's r: ρ = -0.0525 p-value = 0.7109	Spearman's r: ρ = -0.6391 p-value < 0.0001	Spearman's r: ρ = -0.6282, p-value < 0.0001	

© 2025 GBD 2021 US CNS Cancer Collaborators. *JAMA Neurology*.

eTable 7b: Correlation of Prevalence, YLD, YLL count and age-standardized rate of Brain and central nervous system cancer by state, 2021, and percentage change from 1990 to 2021 with SDI, United States

	Prevalence (95% UI)			YLDs (95% UI)			YLLs (95% UI)			
	Absolute number, 2021	Age-standardized rate, per 100 000 people, 2021	Percentage Change, 1990-2021	Absolute number, 2021	Age-standardized rate, per 100 000 people, 2021	Percentage Change, 1990-2021	Absolute number, 2021	Age-standardized rate, per 100 000 people, 2021	Percentage Change, 1990-2021	SDI
**United States of America**	84402.47 (80958.69 to 87130.86)	23.38 (22.43 to 24.28)	8.28% (4.11% to 12.56%)	11009.32 (7989.38 to 14200)	2.6 (1.92 to 3.4)	2.03% (-2.1% to 6.25%)	583986.83 (560205.4 to 599074.57)	131.78 (127.25 to 134.95)	-16.06% (-18.03% to -14%)	0.859
**Alabama**	1280.71 (1090.8 to 1467.92)	23.4 (20.08 to 26.85)	12.11% (-6.5% to 32.03%)	182.18 (125.17 to 247.38)	2.81 (1.96 to 3.84)	8.07% (-10.76% to 27.78%)	11919.02 (10286.49 to 13754.68)	177.36 (154.7 to 202.81)	-2.43% (-14.76% to 12.41%)	0.819
**Alaska**	206.34 (170.98 to 265.27)	28.9 (23.25 to 39.3)	55.88% (22.17% to 110.4%)	23.57 (16.8 to 32.52)	2.94 (2.07 to 4.36)	32.65% (6.52% to 72.42%)	1212.85 (1040.75 to 1417.68)	143.06 (121.65 to 173.3)	-0.58% (-16.15% to 21.73%)	0.851
**Arizona**	1879.58 (1614.77 to 2182.12)	24.48 (21.07 to 28.54)	18.03% (-1.02% to 41.36%)	240.58 (167.56 to 321.35)	2.66 (1.86 to 3.61)	11.28% (-7.73% to 35.78%)	12360.65 (10600.42 to 14236.6)	130.52 (113.17 to 149.8)	-8.96% (-21.22% to 4.29%)	0.842
**Arkansas**	772.76 (666.96 to 889.85)	24.13 (20.68 to 27.98)	7.64% (-10.2% to 27.54%)	106.37 (75.53 to 142.11)	2.82 (2 to 3.71)	3.7% (-14.58% to 25.31%)	6746.29 (5809.6 to 7776.21)	173.45 (150.62 to 198.45)	-5.57% (-18.77% to 8.88%)	0.811
**California**	9671.5 (8396.9 to 11279.52)	23.05 (19.86 to 26.87)	3.4% (-12.7% to 22.61%)	1180.55 (837.39 to 1540.8)	2.43 (1.73 to 3.23)	-3.36% (-19.04% to 15.27%)	60133.68 (52356.23 to 68113.03)	116.75 (103.33 to 131.44)	-22.46% (-31.81% to -12.43%)	0.87
**Colorado**	1566.44 (1308.11 to 1837.03)	24.77 (20.81 to 28.87)	9.2% (-9.41% to 30.08%)	196.16 (134.59 to 267.2)	2.69 (1.86 to 3.64)	3.88% (-15.4% to 28.55%)	9704.03 (8109.27 to 11317.12)	126.08 (107.21 to 145.48)	-16.46% (-29.8% to -3.14%)	0.877
**Connecticut**	1424.28 (1186.67 to 1723.56)	32.69 (27.42 to 38.97)	2% (-15.81% to 22.94%)	169.4 (116.14 to 228.6)	3.41 (2.36 to 4.52)	-2.12% (-21.08% to 20.37%)	6135.63 (5111.75 to 7220.7)	120.91 (100.82 to 140.72)	-18.44% (-31.61% to -4.41%)	0.902
**Delaware**	241.16 (209.75 to 274.82)	22.28 (19.4 to 25.45)	20.94% (2.46% to 42.31%)	32.52 (23.31 to 42.83)	2.5 (1.76 to 3.3)	9.92% (-8.32% to 31.58%)	1740.36 (1542.34 to 1954.64)	127.57 (114.14 to 142.32)	-13.26% (-23.64% to -2.57%)	0.863
**District of Columbia**	85 (72.26 to 98.78)	12.33 (10.39 to 14.37)	-4.8% (-20.68% to 12.93%)	12.35 (8.56 to 16.31)	1.57 (1.1 to 2.07)	-15.94% (-30.08% to 0.61%)	791.34 (676.76 to 912.28)	99.24 (85.67 to 113.89)	-43.53% (-52.01% to -34.23%)	0.886
**Florida**	4673.41 (4001.93 to 5420.73)	21.81 (18.65 to 25.3)	6.13% (-11.45% to 26.42%)	648.62 (422.72 to 891.9)	2.34 (1.59 to 3.23)	1.32% (-15.38% to 24.07%)	40376.49 (34475.39 to 46747.59)	132.49 (114.99 to 152.14)	-16.81% (-27.56% to -4.4%)	0.856
**Georgia**	2383.26 (2043.61 to 2779.76)	20.46 (17.57 to 23.94)	16.15% (-1.56% to 36.79%)	322.24 (219.91 to 435.15)	2.4 (1.66 to 3.22)	4.6% (-13.94% to 24.72%)	18216.07 (15784.37 to 20945.98)	132.13 (115.67 to 150.71)	-18.31% (-28.21% to -6.46%)	0.841
**Hawaii**	252.58 (214.96 to 289.48)	16.18 (13.74 to 18.72)	6.41% (-12.46% to 27.71%)	34.6 (24.46 to 46.16)	1.82 (1.3 to 2.42)	3.33% (-14.51% to 24.78%)	1731.1 (1468.56 to 2002.99)	87.1 (74.67 to 100.93)	-14.83% (-27.08% to -1.4%)	0.86

© 2025 GBD 2021 US CNS Cancer Collaborators. *JAMA Neurology*.

**Idaho**	494.72 (413.81 to 569.02)	24.29 (20.45 to 27.87)	7.15% (-11.81% to 27.08%)	63.77 (44.36 to 86.68)	2.74 (1.88 to 3.68)	3.82% (-17.09% to 25.43%)	3368.57 (2826.13 to 3936.39)	139.17 (118.2 to 161.06)	-12.15% (-25.63% to 2.49%)	0.827
**Illinois**	3119.08 (2686.51 to 3649.48)	22.87 (19.69 to 26.58)	16.31% (-1.06% to 36.44%)	406.84 (280.02 to 565.24)	2.53 (1.77 to 3.46)	6.44% (-11.24% to 27.96%)	20989.02 (17867.07 to 24218.27)	124.41 (107.63 to 142.16)	-19.04% (-30.29% to -7.11%)	0.872
**Indiana**	1733.55 (1487.55 to 1992.48)	23.95 (20.44 to 27.44)	7.97% (-10.41% to 26.92%)	229.51 (157.95 to 304.58)	2.7 (1.87 to 3.58)	3.82% (-14.39% to 24.18%)	13048.09 (11251.07 to 14990.81)	146.76 (128.19 to 167.25)	-7.5% (-20.09% to 6.31%)	0.838
**Iowa**	792.19 (668.39 to 926.68)	22.38 (18.79 to 26.27)	15.1% (-5.95% to 39.72%)	115.99 (80.23 to 162.79)	2.76 (1.93 to 3.85)	9.93% (-11.33% to 34.85%)	6354.77 (5368 to 7361.79)	148.91 (127.37 to 170.85)	-3.21% (-18.11% to 12.21%)	0.864
**Kansas**	872.41 (741.15 to 1015.93)	27.6 (23.55 to 32)	10.63% (-7.43% to 32.17%)	111.59 (78.44 to 146.82)	3.06 (2.15 to 4.01)	8.33% (-13.78% to 32.23%)	5953.54 (4987.99 to 6927.6)	156.14 (131.75 to 181.04)	-1.82% (-16.85% to 14.15%)	0.858
**Kentucky**	1453.94 (1231.46 to 1703.69)	28.67 (24.41 to 33.52)	6.43% (-12.38% to 28.36%)	190.79 (131.93 to 252.79)	3.29 (2.28 to 4.37)	4.36% (-14.89% to 27.62%)	9743.95 (8332.69 to 11164.82)	162.11 (139.09 to 184.79)	-3.01% (-16.67% to 11.64%)	0.815
**Louisiana**	1095.35 (944.84 to 1278.79)	22.07 (19.1 to 25.86)	17.19% (-0.65% to 39.28%)	156.54 (108.95 to 213.49)	2.73 (1.91 to 3.72)	11.97% (-7.89% to 35.88%)	9212.16 (7998.32 to 10650.81)	155.17 (135.26 to 177.27)	-2.46% (-15.76% to 12.28%)	0.823
**Maine**	402.87 (340.7 to 475.68)	25.74 (21.91 to 30.13)	11.8% (-6.81% to 35.2%)	56.44 (39.54 to 78.59)	2.91 (2.03 to 4.04)	10.26% (-10.75% to 35.13%)	3017.56 (2587.54 to 3516.03)	146.21 (126.07 to 169.77)	-2.25% (-17.62% to 13.82%)	0.862
**Maryland**	1372.39 (1175.91 to 1601.73)	20.83 (17.82 to 24.34)	6.34% (-11.37% to 27.43%)	180.28 (124.53 to 251.53)	2.33 (1.63 to 3.25)	-3.15% (-20.97% to 17.02%)	9745.63 (8219.68 to 11425.14)	120.03 (102.5 to 139.57)	-23.95% (-35.68% to -11.53%)	0.887
**Massachusetts**	2064.93 (1702.53 to 2506.22)	24.88 (20.88 to 29.62)	-0.3% (-17.44% to 20.76%)	267.99 (182.48 to 365.1)	2.75 (1.89 to 3.75)	-2.78% (-21.04% to 19.04%)	12131.14 (10084.34 to 14395.89)	118.63 (99.57 to 140.31)	-23.91% (-37% to -9.5%)	0.907
**Michigan**	2562.93 (2167.09 to 3016.29)	21.45 (18.35 to 25.16)	10.44% (-7.01% to 30.23%)	361.77 (253.23 to 493.8)	2.62 (1.85 to 3.57)	1.92% (-17.34% to 22.68%)	19583.72 (16892.98 to 22620.33)	141.89 (123.39 to 161.69)	-15.1% (-26.6% to -2.31%)	0.863
**Minnesota**	1870.96 (1576.67 to 2200.58)	30.3 (25.48 to 35.4)	11.51% (-9.57% to 33.11%)	226.59 (155.83 to 300.81)	3.14 (2.15 to 4.22)	4.19% (-16.7% to 26.99%)	10158.08 (8496.26 to 11852.83)	132.72 (112.72 to 153.84)	-15.21% (-28.5% to -1.4%)	0.886
**Mississippi**	734.43 (624.49 to 843.29)	23.41 (20.12 to 26.93)	18.71% (0.25% to 40.94%)	104.67 (69.08 to 139.5)	2.85 (1.93 to 3.79)	14.69% (-5.57% to 39.78%)	7114.6 (6165.26 to 8208.85)	188.15 (163.68 to 215.11)	4.71% (-8.86% to 20.14%)	0.805
**Missouri**	1643.8 (1402.97 to 1924.55)	24.83 (21.34 to 28.81)	14.07% (-3.85% to 36.18%)	219.51 (151.45 to 287.24)	2.79 (1.98 to 3.7)	8.17% (-10.81% to 33.33%)	12340.24 (10519.82 to 14322.49)	149.72 (128.69 to 172.19)	-6.51% (-20.91% to 8.29%)	0.844
**Montana**	297.3 (255.84 to 343.8)	25.58 (22.2 to 29.61)	20.08% (0.7% to 40.9%)	39.88 (28.38 to 53.7)	2.82 (2.03 to 3.89)	14.16% (-4.15% to 36.54%)	2181.77 (1883.12 to 2528.63)	146.59 (128.49 to 168.97)	-1.46% (-14.92% to 14.45%)	0.856
**Nebraska**	627.83 (534.99 to 726.83)	29.85 (25.56 to 34.51)	12.55% (-6.65% to 35.13%)	78.46 (55.4 to 105.49)	3.23 (2.27 to 4.33)	9.18% (-9.66% to 32.68%)	3931.94 (3355.24 to 4518.18)	154.74 (133.5 to 177.77)	-7.26% (-21.2% to 7.79%)	0.862

© 2025 GBD 2021 US CNS Cancer Collaborators. *JAMA Neurology*.

**Nevada**	663.96 (571.39 to 760.45)	19.6 (16.79 to 22.59)	9.1% (-7.98% to 28.21%)	89.91 (62.68 to 118.33)	2.28 (1.6 to 2.98)	2.73% (-13.06% to 22.71%)	4928.06 (4199.28 to 5701.43)	120 (103.48 to 137.53)	-17.54% (-29.4% to -4.84%)	0.835
**New Hampshire**	405.84 (340.41 to 471.04)	25.61 (21.68 to 29.76)	17.75% (-2.04% to 40.53%)	54.25 (37.11 to 72.9)	2.81 (1.94 to 3.78)	10.71% (-8.63% to 32.66%)	2666.96 (2271.69 to 3115.01)	128.33 (109.68 to 149.24)	-11.6% (-25.06% to 3.81%)	0.898
**New Jersey**	4175.9 (3505.97 to 4905.06)	37 (31.61 to 43.37)	5.32% (-13.62% to 25.46%)	450.11 (312.91 to 616.36)	3.58 (2.52 to 4.95)	1.35% (-19.03% to 24.06%)	14667.51 (12435.62 to 17263.5)	112.24 (96.03 to 130.73)	-23.43% (-35.3% to -10.77%)	0.892
**New Mexico**	522.7 (441.4 to 611.27)	22.66 (19.42 to 26.7)	28.31% (5.04% to 54.72%)	68.03 (47.42 to 91.48)	2.53 (1.73 to 3.41)	24.6% (0.78% to 49.03%)	3783.18 (3234.44 to 4419.67)	137.65 (118.21 to 160.04)	6.04% (-9.97% to 24.14%)	0.826
**New York**	4468.61 (3852.92 to 5232.51)	20.43 (17.56 to 23.61)	14.81% (-1.98% to 36.17%)	581.26 (418.56 to 775.69)	2.23 (1.59 to 3)	3.7% (-14.2% to 25.39%)	28584.52 (24442.5 to 33250.81)	104.85 (90.95 to 120.5)	-26.16% (-36.25% to -14.75%)	0.884
**North Carolina**	2462.21 (2112.97 to 2845.11)	21.77 (18.81 to 24.9)	13.36% (-3.95% to 33.86%)	331.26 (233.53 to 444.09)	2.48 (1.75 to 3.32)	3.07% (-14.54% to 23.6%)	18737.97 (15995.71 to 21676.21)	133.68 (115.11 to 153.31)	-17.7% (-29.55% to -4.93%)	0.838
**North Dakota**	225.83 (196.15 to 261.5)	28.1 (24.21 to 32.88)	14.82% (-3.32% to 37.09%)	27.59 (18.78 to 36.24)	2.95 (2.02 to 3.91)	5.91% (-14.74% to 29.79%)	1421 (1227.1 to 1621.02)	146.42 (127.34 to 166.15)	-8.64% (-20.74% to 5.47%)	0.876
**Ohio**	2948.27 (2531.56 to 3403.96)	22.85 (19.7 to 26.29)	6.87% (-9.3% to 26.73%)	407.32 (289.98 to 543.8)	2.64 (1.86 to 3.51)	4.83% (-12.61% to 24.92%)	23531.51 (20303.29 to 26956.72)	145.54 (126.82 to 165.44)	-6.76% (-18.2% to 6.36%)	0.846
**Oklahoma**	911.01 (792.05 to 1041.48)	21.48 (18.61 to 24.43)	0.54% (-15.65% to 18.48%)	126.47 (87.78 to 173.35)	2.56 (1.77 to 3.46)	0.64% (-17.47% to 20.73%)	7860.3 (6849.26 to 9000.76)	155.1 (136.31 to 175.83)	-2.29% (-14.56% to 11.18%)	0.827
**Oregon**	1303.25 (1108.22 to 1548.32)	27.43 (23.32 to 32.18)	3.76% (-14.01% to 24.44%)	168.31 (118.6 to 234.9)	2.97 (2.08 to 4.13)	-0.79% (-17.66% to 23.31%)	8202.34 (7070.87 to 9685.88)	136.43 (118.49 to 159.74)	-20.28% (-31.48% to -6.26%)	0.868
**Pennsylvania**	3360.97 (2888.35 to 3856.81)	23.79 (20.76 to 27.26)	9.38% (-7.91% to 28.8%)	450.47 (305.63 to 621.34)	2.62 (1.79 to 3.68)	1.48% (-16.41% to 23.23%)	23915.49 (20452.68 to 27583.01)	132.23 (114.69 to 150.83)	-17.61% (-28.67% to -5.54%)	0.87
**Rhode Island**	287.03 (238.14 to 340.92)	23.44 (19.54 to 27.85)	3.17% (-15.35% to 22.12%)	38.41 (27 to 53.36)	2.58 (1.84 to 3.58)	-3.81% (-22.38% to 19.44%)	1904.29 (1609.2 to 2238.97)	121.62 (102.61 to 142.53)	-25.04% (-37.98% to -11.11%)	0.882
**South Carolina**	1296.11 (1101.8 to 1482.11)	23.1 (19.9 to 26.22)	23.68% (2.93% to 45.27%)	181.04 (125.08 to 245.41)	2.7 (1.89 to 3.66)	12.35% (-8.84% to 34.03%)	10928.57 (9323.01 to 12602.18)	156.98 (135.9 to 179.07)	-11.48% (-23.33% to 2.65%)	0.832
**South Dakota**	259.19 (226.2 to 294.17)	28.05 (24.25 to 31.89)	11.62% (-6.93% to 31.79%)	33.43 (24.12 to 44.15)	3.08 (2.24 to 4.05)	7.95% (-10.8% to 29.26%)	1849.53 (1625.29 to 2114.59)	163.65 (145.07 to 185.17)	-4.21% (-16.52% to 9.39%)	0.85
**Tennessee**	1814.54 (1566.9 to 2121.42)	24.2 (20.9 to 28.07)	15.6% (-2.41% to 36.07%)	247.85 (177.79 to 331.14)	2.81 (2.02 to 3.74)	8.54% (-10.86% to 30.17%)	14974.83 (12904.03 to 17356.79)	162.63 (142.02 to 187)	-8.25% (-20.72% to 5.71%)	0.827
**Texas**	6464.01 (5636.98 to 7368.23)	20.77 (18.18 to 23.68)	0.02% (-13.63% to 16.95%)	831.99 (587.66 to 1108.53)	2.38 (1.68 to 3.14)	-5.99% (-21.89% to 12.6%)	44965.2 (39596.46 to 51181.17)	123.37 (109.4 to 139.19)	-23.98% (-32.97% to -13.77%)	0.835

© 2025 GBD 2021 US CNS Cancer Collaborators. *JAMA Neurology*.

**Utah**	763.66 (661.9 to 879.88)	22.01 (19.09 to 25.41)	6.54% (-10.82% to 25.99%)	92.09 (65.03 to 123.28)	2.52 (1.77 to 3.37)	4.11% (-13.78% to 26.37%)	4650.76 (4047.62 to 5343.19)	125.45 (109.61 to 143.62)	-13.83% (-25.19% to -1.2%)	0.851
**Vermont**	181.55 (158.82 to 207.76)	25.75 (22.34 to 29.52)	31.79% (12.52% to 54.41%)	24.12 (17.5 to 31.72)	2.78 (1.95 to 3.79)	17.56% (-2.97% to 40.76%)	1224.18 (1082.89 to 1401.83)	131.73 (118.06 to 148.91)	-10.79% (-20.94% to 1.07%)	0.889
**Virginia**	2195.44 (1846.9 to 2572.26)	23.65 (20.02 to 27.78)	18.55% (-1.14% to 41.72%)	282.39 (200.2 to 382.49)	2.59 (1.82 to 3.51)	8.13% (-10.68% to 31.3%)	14290.32 (12145.34 to 16707.33)	124.63 (106.23 to 144.3)	-16.03% (-28.06% to -2.45%)	0.877
**Washington**	1758.59 (1500.56 to 2045.82)	20.51 (17.69 to 23.73)	4.49% (-11.54% to 22.72%)	251.79 (170.44 to 337.03)	2.49 (1.7 to 3.34)	-4.32% (-21.5% to 16.23%)	14336.48 (12039.22 to 16602.81)	136.7 (115.29 to 156.65)	-19.95% (-32.71% to -7.46%)	0.876
**West Virginia**	456 (394.07 to 531.9)	23.21 (20 to 26.84)	30.14% (9.96% to 54.12%)	66.69 (46.97 to 89.93)	2.78 (1.95 to 3.71)	25.79% (4.48% to 53.39%)	4218.67 (3650.56 to 4876.82)	168.72 (146.5 to 194.48)	15.97% (0.13% to 34.18%)	0.812
**Wisconsin**	1755.74 (1458.28 to 2034.49)	27.6 (23.3 to 31.87)	5.35% (-13.71% to 25.77%)	225.29 (159.99 to 303.78)	2.97 (2.11 to 4.08)	1.36% (-18.51% to 21.69%)	11328.55 (9639.44 to 13294.57)	140.85 (121.09 to 162.61)	-11.76% (-24.77% to 2.97%)	0.869
**Wyoming**	146.36 (126.85 to 165.11)	23.24 (20.06 to 26.35)	8.84% (-8.87% to 27.17%)	19.52 (13.82 to 25.87)	2.65 (1.87 to 3.55)	7.03% (-10.37% to 26.07%)	1074.33 (954.83 to 1210.49)	139.5 (124.67 to 156.52)	-7.92% (-18.12% to 4.33%)	0.856
**Statistical Test for SDI**	Spearman's r: ρ = 0.0455 p-value = 0.7481	Spearman's r: ρ = 0.1725 p-value = 0.2208	Spearman's r: ρ = -0.2634, p-value = 0.0595	Spearman's r: ρ = -0.0680 p-value = 0.6312	Spearman's r: ρ = -0.0412 p-value = 0.7711	Spearman's r: ρ = -0.6282 p-value < 0.0001	Spearman's r: ρ = -0.0644 p-value = 0.6494	Spearman's r: ρ = -0.6929 p-value < 0.0001	Spearman's r: ρ = -0.6296 p-value < 0.0001	

© 2025 GBD 2021 US CNS Cancer Collaborators. *JAMA Neurology*.

eTable 8: Correlation of age-standardized incidence, DALYs, death, prevalence, YLD, YLL rate of Brain and central nervous system cancer by state with SDI related factors, 2021, United States

	Fertility rate under the age of 25, 2021	Achievement of high school graduate or higher (18 years and over)	Income per capita
**Alabama**	47	88.290%	30,608
**Alaska**	52	92.838%	39,509
**Arizona**	50	90.801%	36,295
**Arkansas**	53	89.857%	29,252
**California**	49	89.526%	42,396
**Colorado**	44	93.615%	44,617
**Connecticut**	47	92.433%	48,146
**Delaware**	52	92.010%	38,797
**District of Columbia**	39	93.876%	65,808
**Florida**	45	90.972%	36,196
**Georgia**	52	89.841%	35,086
**Hawaii**	59	93.970%	38,614
**Idaho**	53	92.244%	33,841
**Illinois**	50	91.868%	39,794
**Indiana**	55	90.827%	33,054
**Iowa**	53	93.661%	35,715
**Kansas**	59	92.897%	35,028
**Kentucky**	52	88.732%	30,728
**Louisiana**	55	86.993%	30,117
**Maine**	54	94.291%	38,483
**Maryland**	53	92.629%	46,500
**Massachusetts**	45	92.369%	49,746
**Michigan**	52	91.951%	35,353
**Minnesota**	54	94.427%	41,753
**Mississippi**	53	86.871%	26,941
**Missouri**	52	91.527%	34,593
**Montana**	51	93.896%	36,020
**Nebraska**	62	93.357%	36,227
**Nevada**	51	90.163%	34,933
**New Hampshire**	46	94.361%	45,365
**New Jersey**	53	92.719%	47,338
**New Mexico**	49	88.889%	31,043
**New York**	46	90.318%	43,078
**North Carolina**	48	91.195%	35,254
**North Dakota**	67	93.466%	36,497
**Ohio**	51	91.673%	35,119
**Oklahoma**	53	89.506%	29,969
**Oregon**	46	92.903%	38,975
**Pennsylvania**	50	92.105%	38,315
**Rhode Island**	46	90.829%	40,382
**South Carolina**	51	89.993%	33,339
**South Dakota**	58	92.404%	35,135
**Tennessee**	53	90.577%	33,904
**Texas**	60	89.397%	34,717
**Utah**	58	93.720%	35,220
**Vermont**	41	94.217%	40,016
**Virginia**	47	92.476%	43,756
**Washington**	50	93.521%	46,177
**West Virginia**	48	88.888%	30,195
**Wisconsin**	49	93.582%	37,221
**Wyoming**	56	93.638%	37,156
**Statistical Test (Spearman's r)**	Incidence: ρ = 0.2556 p-value = 0.0705<br>DALY: ρ = 0.6797 p-value < 0.0001<br>Death: ρ = 0.5909 p-value < 0.0001<br>Prevalence: ρ = -0.1765 p-value = 0.2148<br>YLD: ρ = 0.0286 p-value = 0.8418<br>YLL: ρ = 0.6851 p-value < 0.0001	Incidence: ρ = -0.0061 p-value = 0.9664<br>DALY: ρ = -0.2843 p-value = 0.0436<br>Death: ρ = -0.2557 p-value = 0.0704<br>Prevalence: ρ = 0.3139 p-value = 0.0253<br>YLD: ρ = 0.1989 p-value = 0.1613<br>YLL: ρ = -0.2915 p-value = 0.0383	Incidence: ρ = -0.3527 p-value = 0.0115<br>DALY: ρ = -0.7474 p-value < 0.0001<br>Death: ρ = -0.7240 p-value < 0.0001<br>Prevalence: ρ = 0.1348 p-value = 0.3444<br>YLD: ρ = -0.1031 p-value = 0.4705<br>YLL: ρ = -0.7519 p-value < 0.0001

© 2025 GBD 2021 US CNS Cancer Collaborators. *JAMA Neurology*.

© 2025 GBD 2021 US CNS Cancer Collaborators. *JAMA Neurology*.

eTable 9a: Age-standardized incidence rate of Brain and central nervous system cancer by state and year, and percentage change from 1990 to 2021, United States

	Percentage Change, 1990-2021	1990	1995	2000	2005	2010	2015	2020	2021
**United States of America**	-1.45% (-4.41% to 0.91%)	7.01 (6.83 to 7.14)	7.15 (6.98 to 7.28)	7.43 (7.18 to 7.59)	7.12 (6.87 to 7.26)	7.06 (6.81 to 7.22)	7.21 (6.95 to 7.37)	6.93 (6.64 to 7.1)	6.91 (6.58 to 7.12)
**Alabama**	8.51% (-7.36% to 25.85%)	7.51 (7.12 to 7.89)	8.02 (7.62 to 8.46)	8.26 (7.76 to 8.73)	7.94 (7.49 to 8.37)	8.67 (8.12 to 9.17)	8.64 (8.13 to 9.1)	8.15 (7.56 to 8.74)	8.15 (7.05 to 9.33)
**Alaska**	18.53% (-0.7% to 47.07%)	6.23 (5.85 to 6.67)	6.43 (6.02 to 6.85)	6.74 (6.23 to 7.26)	6.72 (6.23 to 7.25)	7.05 (6.58 to 7.57)	7.07 (6.6 to 7.59)	7.63 (6.91 to 8.6)	7.38 (6.29 to 9.24)
**Arizona**	8.88% (-6.21% to 26.03%)	6.4 (6.01 to 6.78)	6.68 (6.25 to 7.07)	6.98 (6.54 to 7.44)	7 (6.59 to 7.47)	6.74 (6.3 to 7.2)	6.76 (6.3 to 7.22)	7 (6.39 to 7.64)	6.97 (6.02 to 8.01)
**Arkansas**	3.75% (-11.37% to 19.38%)	7.73 (7.31 to 8.15)	7.93 (7.52 to 8.4)	8.3 (7.83 to 8.84)	7.7 (7.24 to 8.14)	7.96 (7.51 to 8.44)	8.33 (7.87 to 8.89)	7.97 (7.33 to 8.69)	8.02 (6.95 to 9.18)
**California**	-6.16% (-18.7% to 7.46%)	6.55 (6.21 to 6.87)	6.65 (6.33 to 6.98)	6.92 (6.56 to 7.26)	6.36 (6.04 to 6.66)	6.01 (5.67 to 6.36)	6.33 (5.89 to 6.73)	6.13 (5.58 to 6.67)	6.15 (5.42 to 7.02)
**Colorado**	-1.08% (-17.16% to 15.51%)	7.08 (6.62 to 7.51)	7.35 (6.89 to 7.76)	7.37 (6.91 to 7.85)	7.09 (6.62 to 7.56)	7.2 (6.67 to 7.7)	7.14 (6.62 to 7.59)	6.97 (6.4 to 7.65)	7 (5.88 to 8.18)
**Connecticut**	-3.87% (-19.98% to 14.96%)	8.63 (8.06 to 9.28)	8.81 (8.18 to 9.44)	9 (8.4 to 9.68)	8.82 (8.2 to 9.46)	8.91 (8.23 to 9.57)	9.03 (8.38 to 9.74)	8.5 (7.6 to 9.4)	8.3 (6.93 to 9.77)
**Delaware**	1.63% (-10.61% to 14.82%)	6.45 (6.05 to 6.83)	6.47 (6.07 to 6.89)	6.99 (6.52 to 7.44)	6.58 (6.17 to 7)	6.76 (6.32 to 7.23)	6.67 (6.21 to 7.1)	6.53 (6.07 to 7.05)	6.56 (5.78 to 7.36)
**District of Columbia**	-23.7% (-36.04% to -10.9%)	5.72 (5.37 to 6.08)	5.34 (5 to 5.68)	5.16 (4.82 to 5.51)	4.85 (4.49 to 5.22)	4.22 (3.93 to 4.56)	4.09 (3.79 to 4.39)	4.42 (4.01 to 4.87)	4.36 (3.76 to 5.02)
**Florida**	-1.4% (-16.34% to 15.53%)	6.34 (5.97 to 6.67)	6.65 (6.34 to 6.97)	6.99 (6.63 to 7.33)	6.56 (6.19 to 6.92)	6.35 (5.95 to 6.71)	6.37 (5.97 to 6.75)	6.27 (5.78 to 6.71)	6.25 (5.38 to 7.3)
**Georgia**	-0.94% (-13.4% to 15.11%)	6.69 (6.36 to 7.02)	6.78 (6.36 to 7.19)	6.88 (6.49 to 7.29)	6.62 (6.24 to 7.01)	6.82 (6.42 to 7.21)	6.82 (6.42 to 7.22)	6.61 (6.08 to 7.15)	6.63 (5.75 to 7.6)
**Hawaii**	0.44% (-15.36% to 16.65%)	4.68 (4.36 to 5.02)	4.97 (4.61 to 5.34)	4.97 (4.6 to 5.33)	5.03 (4.65 to 5.4)	5 (4.66 to 5.36)	5.05 (4.66 to 5.44)	4.83 (4.48 to 5.23)	4.7 (4.04 to 5.38)
**Idaho**	2.07% (-14.3% to 17.97%)	7.26 (6.78 to 7.8)	7.58 (7.09 to 8.09)	7.93 (7.34 to 8.45)	7.64 (7.14 to 8.1)	7.74 (7.2 to 8.25)	7.8 (7.25 to 8.34)	7.39 (6.7 to 7.96)	7.41 (6.22 to 8.51)
**Illinois**	-0.73% (-14.83% to 14.85%)	6.66 (6.32 to 7.05)	6.69 (6.33 to 7.06)	7 (6.6 to 7.39)	6.5 (6.12 to 6.88)	6.76 (6.3 to 7.17)	6.88 (6.43 to 7.33)	6.59 (5.99 to 7.2)	6.61 (5.67 to 7.63)
**Indiana**	3.6% (-11.72% to 19.33%)	7.14 (6.77 to 7.53)	7.56 (7.08 to 8)	7.73 (7.27 to 8.21)	7.5 (7.04 to 7.97)	7.41 (6.96 to 7.86)	7.83 (7.38 to 8.29)	7.43 (6.77 to 8.04)	7.4 (6.43 to 8.46)
**Iowa**	10.1% (-7.45% to 30.95%)	7.36 (6.9 to 7.84)	7.94 (7.44 to 8.47)	8.39 (7.78 to 8.94)	8.24 (7.69 to 8.87)	8.38 (7.71 to 8.97)	8.5 (7.89 to 9.08)	8.42 (7.71 to 9.16)	8.1 (6.91 to 9.31)
**Kansas**	8.55% (-8.11% to 27.71%)	7.56 (7.09 to 8.08)	8.06 (7.52 to 8.58)	8.27 (7.77 to 8.84)	8.4 (7.88 to 8.94)	8.33 (7.83 to 8.87)	8.39 (7.87 to 8.94)	8.27 (7.54 to 9.01)	8.21 (6.96 to 9.48)
**Kentucky**	4.12% (-10.6% to 22.22%)	8.58 (8.04 to 9.1)	8.89 (8.29 to 9.48)	9.16 (8.52 to 9.73)	8.6 (8.07 to 9.22)	9.18 (8.58 to 9.83)	9.35 (8.7 to 10)	8.96 (8.19 to 9.68)	8.94 (7.69 to 10.3)
**Louisiana**	11.74% (-3.52% to 29.91%)	7.16 (6.76 to 7.59)	7.41 (6.98 to 7.95)	8.05 (7.57 to 8.56)	8.73 (8.2 to 9.33)	7.95 (7.45 to 8.44)	8.15 (7.66 to 8.64)	7.93 (7.28 to 8.65)	8 (6.91 to 9.28)
**Maine**	7.85% (-8.56% to 26.84%)	7.19 (6.72 to 7.72)	7.48 (6.94 to 8.09)	8.14 (7.52 to 8.79)	8.01 (7.44 to 8.64)	7.81 (7.25 to 8.39)	7.94 (7.34 to 8.53)	7.75 (7.08 to 8.4)	7.75 (6.62 to 9.06)
**Maryland**	-10.53% (-24.45% to 5.36%)	6.87 (6.46 to 7.23)	6.67 (6.28 to 7.07)	6.74 (6.3 to 7.17)	6.31 (5.88 to 6.73)	6.27 (5.88 to 6.68)	6.57 (6.14 to 7)	6.16 (5.65 to 6.72)	6.14 (5.25 to 7.18)

© 2025 GBD 2021 US CNS Cancer Collaborators. *JAMA Neurology*.

**Massachusetts**	-5.84% (-20.41% to 12.62%)	7.61 (7.17 to 8.05)	7.61 (7.09 to 8.06)	8.54 (8.01 to 9.09)	7.89 (7.34 to 8.44)	7.77 (7.21 to 8.38)	8.64 (8.01 to 9.25)	7.43 (6.66 to 8.22)	7.16 (6 to 8.52)
**Michigan**	-1.13% (-16.19% to 14.79%)	7.46 (7.02 to 7.88)	7.39 (6.95 to 7.85)	7.76 (7.31 to 8.24)	7.68 (7.21 to 8.18)	7.79 (7.3 to 8.31)	7.65 (7.17 to 8.15)	7.39 (6.81 to 8.05)	7.37 (6.33 to 8.53)
**Minnesota**	-1.2% (-18.05% to 16.59%)	7.93 (7.42 to 8.44)	8.12 (7.55 to 8.66)	8.84 (8.29 to 9.4)	7.71 (7.16 to 8.23)	8.22 (7.59 to 8.85)	8.29 (7.65 to 8.83)	7.95 (7.19 to 8.71)	7.84 (6.63 to 9.13)
**Mississippi**	15.31% (-0.51% to 34.73%)	7.18 (6.79 to 7.58)	7.72 (7.3 to 8.17)	8.27 (7.8 to 8.7)	7.9 (7.46 to 8.36)	7.7 (7.22 to 8.13)	8.08 (7.62 to 8.49)	8.26 (7.66 to 8.9)	8.27 (7.22 to 9.53)
**Missouri**	6.22% (-10.29% to 24.03%)	7.13 (6.71 to 7.56)	7.48 (7.07 to 7.93)	7.82 (7.38 to 8.29)	7.52 (7.08 to 7.95)	7.47 (7.04 to 7.93)	7.48 (7.01 to 7.92)	7.55 (6.96 to 8.19)	7.57 (6.52 to 8.74)
**Montana**	10.43% (-5.27% to 28.74%)	6.76 (6.32 to 7.24)	7.12 (6.67 to 7.62)	7.82 (7.33 to 8.36)	7.67 (7.16 to 8.21)	7.96 (7.39 to 8.59)	8.04 (7.46 to 8.66)	7.53 (6.86 to 8.16)	7.47 (6.47 to 8.59)
**Nebraska**	6.4% (-9.96% to 23.21%)	7.97 (7.46 to 8.51)	8.28 (7.7 to 8.9)	8.86 (8.23 to 9.54)	8.36 (7.81 to 8.97)	8.47 (7.81 to 9.14)	8.59 (7.95 to 9.25)	8.52 (7.83 to 9.23)	8.48 (7.33 to 9.79)
**Nevada**	-2.03% (-16.05% to 13.61%)	6.26 (5.87 to 6.65)	6.47 (6.08 to 6.86)	6.69 (6.25 to 7.1)	6.86 (6.41 to 7.29)	6.64 (6.18 to 7.12)	6.74 (6.27 to 7.16)	6.16 (5.64 to 6.66)	6.14 (5.25 to 7.01)
**New Hampshire**	4.67% (-11.56% to 22.24%)	6.99 (6.53 to 7.49)	7.42 (6.9 to 7.93)	7.63 (7.11 to 8.15)	7.78 (7.23 to 8.34)	7.59 (7.08 to 8.2)	8.26 (7.63 to 8.92)	7.42 (6.8 to 8.04)	7.32 (6.25 to 8.5)
**New Jersey**	-4.59% (-20.32% to 12.04%)	8.24 (7.66 to 8.89)	8.55 (7.98 to 9.19)	8.78 (8.18 to 9.37)	8.4 (7.89 to 8.93)	8.21 (7.65 to 8.75)	8.54 (7.92 to 9.17)	8.07 (7.25 to 8.91)	7.86 (6.75 to 9.17)
**New Mexico**	20.2% (0.31% to 41.59%)	5.4 (5.03 to 5.78)	5.66 (5.26 to 6.07)	5.8 (5.4 to 6.24)	5.82 (5.42 to 6.24)	5.93 (5.53 to 6.36)	6.24 (5.75 to 6.67)	6.43 (5.86 to 7.06)	6.49 (5.54 to 7.55)
**New York**	-3.95% (-17.61% to 11.62%)	6.01 (5.68 to 6.37)	6.15 (5.79 to 6.53)	6.14 (5.78 to 6.51)	6.24 (5.85 to 6.63)	6.13 (5.7 to 6.52)	6.26 (5.8 to 6.7)	5.82 (5.28 to 6.36)	5.77 (4.9 to 6.61)
**North Carolina**	-2.29% (-16.41% to 13.32%)	6.91 (6.52 to 7.31)	7.08 (6.69 to 7.44)	7.25 (6.8 to 7.67)	6.72 (6.32 to 7.13)	6.91 (6.47 to 7.32)	6.91 (6.49 to 7.32)	6.75 (6.22 to 7.3)	6.75 (5.83 to 7.73)
**North Dakota**	0.22% (-14.58% to 17.22%)	7.61 (7.12 to 8.13)	7.58 (7.08 to 8.14)	8.03 (7.45 to 8.63)	7.82 (7.21 to 8.46)	8.25 (7.6 to 8.89)	8.42 (7.77 to 9.1)	8.08 (7.39 to 8.79)	7.63 (6.62 to 8.72)
**Ohio**	4.62% (-10.38% to 20.75%)	6.96 (6.6 to 7.3)	7.22 (6.86 to 7.63)	7.46 (7.06 to 7.89)	7.3 (6.92 to 7.72)	7.24 (6.85 to 7.68)	7.58 (7.15 to 8)	7.27 (6.72 to 7.85)	7.28 (6.26 to 8.36)
**Oklahoma**	1.98% (-12.44% to 17.19%)	7.09 (6.69 to 7.53)	7.36 (6.9 to 7.83)	7.6 (7.13 to 8.07)	7.44 (7 to 7.89)	7.56 (7.08 to 7.98)	7.63 (7.16 to 8.05)	7.22 (6.62 to 7.83)	7.23 (6.25 to 8.22)
**Oregon**	-6.62% (-20.8% to 10.65%)	8.17 (7.67 to 8.68)	8.65 (8.16 to 9.18)	8.32 (7.83 to 8.85)	8.55 (7.95 to 9.09)	8.05 (7.5 to 8.62)	8.03 (7.45 to 8.55)	7.58 (6.97 to 8.19)	7.63 (6.48 to 8.96)
**Pennsylvania**	-2.95% (-16.64% to 11.87%)	7.2 (6.78 to 7.62)	7.04 (6.66 to 7.43)	7.43 (7 to 7.87)	7.22 (6.79 to 7.71)	7.12 (6.65 to 7.59)	7.43 (6.97 to 7.93)	7.07 (6.42 to 7.7)	6.99 (6 to 8.03)
**Rhode Island**	-9.49% (-24.61% to 7.29%)	7.55 (7.07 to 8.08)	7.31 (6.82 to 7.83)	8.22 (7.59 to 8.84)	7.81 (7.21 to 8.37)	7.62 (7.05 to 8.2)	7.78 (7.17 to 8.34)	7.1 (6.36 to 7.86)	6.83 (5.75 to 7.93)
**South Carolina**	5.83% (-8.85% to 22.18%)	7.1 (6.73 to 7.5)	7.03 (6.68 to 7.44)	7.4 (6.96 to 7.85)	7.1 (6.64 to 7.49)	7.18 (6.72 to 7.58)	7.33 (6.9 to 7.78)	7.49 (6.88 to 8.1)	7.51 (6.42 to 8.55)
**South Dakota**	5.83% (-9.62% to 21.34%)	7.75 (7.23 to 8.27)	8.03 (7.52 to 8.64)	9.12 (8.49 to 9.81)	8.36 (7.78 to 8.93)	8.84 (8.2 to 9.47)	9.11 (8.45 to 9.75)	8.31 (7.65 to 8.97)	8.2 (7.2 to 9.28)
**Tennessee**	4.05% (-10.38% to 19.87%)	7.51 (7.1 to 7.95)	7.9 (7.45 to 8.38)	7.97 (7.51 to 8.42)	7.89 (7.4 to 8.35)	7.7 (7.23 to 8.2)	7.59 (7.13 to 8.09)	7.81 (7.2 to 8.44)	7.82 (6.78 to 8.99)
**Texas**	-9.25% (-20.86% to 4.57%)	7.12 (6.72 to 7.49)	7.12 (6.75 to 7.53)	7.29 (6.87 to 7.74)	6.73 (6.37 to 7.13)	6.63 (6.26 to 7.05)	6.83 (6.41 to 7.27)	6.41 (5.9 to 6.9)	6.46 (5.67 to 7.32)
**Utah**	2.95% (-12.6% to 20.36%)	6.6 (6.17 to 7.07)	7.07 (6.61 to 7.57)	7.4 (6.93 to 7.94)	7.16 (6.69 to 7.64)	7.27 (6.81 to 7.74)	7.4 (6.87 to 7.93)	6.82 (6.25 to 7.42)	6.79 (5.85 to 7.84)

© 2025 GBD 2021 US CNS Cancer Collaborators. *JAMA Neurology*.

**Vermont**	7.34% (-6.71% to 22.79%)	6.72 (6.28 to 7.18)	7.03 (6.56 to 7.54)	7.32 (6.79 to 7.87)	7.24 (6.71 to 7.82)	7.15 (6.6 to 7.68)	7.4 (6.79 to 7.98)	7.36 (6.71 to 8.11)	7.21 (6.31 to 8.18)
**Virginia**	1.22% (-14.5% to 19.09%)	6.68 (6.29 to 7.07)	6.8 (6.38 to 7.19)	7.03 (6.56 to 7.46)	6.91 (6.48 to 7.39)	6.85 (6.41 to 7.34)	6.94 (6.44 to 7.47)	6.73 (6.16 to 7.31)	6.76 (5.79 to 7.9)
**Washington**	-6.85% (-21.17% to 8.27%)	7.67 (7.25 to 8.1)	7.43 (7.02 to 7.86)	8.17 (7.64 to 8.7)	7.61 (7.15 to 8.08)	7.41 (6.91 to 7.93)	7.27 (6.76 to 7.76)	7.16 (6.6 to 7.77)	7.15 (6.1 to 8.24)
**West Virginia**	24.47% (6.71% to 44.56%)	6.28 (5.89 to 6.71)	6.87 (6.46 to 7.31)	7.15 (6.73 to 7.6)	7.34 (6.85 to 7.77)	7.51 (7.02 to 8.02)	7.91 (7.41 to 8.4)	7.79 (7.21 to 8.46)	7.81 (6.8 to 9.03)
**Wisconsin**	-0.93% (-17.26% to 16.45%)	7.8 (7.28 to 8.33)	7.86 (7.39 to 8.39)	8.28 (7.73 to 8.78)	7.95 (7.39 to 8.46)	7.85 (7.3 to 8.4)	8.02 (7.48 to 8.58)	7.81 (7.14 to 8.45)	7.73 (6.49 to 8.98)
**Wyoming**	4.6% (-9.11% to 19.72%)	6.83 (6.42 to 7.32)	7.1 (6.66 to 7.58)	7.78 (7.24 to 8.37)	7.05 (6.57 to 7.53)	7.54 (7.03 to 8.09)	7.7 (7.16 to 8.26)	7.22 (6.66 to 7.84)	7.14 (6.25 to 8.03)

© 2025 GBD 2021 US CNS Cancer Collaborators. *JAMA Neurology*.

eTable 9b: Age-standardized DALY rate of Brain and central nervous system cancer by state and year, and percentage change from 1990 to 2021, United States

	Percentage Change, 1990-2021	1990	1995	2000	2005	2010	2015	2020	2021
**United States of America**	-15.77% (-17.75% to -13.68%)	159.54 (156.74 to 161.86)	155.52 (152.79 to 157.4)	151.87 (148.77 to 154.2)	143.04 (139.81 to 145.18)	138.27 (134.76 to 140.55)	141.6 (137.86 to 143.88)	135.35 (131.37 to 138.17)	134.38 (129.83 to 137.95)
**Alabama**	-2.28% (-14.59% to 12.56%)	184.38 (177.81 to 191.66)	190.85 (184.22 to 198.52)	187.49 (180.01 to 194.13)	178.43 (171.91 to 185)	191.69 (184.83 to 198.44)	193.71 (186.51 to 200.88)	181 (169.66 to 193.21)	180.17 (157.11 to 205.96)
**Alaska**	-0.07% (-15.75% to 22.19%)	146.11 (139.33 to 153.75)	142.6 (135.77 to 150.21)	138.17 (130.1 to 146.3)	135.9 (128.31 to 143.81)	139.31 (132.92 to 147.29)	139.64 (133.3 to 147.71)	152.6 (139.58 to 167.55)	146 (124.29 to 176.99)
**Arizona**	-8.63% (-20.92% to 4.66%)	145.76 (140.32 to 151.8)	146.86 (141.07 to 152.7)	145.79 (139.52 to 152.25)	142.35 (136.63 to 148.24)	131.26 (125.97 to 136.42)	130.58 (124.94 to 135.52)	134.3 (124.59 to 144.41)	133.18 (115.31 to 152.62)
**Arkansas**	-5.44% (-18.7% to 8.89%)	186.4 (178.73 to 194.08)	184.99 (177.31 to 193.43)	186.19 (178.79 to 194.68)	172.29 (165.39 to 179.68)	174.71 (167.49 to 182.69)	186.39 (179.38 to 193.97)	175.82 (164.32 to 189.4)	176.27 (153.36 to 201.8)
**California**	-22.15% (-31.47% to -12.1%)	153.08 (148.67 to 156.97)	145.53 (141.2 to 150.02)	140.27 (135.46 to 144.99)	130.42 (125.54 to 134.68)	120.92 (116.59 to 125.7)	124.46 (119.37 to 128.85)	119.2 (109.65 to 127.46)	119.18 (105.5 to 134.14)
**Colorado**	-16.11% (-29.51% to -2.75%)	153.5 (147.22 to 159.57)	152.91 (146.67 to 159.41)	144.53 (138.79 to 150.31)	136.64 (130.94 to 142.79)	133.96 (128.34 to 139.15)	132.26 (126.75 to 137.7)	128.63 (119.68 to 138.44)	128.77 (109.46 to 148.96)
**Connecticut**	-18.07% (-31.23% to -3.83%)	151.73 (145.68 to 158.87)	149.95 (143.32 to 155.76)	145.97 (139 to 152.66)	136.55 (130.78 to 142.39)	132.39 (126.5 to 138.04)	133.55 (127.32 to 139.87)	127.84 (116.75 to 137.7)	124.32 (103.84 to 144.44)
**Delaware**	-12.9% (-23.3% to -2.16%)	149.34 (142.08 to 156.91)	144.22 (137.94 to 150.75)	147.65 (140.32 to 155.3)	136.12 (129.87 to 143.31)	135.46 (129.52 to 142.25)	131.74 (125.11 to 137.61)	129.95 (122.54 to 139.09)	130.07 (116.15 to 144.98)
**District of Columbia**	-43.24% (-51.77% to -33.88%)	177.6 (167.55 to 187.85)	156.5 (147.27 to 166.43)	137.37 (128.94 to 146.36)	123.51 (116.05 to 132.24)	101.89 (95.97 to 108.44)	96.72 (89.94 to 102.93)	103.03 (94.35 to 113.44)	100.8 (87.13 to 116.03)
**Florida**	-16.55% (-27.28% to -4.04%)	161.57 (156.03 to 167.48)	161.37 (156.64 to 166.63)	155.24 (149.88 to 160.13)	143.67 (138.95 to 148.03)	135.92 (131.4 to 140.29)	137.78 (132.66 to 142.53)	135.79 (126.39 to 143.93)	134.83 (116.98 to 154.42)
**Georgia**	-17.99% (-27.91% to -6.08%)	164.04 (158.91 to 169.21)	155.2 (149.63 to 160.25)	149.19 (143.85 to 154.78)	139.47 (134.74 to 144.29)	137.23 (132.91 to 141.48)	140.51 (135.15 to 145.55)	134.8 (126.63 to 143.7)	134.53 (117.65 to 153.81)
**Hawaii**	-14.52% (-26.75% to -1.07%)	104.02 (99.47 to 109.13)	105.26 (99.87 to 110.5)	98.58 (93.93 to 103.49)	98.24 (93.11 to 103.05)	96.12 (91.37 to 100.88)	95.95 (91.67 to 101.02)	92.12 (86.66 to 97.67)	88.92 (76.15 to 103.05)
**Idaho**	-11.89% (-25.48% to 2.71%)	161.06 (153.58 to 170.11)	162.98 (154.3 to 171.67)	162.3 (154.05 to 171.55)	153.72 (146.26 to 161.71)	150.9 (142.86 to 159.22)	152.7 (145.08 to 159.93)	141.94 (130.52 to 151.63)	141.91 (120.67 to 164.02)
**Illinois**	-18.65% (-29.96% to -6.79%)	156.04 (150.64 to 161.79)	150.62 (145.47 to 155.52)	146.36 (141.07 to 151.52)	130.08 (125.82 to 134.89)	130.52 (125.28 to 135.39)	132.62 (127.11 to 137.57)	127.02 (117.69 to 136.56)	126.94 (110.01 to 145.35)

© 2025 GBD 2021 US CNS Cancer Collaborators. *JAMA Neurology*.

**Indiana**	-7.32% (-19.9% to 6.49%)	161.26 (155.55 to 167.66)	164.75 (157.99 to 170.94)	161.87 (155.7 to 168.24)	156.3 (150.92 to 162.19)	151.88 (146.49 to 157.03)	162.26 (155.12 to 168.37)	150.91 (140.18 to 160.8)	149.46 (130.71 to 170.79)
**Iowa**	-3% (-17.89% to 12.42%)	156.37 (148.89 to 164.02)	162.24 (155.46 to 169.95)	161.86 (154.44 to 168.95)	155.63 (148.66 to 162.93)	156.73 (149.09 to 163.43)	160.68 (153.37 to 167.76)	158.53 (147.09 to 170.49)	151.68 (129.75 to 173.7)
**Kansas**	-1.65% (-16.67% to 14.31%)	161.87 (155.06 to 169.59)	168.02 (159.85 to 175.49)	164.35 (156.2 to 172.68)	166.81 (159.28 to 174.24)	165.01 (157.96 to 172.01)	166.93 (160.51 to 174.66)	161.17 (148.58 to 173.3)	159.2 (134.37 to 184.4)
**Kentucky**	-2.87% (-16.54% to 11.71%)	170.3 (163.94 to 177.37)	168.55 (162.08 to 175.01)	163.73 (157.57 to 170.27)	155.2 (149.76 to 161.26)	168.06 (161.82 to 174.51)	175.21 (168.18 to 183)	166.85 (155.82 to 178.03)	165.41 (141.72 to 189.2)
**Louisiana**	-2.25% (-15.6% to 12.5%)	161.53 (155.6 to 167.42)	159.29 (153.11 to 165.32)	163.89 (158.15 to 170.05)	172.4 (166.05 to 179.26)	155 (149.9 to 160.48)	162.62 (156.58 to 169.13)	157.26 (146.9 to 168.85)	157.9 (137.87 to 180.31)
**Maine**	-2.03% (-17.38% to 14.16%)	152.2 (144.28 to 160.48)	150.86 (142.28 to 160.15)	157.08 (148.23 to 165.52)	152.87 (144.2 to 161.06)	146.89 (139.18 to 155.25)	153.23 (144.7 to 162.86)	149.97 (139.22 to 160.5)	149.12 (128.42 to 173.16)
**Maryland**	-23.64% (-35.5% to -11.19%)	160.24 (152.95 to 167.01)	149.63 (143.47 to 155.63)	144.54 (138.58 to 151.08)	129.2 (123.21 to 134.94)	122.83 (117.15 to 128.47)	130.56 (125.1 to 137.11)	123.16 (114.11 to 132.83)	122.36 (104.67 to 142.28)
**Massachusetts**	-23.54% (-36.75% to -8.91%)	158.74 (152.9 to 164.56)	151.04 (145.18 to 156.95)	155.94 (149.69 to 162.25)	139.74 (133.83 to 145.27)	133.37 (128.07 to 139.16)	146.98 (140.56 to 154.61)	126.63 (115.95 to 137.6)	121.38 (101.83 to 143.06)
**Michigan**	-14.85% (-26.33% to -2.02%)	169.71 (164.08 to 175.82)	160.59 (155.09 to 166.19)	161.57 (154.93 to 167.86)	153.23 (147.74 to 158.44)	153.12 (147.66 to 158.07)	151.24 (145.24 to 156.43)	145.47 (136.26 to 156.21)	144.51 (125.37 to 164.88)
**Minnesota**	-14.84% (-28.2% to -1.15%)	159.55 (153.09 to 166.01)	156.93 (149.67 to 163.5)	162.91 (156.31 to 169.37)	137.22 (130.87 to 143.18)	143.71 (137.74 to 150.58)	145.51 (139.11 to 151.96)	138.13 (127.82 to 148.09)	135.87 (115.22 to 157.35)
**Mississippi**	4.85% (-8.82% to 20.26%)	182.16 (175.5 to 189.5)	191.38 (183.96 to 199.54)	197.42 (190.14 to 205.02)	189.15 (181.8 to 196.48)	178.79 (172.31 to 185.59)	189.49 (182.27 to 196.48)	191.7 (179.55 to 205.01)	190.99 (166.57 to 218.22)
**Missouri**	-6.28% (-20.62% to 8.5%)	162.73 (156.39 to 169.84)	166.6 (160.45 to 172.86)	164.39 (157.93 to 171.23)	157.47 (151.74 to 163.88)	151.57 (145.85 to 157.34)	153.64 (147.24 to 159.55)	152.82 (142.49 to 164.43)	152.51 (130.91 to 175.56)
**Montana**	-1.2% (-14.66% to 14.87%)	151.23 (143.32 to 160.11)	152.79 (145.62 to 160.35)	161.06 (153.07 to 169.5)	156.22 (148.51 to 164.17)	159.66 (151.61 to 168.87)	164.9 (156.18 to 173.5)	151.7 (140.54 to 162.64)	149.41 (131 to 172.5)
**Nebraska**	-6.97% (-20.95% to 8.06%)	169.81 (162.03 to 178.94)	169.85 (161.53 to 179.25)	172.28 (162.82 to 182.3)	159.52 (152.25 to 167.72)	157.99 (150.3 to 165.89)	163.15 (154.31 to 172.68)	159.46 (147.11 to 170.85)	157.97 (136.3 to 181.23)
**Nevada**	-17.23% (-29.16% to -4.55%)	147.73 (141.65 to 154.73)	148.69 (141.74 to 155.78)	144.31 (137.04 to 151.28)	145.01 (138.27 to 151.51)	134.29 (128.84 to 140.26)	135.98 (129.77 to 141.86)	123.21 (114.54 to 131.97)	122.27 (105.48 to 140.03)
**New Hampshire**	-11.21% (-24.68% to 4.28%)	147.71 (140.12 to 154.74)	149.61 (142.33 to 157.32)	143.56 (136.71 to 150.3)	141.31 (133.83 to 148.92)	134.47 (127.39 to 141.03)	147.58 (139.84 to 155.03)	133.36 (124.21 to 142.49)	131.15 (112.06 to 152.69)
**New Jersey**	-22.84% (-34.72% to -10.03%)	150.12 (144.36 to 155.71)	143.8 (138.57 to 149.31)	135.44 (130.34 to 140.53)	128.17 (122.92 to 132.77)	121.63 (116.86 to 126.12)	126.27 (120.72 to 131.2)	119.35 (109.76 to 129.24)	115.83 (99.03 to 134.88)

© 2025 GBD 2021 US CNS Cancer Collaborators. *JAMA Neurology*.

**New Mexico**	6.32% (-9.7% to 24.49%)	131.84 (124.19 to 139.4)	131.34 (124.33 to 137.87)	127.11 (119.67 to 133.87)	126.61 (119.42 to 132.92)	125.02 (118.67 to 132.09)	134.36 (127.27 to 141.18)	139.68 (128.67 to 149.44)	140.18 (120.51 to 162.98)
**New York**	-25.71% (-35.88% to -14.22%)	144.15 (138.79 to 149.83)	137.05 (131.72 to 142.74)	124.79 (119.78 to 129.5)	122.19 (117.71 to 127.48)	114.84 (110.53 to 119.54)	116.02 (110.99 to 120.75)	108.47 (99.61 to 116.96)	107.08 (92.7 to 123.05)
**North Carolina**	-17.4% (-29.19% to -4.57%)	164.84 (158.5 to 171.03)	161.73 (155.49 to 168.61)	157.05 (150.51 to 162.36)	141.04 (135.36 to 146.32)	138.97 (133.68 to 144.2)	139.72 (134.03 to 144.89)	136.71 (127.56 to 145.96)	136.16 (117.31 to 156.27)
**North Dakota**	-8.39% (-20.47% to 5.77%)	163.05 (155.77 to 170.95)	156.86 (148.18 to 164.96)	156.19 (147.59 to 165.61)	152.95 (144.27 to 162.18)	161.31 (152.72 to 169.35)	168.1 (159.41 to 176.88)	158.94 (148.1 to 170.28)	149.37 (129.48 to 169.48)
**Ohio**	-6.58% (-18.03% to 6.62%)	158.61 (153.5 to 163.55)	159.51 (154.54 to 164.88)	155.58 (150.34 to 161.06)	151.47 (146.52 to 156.17)	147.46 (142.07 to 152.6)	157.32 (151.86 to 163.04)	148.59 (138.69 to 158.04)	148.18 (129.22 to 168.38)
**Oklahoma**	-2.24% (-14.6% to 11.19%)	161.28 (155.25 to 168.33)	165.58 (158.52 to 172.46)	167.11 (160.01 to 174.46)	164.67 (158.12 to 170.82)	166.39 (158.95 to 173.57)	170.35 (163.08 to 177.54)	158.21 (147.03 to 169.47)	157.67 (138.54 to 178.51)
**Oregon**	-19.94% (-31.2% to -5.79%)	174.12 (166.64 to 181.74)	177.41 (170.25 to 185.08)	161.1 (154.33 to 167.51)	161.3 (154.62 to 167.9)	149.06 (142.81 to 155.83)	148.53 (141.57 to 154.61)	138.73 (130.21 to 147.47)	139.39 (120.78 to 163.47)
**Pennsylvania**	-17.31% (-28.41% to -5.28%)	163.08 (156.86 to 169.28)	152.1 (146.79 to 157.2)	152.07 (146.59 to 157.53)	144.04 (138.89 to 149.44)	139.41 (133.54 to 144.98)	144.25 (138.29 to 149.62)	137.05 (127.39 to 147.41)	134.86 (116.92 to 153.91)
**Rhode Island**	-24.7% (-37.61% to -10.8%)	164.94 (155.91 to 174.3)	151.73 (142.87 to 160.43)	160.16 (150.58 to 168.32)	148.31 (139.75 to 156.57)	141.67 (133.96 to 149.64)	143.99 (135.75 to 152.23)	129.69 (117.29 to 141.43)	124.2 (104.76 to 145.85)
**South Carolina**	-11.16% (-22.98% to 2.92%)	179.74 (172.31 to 188.26)	169.41 (161.84 to 176.84)	169.23 (162.06 to 176.6)	158.54 (151.89 to 165.58)	154.27 (147.53 to 160.61)	156.92 (149.98 to 163.42)	159.91 (148.87 to 172.18)	159.68 (137.93 to 182.62)
**South Dakota**	-4.01% (-16.42% to 9.54%)	173.71 (166.1 to 182)	173.34 (164.08 to 182.13)	187.57 (177 to 197.75)	172.15 (163.06 to 181.22)	178.83 (169.15 to 187.65)	188.4 (178.38 to 197.41)	169.48 (158.14 to 181.94)	166.73 (147.66 to 188.62)
**Tennessee**	-8.01% (-20.46% to 5.89%)	179.85 (172.36 to 187.33)	183.25 (176.06 to 190.53)	176.22 (169.72 to 182.76)	173.39 (166.71 to 179.81)	164.8 (158.53 to 170.79)	164.1 (156.73 to 170.6)	166.13 (154.59 to 177.27)	165.44 (144.36 to 190.32)
**Texas**	-23.7% (-32.75% to -13.49%)	164.81 (159.64 to 170.45)	157.36 (152.86 to 161.94)	150.72 (145.94 to 155.66)	137.89 (133.41 to 142.2)	131.91 (127.74 to 135.75)	136.26 (131.57 to 141)	125.32 (116.96 to 134.12)	125.75 (111.4 to 141.99)
**Utah**	-13.54% (-24.83% to -0.9%)	148.01 (141.07 to 155.24)	152.97 (145.49 to 160.51)	150.2 (142.58 to 158.3)	141.64 (134.31 to 147.57)	140 (133.45 to 146.45)	143.17 (136.48 to 149.89)	128.97 (119.78 to 138.06)	127.97 (111.6 to 145.88)
**Vermont**	-10.34% (-20.66% to 1.62%)	150.03 (142.21 to 157.37)	149.21 (142.35 to 157.46)	143.67 (136.15 to 152.05)	137.54 (130.15 to 145.24)	132.05 (124.46 to 140.32)	136.51 (128.04 to 143.47)	138.12 (128.65 to 148.33)	134.51 (120.29 to 152.18)
**Virginia**	-15.64% (-27.74% to -2.13%)	150.81 (144.97 to 156.67)	146.51 (140.23 to 152.56)	142.52 (136.2 to 148.85)	134.85 (128.62 to 140.55)	129.27 (123.44 to 135.45)	130.09 (124.47 to 135.73)	126.85 (118.46 to 135.56)	127.22 (108.47 to 147.46)
**Washington**	-19.71% (-32.53% to -7.22%)	173.37 (166.69 to 180.58)	160.29 (153.93 to 166.53)	164.15 (157.21 to 171.52)	151.31 (144.92 to 157.28)	145.43 (139.25 to 151.24)	143.84 (137.56 to 149.84)	139.97 (131.24 to 148.59)	139.19 (117.82 to 159.54)

© 2025 GBD 2021 US CNS Cancer Collaborators. *JAMA Neurology*.

**West Virginia**	16.12% (0.32% to 34.27%)	147.69 (140.91 to 155.81)	157.1 (149.55 to 164.03)	157.53 (150.3 to 164.37)	161.89 (154.45 to 169.42)	164.58 (156.61 to 172.06)	175.95 (168.62 to 184.2)	172.09 (161.26 to 184.76)	171.5 (148.81 to 197.7)
**Wisconsin**	-11.52% (-24.62% to 3.03%)	162.54 (155.24 to 168.74)	159.61 (153.48 to 166.18)	161.53 (155.22 to 168.12)	150.37 (143.22 to 156.07)	145.07 (138.3 to 151.27)	149.69 (143.87 to 156.42)	146.07 (135.94 to 155.8)	143.82 (123.71 to 166.72)
**Wyoming**	-7.68% (-17.94% to 4.49%)	153.98 (146.78 to 161.94)	154.06 (146.33 to 161.75)	163.52 (154.37 to 172.34)	146.07 (138.72 to 154.16)	153.7 (145.65 to 162.33)	156.99 (148.42 to 165.22)	144.2 (134.21 to 153.72)	142.15 (127.18 to 159.27)

© 2025 GBD 2021 US CNS Cancer Collaborators. *JAMA Neurology*.

eTable 9c: Age-standardized death rate of Brain and central nervous system cancer by state and year, and percentage change from 1990 to 2021, United States

	Percentage Change, 1990-2021	1990	1995	2000	2005	2010	2015	2020	2021
**United States of America**	-8.41% (-11.09% to -6.22%)	4.48 (4.34 to 4.56)	4.43 (4.28 to 4.5)	4.48 (4.31 to 4.58)	4.24 (4.04 to 4.33)	4.14 (3.94 to 4.25)	4.24 (4.03 to 4.35)	4.1 (3.88 to 4.21)	4.1 (3.87 to 4.22)
**Alabama**	5.79% (-8.7% to 22.35%)	5.14 (4.92 to 5.37)	5.34 (5.09 to 5.57)	5.49 (5.21 to 5.72)	5.22 (4.98 to 5.42)	5.71 (5.43 to 5.95)	5.68 (5.39 to 5.89)	5.42 (5.04 to 5.79)	5.44 (4.69 to 6.26)
**Alaska**	-4.8% (-17.4% to 10.28%)	4.1 (3.89 to 4.33)	4.03 (3.82 to 4.26)	4.01 (3.75 to 4.25)	3.84 (3.61 to 4.09)	4 (3.77 to 4.24)	3.94 (3.71 to 4.18)	3.94 (3.65 to 4.22)	3.9 (3.38 to 4.51)
**Arizona**	1.42% (-12.51% to 16.81%)	3.94 (3.76 to 4.11)	4.03 (3.84 to 4.21)	4.17 (3.95 to 4.38)	4.1 (3.87 to 4.3)	3.79 (3.57 to 3.97)	3.88 (3.62 to 4.07)	3.97 (3.62 to 4.3)	3.99 (3.45 to 4.6)
**Arkansas**	0.64% (-13.7% to 16.89%)	5.14 (4.88 to 5.37)	5.17 (4.92 to 5.42)	5.44 (5.17 to 5.73)	5 (4.76 to 5.24)	5.12 (4.84 to 5.37)	5.45 (5.18 to 5.7)	5.16 (4.78 to 5.55)	5.17 (4.47 to 5.96)
**California**	-12.56% (-24.18% to -1.2%)	4.24 (4.09 to 4.36)	4.14 (3.98 to 4.28)	4.1 (3.89 to 4.26)	3.86 (3.65 to 4)	3.64 (3.41 to 3.82)	3.78 (3.53 to 3.95)	3.67 (3.35 to 3.95)	3.7 (3.17 to 4.19)
**Colorado**	-9.33% (-24.2% to 6%)	4.36 (4.15 to 4.56)	4.44 (4.22 to 4.64)	4.29 (4.06 to 4.49)	4.04 (3.8 to 4.25)	4.08 (3.82 to 4.26)	3.99 (3.74 to 4.19)	3.9 (3.61 to 4.22)	3.96 (3.29 to 4.62)
**Connecticut**	-11.33% (-25.37% to 4.96%)	4.28 (4.07 to 4.5)	4.28 (4.06 to 4.47)	4.43 (4.17 to 4.65)	4.14 (3.91 to 4.35)	4.11 (3.85 to 4.32)	4.14 (3.89 to 4.37)	3.88 (3.52 to 4.21)	3.8 (3.17 to 4.46)
**Delaware**	-10.88% (-21.09% to 0.47%)	4.32 (4.08 to 4.55)	4.15 (3.93 to 4.36)	4.35 (4.08 to 4.59)	4.02 (3.8 to 4.25)	3.9 (3.67 to 4.11)	3.88 (3.64 to 4.06)	3.8 (3.56 to 4.09)	3.85 (3.42 to 4.34)
**District of Columbia**	-34.07% (-44.23% to -22.97%)	4.46 (4.19 to 4.72)	4.03 (3.8 to 4.28)	3.76 (3.53 to 4.01)	3.37 (3.18 to 3.61)	2.92 (2.74 to 3.1)	2.79 (2.6 to 2.97)	2.94 (2.68 to 3.23)	2.94 (2.53 to 3.39)
**Florida**	-9% (-21.41% to 4.78%)	4.45 (4.24 to 4.62)	4.52 (4.33 to 4.7)	4.53 (4.31 to 4.71)	4.15 (3.94 to 4.3)	3.98 (3.75 to 4.13)	4.05 (3.8 to 4.23)	4.04 (3.73 to 4.3)	4.05 (3.45 to 4.68)
**Georgia**	-10.12% (-21.77% to 3.63%)	4.55 (4.37 to 4.71)	4.46 (4.27 to 4.63)	4.4 (4.2 to 4.58)	4.11 (3.94 to 4.27)	4.17 (3.95 to 4.34)	4.23 (4 to 4.42)	4.07 (3.78 to 4.36)	4.09 (3.54 to 4.7)
**Hawaii**	-7.3% (-21.29% to 8.23%)	2.86 (2.7 to 3)	2.91 (2.74 to 3.06)	2.89 (2.71 to 3.06)	2.82 (2.63 to 2.99)	2.77 (2.58 to 2.93)	2.8 (2.62 to 2.97)	2.67 (2.45 to 2.85)	2.65 (2.25 to 3.06)
**Idaho**	-3.44% (-18.46% to 12.87%)	4.61 (4.33 to 4.91)	4.57 (4.32 to 4.84)	4.8 (4.49 to 5.08)	4.61 (4.35 to 4.86)	4.51 (4.19 to 4.79)	4.66 (4.39 to 4.93)	4.42 (4.04 to 4.75)	4.45 (3.73 to 5.18)
**Illinois**	-12.17% (-24.69% to 1.4%)	4.37 (4.19 to 4.55)	4.21 (4.03 to 4.36)	4.35 (4.14 to 4.54)	3.91 (3.71 to 4.07)	3.94 (3.73 to 4.12)	3.97 (3.73 to 4.14)	3.82 (3.51 to 4.13)	3.84 (3.27 to 4.43)
**Indiana**	0.1% (-13.45% to 15.3%)	4.52 (4.32 to 4.69)	4.74 (4.51 to 4.93)	4.77 (4.55 to 4.98)	4.58 (4.35 to 4.77)	4.52 (4.29 to 4.7)	4.77 (4.53 to 4.96)	4.52 (4.18 to 4.83)	4.53 (3.89 to 5.2)
**Iowa**	3% (-13.12% to 19.52%)	4.46 (4.21 to 4.69)	4.65 (4.43 to 4.88)	4.82 (4.56 to 5.04)	4.7 (4.43 to 4.96)	4.7 (4.41 to 4.93)	4.83 (4.53 to 5.06)	4.75 (4.36 to 5.14)	4.6 (3.89 to 5.31)
**Kansas**	6.28% (-10.57% to 23.74%)	4.56 (4.34 to 4.8)	4.79 (4.53 to 5.02)	4.93 (4.65 to 5.21)	4.99 (4.7 to 5.22)	4.82 (4.55 to 5.07)	4.97 (4.72 to 5.2)	4.89 (4.47 to 5.3)	4.85 (4.06 to 5.63)
**Kentucky**	1.86% (-12.88% to 17.21%)	4.84 (4.62 to 5.06)	4.85 (4.63 to 5.05)	4.85 (4.64 to 5.06)	4.54 (4.35 to 4.74)	4.95 (4.72 to 5.15)	5.15 (4.9 to 5.41)	4.92 (4.58 to 5.27)	4.93 (4.22 to 5.64)
**Louisiana**	4.59% (-9.95% to 21.43%)	4.44 (4.25 to 4.63)	4.43 (4.22 to 4.6)	4.68 (4.45 to 4.86)	5 (4.74 to 5.2)	4.52 (4.33 to 4.7)	4.73 (4.52 to 4.94)	4.61 (4.28 to 4.96)	4.65 (4.03 to 5.39)
**Maine**	3.04% (-12.96% to 19.54%)	4.44 (4.18 to 4.7)	4.52 (4.25 to 4.8)	4.8 (4.48 to 5.07)	4.64 (4.33 to 4.91)	4.5 (4.2 to 4.79)	4.62 (4.34 to 4.91)	4.53 (4.17 to 4.84)	4.58 (3.95 to 5.32)
**Maryland**	-20.78% (-32.67% to -7.05%)	4.58 (4.37 to 4.77)	4.27 (4.06 to 4.44)	4.22 (4.02 to 4.4)	3.78 (3.57 to 3.96)	3.67 (3.45 to 3.84)	3.78 (3.56 to 3.98)	3.62 (3.31 to 3.89)	3.62 (3.09 to 4.21)

© 2025 GBD 2021 US CNS Cancer Collaborators. *JAMA Neurology*.

**Massachusetts**	-11.83% (-26.33% to 5.12%)	4.59 (4.4 to 4.79)	4.38 (4.16 to 4.56)	4.81 (4.54 to 5.04)	4.38 (4.11 to 4.6)	4.24 (3.96 to 4.45)	4.72 (4.41 to 4.97)	4.19 (3.79 to 4.58)	4.05 (3.39 to 4.79)
**Michigan**	-8.83% (-21.45% to 5.63%)	4.73 (4.54 to 4.92)	4.55 (4.35 to 4.73)	4.75 (4.53 to 4.96)	4.56 (4.34 to 4.74)	4.51 (4.3 to 4.69)	4.45 (4.2 to 4.63)	4.32 (3.97 to 4.66)	4.31 (3.72 to 4.97)
**Minnesota**	-10.2% (-24.67% to 5.5%)	4.61 (4.38 to 4.82)	4.59 (4.35 to 4.81)	4.86 (4.6 to 5.09)	4.16 (3.91 to 4.38)	4.31 (4.07 to 4.54)	4.4 (4.12 to 4.61)	4.17 (3.82 to 4.5)	4.14 (3.47 to 4.85)
**Mississippi**	12.39% (-2.59% to 30.38%)	4.97 (4.75 to 5.2)	5.33 (5.09 to 5.57)	5.75 (5.49 to 6)	5.4 (5.16 to 5.64)	5.18 (4.92 to 5.4)	5.53 (5.27 to 5.77)	5.56 (5.14 to 5.97)	5.59 (4.83 to 6.43)
**Missouri**	0.46% (-14.45% to 16.53%)	4.55 (4.37 to 4.75)	4.64 (4.44 to 4.82)	4.8 (4.58 to 5)	4.56 (4.36 to 4.76)	4.43 (4.21 to 4.62)	4.47 (4.25 to 4.67)	4.54 (4.18 to 4.86)	4.57 (3.92 to 5.29)
**Montana**	2.83% (-11.83% to 18.94%)	4.25 (3.99 to 4.51)	4.38 (4.14 to 4.61)	4.67 (4.39 to 4.95)	4.53 (4.26 to 4.78)	4.51 (4.24 to 4.78)	4.73 (4.41 to 5)	4.39 (4.01 to 4.7)	4.37 (3.77 to 5.05)
**Nebraska**	0.43% (-14.31% to 16.03%)	4.81 (4.55 to 5.07)	4.89 (4.62 to 5.16)	5.2 (4.84 to 5.51)	4.75 (4.44 to 5.02)	4.75 (4.44 to 5.01)	4.92 (4.6 to 5.22)	4.81 (4.4 to 5.16)	4.83 (4.14 to 5.55)
**Nevada**	-9.48% (-22.91% to 4.97%)	4.17 (3.97 to 4.38)	4.25 (4.02 to 4.46)	4.31 (4.05 to 4.54)	4.32 (4.07 to 4.53)	4.04 (3.81 to 4.25)	4.09 (3.84 to 4.28)	3.79 (3.5 to 4.08)	3.78 (3.21 to 4.36)
**New Hampshire**	-5.77% (-20.29% to 10.74%)	4.38 (4.15 to 4.61)	4.55 (4.28 to 4.8)	4.49 (4.23 to 4.73)	4.39 (4.11 to 4.66)	4.22 (3.94 to 4.47)	4.41 (4.1 to 4.67)	4.14 (3.8 to 4.46)	4.13 (3.5 to 4.8)
**New Jersey**	-12.25% (-25.75% to 2.38%)	4.14 (3.96 to 4.3)	4.05 (3.86 to 4.21)	4.01 (3.82 to 4.17)	3.78 (3.58 to 3.93)	3.65 (3.44 to 3.8)	3.75 (3.52 to 3.92)	3.72 (3.37 to 4.04)	3.63 (3.07 to 4.25)
**New Mexico**	12.96% (-4.28% to 32.98%)	3.53 (3.31 to 3.74)	3.59 (3.36 to 3.79)	3.62 (3.39 to 3.82)	3.58 (3.34 to 3.78)	3.59 (3.35 to 3.8)	3.76 (3.53 to 3.97)	3.93 (3.59 to 4.24)	3.99 (3.4 to 4.68)
**New York**	-16.71% (-28.68% to -2.53%)	3.94 (3.77 to 4.1)	3.83 (3.65 to 4)	3.65 (3.47 to 3.8)	3.57 (3.39 to 3.73)	3.41 (3.21 to 3.57)	3.44 (3.24 to 3.61)	3.29 (2.99 to 3.56)	3.28 (2.78 to 3.81)
**North Carolina**	-11.71% (-24.77% to 2.24%)	4.69 (4.5 to 4.89)	4.65 (4.42 to 4.86)	4.69 (4.43 to 4.86)	4.21 (3.97 to 4.41)	4.2 (3.95 to 4.39)	4.22 (3.99 to 4.41)	4.12 (3.83 to 4.43)	4.14 (3.53 to 4.78)
**North Dakota**	-9.76% (-22.63% to 4.27%)	4.69 (4.45 to 4.97)	4.52 (4.26 to 4.78)	4.69 (4.39 to 4.98)	4.44 (4.11 to 4.76)	4.63 (4.3 to 4.89)	4.8 (4.49 to 5.1)	4.5 (4.12 to 4.84)	4.24 (3.62 to 4.86)
**Ohio**	1.92% (-11.44% to 16.96%)	4.44 (4.28 to 4.59)	4.51 (4.34 to 4.68)	4.59 (4.39 to 4.78)	4.47 (4.26 to 4.63)	4.43 (4.21 to 4.59)	4.63 (4.4 to 4.8)	4.5 (4.16 to 4.8)	4.53 (3.91 to 5.17)
**Oklahoma**	2.38% (-11.06% to 17.15%)	4.59 (4.36 to 4.81)	4.68 (4.45 to 4.88)	4.85 (4.63 to 5.07)	4.77 (4.55 to 4.96)	4.86 (4.61 to 5.08)	4.92 (4.65 to 5.14)	4.69 (4.33 to 5.03)	4.7 (4.1 to 5.41)
**Oregon**	-14.28% (-26.75% to 1.14%)	4.99 (4.74 to 5.23)	5.17 (4.92 to 5.42)	4.78 (4.53 to 4.99)	4.88 (4.62 to 5.12)	4.49 (4.24 to 4.7)	4.55 (4.25 to 4.76)	4.24 (3.91 to 4.52)	4.28 (3.67 to 5.05)
**Pennsylvania**	-11.06% (-23.71% to 2.53%)	4.61 (4.41 to 4.79)	4.35 (4.15 to 4.5)	4.43 (4.21 to 4.61)	4.26 (4.03 to 4.46)	4.2 (3.98 to 4.4)	4.33 (4.1 to 4.52)	4.13 (3.78 to 4.46)	4.1 (3.52 to 4.73)
**Rhode Island**	-18.17% (-31.43% to -3.07%)	4.84 (4.56 to 5.13)	4.52 (4.24 to 4.79)	4.79 (4.49 to 5.06)	4.54 (4.26 to 4.82)	4.36 (4.05 to 4.62)	4.43 (4.14 to 4.69)	4.05 (3.61 to 4.43)	3.96 (3.37 to 4.65)
**South Carolina**	-5.08% (-19.19% to 9.53%)	5.03 (4.79 to 5.28)	4.76 (4.53 to 5)	4.98 (4.69 to 5.21)	4.61 (4.35 to 4.82)	4.57 (4.3 to 4.8)	4.71 (4.45 to 4.92)	4.74 (4.38 to 5.12)	4.77 (4.08 to 5.46)
**South Dakota**	0.49% (-13.29% to 15.36%)	4.8 (4.54 to 5.05)	4.88 (4.58 to 5.17)	5.35 (4.98 to 5.68)	4.86 (4.54 to 5.16)	5.04 (4.71 to 5.31)	5.21 (4.87 to 5.51)	4.89 (4.51 to 5.27)	4.82 (4.24 to 5.51)
**Tennessee**	-3.73% (-17.06% to 11.01%)	5.11 (4.87 to 5.35)	5.22 (4.99 to 5.45)	5.15 (4.91 to 5.37)	5.12 (4.89 to 5.34)	4.87 (4.63 to 5.07)	4.8 (4.54 to 5)	4.89 (4.53 to 5.21)	4.92 (4.25 to 5.68)
**Texas**	-15.48% (-25.61% to -3.85%)	4.69 (4.52 to 4.87)	4.55 (4.37 to 4.71)	4.54 (4.31 to 4.72)	4.14 (3.97 to 4.29)	3.97 (3.77 to 4.12)	4.16 (3.93 to 4.34)	3.93 (3.63 to 4.23)	3.97 (3.49 to 4.5)
**Utah**	-2.74% (-16.17% to 11.84%)	4.17 (3.94 to 4.4)	4.35 (4.1 to 4.58)	4.49 (4.21 to 4.75)	4.23 (3.96 to 4.43)	4.15 (3.89 to 4.38)	4.36 (4.09 to 4.59)	4.06 (3.75 to 4.37)	4.06 (3.5 to 4.69)

© 2025 GBD 2021 US CNS Cancer Collaborators. *JAMA Neurology*.

**Vermont**	-8.87%<br>(-19.78% to 3.77%)	4.43<br>(4.16 to 4.68)	4.41<br>(4.18 to 4.65)	4.37<br>(4.1 to 4.66)	4.06<br>(3.81 to 4.29)	4.13<br>(3.85 to 4.43)	4.19<br>(3.88 to 4.43)	4.04<br>(3.71 to 4.33)	4.04<br>(3.55 to 4.59)
**Virginia**	-10.21%<br>(-23.22% to 4.85%)	4.33<br>(4.13 to 4.51)	4.26<br>(4.05 to 4.45)	4.3<br>(4.05 to 4.52)	4.06<br>(3.82 to 4.26)	3.97<br>(3.74 to 4.19)	3.99<br>(3.73 to 4.2)	3.85<br>(3.55 to 4.15)	3.89<br>(3.33 to 4.52)
**Washington**	-14.16%<br>(-27.5% to 0.2%)	5.01<br>(4.76 to 5.24)	4.58<br>(4.34 to 4.78)	4.98<br>(4.67 to 5.23)	4.65<br>(4.38 to 4.88)	4.48<br>(4.19 to 4.7)	4.43<br>(4.15 to 4.65)	4.27<br>(3.94 to 4.57)	4.3<br>(3.63 to 4.96)
**West Virginia**	19.21%<br>(2.23% to 38.8%)	4.2<br>(3.99 to 4.44)	4.51<br>(4.26 to 4.74)	4.58<br>(4.34 to 4.82)	4.69<br>(4.43 to 4.93)	4.81<br>(4.54 to 5.05)	5.09<br>(4.82 to 5.34)	4.98<br>(4.64 to 5.36)	5.01<br>(4.34 to 5.79)
**Wisconsin**	-5.96%<br>(-19.87% to 11.32%)	4.64<br>(4.42 to 4.82)	4.63<br>(4.42 to 4.84)	4.74<br>(4.51 to 4.96)	4.52<br>(4.25 to 4.72)	4.46<br>(4.2 to 4.69)	4.52<br>(4.27 to 4.74)	4.39<br>(4.04 to 4.7)	4.36<br>(3.71 to 5.11)
**Wyoming**	-0.6%<br>(-12.2% to 12.84%)	4.33<br>(4.09 to 4.57)	4.38<br>(4.13 to 4.62)	4.69<br>(4.39 to 4.95)	4.31<br>(4.05 to 4.56)	4.46<br>(4.21 to 4.73)	4.52<br>(4.26 to 4.79)	4.29<br>(3.99 to 4.58)	4.3<br>(3.8 to 4.82)

© 2025 GBD 2021 US CNS Cancer Collaborators. *JAMA Neurology*.

eTable 9d: Age-standardized prevalence rate of Brain and central nervous system cancer by state and year, and percentage change from 1990 to 2021, United States

	Percentage Change, 1990-2021	1990	1995	2000	2005	2010	2015	2020	2021
**United States of America**	8.28% (4.11% to 12.56%)	21.59 (21.12 to 22.04)	22.83 (22.37 to 23.26)	24.73 (24.2 to 25.22)	24.5 (23.97 to 24.92)	24.49 (23.95 to 25.04)	24.92 (24.3 to 25.47)	23.64 (22.96 to 24.29)	23.38 (22.43 to 24.28)
**Alabama**	12.11% (-6.5% to 32.03%)	20.87 (19.28 to 22.64)	23.32 (21.56 to 25.35)	23.65 (21.75 to 25.68)	23.08 (21.27 to 25.1)	25.47 (23.24 to 27.64)	25.54 (23.39 to 27.73)	23.5 (21.26 to 26.04)	23.4 (20.08 to 26.85)
**Alaska**	55.88% (22.17% to 110.4%)	18.54 (16.87 to 20.37)	20.02 (18.07 to 21.92)	22.55 (20.27 to 24.87)	24.21 (21.72 to 26.82)	25.44 (23.06 to 28.23)	25.94 (23.51 to 28.61)	30.98 (26.67 to 37.17)	28.9 (23.25 to 39.3)
**Arizona**	18.03% (-1.02% to 41.36%)	20.74 (18.73 to 22.74)	22.21 (20.15 to 24.24)	23.53 (21.28 to 25.93)	24.48 (22.33 to 26.74)	24.42 (22.06 to 27.03)	23.72 (21.43 to 26.18)	24.94 (22.66 to 27.79)	24.48 (21.07 to 28.54)
**Arkansas**	7.64% (-10.2% to 27.54%)	22.42 (20.61 to 24.37)	23.94 (22.02 to 26.12)	24.34 (22.23 to 26.65)	22.85 (20.85 to 24.95)	24.19 (22.27 to 26.5)	24.73 (22.75 to 27.06)	23.91 (21.6 to 26.61)	24.13 (20.68 to 27.98)
**California**	3.4% (-12.7% to 22.61%)	22.3 (20.6 to 24.3)	23.36 (21.69 to 25.17)	26.56 (24.9 to 28.57)	24.29 (22.64 to 25.98)	22.58 (20.77 to 24.52)	24.04 (21.96 to 26.46)	23.21 (20.7 to 26.15)	23.05 (19.86 to 26.87)
**Colorado**	9.2% (-9.41% to 30.08%)	22.69 (20.81 to 24.86)	23.72 (21.65 to 25.69)	25.07 (22.96 to 27.71)	25.28 (22.93 to 27.75)	25.64 (23.22 to 28.05)	25.67 (23.33 to 28.29)	25.01 (22.36 to 27.89)	24.77 (20.81 to 28.87)
**Connecticut**	2% (-15.81% to 22.94%)	32.05 (29.38 to 35.25)	33.01 (30.18 to 36.56)	34.75 (31.84 to 38.24)	35.87 (32.49 to 39.37)	35.53 (32.35 to 39.09)	35.56 (32.36 to 39.37)	33.59 (29.52 to 37.72)	32.69 (27.42 to 38.97)
**Delaware**	20.94% (2.46% to 42.31%)	18.42 (16.68 to 20.14)	19.26 (17.56 to 21.19)	21.93 (19.9 to 24.27)	21.62 (19.62 to 23.66)	23.83 (21.6 to 26.32)	22.96 (20.78 to 25.41)	22.49 (20.33 to 24.89)	22.28 (19.4 to 25.45)
**District of Columbia**	-4.8% (-20.68% to 12.93%)	12.95 (11.95 to 14.24)	12.56 (11.55 to 13.62)	12.98 (11.72 to 14.31)	13.73 (12.34 to 15.24)	11.62 (10.56 to 13.06)	11.36 (10.26 to 12.66)	12.9 (11.45 to 14.77)	12.33 (10.39 to 14.37)
**Florida**	6.13% (-11.45% to 26.42%)	20.55 (18.86 to 22.47)	22.27 (20.62 to 24.13)	25.14 (23.3 to 27.07)	24.28 (22.3 to 26.54)	23.38 (21.37 to 25.61)	23 (21.06 to 25.2)	22.13 (19.94 to 24.6)	21.81 (18.65 to 25.3)
**Georgia**	16.15% (-1.56% to 36.79%)	17.61 (16.32 to 19)	18.41 (16.89 to 19.97)	20.32 (18.73 to 22.13)	21.45 (19.72 to 23.43)	22.27 (20.6 to 24.19)	21.52 (19.67 to 23.53)	20.52 (18.49 to 22.91)	20.46 (17.57 to 23.94)
**Hawaii**	6.41% (-12.46% to 27.71%)	15.21 (13.8 to 16.87)	16.87 (15.18 to 18.71)	16.77 (15.1 to 18.74)	18 (16.15 to 19.96)	17.86 (16.16 to 19.76)	17.94 (16.13 to 19.79)	17.17 (15.56 to 19.06)	16.18 (13.74 to 18.72)
**Idaho**	7.15% (-11.81% to 27.08%)	22.67 (20.63 to 25.04)	25.44 (22.95 to 28.04)	26.32 (23.84 to 29.15)	25.49 (23.34 to 27.92)	26.79 (24.42 to 29.43)	26.17 (23.91 to 28.87)	24.28 (21.77 to 26.79)	24.29 (20.45 to 27.87)
**Illinois**	16.31% (-1.06% to 36.44%)	19.67 (17.97 to 21.42)	20.8 (19.1 to 22.81)	22.25 (20.47 to 24.43)	21.84 (19.85 to 23.98)	23.42 (21.11 to 25.7)	23.98 (21.88 to 26.39)	22.84 (20.48 to 25.58)	22.87 (19.69 to 26.58)
**Indiana**	7.97% (-10.41% to 26.92%)	22.19 (20.51 to 24.14)	23.83 (21.78 to 26.24)	24.68 (22.39 to 27.11)	24.41 (22.2 to 26.88)	24.21 (22.12 to 26.47)	25.78 (23.68 to 28.21)	24.26 (21.8 to 26.7)	23.95 (20.44 to 27.44)
**Iowa**	15.1% (-5.95% to 39.72%)	19.44 (17.79 to 21.3)	21.27 (19.4 to 23.4)	23.31 (21.22 to 25.48)	23.44 (21.43 to 25.95)	24.19 (21.99 to 26.4)	23.69 (21.38 to 25.91)	23.47 (21.19 to 26.11)	22.38 (18.79 to 26.27)
**Kansas**	10.63% (-7.43% to 32.17%)	24.95 (22.78 to 27.55)	27.32 (24.79 to 29.72)	27.71 (25.22 to 30.22)	28.29 (25.87 to 30.93)	29.24 (26.69 to 32.38)	28.62 (26.04 to 31.83)	27.84 (25.05 to 30.93)	27.6 (23.55 to 32)
**Kentucky**	6.43% (-12.38% to 28.36%)	26.94 (24.61 to 29.57)	29.01 (26.29 to 31.72)	30.2 (27.43 to 33.07)	29.38 (26.89 to 32.03)	30.93 (28.27 to 33.82)	30.62 (27.7 to 33.44)	28.93 (26.11 to 32.1)	28.67 (24.41 to 33.52)
**Louisiana**	17.19% (-0.65% to 39.28%)	18.84 (17.28 to 20.55)	20.35 (18.61 to 22.37)	22.88 (20.99 to 25.14)	25.68 (23.68 to 28.14)	23.44 (21.5 to 25.64)	23.2 (21.33 to 25.38)	21.96 (19.82 to 24.33)	22.07 (19.1 to 25.86)
**Maine**	11.8% (-6.81% to 35.2%)	23.02 (21 to 25.55)	24.57 (22.33 to 27.37)	27.43 (24.91 to 30.39)	27.68 (25.23 to 30.46)	27.17 (24.5 to 29.93)	27.2 (24.77 to 30.17)	26.1 (23.4 to 29.19)	25.74 (21.91 to 30.13)
**Maryland**	6.34% (-11.37% to 27.43%)	19.59 (17.9 to 21.34)	20.18 (18.42 to 22)	21.42 (19.57 to 23.52)	21.55 (19.59 to 23.97)	21.6 (19.76 to 23.76)	23.13 (21.04 to 25.38)	21.03 (18.78 to 23.64)	20.83 (17.82 to 24.34)

© 2025 GBD 2021 US CNS Cancer Collaborators. *JAMA Neurology*.

**Massachusetts**	-0.3% (-17.44% to 20.76%)	24.96 (22.84 to 27.42)	25.95 (23.64 to 28.35)	30.18 (27.51 to 33.11)	28.84 (26.06 to 31.54)	28.67 (26.18 to 31.41)	31.93 (29.16 to 34.8)	25.92 (22.9 to 29.05)	24.88 (20.88 to 29.62)
**Michigan**	10.44% (-7.01% to 30.23%)	19.43 (17.94 to 20.89)	20.62 (19.12 to 22.34)	22.52 (20.87 to 24.31)	23.42 (21.53 to 25.71)	23.91 (21.93 to 26.06)	22.86 (20.92 to 25.07)	21.59 (19.39 to 23.98)	21.45 (18.35 to 25.16)
**Minnesota**	11.51% (-9.57% to 33.11%)	27.18 (24.5 to 29.98)	28.77 (25.81 to 31.82)	32.69 (29.57 to 35.86)	29.45 (26.91 to 32.54)	32.2 (28.96 to 35.46)	32.06 (28.92 to 35)	30.99 (27.96 to 34.3)	30.3 (25.48 to 35.4)
**Mississippi**	18.71% (0.25% to 40.94%)	19.72 (18.07 to 21.57)	21.57 (19.86 to 23.45)	22.66 (20.97 to 24.51)	21.99 (20.2 to 23.92)	22.11 (20.36 to 24.14)	22.68 (20.75 to 24.63)	23.5 (21.4 to 25.98)	23.41 (20.12 to 26.93)
**Missouri**	14.07% (-3.85% to 36.18%)	21.76 (19.72 to 23.85)	23.71 (21.73 to 25.95)	25.25 (23.04 to 27.59)	24.74 (22.56 to 26.95)	25.25 (23.1 to 27.67)	25.18 (23.14 to 27.53)	24.9 (22.36 to 27.61)	24.83 (21.34 to 28.81)
**Montana**	20.08% (0.7% to 40.9%)	21.3 (19.35 to 23.27)	22.98 (21.1 to 25.34)	26.42 (24.11 to 29.3)	26.31 (23.9 to 28.78)	28.75 (26.03 to 32.3)	27.79 (25.39 to 30.76)	26.03 (23.36 to 28.96)	25.58 (22.2 to 29.61)
**Nebraska**	12.55% (-6.65% to 35.13%)	26.52 (24.15 to 29.26)	27.96 (25.48 to 30.94)	30.3 (27.36 to 33.9)	30.31 (27.3 to 33.64)	30.76 (27.53 to 33.72)	30.49 (27.65 to 33.66)	30.38 (27.24 to 33.68)	29.85 (25.56 to 34.51)
**Nevada**	9.1% (-7.98% to 28.21%)	17.96 (16.43 to 19.75)	19.03 (17.36 to 20.76)	20.07 (18.2 to 22.11)	21.38 (19.41 to 23.28)	21.51 (19.54 to 23.82)	22.09 (19.98 to 24.45)	19.71 (17.64 to 21.81)	19.6 (16.79 to 22.59)
**New Hampshire**	17.75% (-2.04% to 40.53%)	21.75 (19.78 to 23.77)	23.37 (21.35 to 25.52)	25.55 (23.1 to 27.99)	28.07 (25.18 to 30.93)	27.51 (25.05 to 30.54)	31.41 (28.23 to 34.93)	26.34 (23.84 to 29.33)	25.61 (21.68 to 29.76)
**New Jersey**	5.32% (-13.62% to 25.46%)	35.13 (31.72 to 38.71)	37.67 (34.04 to 41.68)	41.45 (38.21 to 45.03)	41.77 (38.64 to 45.07)	40.39 (37.22 to 43.65)	42.08 (38.39 to 45.57)	38.14 (34.21 to 42.62)	37 (31.61 to 43.37)
**New Mexico**	28.31% (5.04% to 54.72%)	17.66 (16.03 to 19.42)	19.32 (17.68 to 21.09)	19.87 (18.24 to 22.02)	20.49 (18.58 to 22.87)	21.5 (19.4 to 23.58)	22.63 (20.39 to 24.95)	22.61 (20.29 to 25.16)	22.66 (19.42 to 26.7)
**New York**	14.81% (-1.98% to 36.17%)	17.79 (16.25 to 19.26)	18.63 (17.08 to 20.34)	20.08 (18.48 to 21.95)	22.38 (20.41 to 24.57)	22.46 (20.42 to 24.56)	23.15 (20.86 to 25.31)	20.8 (18.57 to 23.2)	20.43 (17.56 to 23.61)
**North Carolina**	13.36% (-3.95% to 33.86%)	19.2 (17.6 to 21.05)	20.52 (18.84 to 22.31)	21.57 (19.63 to 23.56)	21.36 (19.51 to 23.4)	22.51 (20.52 to 24.76)	22.45 (20.65 to 24.41)	21.9 (19.57 to 24.22)	21.77 (18.81 to 24.9)
**North Dakota**	14.82% (-3.32% to 37.09%)	24.47 (22.22 to 26.92)	25.57 (22.97 to 28.3)	27.35 (24.89 to 30.07)	28.19 (25.3 to 31.32)	30.29 (27.35 to 33.4)	30.08 (27.02 to 33.11)	29.67 (26.45 to 33.51)	28.1 (24.21 to 32.88)
**Ohio**	6.87% (-9.3% to 26.73%)	21.38 (19.64 to 23.36)	22.69 (21.01 to 24.72)	23.68 (21.8 to 25.62)	23.51 (21.58 to 25.69)	23.38 (21.39 to 25.66)	24.76 (22.81 to 27.15)	22.97 (20.81 to 25.16)	22.85 (19.7 to 26.29)
**Oklahoma**	0.54% (-15.65% to 18.48%)	21.36 (19.57 to 23.46)	23.01 (20.79 to 25.36)	22.98 (20.98 to 25.03)	22.28 (20.39 to 24.46)	23.1 (21.2 to 25.16)	23.42 (21.42 to 25.68)	21.5 (19.38 to 23.67)	21.48 (18.61 to 24.43)
**Oregon**	3.76% (-14.01% to 24.44%)	26.43 (24.07 to 28.88)	28.47 (26.25 to 31.17)	28.86 (26.31 to 31.56)	30.33 (27.36 to 33.22)	29.08 (26.56 to 32.02)	28.67 (25.78 to 31.35)	27.29 (24.53 to 30.25)	27.43 (23.32 to 32.18)
**Pennsylvania**	9.38% (-7.91% to 28.8%)	21.75 (19.81 to 23.84)	22.11 (20.38 to 24.32)	24.64 (22.65 to 27.15)	24.57 (22.41 to 27.1)	24.01 (21.82 to 26.32)	25.37 (23.26 to 27.82)	24.15 (21.66 to 26.93)	23.79 (20.76 to 27.26)
**Rhode Island**	3.17% (-15.35% to 22.12%)	22.72 (20.64 to 24.8)	22.58 (20.45 to 25.09)	27.59 (24.84 to 30.58)	27.05 (24.6 to 29.77)	26.72 (24.09 to 29.61)	27.35 (24.73 to 30.07)	24.96 (22.27 to 28.01)	23.44 (19.54 to 27.85)
**South Carolina**	23.68% (2.93% to 45.27%)	18.67 (17.08 to 20.29)	19.9 (18.31 to 21.75)	21.03 (19.32 to 22.81)	21.55 (19.65 to 23.55)	22.02 (20 to 23.91)	22.4 (20.53 to 24.53)	23.18 (20.79 to 25.6)	23.1 (19.9 to 26.22)
**South Dakota**	11.62% (-6.93% to 31.79%)	25.13 (22.7 to 27.56)	26.7 (24.38 to 29.53)	31.78 (28.41 to 35.43)	29.38 (26.35 to 32.41)	31.93 (28.88 to 35.57)	33.03 (29.51 to 36.58)	28.3 (25.06 to 31.52)	28.05 (24.25 to 31.89)
**Tennessee**	15.6% (-2.41% to 36.07%)	20.94 (19.16 to 22.75)	23.1 (20.98 to 25.2)	23.94 (21.83 to 26.15)	23.53 (21.51 to 25.62)	24.02 (22 to 26.33)	23.85 (21.76 to 26.16)	24.34 (22.02 to 26.87)	24.2 (20.9 to 28.07)
**Texas**	0.02% (-13.63% to 16.95%)	20.77 (19.01 to 22.45)	21.52 (19.74 to 23.45)	22.58 (20.69 to 24.59)	21.74 (19.77 to 23.69)	22.38 (20.25 to 24.56)	22.57 (20.64 to 24.73)	20.72 (18.75 to 22.93)	20.77 (18.18 to 23.68)
**Utah**	6.54% (-10.82% to 25.99%)	20.66 (18.78 to 22.77)	22.79 (20.58 to 25.17)	24.1 (22.03 to 26.33)	24.49 (22.09 to 26.89)	25.52 (23.27 to 27.85)	24.8 (22.62 to 27.17)	22.3 (20.32 to 24.65)	22.01 (19.09 to 25.41)

© 2025 GBD 2021 US CNS Cancer Collaborators. *JAMA Neurology*.

**Vermont**	31.79% (12.52% to 54.41%)	19.54 (17.72 to 21.47)	21.43 (19.16 to 23.67)	24.11 (21.85 to 26.57)	26.73 (23.87 to 30.08)	24.67 (22.26 to 27.12)	25.96 (23.29 to 28.85)	26.98 (24.03 to 30.45)	25.75 (22.34 to 29.52)
**Virginia**	18.55% (-1.14% to 41.72%)	19.95 (18.26 to 21.67)	21.01 (19.1 to 23.08)	22.62 (20.58 to 24.78)	23.93 (21.83 to 26.41)	23.73 (21.59 to 26.05)	24.15 (21.92 to 26.33)	23.68 (21.29 to 26.28)	23.65 (20.02 to 27.78)
**Washington**	4.49% (-11.54% to 22.72%)	19.62 (18.05 to 21.1)	20.73 (19.18 to 22.47)	23.27 (21.5 to 25.3)	22.5 (20.86 to 24.62)	22.12 (20.26 to 24.05)	21.17 (19.32 to 23.09)	20.96 (19.05 to 23.17)	20.51 (17.69 to 23.73)
**West Virginia**	30.14% (9.96% to 54.12%)	17.83 (16.34 to 19.69)	20.27 (18.56 to 22.26)	21.77 (19.88 to 23.83)	22.22 (20.2 to 24.26)	22.84 (20.75 to 25.05)	23.78 (21.61 to 26.03)	23.26 (21.15 to 25.8)	23.21 (20 to 26.84)
**Wisconsin**	5.35% (-13.71% to 25.77%)	26.2 (23.83 to 28.92)	26.78 (24.41 to 29.45)	29.39 (26.67 to 32.35)	28.49 (25.82 to 31.14)	27.77 (24.9 to 30.5)	28.62 (25.98 to 31.32)	28.04 (25.16 to 31.36)	27.6 (23.3 to 31.87)
**Wyoming**	8.84% (-8.87% to 27.17%)	21.35 (19.43 to 23.58)	23.08 (20.84 to 25.64)	25.78 (23.09 to 28.71)	22.87 (20.81 to 25.23)	25.71 (23.35 to 28.37)	26.32 (23.64 to 29.1)	24.11 (21.62 to 26.73)	23.24 (20.06 to 26.35)

© 2025 GBD 2021 US CNS Cancer Collaborators. *JAMA Neurology*.

eTable 9e: Age-standardized YLD rate of Brain and central nervous system cancer by state and year, and percentage change from 1990 to 2021, United States

	Percentage Change, 1990-2021	1990	1995	2000	2005	2010	2015	2020	2021
**United States of America**	2.03% (-2.1% to 6.25%)	2.55 (1.86 to 3.32)	2.64 (1.92 to 3.43)	2.78 (2.05 to 3.65)	2.71 (2 to 3.57)	2.69 (2.02 to 3.53)	2.74 (2.03 to 3.57)	2.62 (1.93 to 3.44)	2.6 (1.92 to 3.4)
**Alabama**	8.07% (-10.76% to 27.78%)	2.6 (1.85 to 3.41)	2.81 (2.06 to 3.69)	2.86 (2.1 to 3.78)	2.76 (1.98 to 3.62)	3.04 (2.16 to 4.09)	3.02 (2.18 to 4.04)	2.82 (1.98 to 3.79)	2.81 (1.96 to 3.84)
**Alaska**	32.65% (6.52% to 72.42%)	2.21 (1.59 to 2.92)	2.33 (1.63 to 3.08)	2.52 (1.81 to 3.34)	2.59 (1.87 to 3.46)	2.72 (1.94 to 3.62)	2.76 (1.99 to 3.69)	3.08 (2.2 to 4.24)	2.94 (2.07 to 4.36)
**Arizona**	11.28% (-7.73% to 35.78%)	2.39 (1.76 to 3.15)	2.5 (1.78 to 3.35)	2.61 (1.83 to 3.47)	2.67 (1.88 to 3.54)	2.63 (1.88 to 3.55)	2.59 (1.83 to 3.45)	2.69 (1.94 to 3.51)	2.66 (1.86 to 3.61)
**Arkansas**	3.7% (-14.58% to 25.31%)	2.72 (1.96 to 3.62)	2.84 (2.03 to 3.7)	2.89 (2.09 to 3.78)	2.71 (1.98 to 3.57)	2.85 (2.04 to 3.78)	2.96 (2.13 to 3.86)	2.84 (2.06 to 3.8)	2.82 (2 to 3.71)
**California**	-3.36% (-19.04% to 15.27%)	2.52 (1.8 to 3.32)	2.6 (1.82 to 3.45)	2.81 (2.05 to 3.68)	2.57 (1.87 to 3.41)	2.4 (1.72 to 3.2)	2.52 (1.79 to 3.29)	2.43 (1.75 to 3.22)	2.43 (1.73 to 3.23)
**Colorado**	3.88% (-15.4% to 28.55%)	2.59 (1.83 to 3.39)	2.72 (1.93 to 3.57)	2.76 (1.99 to 3.63)	2.73 (1.95 to 3.7)	2.76 (1.94 to 3.65)	2.77 (1.93 to 3.62)	2.7 (1.91 to 3.64)	2.69 (1.86 to 3.64)
**Connecticut**	-2.12% (-21.08% to 20.37%)	3.48 (2.46 to 4.62)	3.58 (2.58 to 4.65)	3.7 (2.7 to 4.85)	3.75 (2.74 to 4.93)	3.72 (2.72 to 4.91)	3.73 (2.65 to 4.97)	3.53 (2.49 to 4.67)	3.41 (2.36 to 4.52)
**Delaware**	9.92% (-8.32% to 31.58%)	2.27 (1.63 to 2.99)	2.32 (1.68 to 3.06)	2.54 (1.83 to 3.33)	2.45 (1.79 to 3.28)	2.59 (1.86 to 3.42)	2.55 (1.82 to 3.41)	2.5 (1.79 to 3.29)	2.5 (1.76 to 3.3)
**District of Columbia**	-15.94% (-30.08% to 0.61%)	1.86 (1.34 to 2.45)	1.77 (1.25 to 2.35)	1.76 (1.26 to 2.3)	1.73 (1.23 to 2.25)	1.49 (1.06 to 1.94)	1.47 (1.06 to 1.94)	1.6 (1.14 to 2.11)	1.57 (1.1 to 2.07)
**Florida**	1.32% (-15.38% to 24.07%)	2.31 (1.66 to 3)	2.48 (1.77 to 3.24)	2.69 (1.93 to 3.52)	2.56 (1.82 to 3.4)	2.46 (1.75 to 3.29)	2.44 (1.78 to 3.28)	2.35 (1.68 to 3.12)	2.34 (1.59 to 3.23)
**Georgia**	4.6% (-13.94% to 24.72%)	2.3 (1.64 to 2.99)	2.35 (1.65 to 3.1)	2.47 (1.76 to 3.22)	2.49 (1.76 to 3.3)	2.56 (1.86 to 3.41)	2.5 (1.83 to 3.34)	2.41 (1.73 to 3.23)	2.4 (1.66 to 3.22)
**Hawaii**	3.33% (-14.51% to 24.78%)	1.76 (1.24 to 2.35)	1.9 (1.34 to 2.52)	1.9 (1.32 to 2.51)	1.96 (1.4 to 2.59)	1.95 (1.39 to 2.63)	1.96 (1.43 to 2.59)	1.89 (1.38 to 2.51)	1.82 (1.3 to 2.42)
**Idaho**	3.82% (-17.09% to 25.43%)	2.64 (1.91 to 3.58)	2.83 (2.01 to 3.76)	2.93 (2.1 to 3.87)	2.84 (2.01 to 3.77)	2.94 (2.11 to 3.92)	2.91 (2.07 to 3.92)	2.72 (1.93 to 3.53)	2.74 (1.88 to 3.68)
**Illinois**	6.44% (-11.24% to 27.96%)	2.38 (1.73 to 3.14)	2.44 (1.77 to 3.2)	2.56 (1.82 to 3.44)	2.45 (1.76 to 3.25)	2.57 (1.83 to 3.41)	2.63 (1.89 to 3.44)	2.52 (1.76 to 3.39)	2.53 (1.77 to 3.46)
**Indiana**	3.82% (-14.39% to 24.18%)	2.6 (1.85 to 3.39)	2.75 (1.99 to 3.56)	2.81 (1.99 to 3.7)	2.75 (1.95 to 3.62)	2.74 (1.98 to 3.56)	2.89 (2.06 to 3.79)	2.73 (1.93 to 3.62)	2.7 (1.87 to 3.58)
**Iowa**	9.93% (-11.33% to 34.85%)	2.51 (1.83 to 3.29)	2.69 (1.97 to 3.49)	2.89 (2.09 to 3.79)	2.88 (2.07 to 3.78)	2.95 (2.16 to 3.9)	2.93 (2.11 to 3.88)	2.87 (2.03 to 3.78)	2.76 (1.93 to 3.85)
**Kansas**	8.33% (-13.78% to 32.23%)	2.82 (2.06 to 3.74)	3.03 (2.15 to 4.04)	3.07 (2.21 to 4.03)	3.12 (2.27 to 4.13)	3.17 (2.28 to 4.14)	3.14 (2.28 to 4.1)	3.09 (2.19 to 4.06)	3.06 (2.15 to 4.01)
**Kentucky**	4.36% (-14.89% to 27.62%)	3.15 (2.29 to 4.23)	3.36 (2.38 to 4.52)	3.45 (2.47 to 4.54)	3.3 (2.34 to 4.36)	3.49 (2.46 to 4.59)	3.49 (2.48 to 4.53)	3.3 (2.38 to 4.3)	3.29 (2.28 to 4.37)
**Louisiana**	11.97% (-7.89% to 35.88%)	2.44 (1.72 to 3.2)	2.57 (1.84 to 3.44)	2.82 (2.02 to 3.73)	3.09 (2.24 to 4.04)	2.82 (2.06 to 3.7)	2.81 (2.03 to 3.77)	2.7 (1.92 to 3.55)	2.73 (1.91 to 3.72)
**Maine**	10.26% (-10.75% to 35.13%)	2.64 (1.89 to 3.51)	2.76 (2.01 to 3.74)	3.02 (2.15 to 4.03)	3.04 (2.21 to 4.01)	2.96 (2.1 to 3.88)	3.01 (2.1 to 3.95)	2.91 (2.09 to 3.89)	2.91 (2.03 to 4.04)
**Maryland**	-3.15% (-20.97% to 17.02%)	2.41 (1.7 to 3.22)	2.41 (1.72 to 3.19)	2.47 (1.76 to 3.33)	2.4 (1.73 to 3.24)	2.4 (1.74 to 3.22)	2.54 (1.85 to 3.44)	2.34 (1.64 to 3.18)	2.33 (1.63 to 3.25)

© 2025 GBD 2021 US CNS Cancer Collaborators. *JAMA Neurology*.

**Massachusetts**	-2.78% (-21.04% to 19.04%)	2.83 (2.04 to 3.74)	2.89 (2.08 to 3.79)	3.26 (2.33 to 4.27)	3.09 (2.22 to 4.09)	3.06 (2.22 to 4.06)	3.38 (2.47 to 4.47)	2.86 (2.05 to 3.85)	2.75 (1.89 to 3.75)
**Michigan**	1.92% (-17.34% to 22.68%)	2.57 (1.82 to 3.35)	2.63 (1.91 to 3.45)	2.79 (2.01 to 3.7)	2.82 (2.02 to 3.68)	2.87 (2.09 to 3.8)	2.75 (1.96 to 3.6)	2.62 (1.92 to 3.46)	2.62 (1.85 to 3.57)
**Minnesota**	4.19% (-16.7% to 26.99%)	3.02 (2.16 to 3.95)	3.13 (2.25 to 4.11)	3.47 (2.52 to 4.67)	3.08 (2.26 to 4.16)	3.33 (2.4 to 4.45)	3.32 (2.4 to 4.49)	3.19 (2.28 to 4.21)	3.14 (2.15 to 4.22)
**Mississippi**	14.69% (-5.57% to 39.78%)	2.48 (1.82 to 3.22)	2.67 (1.9 to 3.51)	2.8 (2.04 to 3.68)	2.7 (1.96 to 3.6)	2.68 (1.92 to 3.55)	2.79 (2.02 to 3.69)	2.84 (2.03 to 3.78)	2.85 (1.93 to 3.79)
**Missouri**	8.17% (-10.81% to 33.33%)	2.58 (1.82 to 3.44)	2.73 (2 to 3.6)	2.85 (2.05 to 3.81)	2.79 (1.97 to 3.64)	2.8 (2.04 to 3.67)	2.8 (2.04 to 3.61)	2.8 (1.98 to 3.71)	2.79 (1.98 to 3.7)
**Montana**	14.16% (-4.15% to 36.54%)	2.47 (1.75 to 3.31)	2.62 (1.83 to 3.49)	2.9 (2.11 to 3.81)	2.89 (2.12 to 3.86)	3.05 (2.24 to 4.07)	3.03 (2.17 to 4.1)	2.85 (2.07 to 3.78)	2.82 (2.03 to 3.89)
**Nebraska**	9.18% (-9.66% to 32.68%)	2.96 (2.1 to 3.95)	3.08 (2.23 to 4.05)	3.28 (2.36 to 4.35)	3.22 (2.3 to 4.23)	3.27 (2.36 to 4.36)	3.27 (2.35 to 4.2)	3.27 (2.32 to 4.35)	3.23 (2.27 to 4.33)
**Nevada**	2.73% (-13.06% to 22.71%)	2.22 (1.58 to 2.9)	2.3 (1.6 to 3.04)	2.39 (1.72 to 3.19)	2.48 (1.77 to 3.27)	2.45 (1.79 to 3.27)	2.5 (1.82 to 3.33)	2.29 (1.62 to 3.04)	2.28 (1.6 to 2.98)
**New Hampshire**	10.71% (-8.63% to 32.66%)	2.54 (1.82 to 3.33)	2.7 (1.99 to 3.51)	2.85 (2.1 to 3.76)	3.01 (2.18 to 3.95)	2.94 (2.13 to 3.95)	3.26 (2.35 to 4.32)	2.87 (2.02 to 3.75)	2.81 (1.94 to 3.78)
**New Jersey**	1.35% (-19.03% to 24.06%)	3.53 (2.52 to 4.64)	3.73 (2.66 to 4.9)	4.02 (2.88 to 5.29)	3.96 (2.85 to 5.19)	3.83 (2.74 to 5.15)	3.97 (2.84 to 5.33)	3.69 (2.72 to 4.86)	3.58 (2.52 to 4.95)
**New Mexico**	24.6% (0.78% to 49.03%)	2.03 (1.49 to 2.74)	2.18 (1.51 to 2.89)	2.23 (1.61 to 2.95)	2.28 (1.59 to 3.03)	2.36 (1.69 to 3.11)	2.46 (1.74 to 3.27)	2.53 (1.76 to 3.34)	2.53 (1.73 to 3.41)
**New York**	3.7% (-14.2% to 25.39%)	2.15 (1.54 to 2.85)	2.23 (1.57 to 2.93)	2.3 (1.62 to 3.06)	2.44 (1.74 to 3.28)	2.39 (1.71 to 3.14)	2.47 (1.8 to 3.31)	2.27 (1.65 to 3.03)	2.23 (1.59 to 3)
**North Carolina**	3.07% (-14.54% to 23.6%)	2.4 (1.68 to 3.15)	2.52 (1.82 to 3.33)	2.58 (1.83 to 3.46)	2.47 (1.81 to 3.25)	2.55 (1.82 to 3.36)	2.54 (1.83 to 3.34)	2.48 (1.69 to 3.37)	2.48 (1.75 to 3.32)
**North Dakota**	5.91% (-14.74% to 29.79%)	2.78 (2 to 3.69)	2.83 (2.03 to 3.68)	3.01 (2.19 to 4.01)	3.01 (2.12 to 3.99)	3.19 (2.3 to 4.28)	3.23 (2.28 to 4.3)	3.12 (2.17 to 4.1)	2.95 (2.02 to 3.91)
**Ohio**	4.83% (-12.61% to 24.92%)	2.52 (1.81 to 3.39)	2.64 (1.91 to 3.47)	2.72 (1.95 to 3.69)	2.67 (1.88 to 3.53)	2.66 (1.92 to 3.53)	2.79 (2.05 to 3.71)	2.64 (1.87 to 3.54)	2.64 (1.86 to 3.51)
**Oklahoma**	0.64% (-17.47% to 20.73%)	2.55 (1.79 to 3.39)	2.68 (1.96 to 3.52)	2.7 (1.95 to 3.57)	2.63 (1.9 to 3.46)	2.7 (1.98 to 3.52)	2.73 (1.96 to 3.53)	2.55 (1.8 to 3.38)	2.56 (1.77 to 3.46)
**Oregon**	-0.79% (-17.66% to 23.31%)	2.99 (2.12 to 3.97)	3.22 (2.29 to 4.23)	3.17 (2.3 to 4.14)	3.29 (2.36 to 4.32)	3.13 (2.24 to 4.11)	3.1 (2.23 to 4.04)	2.96 (2.11 to 3.94)	2.97 (2.08 to 4.13)
**Pennsylvania**	1.48% (-16.41% to 23.23%)	2.59 (1.89 to 3.43)	2.58 (1.86 to 3.47)	2.77 (2.01 to 3.71)	2.71 (1.93 to 3.63)	2.68 (1.97 to 3.54)	2.8 (2.02 to 3.72)	2.66 (1.89 to 3.53)	2.62 (1.79 to 3.68)
**Rhode Island**	-3.81% (-22.38% to 19.44%)	2.69 (1.92 to 3.54)	2.63 (1.89 to 3.48)	3.06 (2.23 to 4.13)	2.96 (2.12 to 3.95)	2.93 (2.12 to 3.93)	2.98 (2.17 to 3.87)	2.69 (1.87 to 3.6)	2.58 (1.84 to 3.58)
**South Carolina**	12.35% (-8.84% to 34.03%)	2.41 (1.73 to 3.21)	2.46 (1.78 to 3.29)	2.57 (1.8 to 3.36)	2.54 (1.81 to 3.39)	2.57 (1.88 to 3.33)	2.62 (1.9 to 3.45)	2.7 (1.9 to 3.62)	2.7 (1.89 to 3.66)
**South Dakota**	7.95% (-10.8% to 29.26%)	2.85 (2.07 to 3.81)	2.98 (2.19 to 3.87)	3.41 (2.47 to 4.51)	3.15 (2.31 to 4.22)	3.37 (2.45 to 4.41)	3.48 (2.49 to 4.6)	3.11 (2.21 to 4.1)	3.08 (2.24 to 4.05)
**Tennessee**	8.54% (-10.86% to 30.17%)	2.59 (1.84 to 3.41)	2.8 (2.02 to 3.71)	2.81 (2 to 3.65)	2.78 (1.97 to 3.62)	2.79 (1.99 to 3.64)	2.75 (1.96 to 3.66)	2.81 (1.99 to 3.82)	2.81 (2.02 to 3.74)
**Texas**	-5.99% (-21.89% to 12.6%)	2.53 (1.82 to 3.31)	2.55 (1.83 to 3.35)	2.62 (1.89 to 3.42)	2.48 (1.79 to 3.32)	2.49 (1.77 to 3.27)	2.53 (1.81 to 3.39)	2.35 (1.66 to 3.1)	2.38 (1.68 to 3.14)
**Utah**	4.11% (-13.78% to 26.37%)	2.42 (1.74 to 3.32)	2.62 (1.83 to 3.45)	2.76 (1.96 to 3.6)	2.73 (1.93 to 3.58)	2.79 (2.04 to 3.66)	2.79 (2 to 3.69)	2.54 (1.8 to 3.33)	2.52 (1.77 to 3.37)

© 2025 GBD 2021 US CNS Cancer Collaborators. *JAMA Neurology*.

**Vermont**	17.56% (-2.97% to 40.76%)	2.36 (1.66 to 3.14)	2.54 (1.8 to 3.4)	2.7 (1.95 to 3.57)	2.83 (2.02 to 3.87)	2.73 (1.99 to 3.66)	2.84 (2.05 to 3.79)	2.87 (2.05 to 3.85)	2.78 (1.95 to 3.79)
**Virginia**	8.13% (-10.68% to 31.3%)	2.4 (1.73 to 3.16)	2.48 (1.77 to 3.29)	2.58 (1.86 to 3.39)	2.62 (1.86 to 3.5)	2.63 (1.92 to 3.51)	2.65 (1.92 to 3.56)	2.59 (1.88 to 3.43)	2.59 (1.82 to 3.51)
**Washington**	-4.32% (-21.5% to 16.23%)	2.6 (1.86 to 3.44)	2.61 (1.85 to 3.42)	2.86 (1.98 to 3.79)	2.72 (2.01 to 3.61)	2.64 (1.84 to 3.54)	2.56 (1.85 to 3.39)	2.51 (1.81 to 3.32)	2.49 (1.7 to 3.34)
**West Virginia**	25.79% (4.48% to 53.39%)	2.21 (1.61 to 2.93)	2.45 (1.78 to 3.23)	2.56 (1.84 to 3.39)	2.61 (1.92 to 3.47)	2.69 (1.96 to 3.58)	2.81 (2.05 to 3.8)	2.78 (2.02 to 3.71)	2.78 (1.95 to 3.71)
**Wisconsin**	1.36% (-18.51% to 21.69%)	2.93 (2.07 to 3.84)	2.97 (2.15 to 3.89)	3.17 (2.31 to 4.18)	3.07 (2.22 to 4.04)	2.99 (2.15 to 4.02)	3.09 (2.25 to 4.16)	3.01 (2.18 to 4.05)	2.97 (2.11 to 4.08)
**Wyoming**	7.03% (-10.37% to 26.07%)	2.48 (1.78 to 3.25)	2.63 (1.88 to 3.49)	2.86 (2.09 to 3.73)	2.6 (1.89 to 3.42)	2.84 (2.03 to 3.78)	2.91 (2.15 to 3.83)	2.7 (1.96 to 3.59)	2.65 (1.87 to 3.55)

© 2025 GBD 2021 US CNS Cancer Collaborators. *JAMA Neurology*.

eTable 9f: Age-standardized YLL rate of Brain and central nervous system cancer by state and year, and percentage change from 1990 to 2021, United States

	Percentage Change, 1990-2021	1990	1995	2000	2005	2010	2015	2020	2021
**United States of America**	-16.06% (-18.03% to -14%)	156.99 (154.18 to 159.12)	152.87 (150.32 to 154.53)	149.09 (146.1 to 151.25)	140.33 (137.17 to 142.31)	135.58 (132.32 to 137.8)	138.86 (135.39 to 140.96)	132.74 (128.82 to 135.47)	131.78 (127.25 to 134.95)
**Alabama**	-2.43% (-14.76% to 12.41%)	181.78 (175.28 to 188.63)	188.04 (180.94 to 195.31)	184.62 (177.11 to 191.11)	175.68 (169.41 to 182.02)	188.66 (181.93 to 195.52)	190.68 (183.53 to 197.69)	178.17 (166.82 to 190.25)	177.36 (154.7 to 202.81)
**Alaska**	-0.58% (-16.15% to 21.73%)	143.9 (137.03 to 151.22)	140.27 (133.41 to 147.7)	135.65 (127.94 to 143.88)	133.31 (125.97 to 141.01)	136.59 (130.34 to 143.87)	136.89 (130.55 to 144.4)	149.52 (136.67 to 164.11)	143.06 (121.65 to 173.3)
**Arizona**	-8.96% (-21.22% to 4.29%)	143.37 (137.99 to 149.1)	144.36 (138.83 to 150.15)	143.18 (137.05 to 149.52)	139.68 (134.02 to 145.49)	128.63 (123.15 to 133.73)	127.99 (122.39 to 133)	131.61 (122 to 141.71)	130.52 (113.17 to 149.8)
**Arkansas**	-5.57% (-18.77% to 8.88%)	183.68 (176.35 to 191.53)	182.15 (174.72 to 190.21)	183.29 (175.85 to 191.56)	169.58 (162.8 to 176.72)	171.86 (164.7 to 179.26)	183.43 (176.26 to 190.82)	172.98 (161.74 to 185.96)	173.45 (150.62 to 198.45)
**California**	-22.46% (-31.81% to -12.43%)	150.57 (146.06 to 154.5)	142.93 (138.66 to 147.14)	137.46 (132.74 to 142.03)	127.85 (123.05 to 131.8)	118.52 (113.97 to 123.22)	121.94 (117.17 to 126.14)	116.77 (107.31 to 124.91)	116.75 (103.33 to 131.44)
**Colorado**	-16.46% (-29.8% to -3.14%)	150.91 (144.83 to 156.8)	150.19 (144.42 to 156.62)	141.78 (135.95 to 147.41)	133.91 (128.18 to 139.85)	131.2 (125.67 to 136.17)	129.5 (124.03 to 134.59)	125.93 (117.22 to 135.44)	126.08 (107.21 to 145.48)
**Connecticut**	-18.44% (-31.61% to -4.41%)	148.25 (142.47 to 155.24)	146.37 (139.92 to 152.04)	142.28 (135.58 to 148.6)	132.8 (126.98 to 138.43)	128.67 (123.15 to 134.28)	129.81 (124.03 to 136.15)	124.31 (114.01 to 134.07)	120.91 (100.82 to 140.72)
**Delaware**	-13.26% (-23.64% to -2.57%)	147.07 (139.92 to 154.45)	141.9 (135.61 to 148.18)	145.11 (137.98 to 152.25)	133.67 (127.6 to 140.85)	132.87 (127.14 to 139.55)	129.19 (122.58 to 135)	127.45 (120.07 to 136.38)	127.57 (114.14 to 142.32)
**District of Columbia**	-43.53% (-52.01% to -34.23%)	175.73 (165.91 to 185.65)	154.73 (145.98 to 164.7)	135.62 (127.3 to 144.64)	121.78 (114.47 to 130.22)	100.4 (94.69 to 106.97)	95.26 (88.51 to 101.39)	101.43 (92.83 to 111.54)	99.24 (85.67 to 113.89)
**Florida**	-16.81% (-27.56% to -4.4%)	159.26 (153.69 to 165.1)	158.89 (154.35 to 164.1)	152.55 (147.32 to 157.52)	141.11 (136.46 to 145.57)	133.46 (128.86 to 137.79)	135.34 (130.18 to 140.29)	133.44 (124.23 to 141.28)	132.49 (114.99 to 152.14)
**Georgia**	-18.31% (-28.21% to -6.46%)	161.74 (156.52 to 166.91)	152.84 (147.49 to 157.81)	146.72 (141.49 to 152.2)	136.98 (132.35 to 141.55)	134.68 (130.23 to 138.89)	138.02 (132.83 to 142.92)	132.39 (124.58 to 140.68)	132.13 (115.67 to 150.71)
**Hawaii**	-14.83% (-27.08% to -1.4%)	102.27 (97.82 to 107.03)	103.37 (98.08 to 108.34)	96.68 (92.23 to 101.58)	96.28 (91.11 to 101.15)	94.17 (89.44 to 98.78)	94 (89.93 to 98.92)	90.24 (84.88 to 95.64)	87.1 (74.67 to 100.93)
**Idaho**	-12.15% (-25.63% to 2.49%)	158.42 (150.63 to 167)	160.15 (151.53 to 168.87)	159.37 (150.94 to 168.31)	150.88 (143.79 to 158.61)	147.96 (140.1 to 156.01)	149.79 (142.52 to 157.1)	139.22 (127.81 to 148.56)	139.17 (118.2 to 161.06)
**Illinois**	-19.04% (-30.29% to -7.11%)	153.67 (148.18 to 159.36)	148.19 (142.96 to 152.97)	143.8 (138.7 to 148.9)	127.63 (123.47 to 132.07)	127.96 (123 to 132.67)	129.99 (124.58 to 134.81)	124.5 (115.53 to 133.89)	124.41 (107.63 to 142.16)

© 2025 GBD 2021 US CNS Cancer Collaborators. *JAMA Neurology*.

**Indiana**	-7.5% (-20.09% to 6.31%)	158.66 (153.3 to 165.22)	161.99 (155.13 to 167.89)	159.06 (153.05 to 165.41)	153.55 (148.15 to 159.01)	149.14 (144.01 to 154.15)	159.37 (152.81 to 165.49)	148.19 (137.91 to 157.75)	146.76 (128.19 to 167.25)
**Iowa**	-3.21% (-18.11% to 12.21%)	153.86 (146.48 to 161.29)	159.56 (152.74 to 166.94)	158.97 (151.61 to 165.77)	152.76 (146.05 to 159.87)	153.78 (146.61 to 160.47)	157.75 (150.74 to 164.45)	155.65 (144.67 to 167.34)	148.91 (127.37 to 170.85)
**Kansas**	-1.82% (-16.85% to 14.15%)	159.04 (152.19 to 166.71)	165 (156.76 to 172.32)	161.27 (153.58 to 169.3)	163.69 (156.4 to 170.82)	161.84 (154.95 to 168.46)	163.79 (157.3 to 171.03)	158.09 (145.78 to 170.08)	156.14 (131.75 to 181.04)
**Kentucky**	-3.01% (-16.67% to 11.64%)	167.14 (161.24 to 173.93)	165.19 (158.93 to 171.57)	160.28 (154.55 to 166.81)	151.9 (146.45 to 157.84)	164.58 (158.79 to 170.69)	171.72 (164.95 to 179.6)	163.55 (152.74 to 174.39)	162.11 (139.09 to 184.79)
**Louisiana**	-2.46% (-15.76% to 12.28%)	159.09 (153.05 to 164.86)	156.71 (150.68 to 162.61)	161.07 (155.37 to 166.88)	169.31 (163.16 to 175.64)	152.18 (147.26 to 157.36)	159.81 (153.75 to 166.2)	154.56 (144.25 to 166.08)	155.17 (135.26 to 177.27)
**Maine**	-2.25% (-17.62% to 13.82%)	149.57 (142.09 to 157.92)	148.1 (139.84 to 157.21)	154.06 (145.71 to 162.29)	149.83 (141.66 to 157.64)	143.93 (136.35 to 151.9)	150.23 (141.97 to 159.56)	147.06 (136.19 to 157.27)	146.21 (126.07 to 169.77)
**Maryland**	-23.95% (-35.68% to -11.53%)	157.84 (150.6 to 164.29)	147.22 (141.3 to 152.99)	142.07 (136.04 to 148.19)	126.8 (121 to 132.43)	120.43 (114.92 to 125.94)	128.02 (122.46 to 134.48)	120.81 (112.35 to 130.19)	120.03 (102.5 to 139.57)
**Massachusetts**	-23.91% (-37% to -9.5%)	155.91 (150.34 to 161.72)	148.15 (142.4 to 153.81)	152.68 (146.31 to 158.85)	136.64 (130.75 to 142.03)	130.32 (125.14 to 135.81)	143.6 (137.48 to 150.96)	123.77 (113.14 to 134.56)	118.63 (99.57 to 140.31)
**Michigan**	-15.1% (-26.6% to -2.31%)	167.14 (161.81 to 172.86)	157.96 (152.57 to 163.51)	158.78 (152.43 to 164.8)	150.41 (144.8 to 155.12)	150.25 (144.8 to 155.5)	148.49 (142.64 to 153.62)	142.85 (133.61 to 153.56)	141.89 (123.39 to 161.69)
**Minnesota**	-15.21% (-28.5% to -1.4%)	156.53 (150.05 to 162.7)	153.79 (146.82 to 160.36)	159.44 (152.87 to 165.8)	134.14 (127.87 to 140.06)	140.38 (134.28 to 147.24)	142.19 (135.85 to 148.72)	134.94 (124.9 to 144.52)	132.72 (112.72 to 153.84)
**Mississippi**	4.71% (-8.86% to 20.14%)	179.68 (173.08 to 186.94)	188.71 (181.48 to 196.63)	194.62 (187.38 to 202.07)	186.44 (179.12 to 193.71)	176.11 (169.75 to 182.6)	186.7 (179.63 to 193.67)	188.85 (176.61 to 202)	188.15 (163.68 to 215.11)
**Missouri**	-6.51% (-20.91% to 8.29%)	160.15 (153.87 to 166.94)	163.87 (157.96 to 170.34)	161.53 (155.34 to 168.04)	154.69 (148.83 to 160.93)	148.77 (143.4 to 154.2)	150.84 (144.63 to 156.75)	150.03 (139.74 to 160.84)	149.72 (128.69 to 172.19)
**Montana**	-1.46% (-14.92% to 14.45%)	148.76 (141.06 to 157.31)	150.16 (143.05 to 157.45)	158.15 (150.35 to 166.77)	153.33 (145.65 to 161.14)	156.61 (148.94 to 165.59)	161.87 (153.27 to 170.02)	148.84 (138.02 to 159.39)	146.59 (128.49 to 168.97)
**Nebraska**	-7.26% (-21.2% to 7.79%)	166.85 (159.05 to 175.84)	166.76 (158.9 to 175.77)	169 (159.95 to 179.08)	156.3 (148.98 to 163.78)	154.71 (147 to 162.12)	159.87 (151.57 to 168.93)	156.19 (144.29 to 167.22)	154.74 (133.5 to 177.77)
**Nevada**	-17.54% (-29.4% to -4.84%)	145.52 (139.28 to 152.07)	146.39 (139.55 to 153.43)	141.92 (134.66 to 148.6)	142.53 (136.05 to 148.66)	131.85 (126.51 to 137.44)	133.48 (127.32 to 139.26)	120.92 (112.53 to 129.63)	120 (103.48 to 137.53)
**New Hampshire**	-11.6% (-25.06% to 3.81%)	145.17 (137.75 to 152.14)	146.91 (139.52 to 154.38)	140.71 (134.07 to 147.54)	138.3 (130.85 to 145.86)	131.52 (124.64 to 137.93)	144.33 (136.55 to 151.74)	130.49 (121.44 to 139.35)	128.33 (109.68 to 149.24)
**New Jersey**	-23.43% (-35.3% to -10.77%)	146.58 (140.97 to 151.75)	140.07 (134.73 to 145.05)	131.42 (126.56 to 136.24)	124.21 (119.51 to 128.87)	117.8 (113.14 to 122.22)	122.3 (116.8 to 126.88)	115.66 (106.22 to 125.36)	112.24 (96.03 to 130.73)

© 2025 GBD 2021 US CNS Cancer Collaborators. *JAMA Neurology*.

**New Mexico**	6.04% (-9.97% to 24.14%)	129.81 (122.18 to 137.25)	129.15 (122.2 to 135.73)	124.88 (117.86 to 131.73)	124.33 (117.12 to 130.78)	122.66 (116.16 to 129.52)	131.91 (124.91 to 138.4)	137.14 (126.39 to 146.82)	137.65 (118.21 to 160.04)
**New York**	-26.16% (-36.25% to -14.75%)	142 (136.75 to 147.71)	134.82 (129.56 to 140.47)	122.49 (117.57 to 126.93)	119.75 (115.19 to 124.48)	112.45 (108.1 to 117.34)	113.55 (108.78 to 118.11)	106.2 (97.39 to 114.67)	104.85 (90.95 to 120.5)
**North Carolina**	-17.7% (-29.55% to -4.93%)	162.44 (156.34 to 168.37)	159.21 (153.06 to 165.75)	154.47 (148.02 to 159.79)	138.58 (133.14 to 144)	136.42 (131.2 to 141.65)	137.18 (131.7 to 142.27)	134.23 (125.32 to 143.23)	133.68 (115.11 to 153.31)
**North Dakota**	-8.64% (-20.74% to 5.47%)	160.26 (153.19 to 168.28)	154.03 (145.57 to 162.1)	153.18 (144.75 to 162.46)	149.94 (141.56 to 158.83)	158.12 (149.55 to 165.63)	164.87 (156.69 to 173.57)	155.82 (145.35 to 167.19)	146.42 (127.34 to 166.15)
**Ohio**	-6.76% (-18.2% to 6.36%)	156.1 (151.07 to 160.98)	156.87 (151.72 to 162.22)	152.86 (147.63 to 158.04)	148.8 (143.93 to 153.31)	144.8 (139.6 to 149.69)	154.53 (149.32 to 159.96)	145.95 (136.2 to 154.95)	145.54 (126.82 to 165.44)
**Oklahoma**	-2.29% (-14.56% to 11.18%)	158.74 (152.72 to 165.57)	162.9 (156.01 to 169.67)	164.41 (157.45 to 171.71)	162.04 (155.44 to 168.19)	163.69 (156.49 to 170.8)	167.62 (160.72 to 174.6)	155.65 (144.37 to 166.68)	155.1 (136.31 to 175.83)
**Oregon**	-20.28% (-31.48% to -6.26%)	171.13 (163.9 to 178.4)	174.18 (167.45 to 181.65)	157.93 (151.33 to 164.3)	158 (151.18 to 164.56)	145.93 (140.04 to 152.28)	145.43 (138.5 to 151.77)	135.77 (127.42 to 144.1)	136.43 (118.49 to 159.74)
**Pennsylvania**	-17.61% (-28.67% to -5.54%)	160.5 (154.49 to 166.35)	149.52 (144.11 to 154.48)	149.3 (143.73 to 154.68)	141.33 (136.17 to 146.6)	136.72 (131.13 to 142.43)	141.44 (135.67 to 146.55)	134.39 (124.9 to 144.88)	132.23 (114.69 to 150.83)
**Rhode Island**	-25.04% (-37.98% to -11.11%)	162.25 (153.41 to 171.13)	149.1 (140.29 to 157.71)	157.1 (147.83 to 165.29)	145.36 (137.13 to 153.45)	138.74 (131.35 to 146.31)	141.01 (132.86 to 149.32)	127 (114.69 to 138.66)	121.62 (102.61 to 142.53)
**South Carolina**	-11.48% (-23.33% to 2.65%)	177.33 (169.8 to 185.69)	166.95 (159.54 to 174.69)	166.67 (159.54 to 174.1)	156 (149.47 to 162.7)	151.7 (145.05 to 158.22)	154.3 (147.55 to 160.85)	157.22 (146.29 to 169.16)	156.98 (135.9 to 179.07)
**South Dakota**	-4.21% (-16.52% to 9.39%)	170.85 (163.36 to 178.89)	170.36 (161.49 to 178.82)	184.16 (173.66 to 193.98)	169.01 (159.98 to 177.95)	175.47 (166.54 to 184.03)	184.93 (175.23 to 194.04)	166.36 (155.1 to 178.39)	163.65 (145.07 to 185.17)
**Tennessee**	-8.25% (-20.72% to 5.71%)	177.26 (169.79 to 184.54)	180.46 (173.46 to 187.58)	173.4 (166.73 to 179.93)	170.6 (164.28 to 176.76)	162.01 (155.88 to 167.82)	161.36 (154.05 to 167.7)	163.32 (152.01 to 174.1)	162.63 (142.02 to 187)
**Texas**	-23.98% (-32.97% to -13.77%)	162.28 (157.21 to 167.94)	154.81 (150.37 to 159.31)	148.1 (143.73 to 153.19)	135.41 (131.03 to 139.67)	129.41 (125.3 to 133.21)	133.73 (129.33 to 138.55)	122.98 (114.73 to 131.71)	123.37 (109.4 to 139.19)
**Utah**	-13.83% (-25.19% to -1.2%)	145.59 (138.55 to 152.6)	150.35 (143.06 to 158.08)	147.44 (140.21 to 154.99)	138.92 (131.7 to 144.63)	137.21 (130.93 to 143.43)	140.37 (133.85 to 146.99)	126.43 (117.38 to 135.16)	125.45 (109.61 to 143.62)
**Vermont**	-10.79% (-20.94% to 1.07%)	147.66 (140.17 to 154.69)	146.67 (139.67 to 154.85)	140.97 (133.58 to 149.69)	134.71 (127.4 to 142.2)	129.33 (122.14 to 137.48)	133.68 (125.66 to 140.7)	135.25 (125.65 to 145.17)	131.73 (118.06 to 148.91)
**Virginia**	-16.03% (-28.06% to -2.45%)	148.41 (142.67 to 154.27)	144.03 (138 to 150.11)	139.94 (133.8 to 146.24)	132.22 (126.18 to 137.85)	126.65 (121.02 to 132.68)	127.44 (121.84 to 132.97)	124.26 (116.01 to 133.08)	124.63 (106.23 to 144.3)
**Washington**	-19.95% (-32.71% to -7.46%)	170.77 (164.12 to 177.98)	157.68 (151.31 to 163.89)	161.3 (154.33 to 168.37)	148.6 (142.45 to 154.54)	142.8 (136.64 to 148.32)	141.27 (135 to 147.03)	137.45 (128.68 to 145.99)	136.7 (115.29 to 156.65)

© 2025 GBD 2021 US CNS Cancer Collaborators. *JAMA Neurology*.

**West Virginia**	15.97% (0.13% to 34.18%)	145.49 (138.85 to 153.34)	154.65 (147.78 to 161.62)	154.97 (148.02 to 161.71)	159.28 (151.75 to 166.34)	161.89 (154.17 to 169.23)	173.14 (165.88 to 181.17)	169.31 (158.52 to 181.55)	168.72 (146.5 to 194.48)
**Wisconsin**	-11.76% (-24.77% to 2.97%)	159.61 (152.7 to 165.78)	156.64 (150.4 to 163.21)	158.35 (152.41 to 164.9)	147.3 (140.64 to 153.02)	142.08 (135.72 to 148.36)	146.6 (140.91 to 152.83)	143.06 (133.06 to 152.71)	140.85 (121.09 to 162.61)
**Wyoming**	-7.92% (-18.12% to 4.33%)	151.51 (144.58 to 159.12)	151.43 (143.86 to 158.76)	160.66 (151.73 to 169.23)	143.47 (136.26 to 151.04)	150.86 (142.97 to 159.41)	154.08 (145.56 to 162.18)	141.5 (131.4 to 151.14)	139.5 (124.67 to 156.52)

© 2025 GBD 2021 US CNS Cancer Collaborators. *JAMA Neurology*.

eAppendix V: Supplementary Figures

(A) Incidence

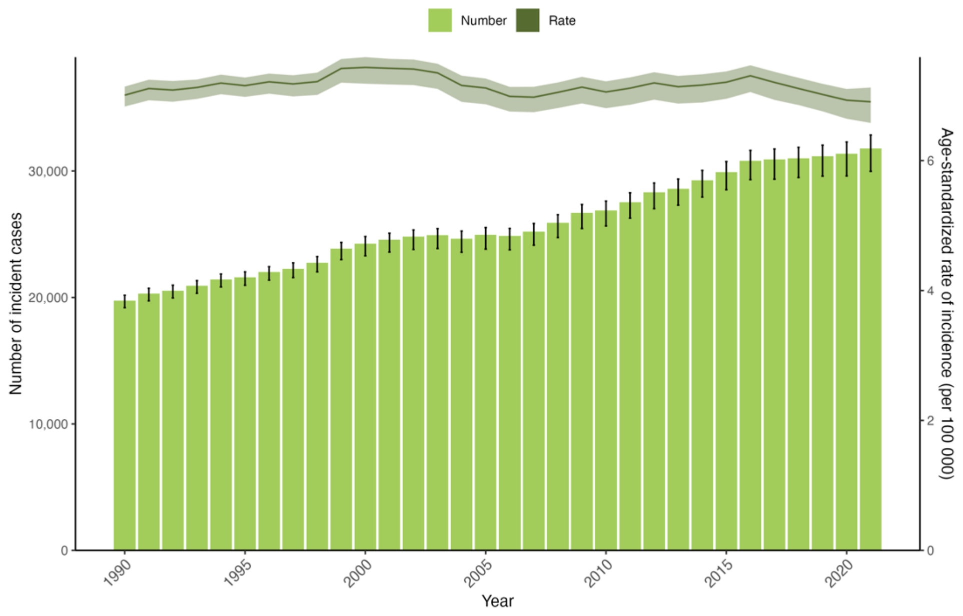

(B) DALYs

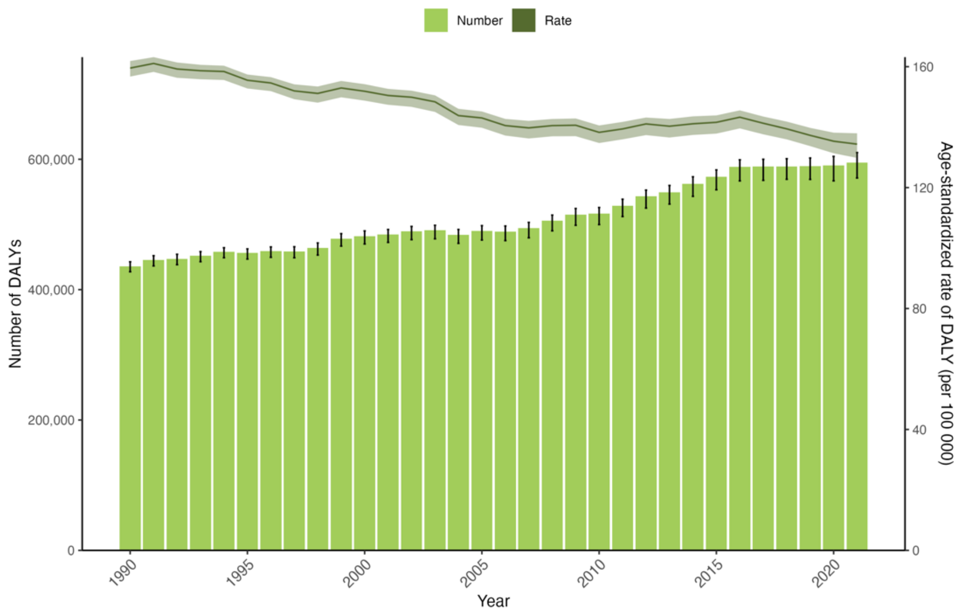

(C) Death

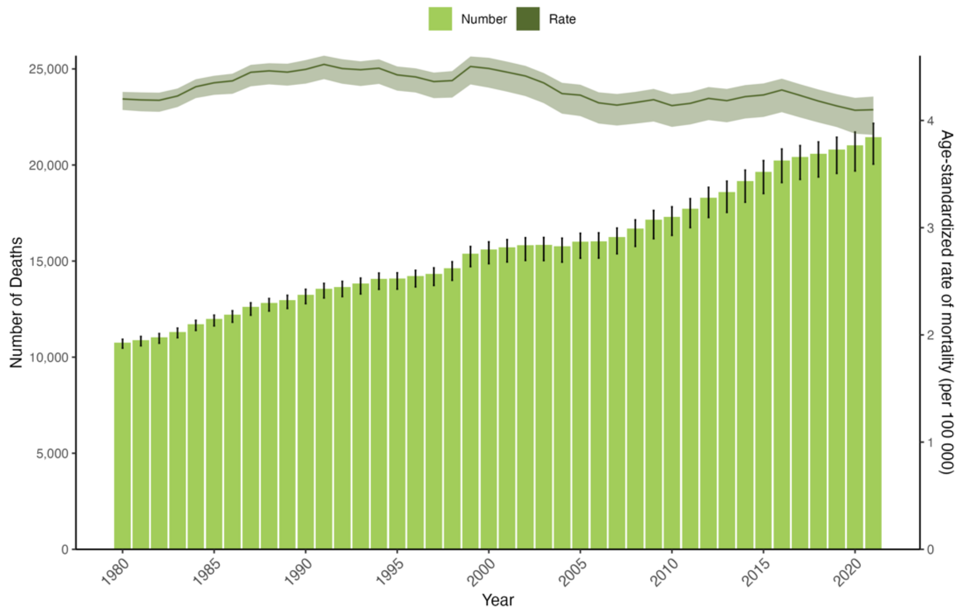

(D) Prevalence

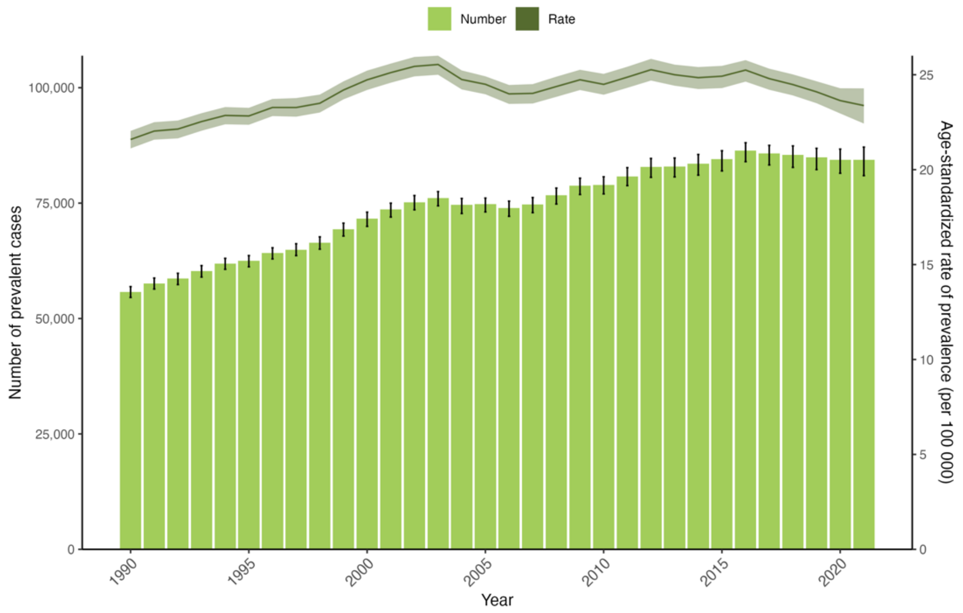

© 2025 GBD 2021 US CNS Cancer Collaborators. *JAMA Neurology*.

(E) YLDs

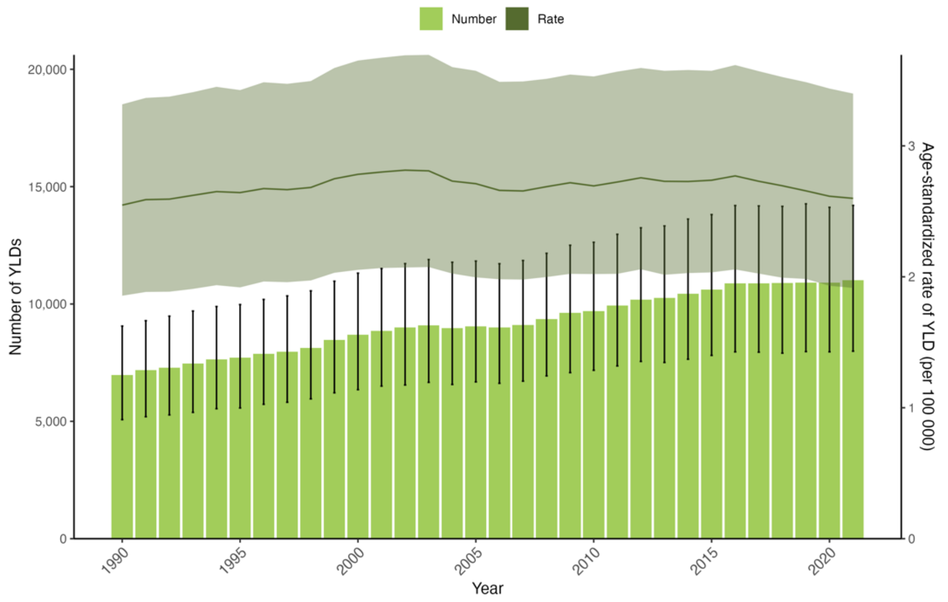

(F) YLLs

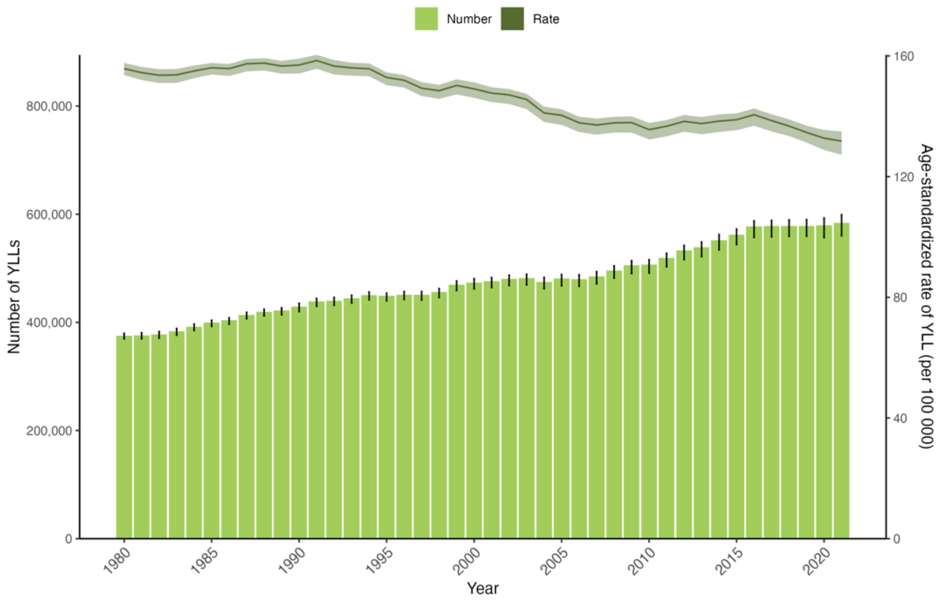

eFigure 1a: (A) incidence (B) DALYs (C) death (D) prevalence (E) YLDs (F) YLLs count and age-standardized rate of Brain and central nervous system cancer in the United States, 1990 to 2021

© 2025 GBD 2021 US CNS Cancer Collaborators. *JAMA Neurology*.

(A) New England

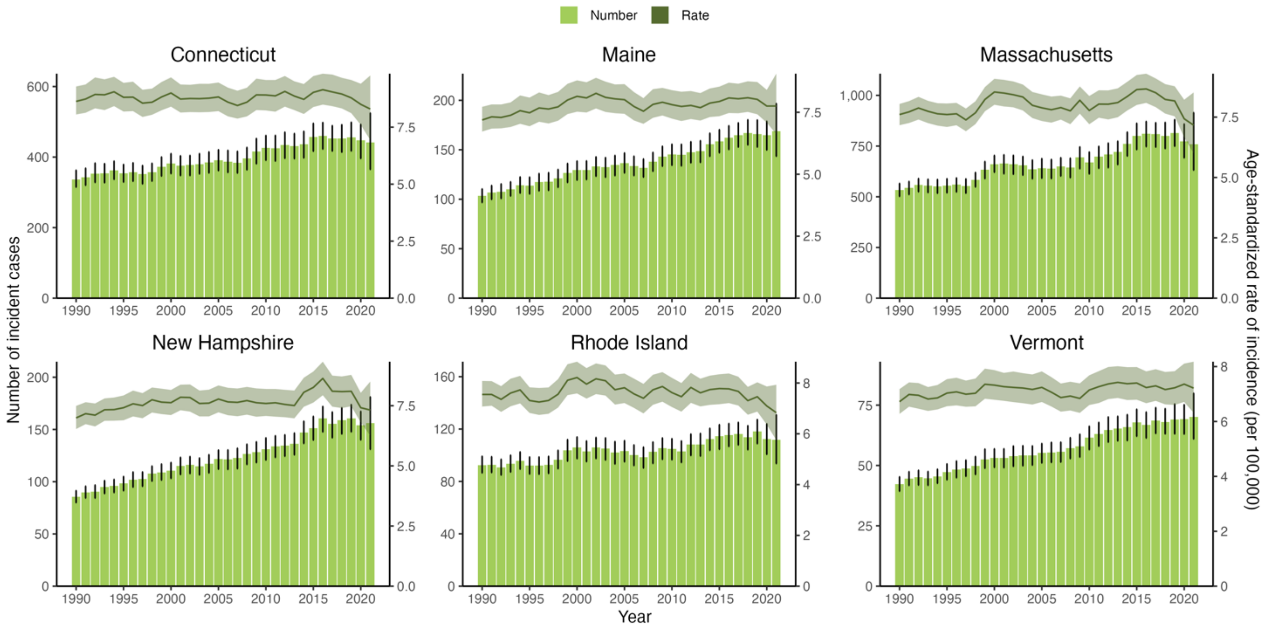

(B) Middle Atlantic

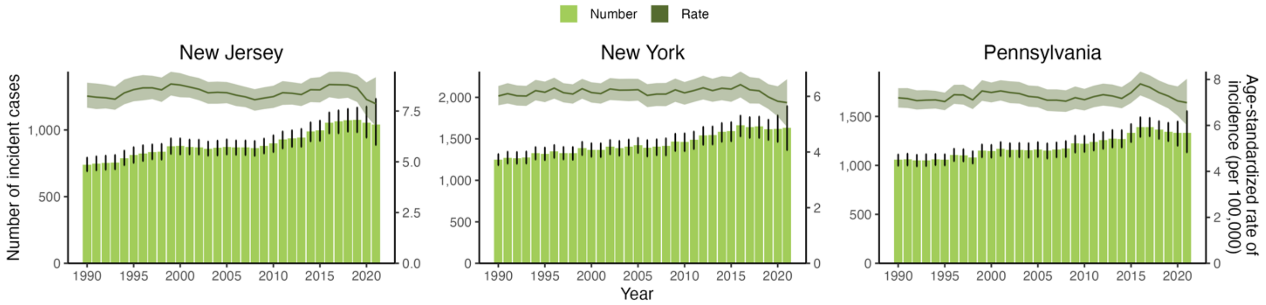

© 2025 GBD 2021 US CNS Cancer Collaborators. *JAMA Neurology*.

(C) East North Central

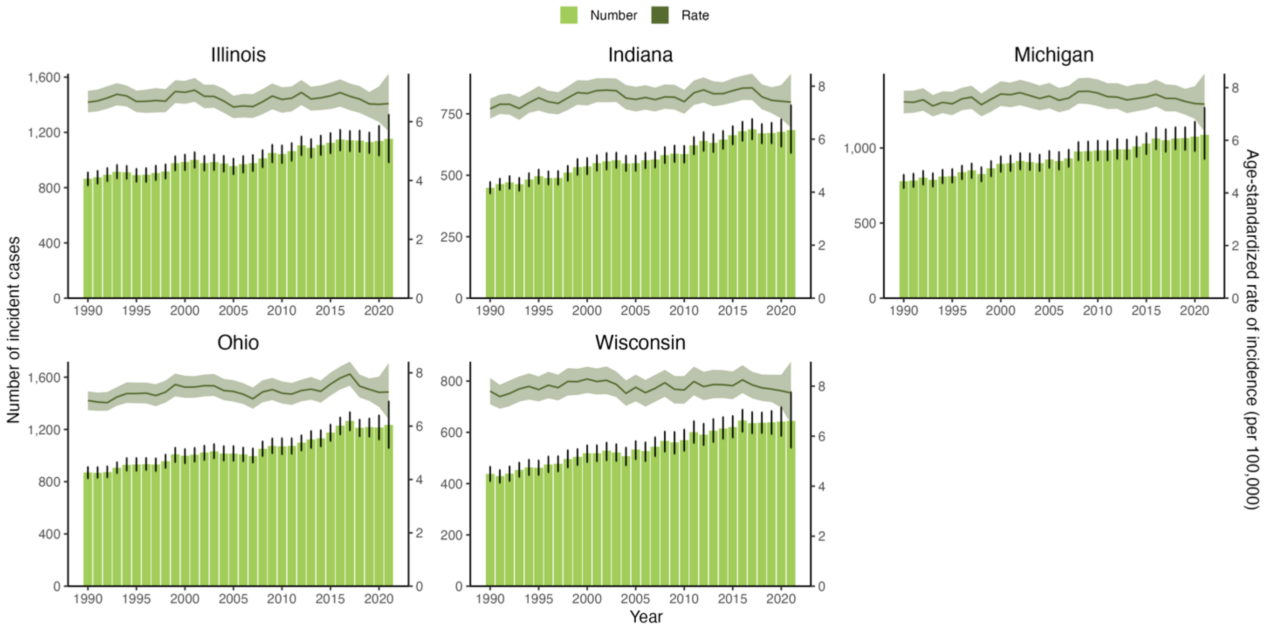

© 2025 GBD 2021 US CNS Cancer Collaborators. *JAMA Neurology*.

(D) West North Central

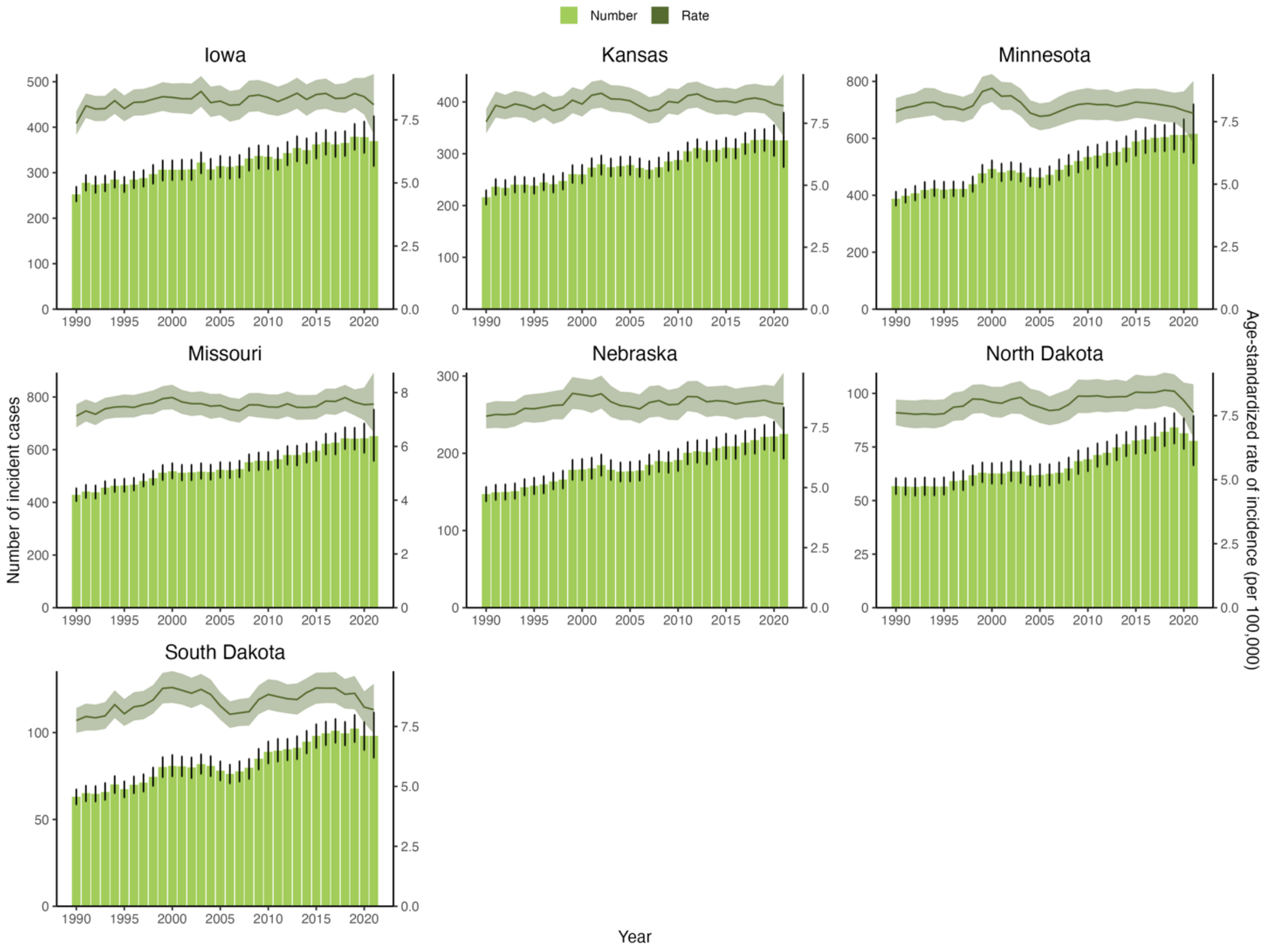

© 2025 GBD 2021 US CNS Cancer Collaborators. *JAMA Neurology*.

(E) South Atlantic

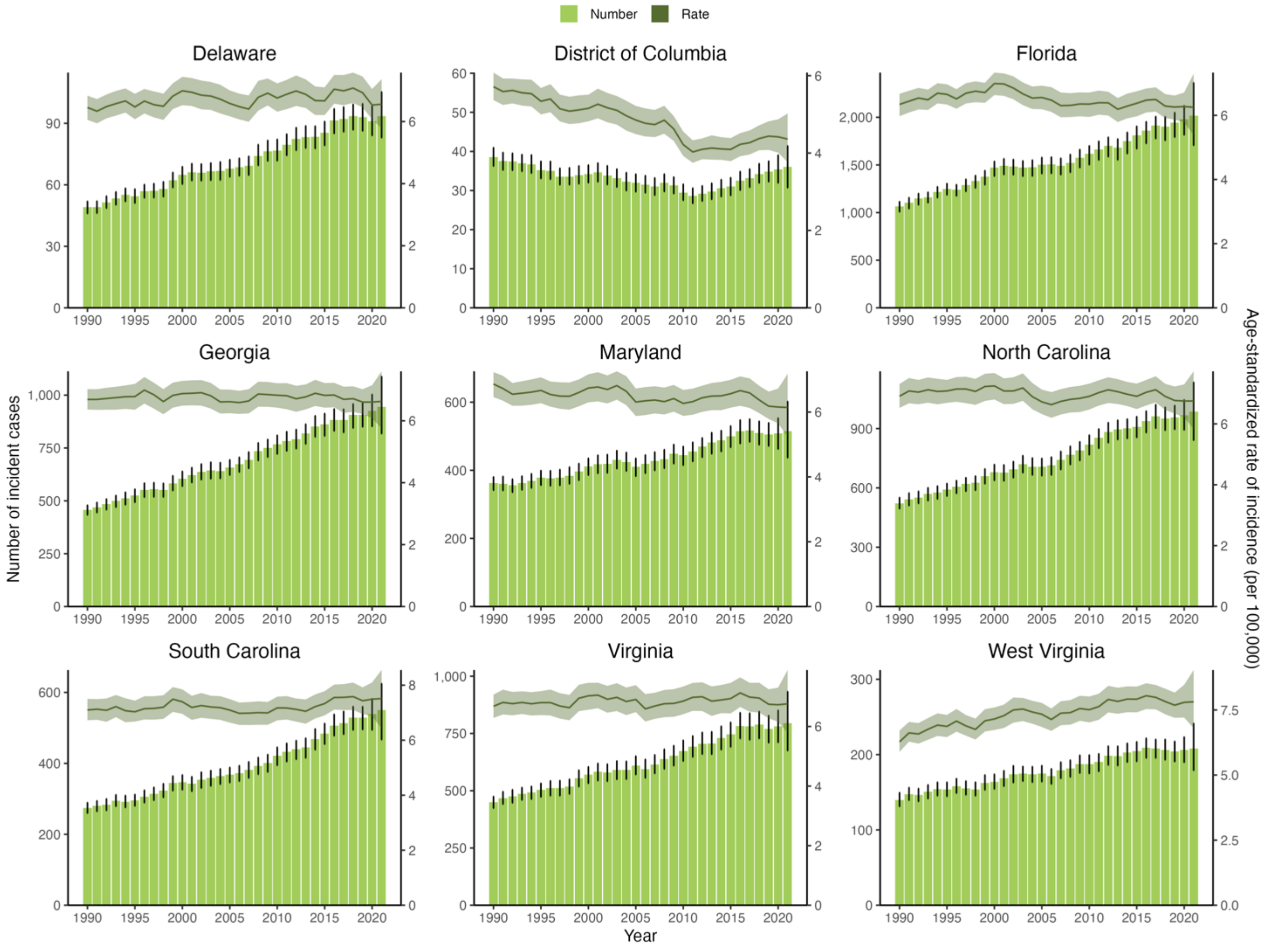

© 2025 GBD 2021 US CNS Cancer Collaborators. *JAMA Neurology*.

(F) East South Central

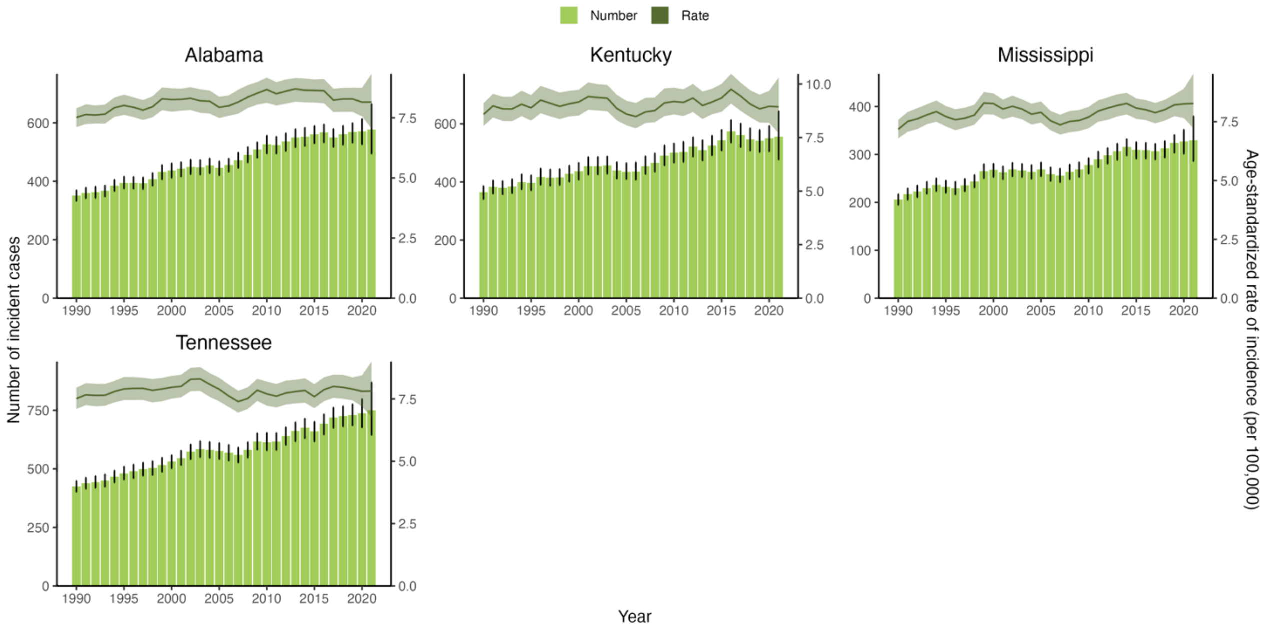

(G) West South Central

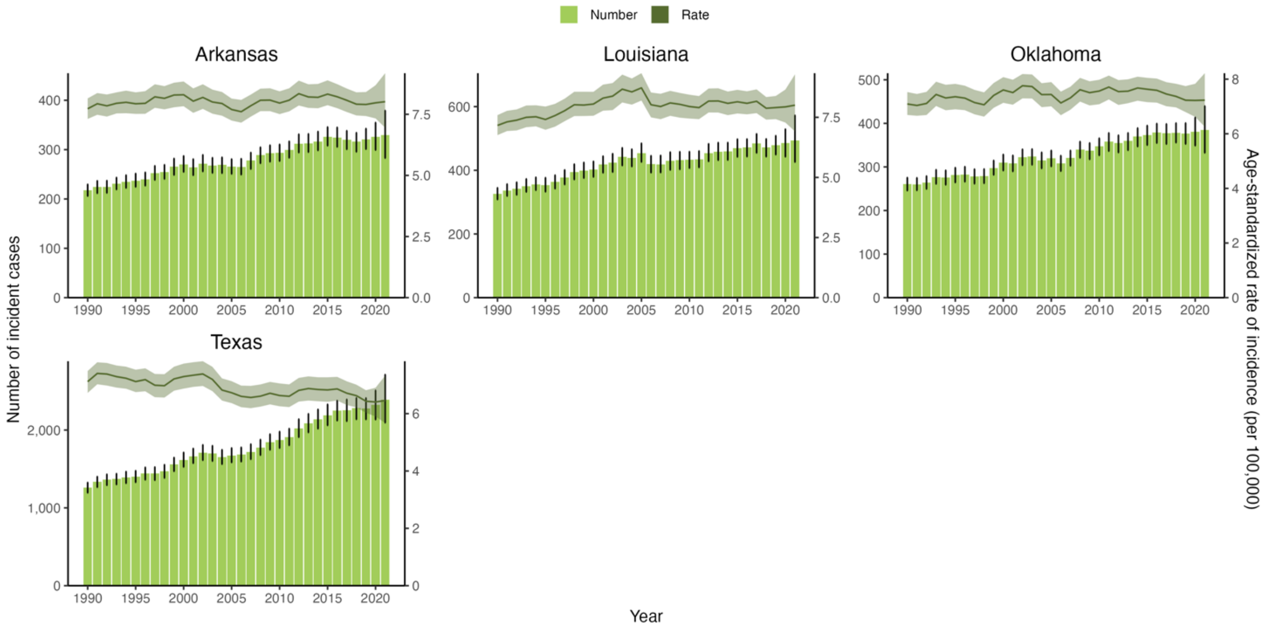

© 2025 GBD 2021 US CNS Cancer Collaborators. *JAMA Neurology*.

(H) Mountain

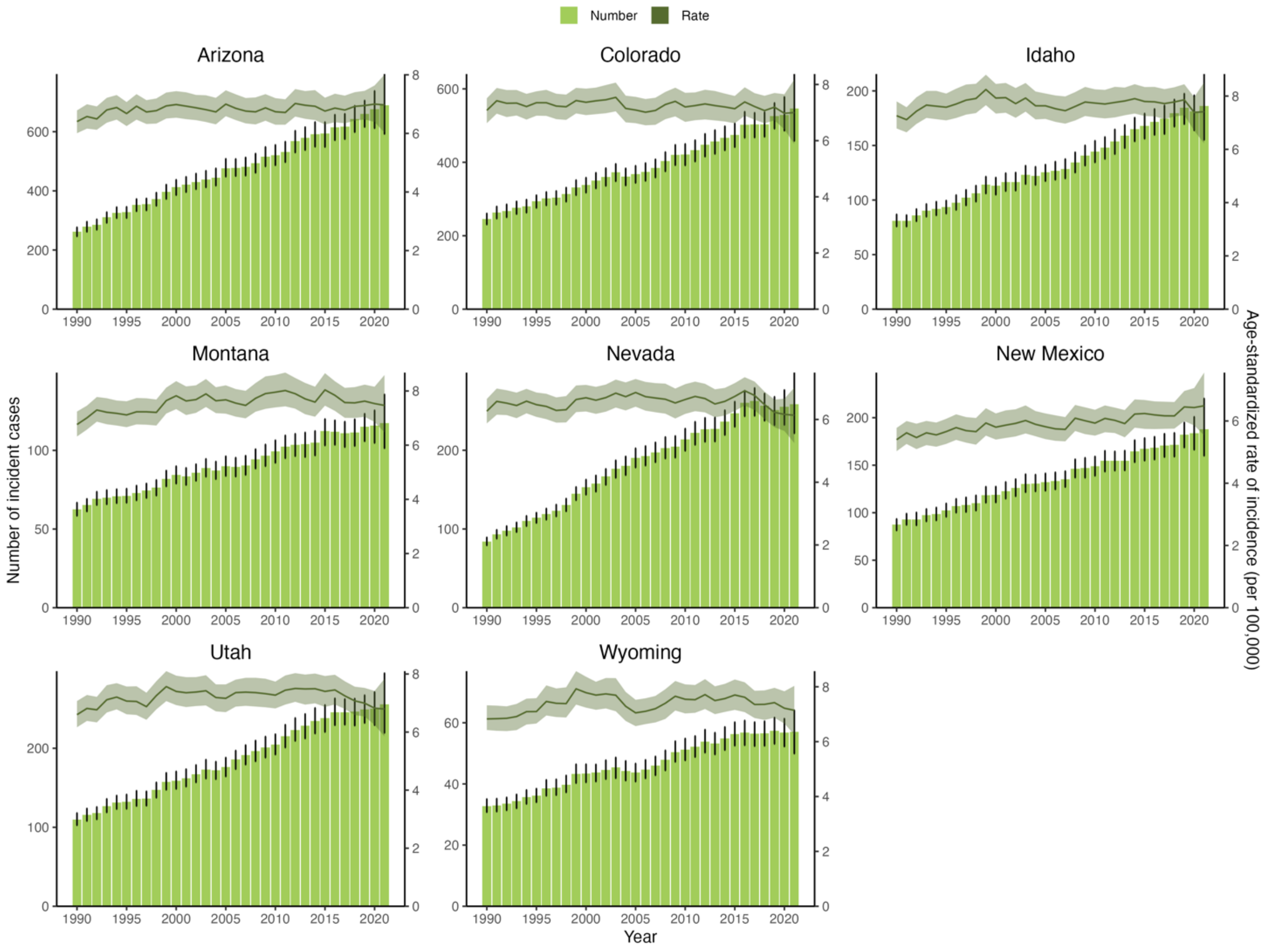

© 2025 GBD 2021 US CNS Cancer Collaborators. *JAMA Neurology*.

(I) Pacific

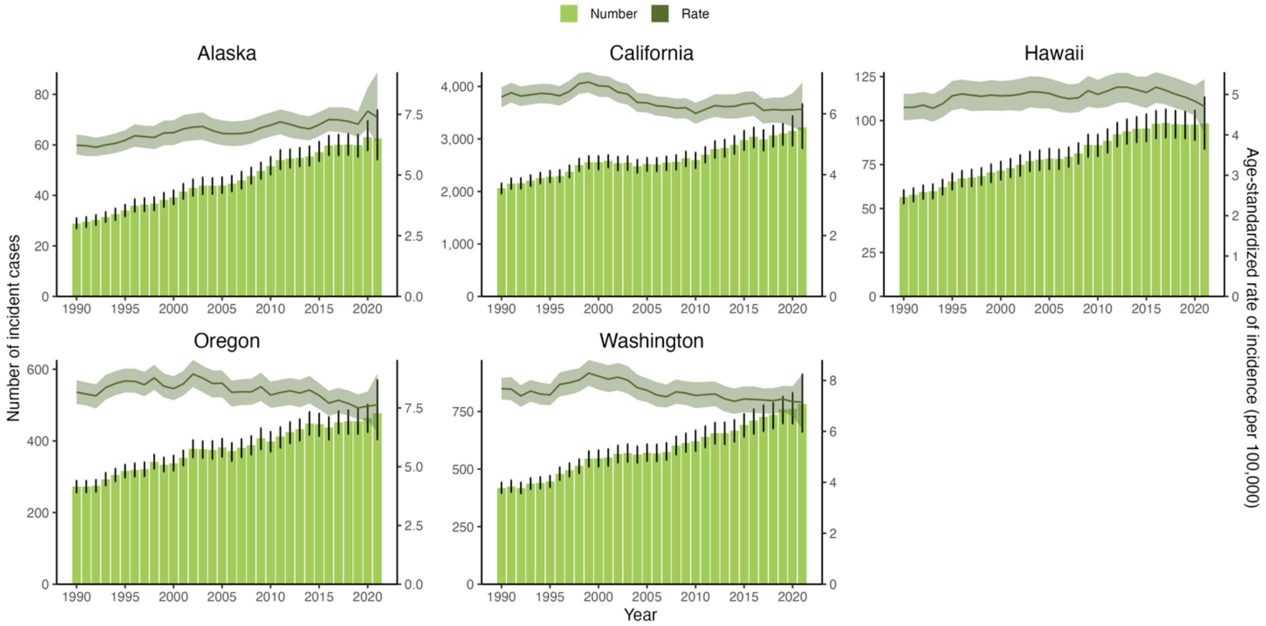

eFigure 1b: Incidence count and age-standardized rate of Brain and central nervous system cancer in the United States by division, (A) New England, (B) Middle Atlantic, (C) East North Central, (D) West North Central, (E) South Atlantic, (F) East South Central, (G) West South Central, (H) Mountain, (I) Pacific division, 1990 to 2021

© 2025 GBD 2021 US CNS Cancer Collaborators. *JAMA Neurology*.

(A) New England

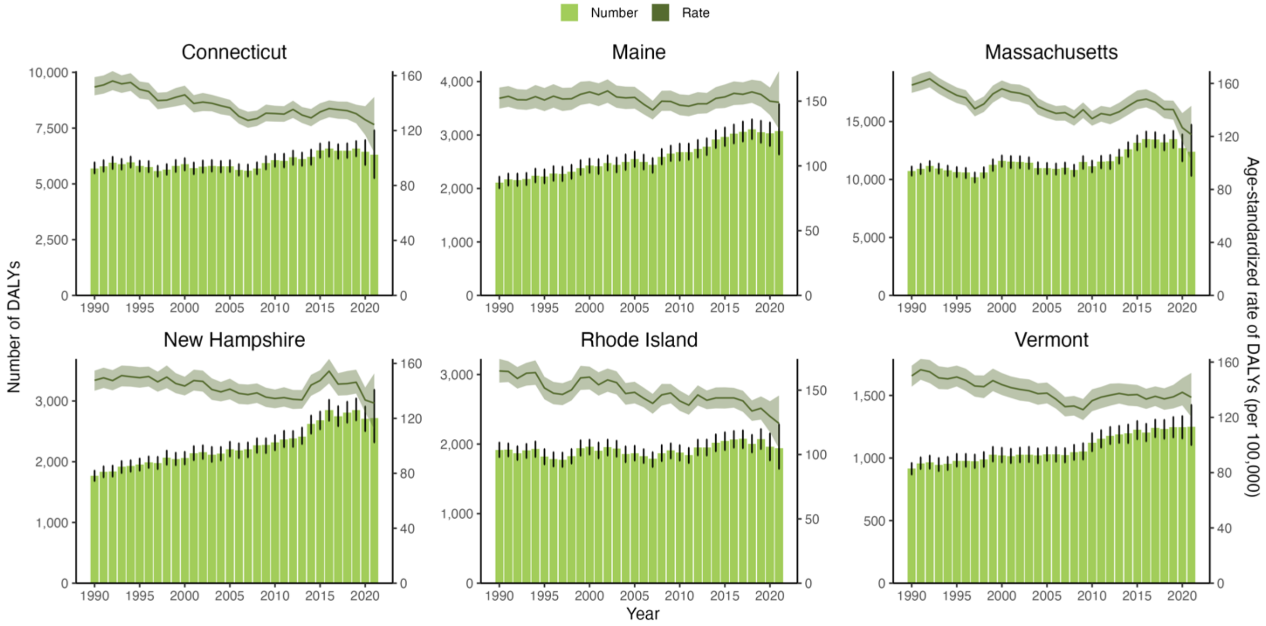

(B) Middle Atlantic

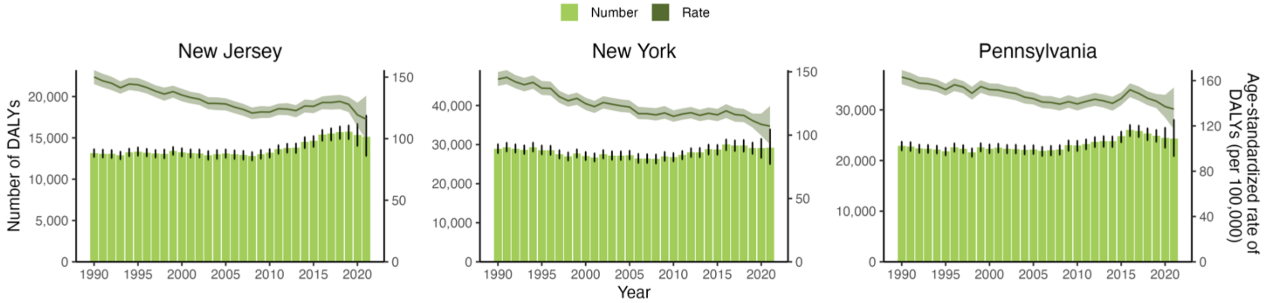

© 2025 GBD 2021 US CNS Cancer Collaborators. *JAMA Neurology*.

(C) East North Central

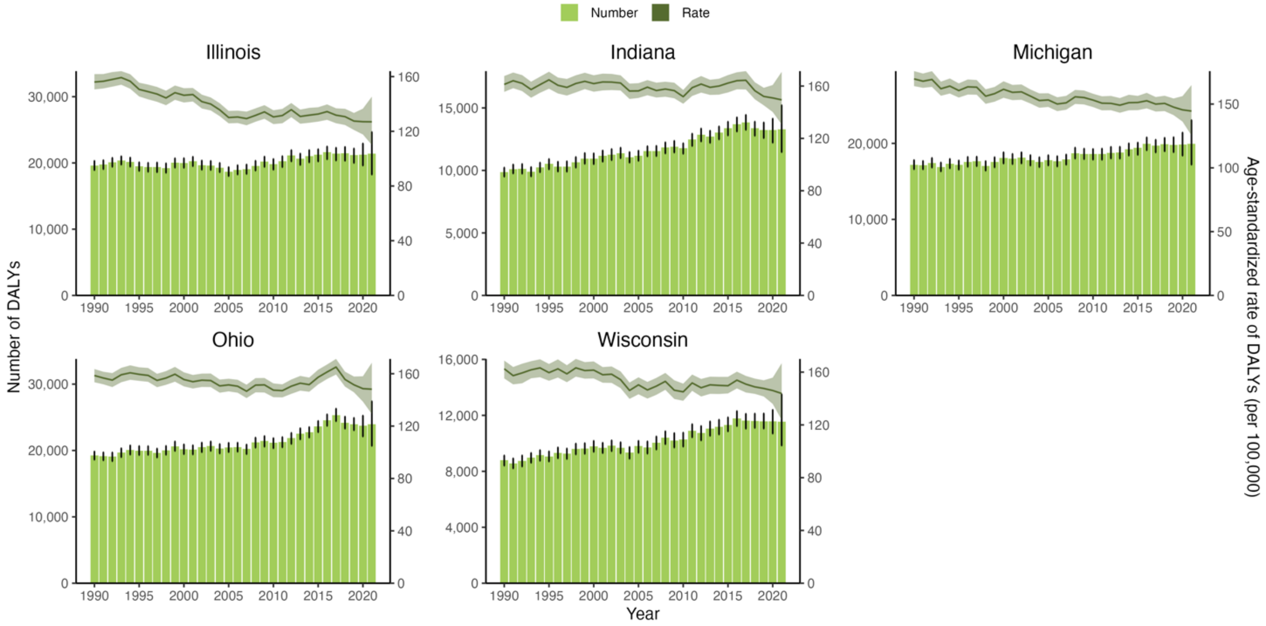

© 2025 GBD 2021 US CNS Cancer Collaborators. *JAMA Neurology*.

(D) West North Central

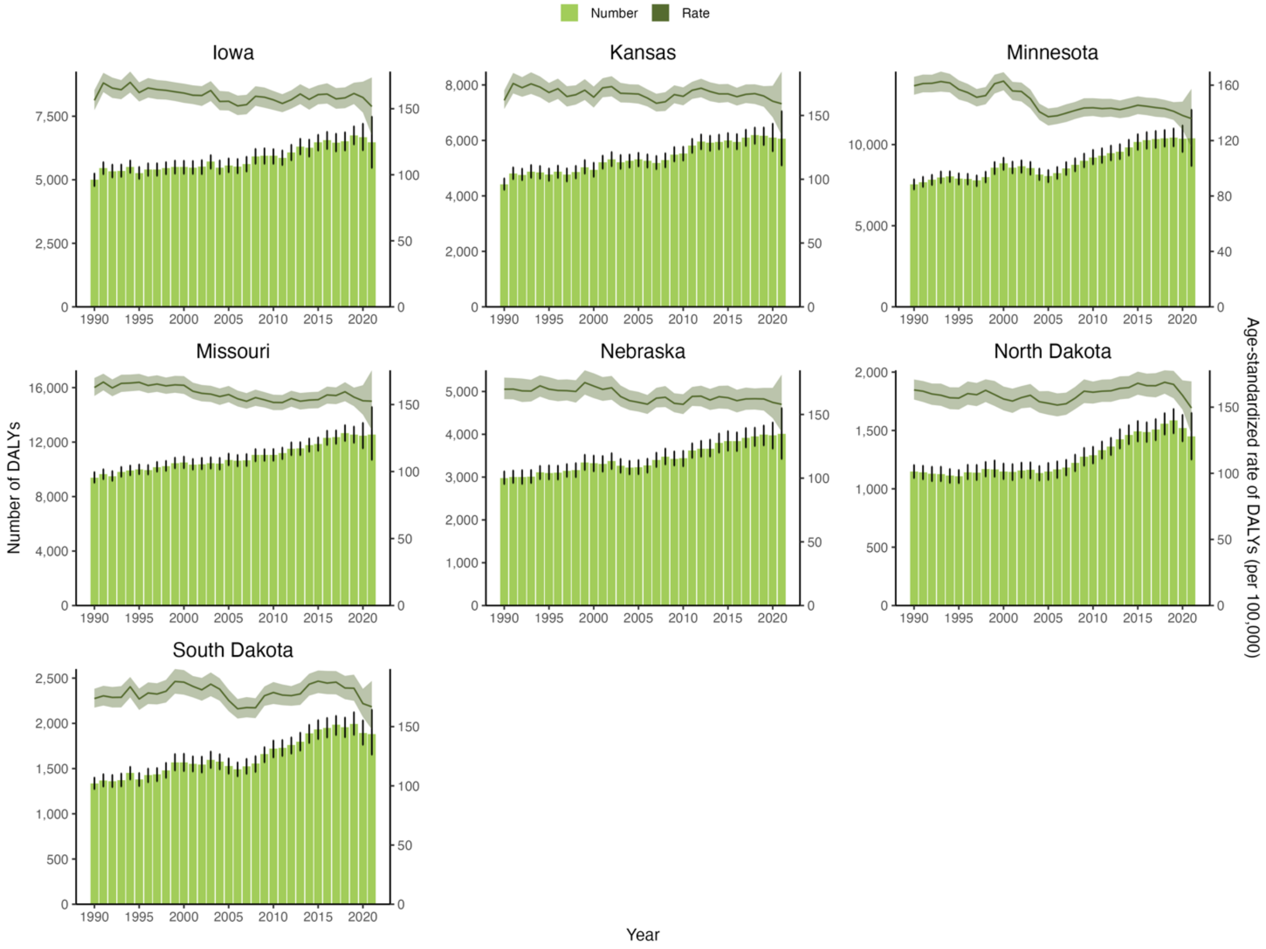

© 2025 GBD 2021 US CNS Cancer Collaborators. *JAMA Neurology*.

(E) South Atlantic

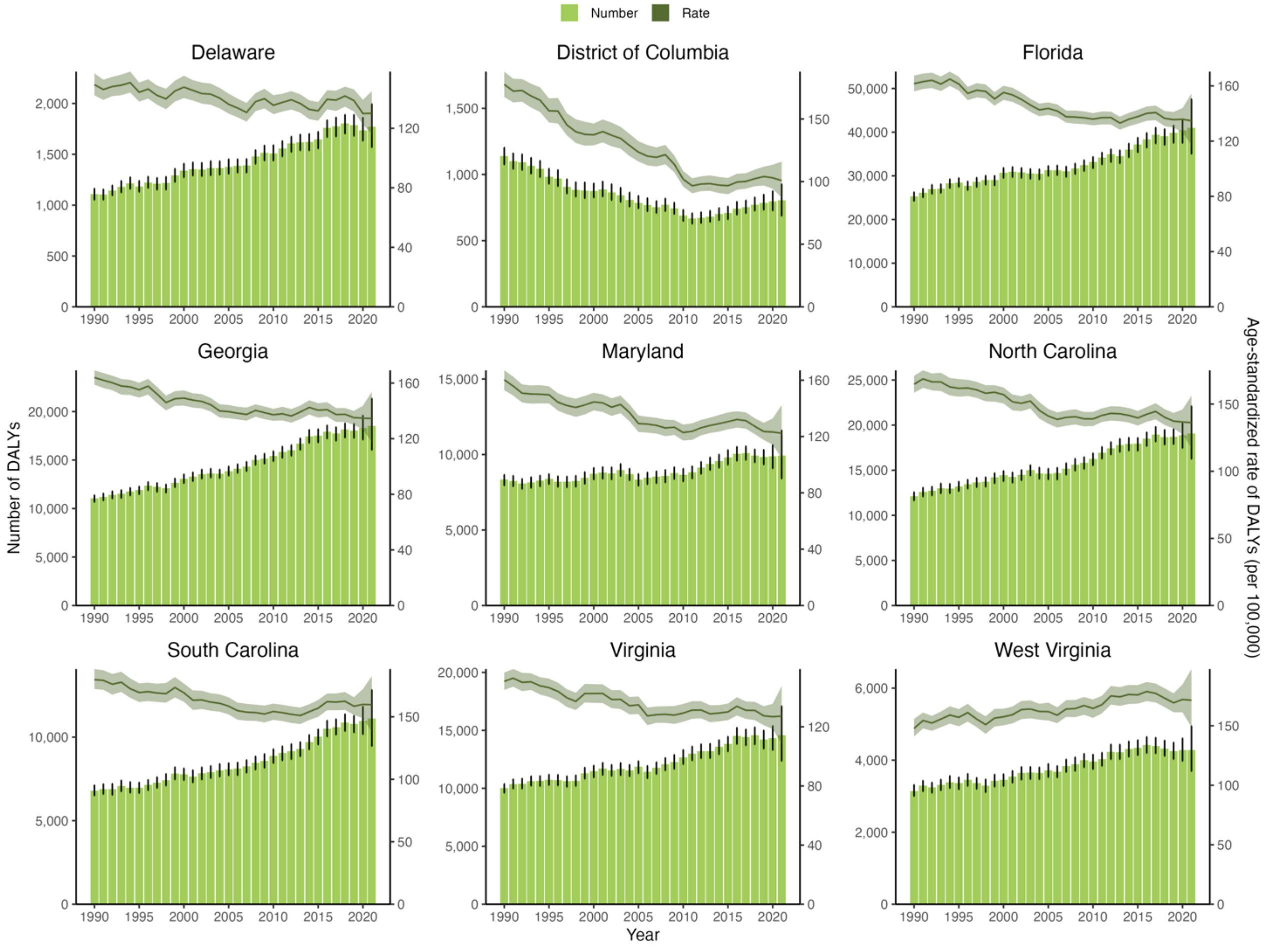

© 2025 GBD 2021 US CNS Cancer Collaborators. *JAMA Neurology*.

(F) East South Central

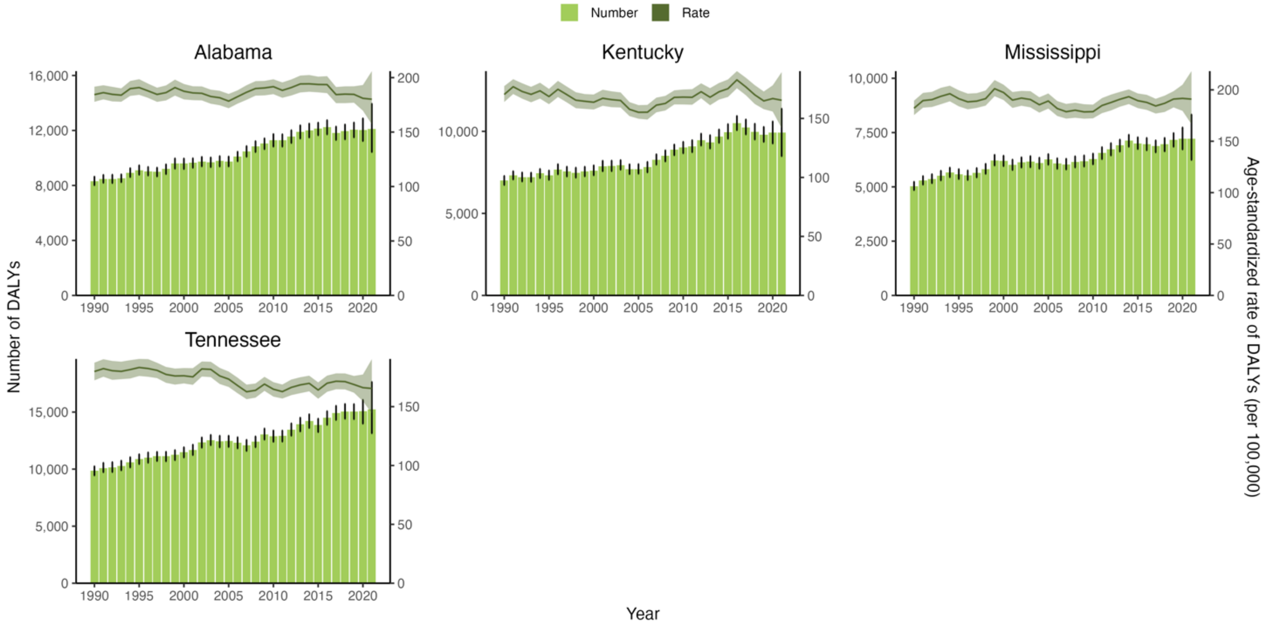

(G) West South Central

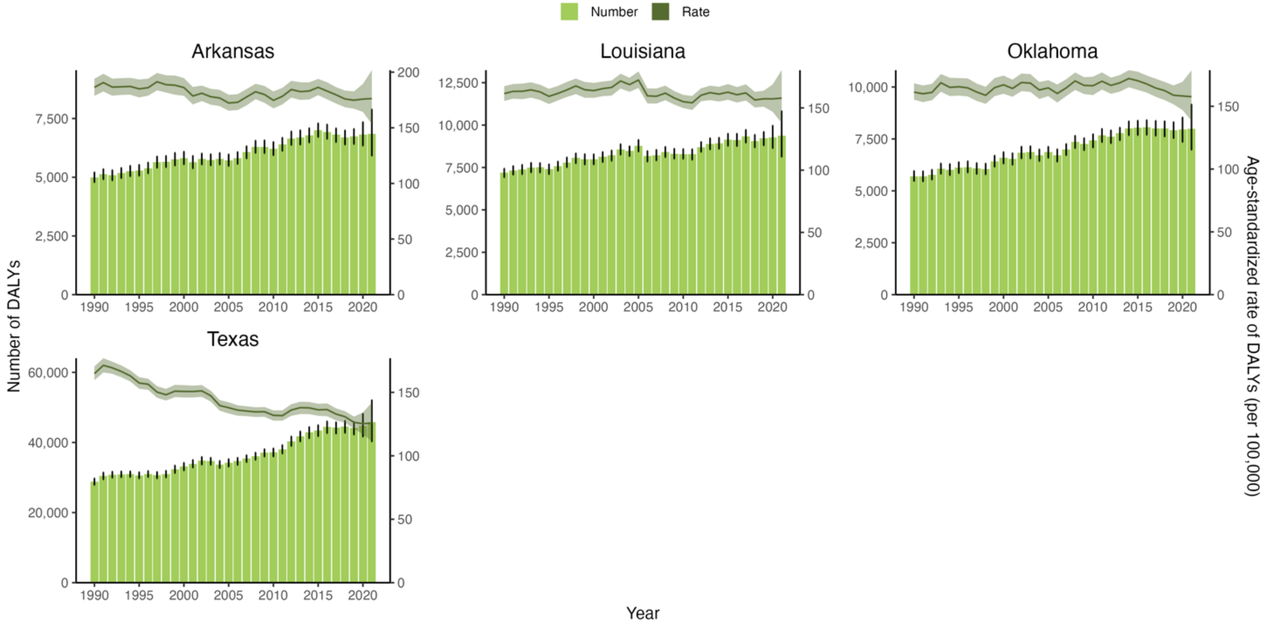

© 2025 GBD 2021 US CNS Cancer Collaborators. *JAMA Neurology*.

(H) Mountain

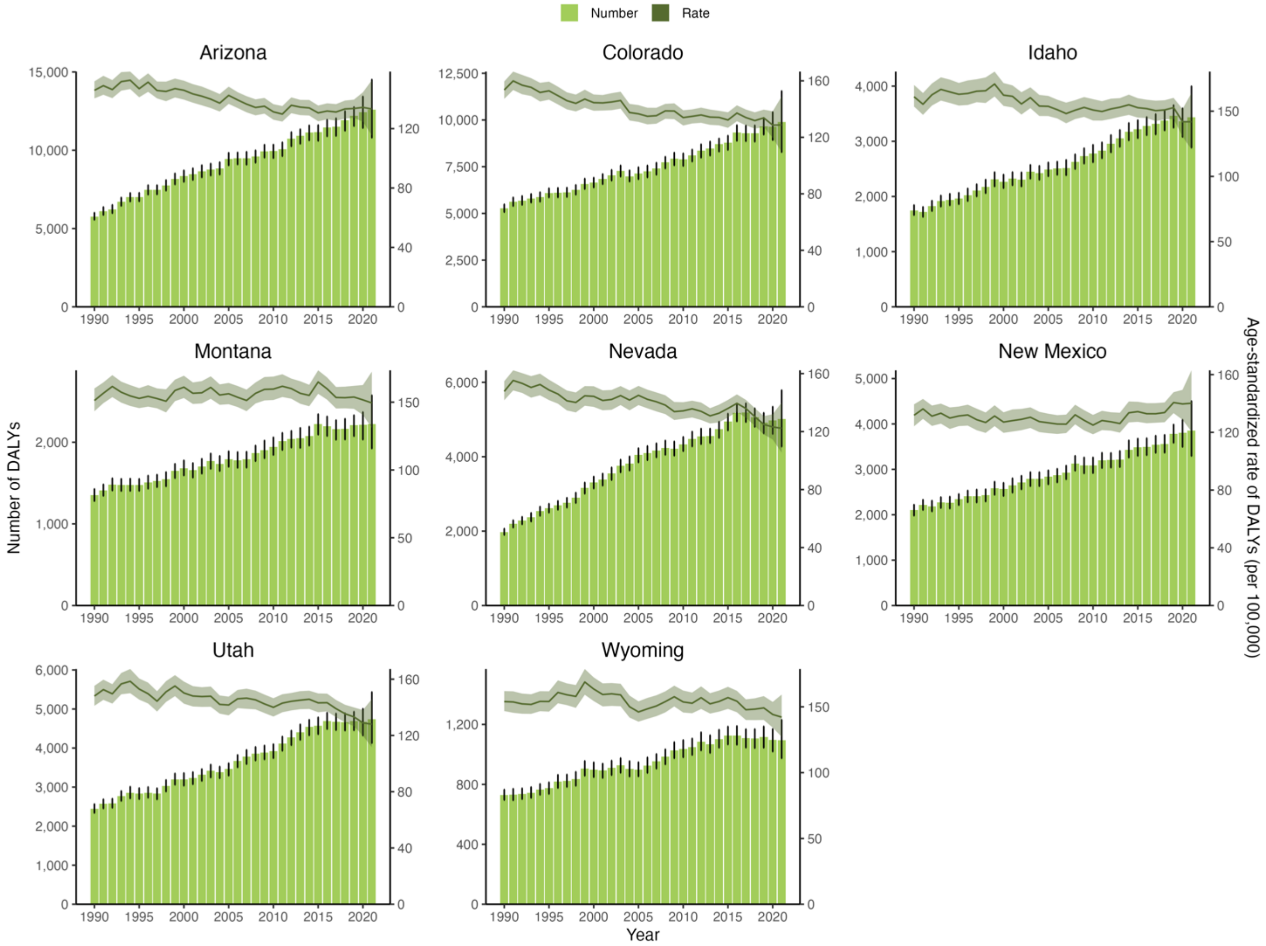

© 2025 GBD 2021 US CNS Cancer Collaborators. *JAMA Neurology*.

(I) Pacific

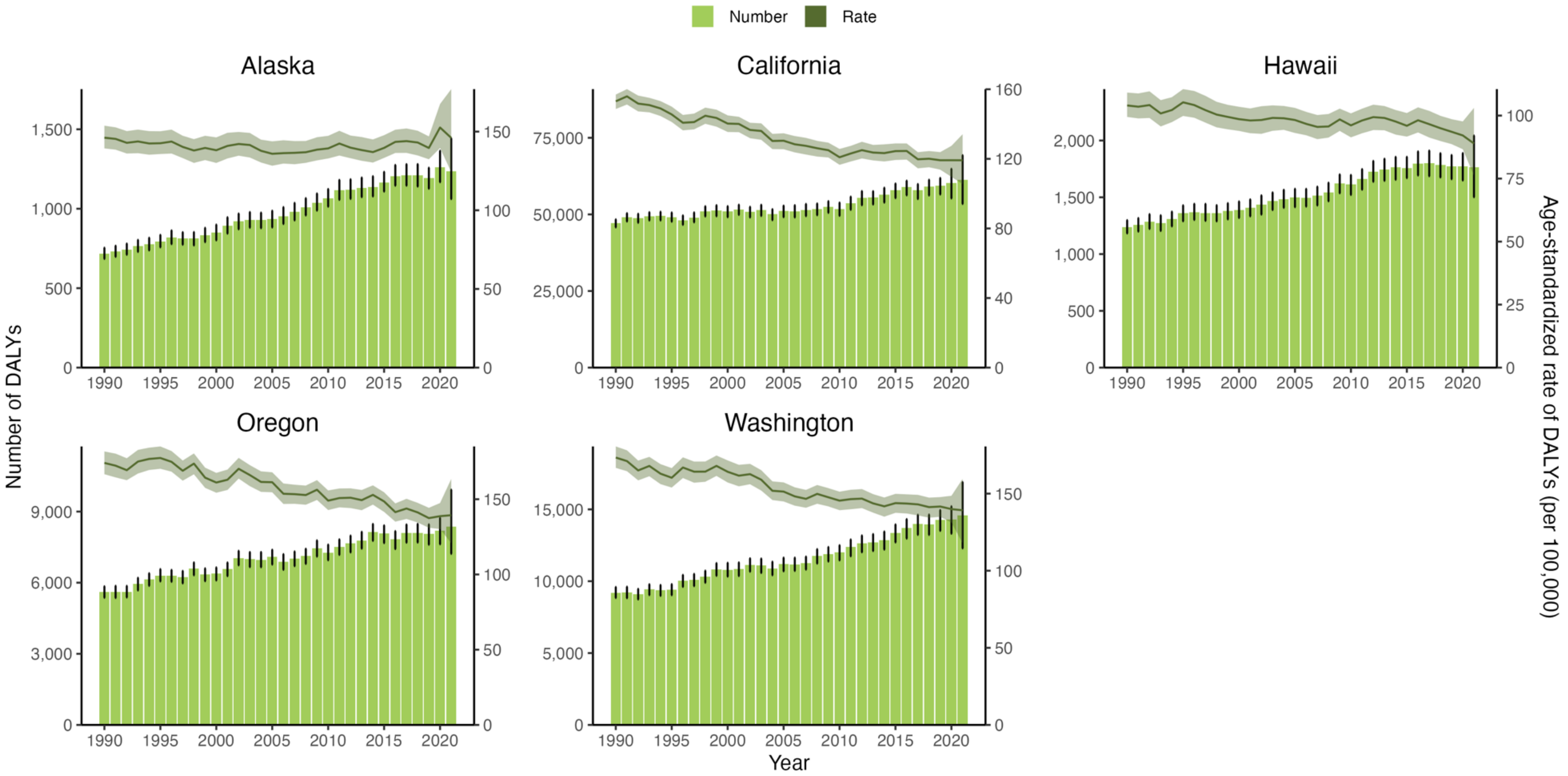

eFigure 1c: DALYs count and age-standardized rate of Brain and central nervous system cancer in the United States by division, (A) New England, (B) Middle Atlantic, (C) East North Central, (D) West North Central, (E) South Atlantic, (F) East South Central, (G) West South Central, (H) Mountain, (I) Pacific division, 1990 to 2021

© 2025 GBD 2021 US CNS Cancer Collaborators. *JAMA Neurology*.

(A) New England

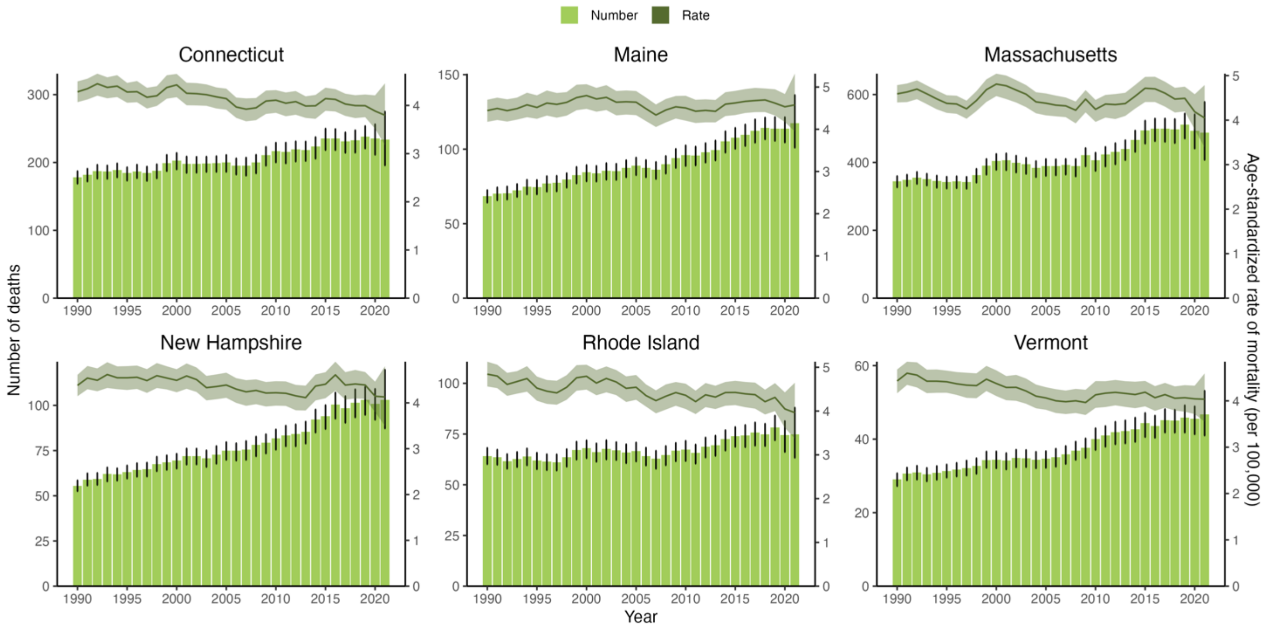

(B) Middle Atlantic

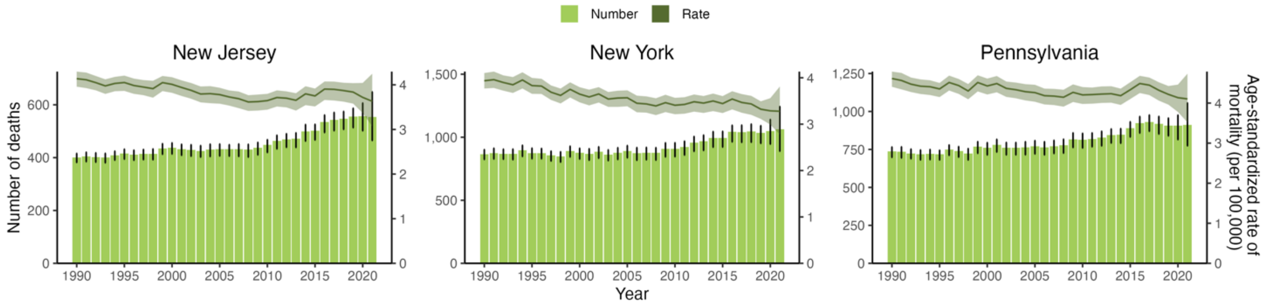

© 2025 GBD 2021 US CNS Cancer Collaborators. *JAMA Neurology*.

(C) East North Central

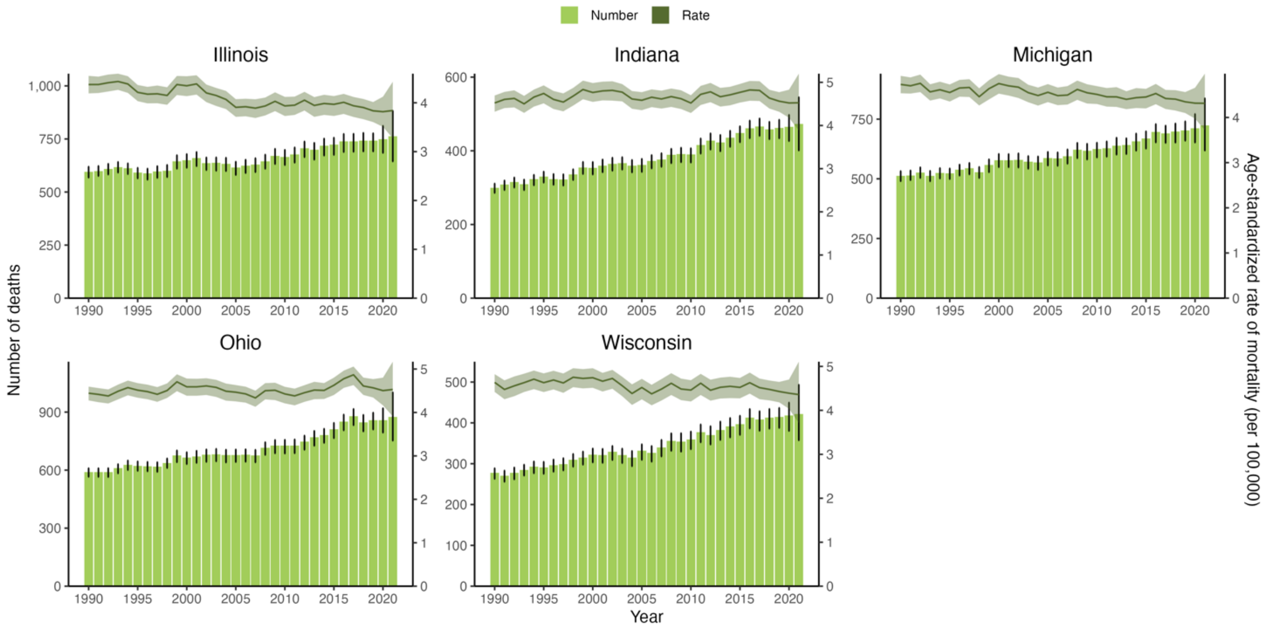

© 2025 GBD 2021 US CNS Cancer Collaborators. *JAMA Neurology*.

(D) West North Central

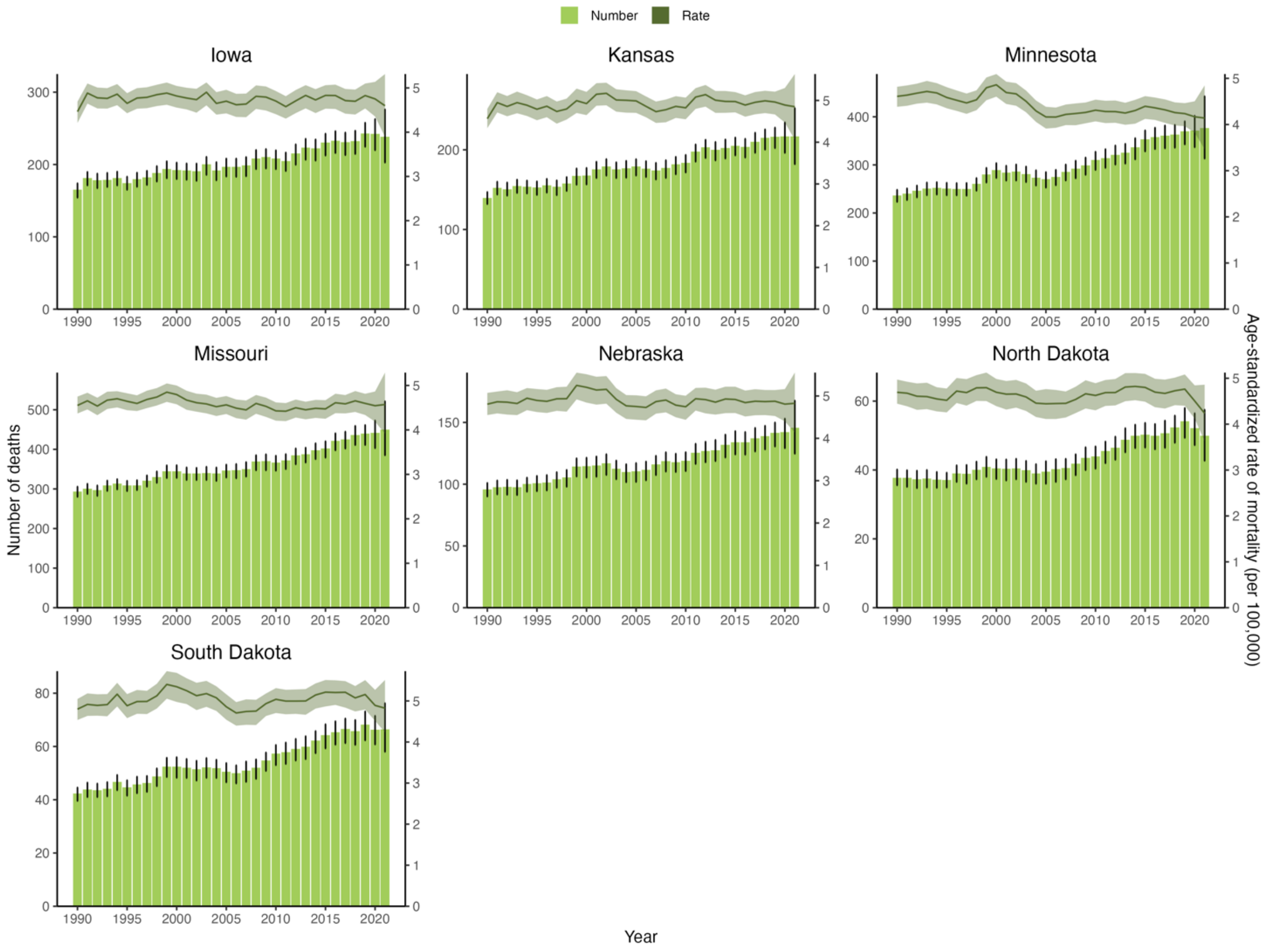

© 2025 GBD 2021 US CNS Cancer Collaborators. *JAMA Neurology*.

(E) South Atlantic

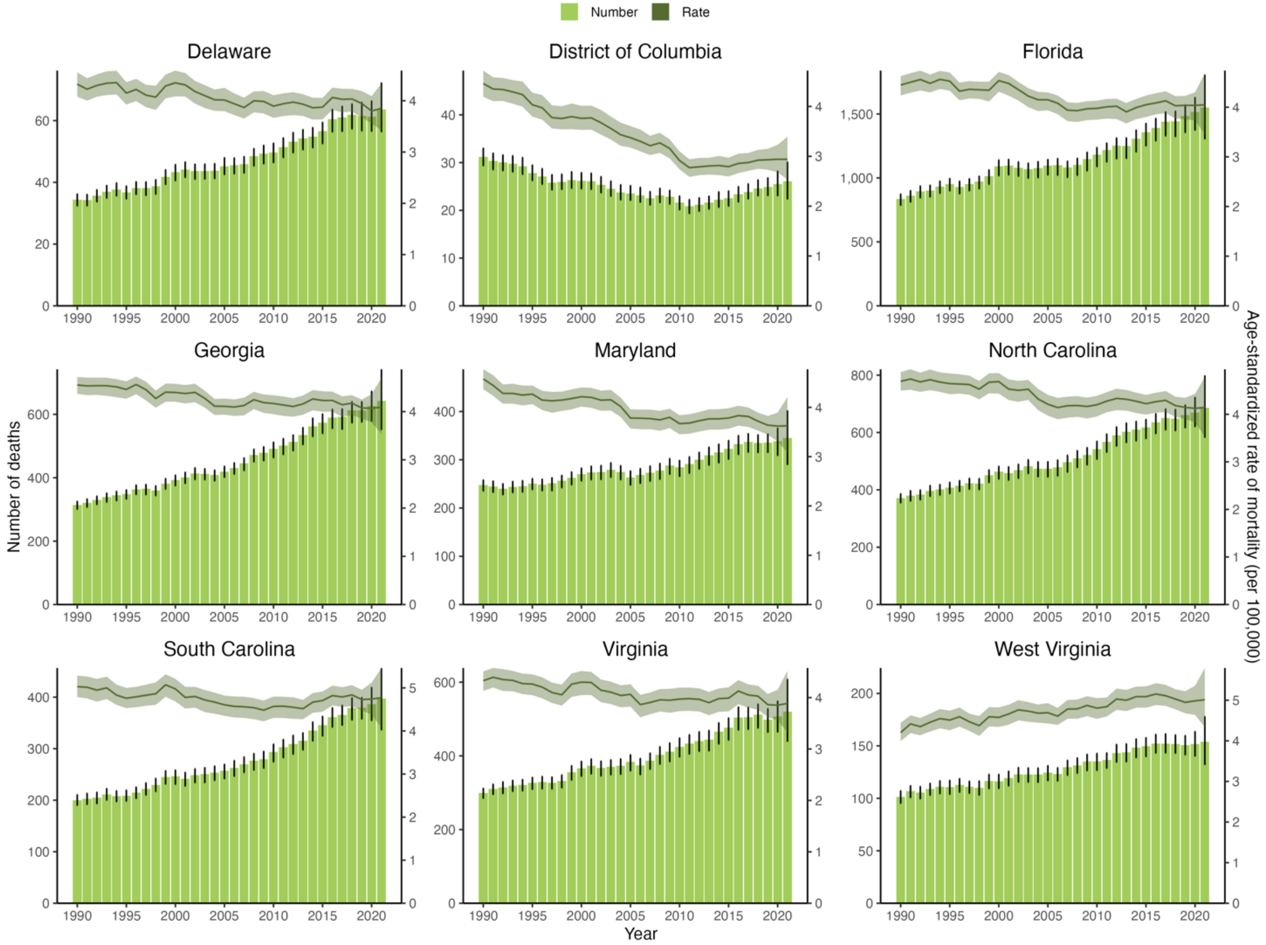

© 2025 GBD 2021 US CNS Cancer Collaborators. *JAMA Neurology*.

(F) East South Central

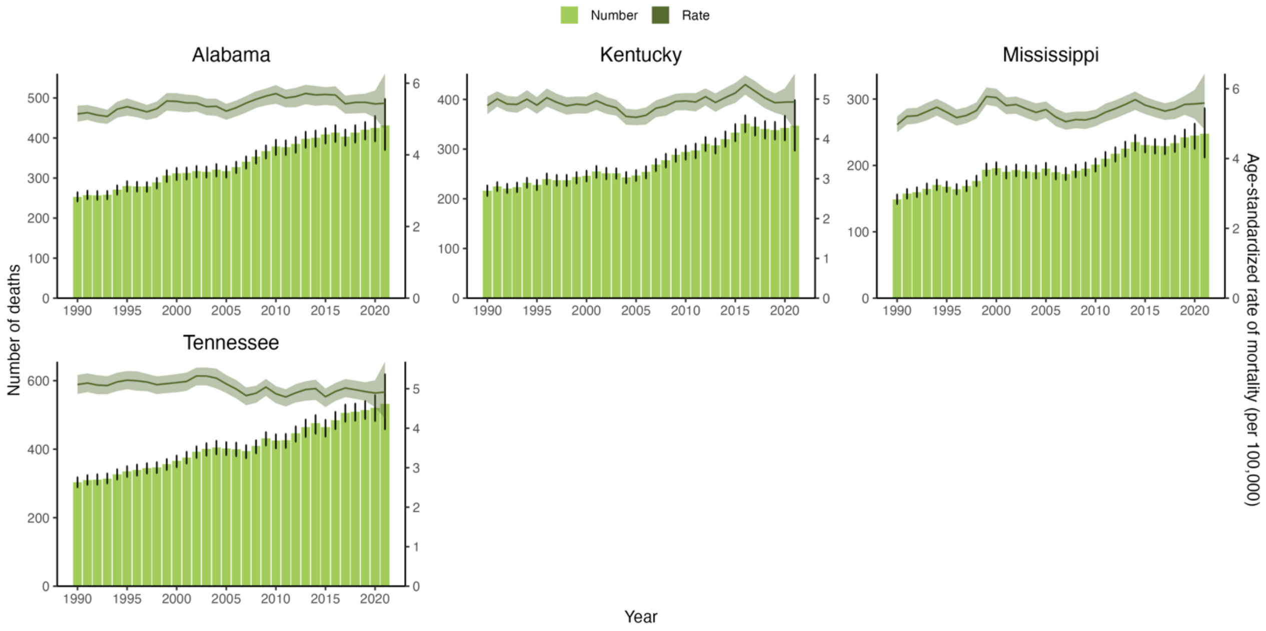

(G) West South Central

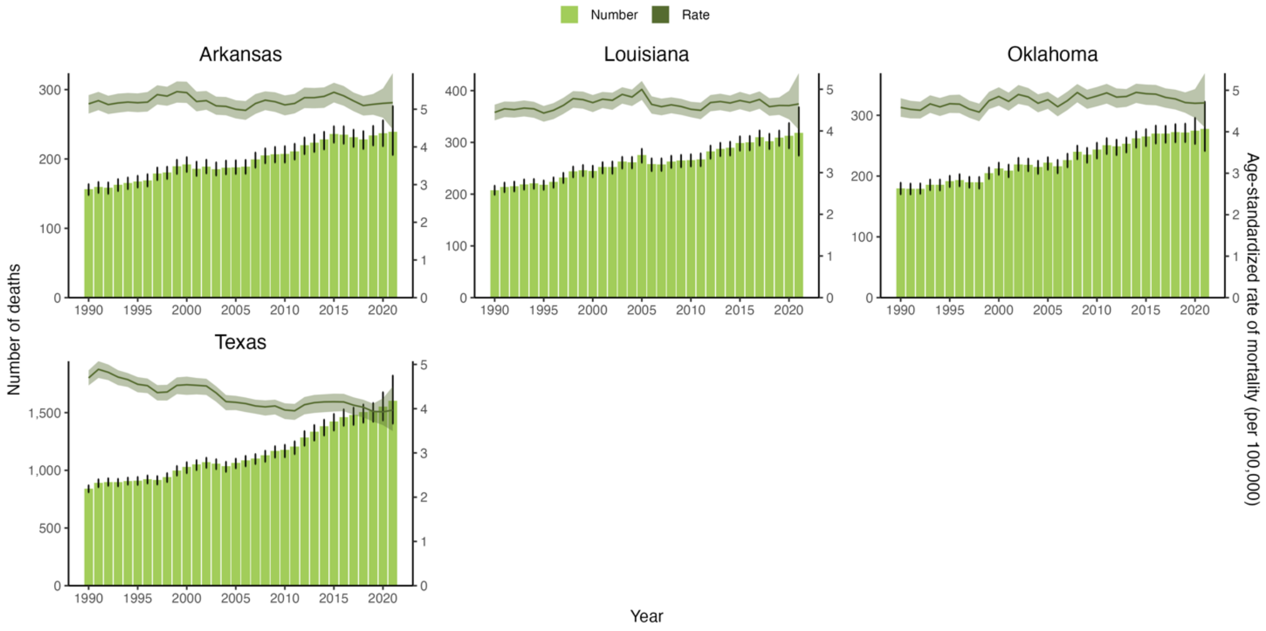

© 2025 GBD 2021 US CNS Cancer Collaborators. *JAMA Neurology*.

(H) Mountain

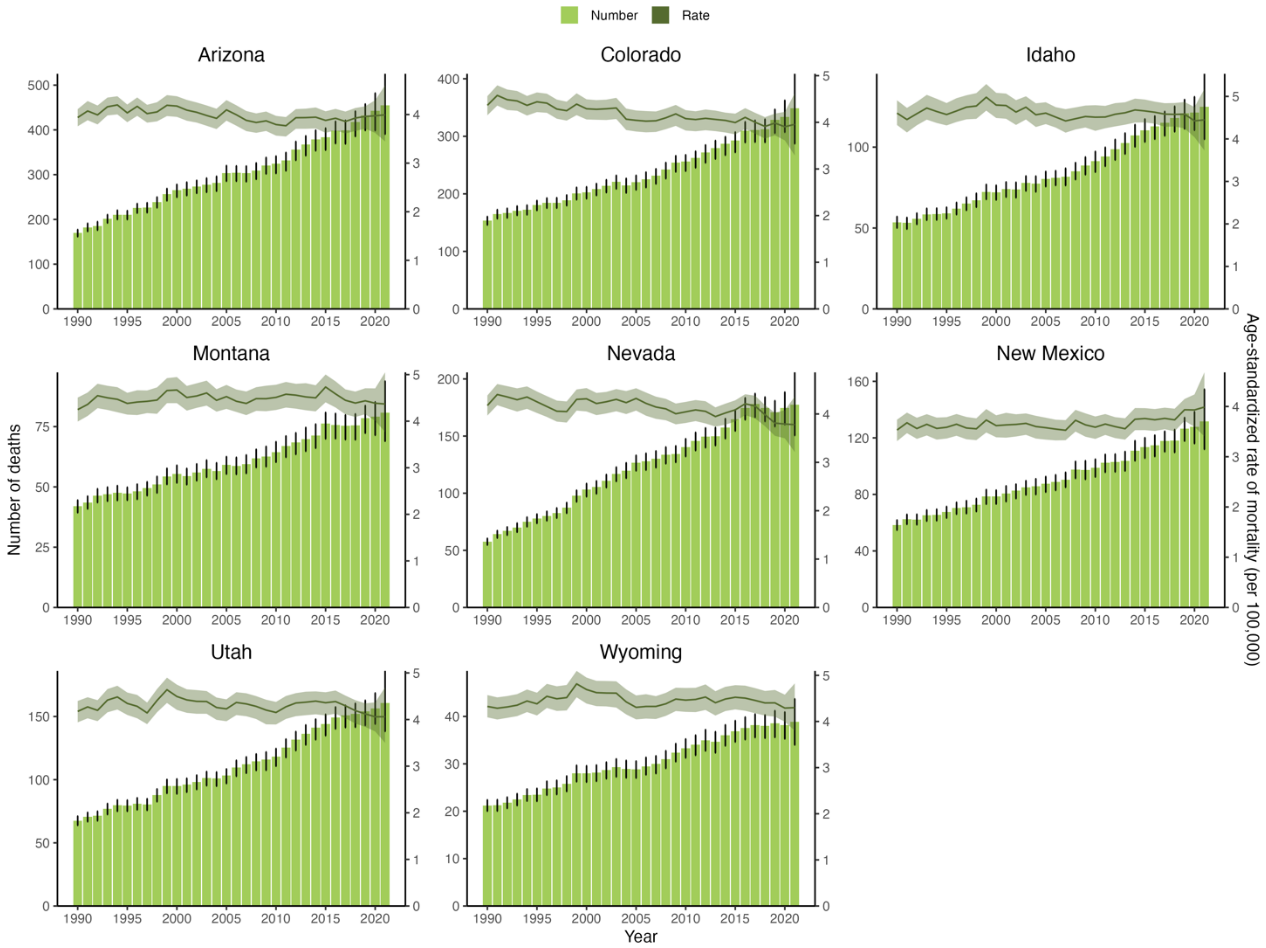

© 2025 GBD 2021 US CNS Cancer Collaborators. *JAMA Neurology*.

(I) Pacific

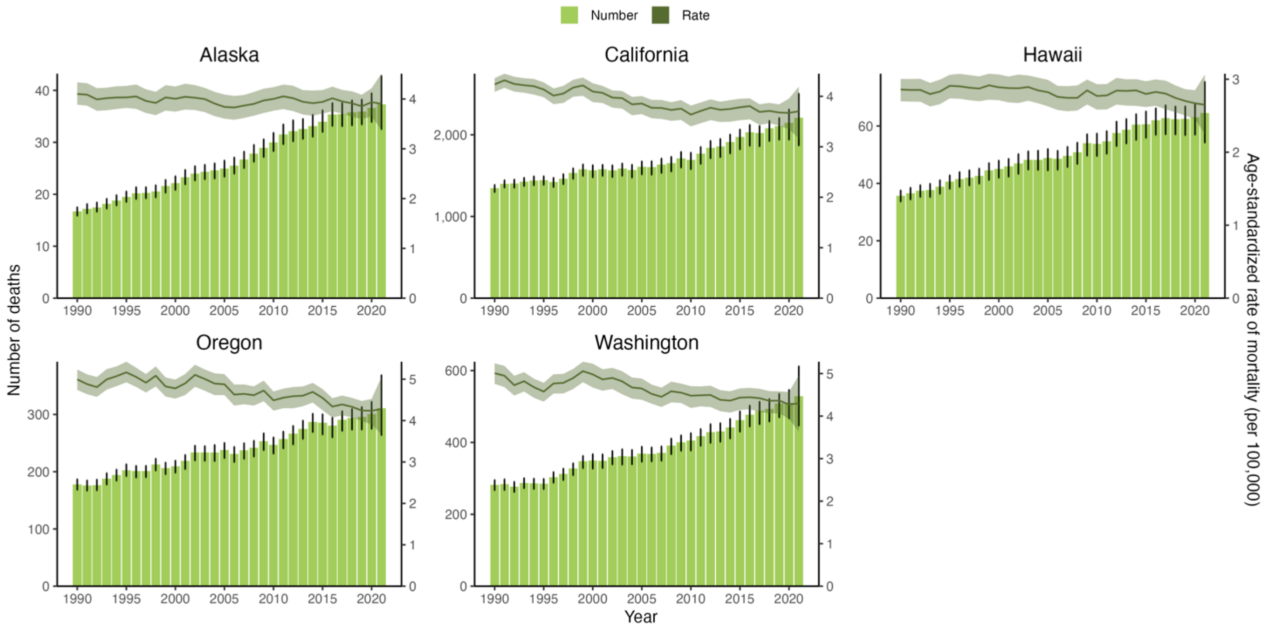

eFigure 1d: Death count and age-standardized rate of Brain and central nervous system cancer in the United States by division, (A) New England, (B) Middle Atlantic, (C) East North Central, (D) West North Central, (E) South Atlantic, (F) East South Central, (G) West South Central, (H) Mountain, (I) Pacific division, 1990 to 2021

© 2025 GBD 2021 US CNS Cancer Collaborators. *JAMA Neurology*.

(A) New England

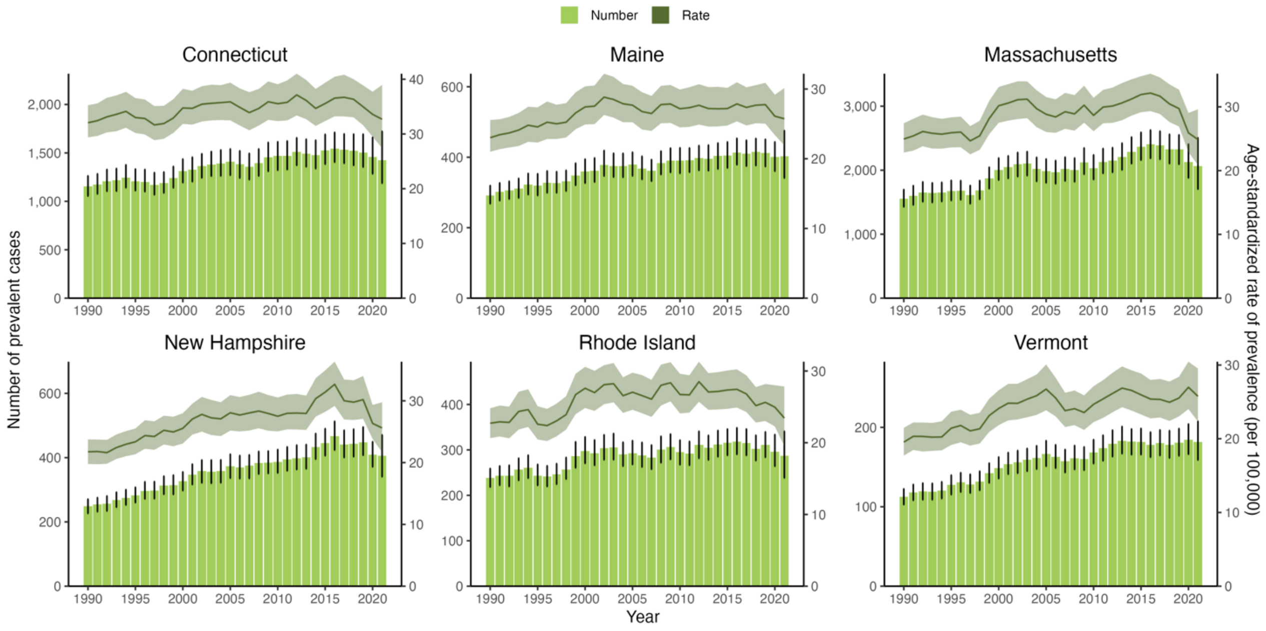

(B) Middle Atlantic

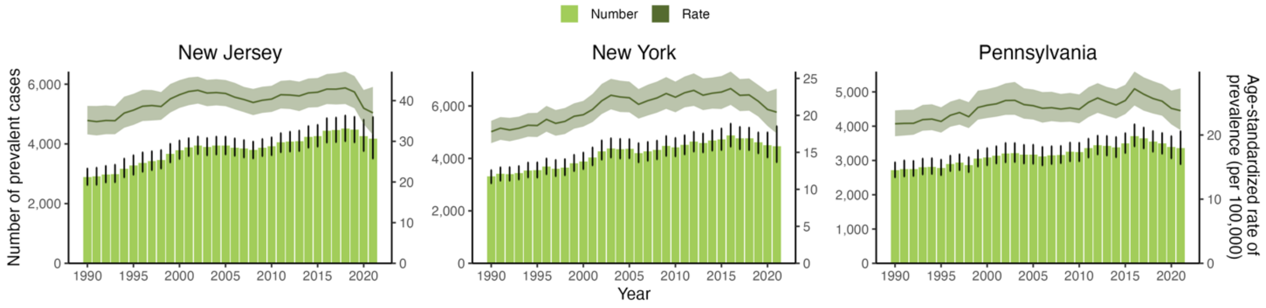

© 2025 GBD 2021 US CNS Cancer Collaborators. *JAMA Neurology*.

(C) East North Central

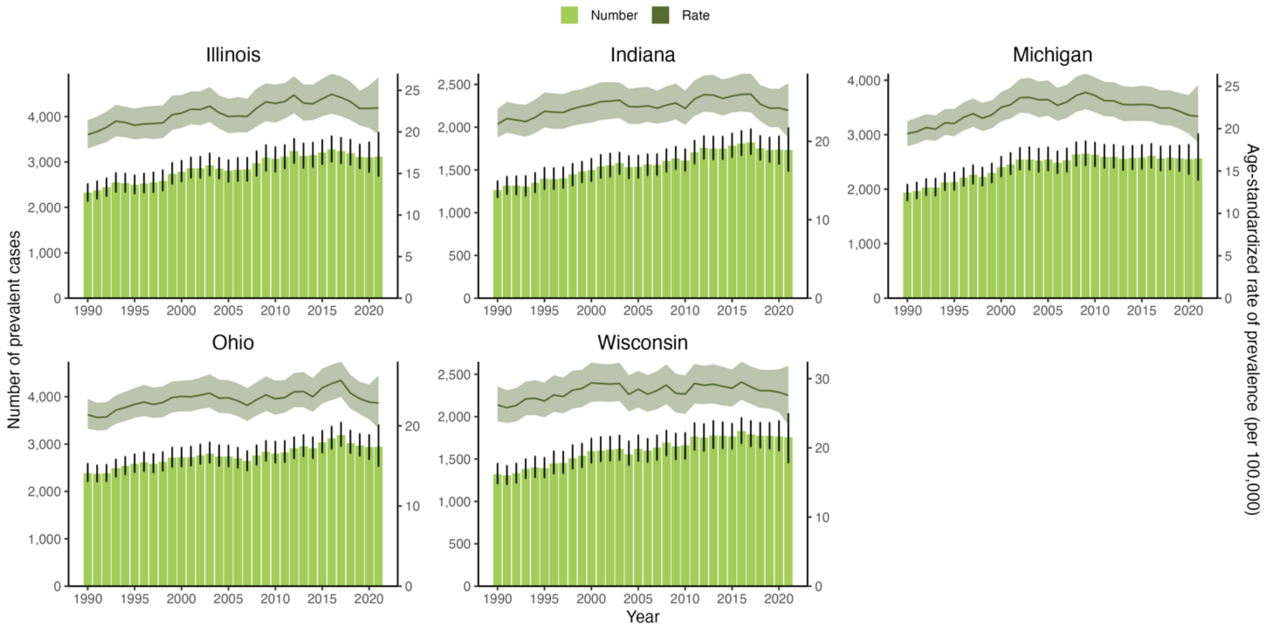

© 2025 GBD 2021 US CNS Cancer Collaborators. *JAMA Neurology*.

(D) West North Central

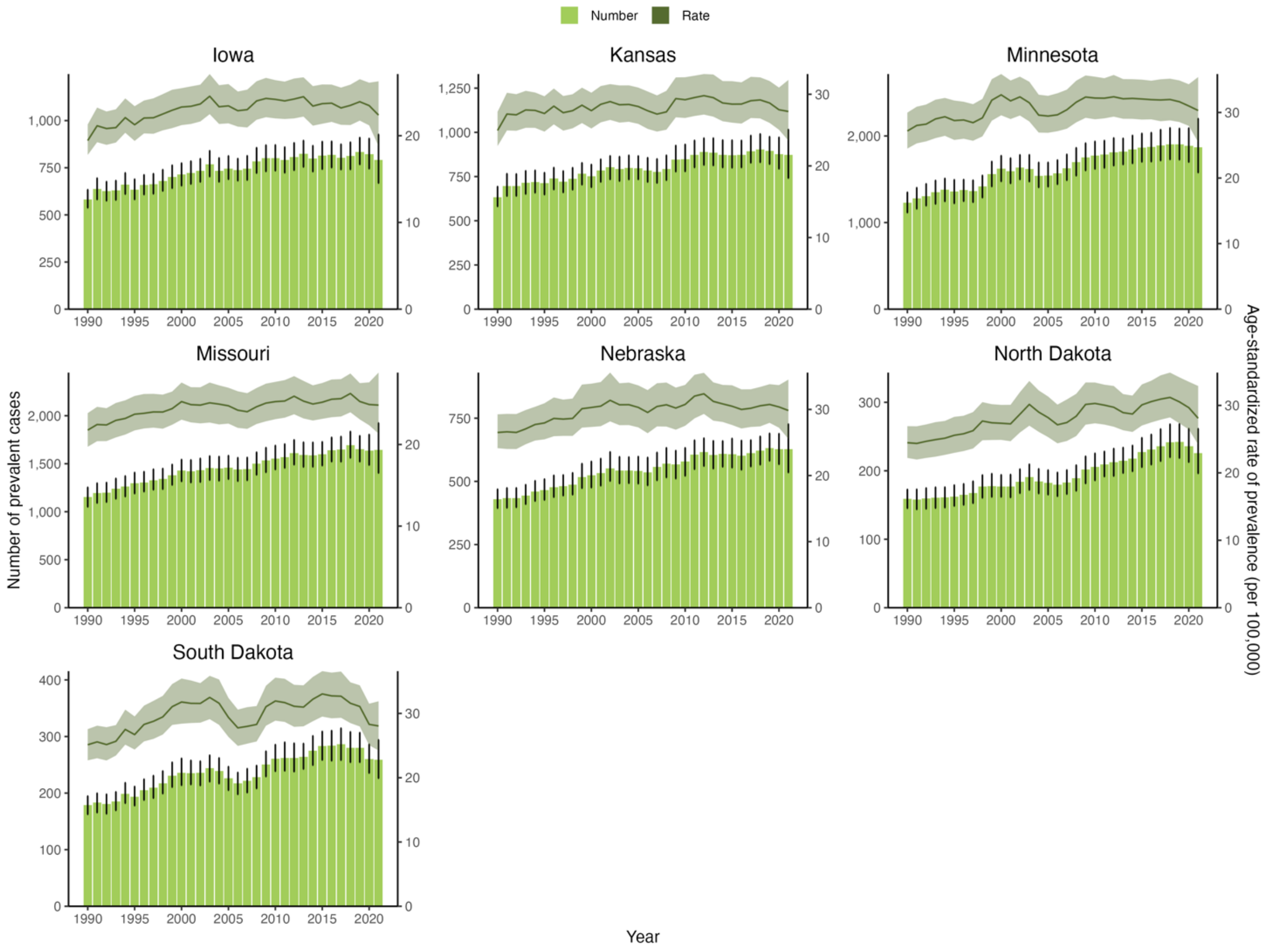

© 2025 GBD 2021 US CNS Cancer Collaborators. *JAMA Neurology*.

(E) South Atlantic

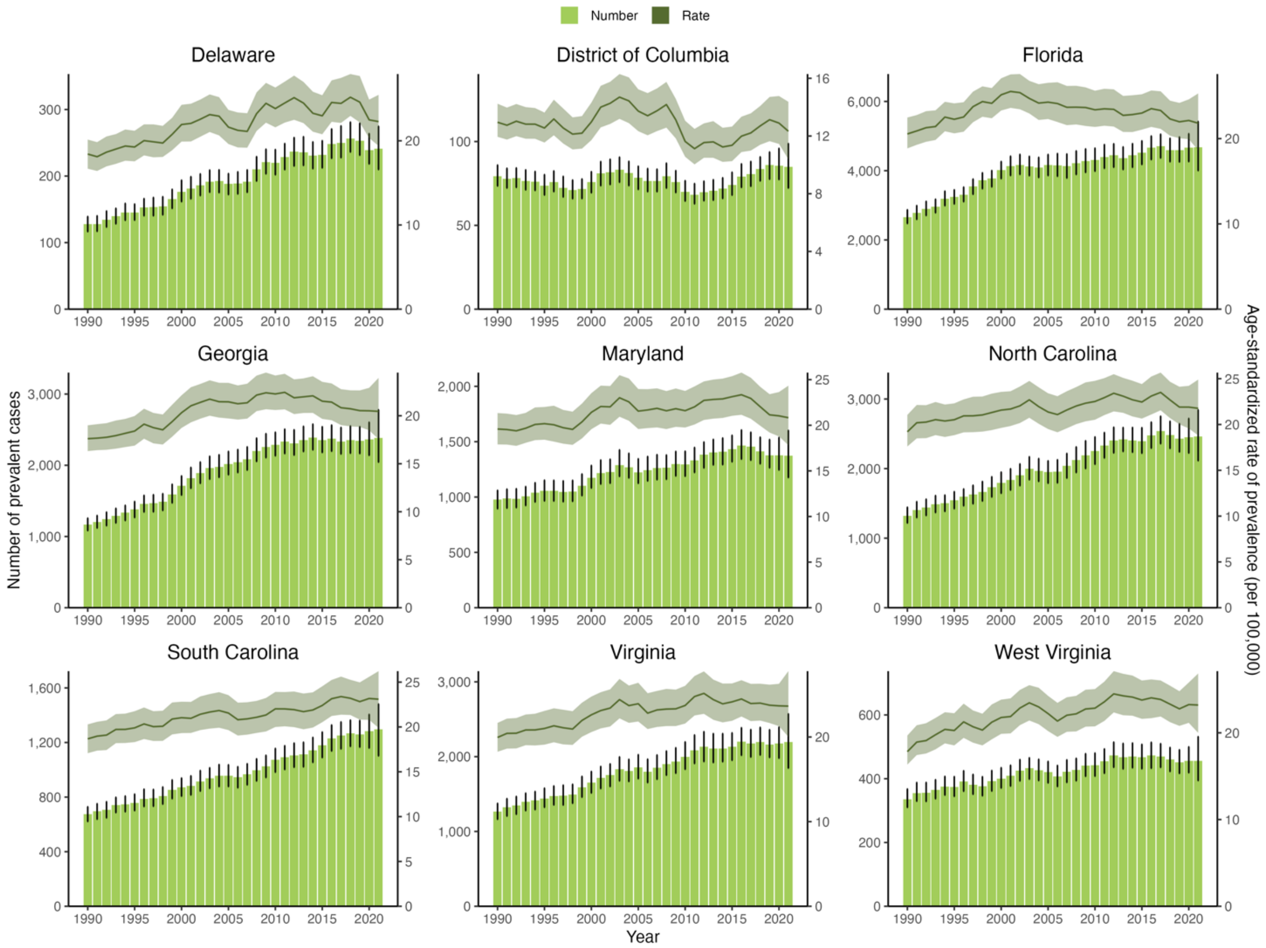

© 2025 GBD 2021 US CNS Cancer Collaborators. *JAMA Neurology*.

(F) East South Central

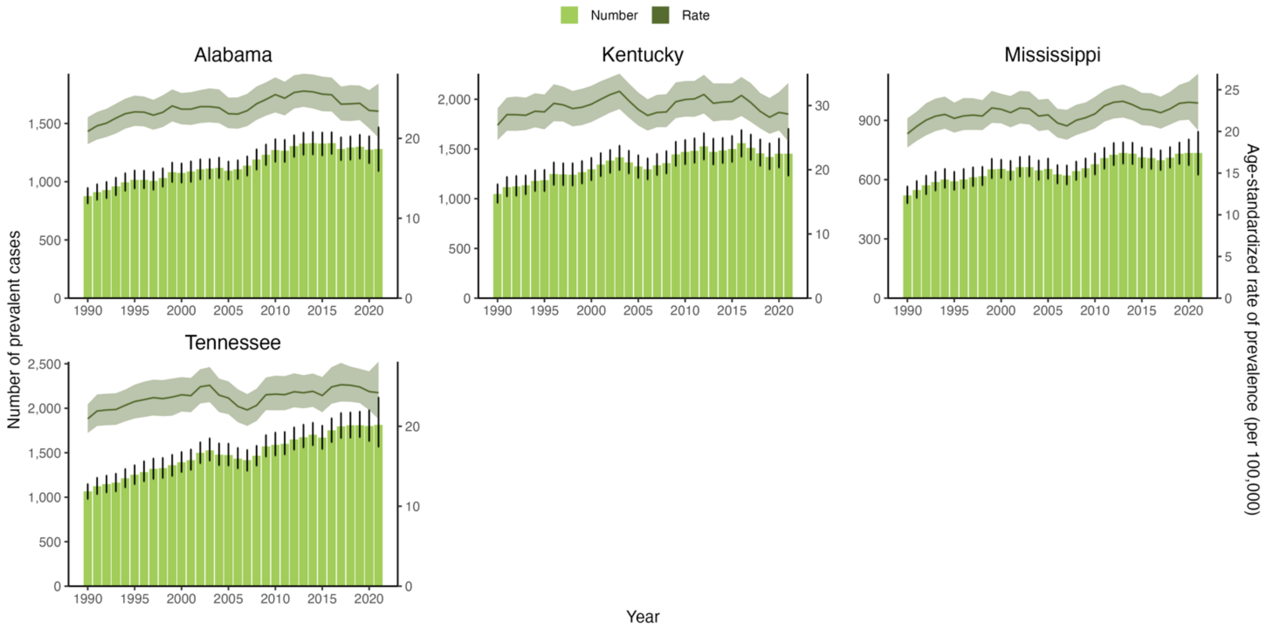

(G) West South Central

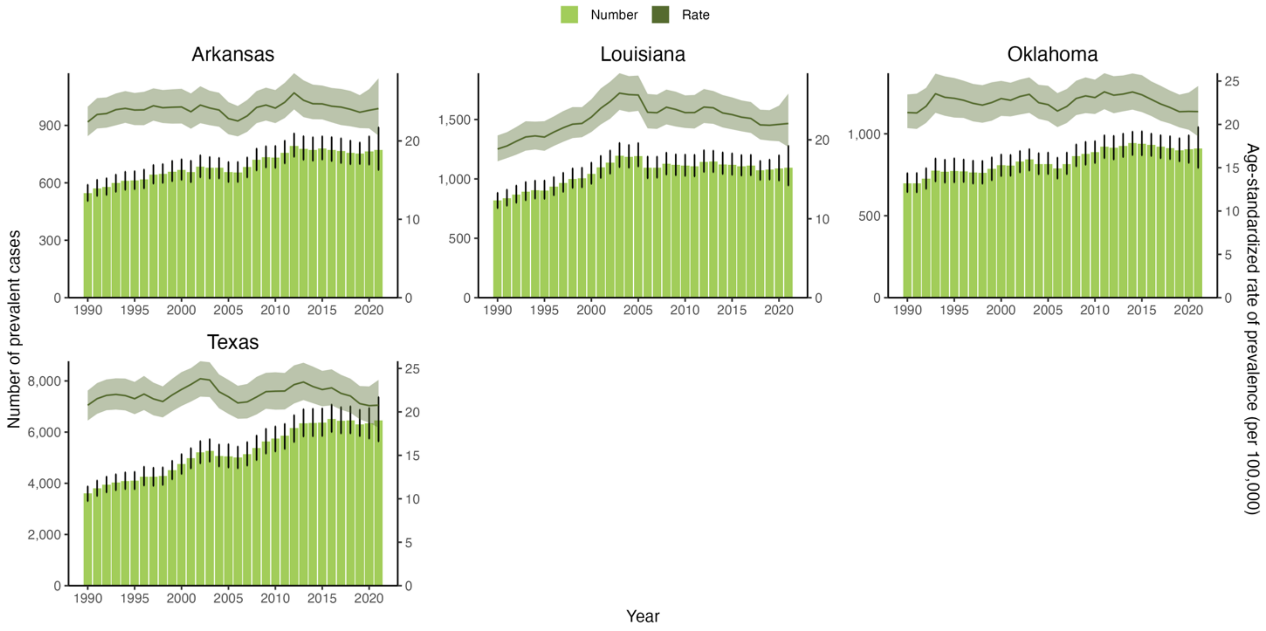

© 2025 GBD 2021 US CNS Cancer Collaborators. *JAMA Neurology*.

(H) Mountain

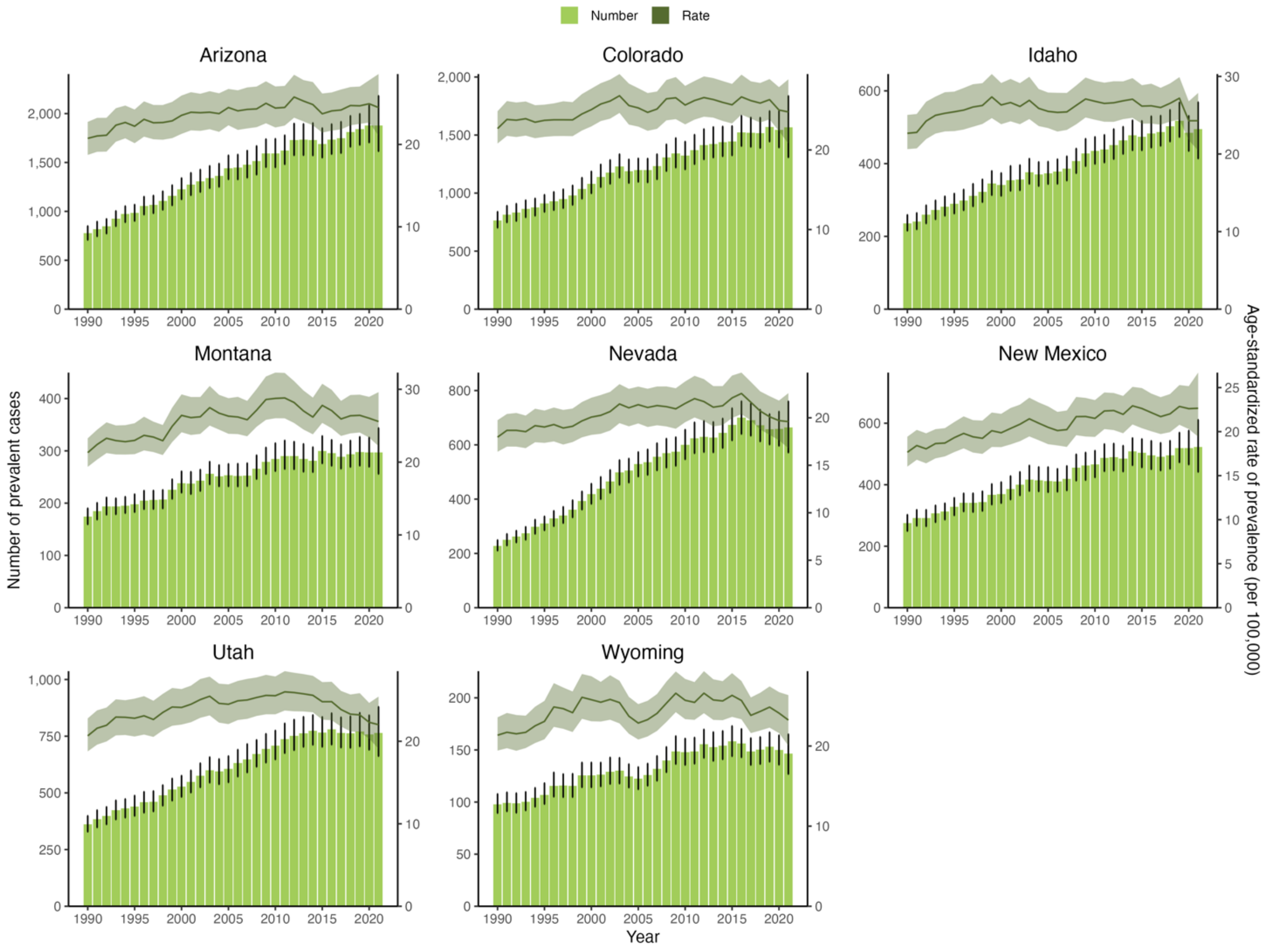

© 2025 GBD 2021 US CNS Cancer Collaborators. *JAMA Neurology*.

(I) Pacific

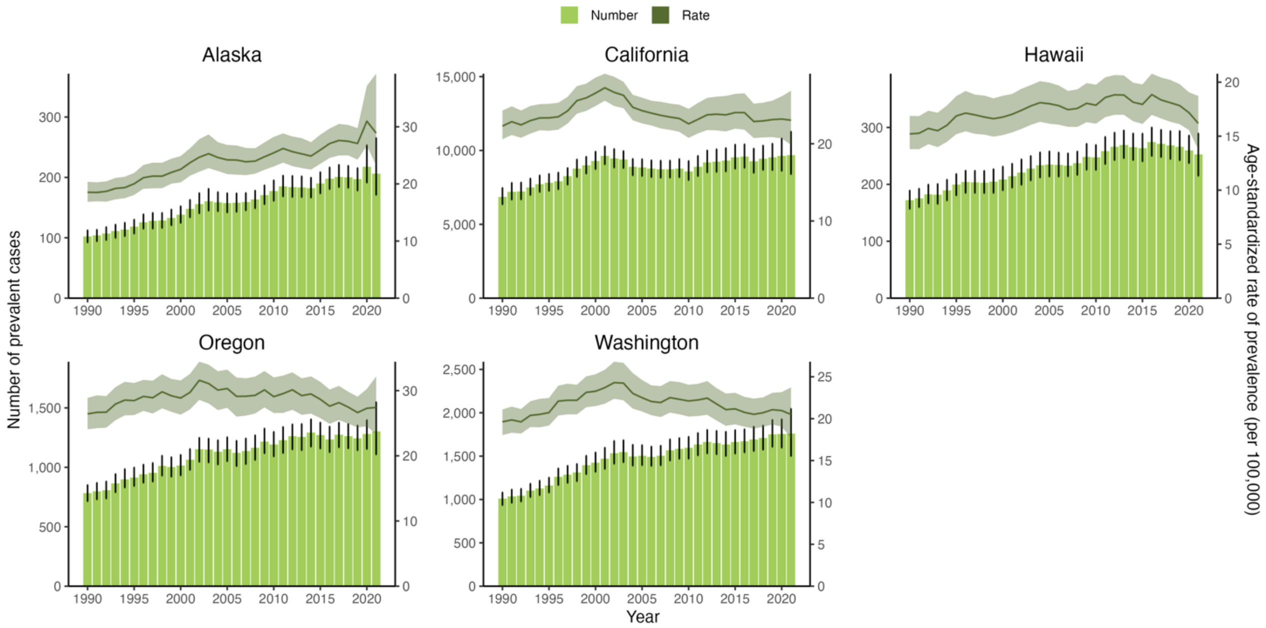

eFigure 1e: Prevalence count and age-standardized rate of Brain and central nervous system cancer in the United States by division, (A) New England, (B) Middle Atlantic, (C) East North Central, (D) West North Central, (E) South Atlantic, (F) East South Central, (G) West South Central, (H) Mountain, (I) Pacific division, 1990 to 2021

© 2025 GBD 2021 US CNS Cancer Collaborators. *JAMA Neurology*.

(A) New England

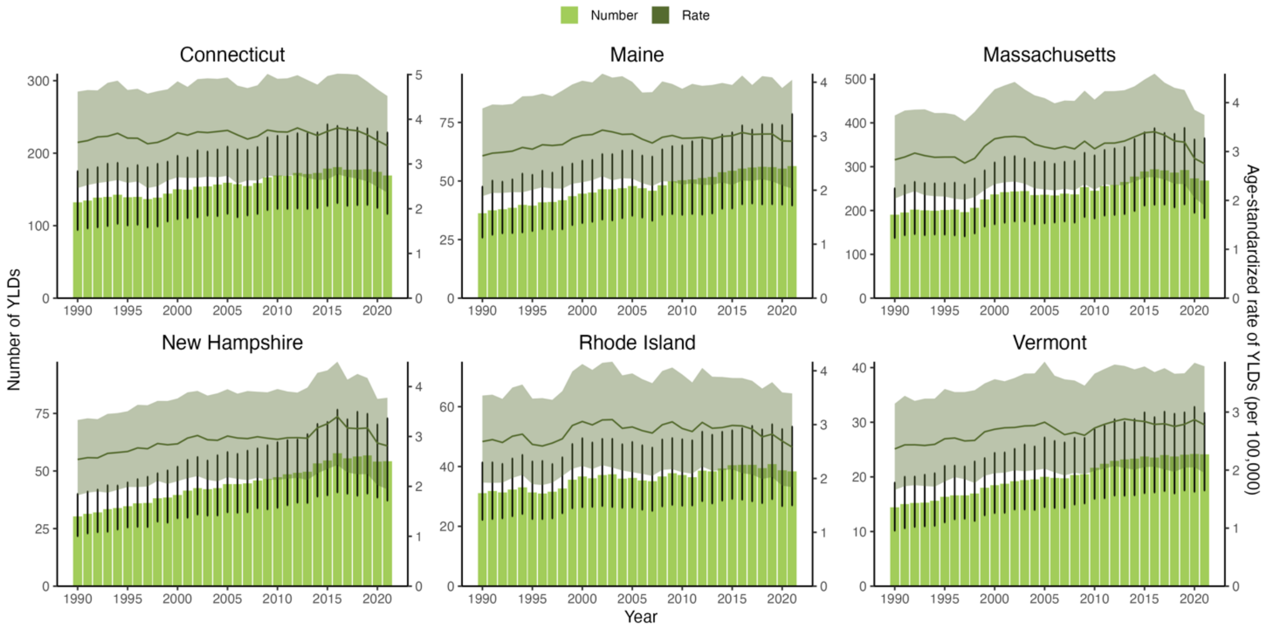

(B) Middle Atlantic

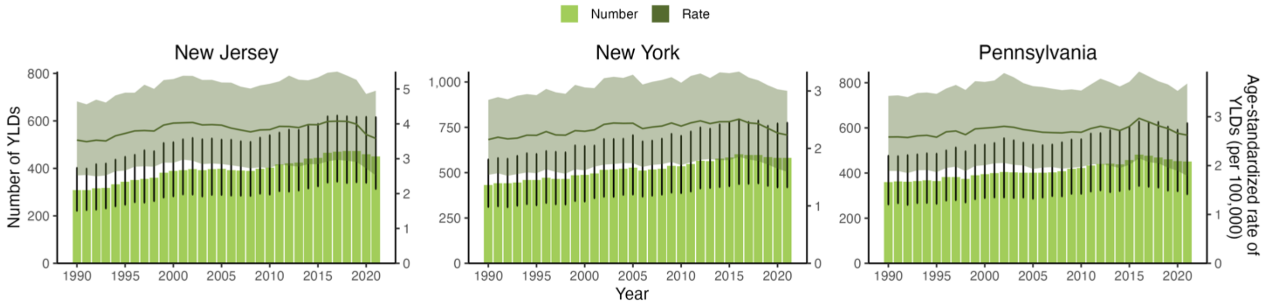

© 2025 GBD 2021 US CNS Cancer Collaborators. *JAMA Neurology*.

(C) East North Central

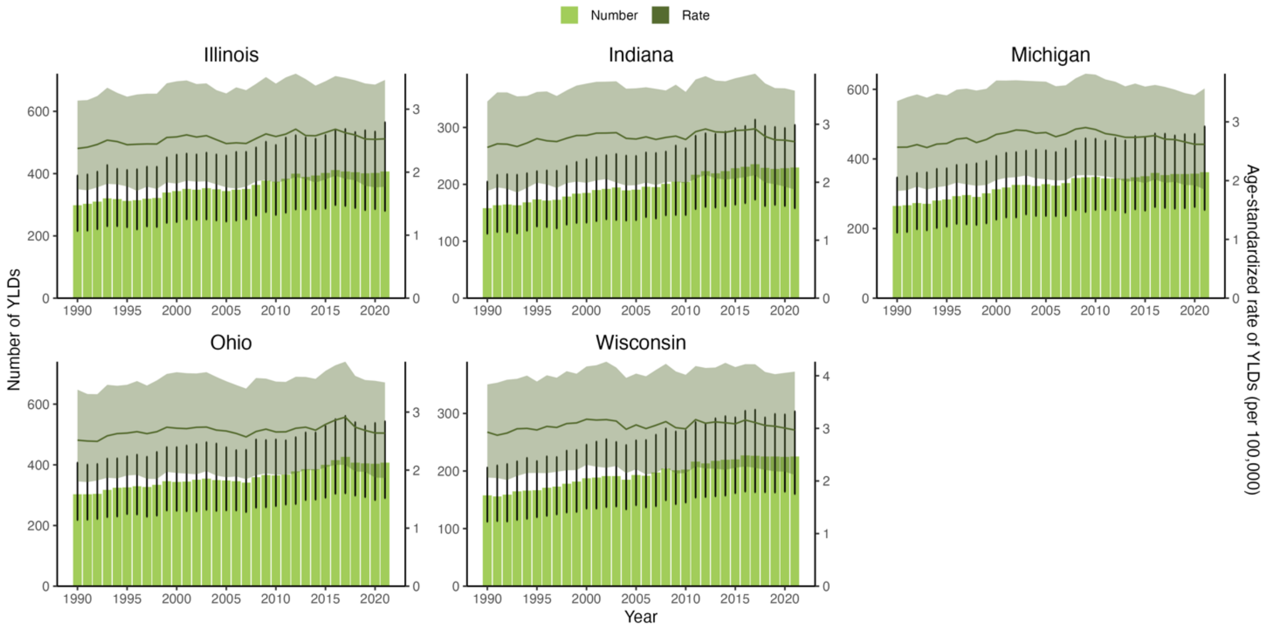

© 2025 GBD 2021 US CNS Cancer Collaborators. *JAMA Neurology*.

(D) West North Central

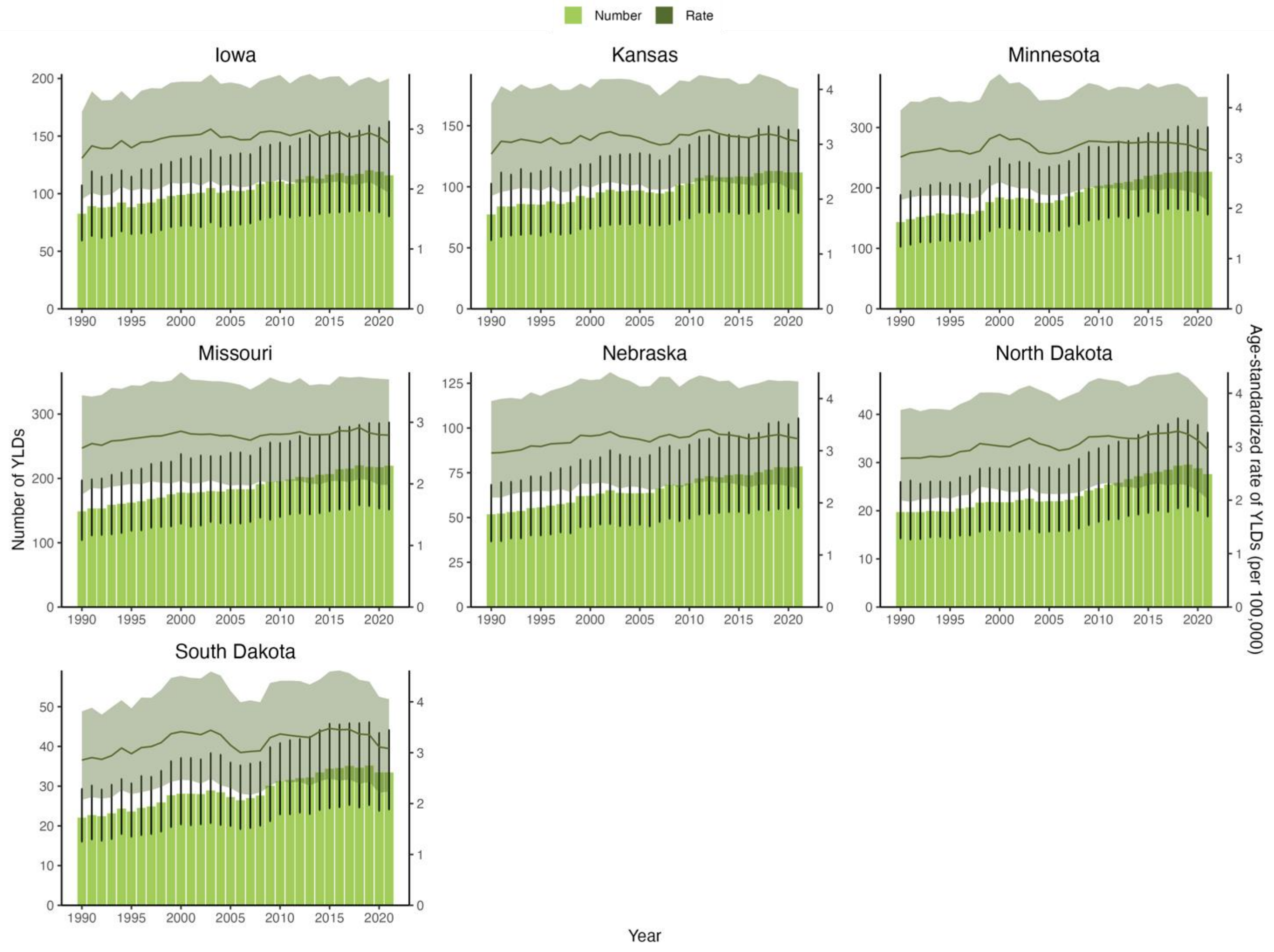

© 2025 GBD 2021 US CNS Cancer Collaborators. *JAMA Neurology*.

(E) South Atlantic

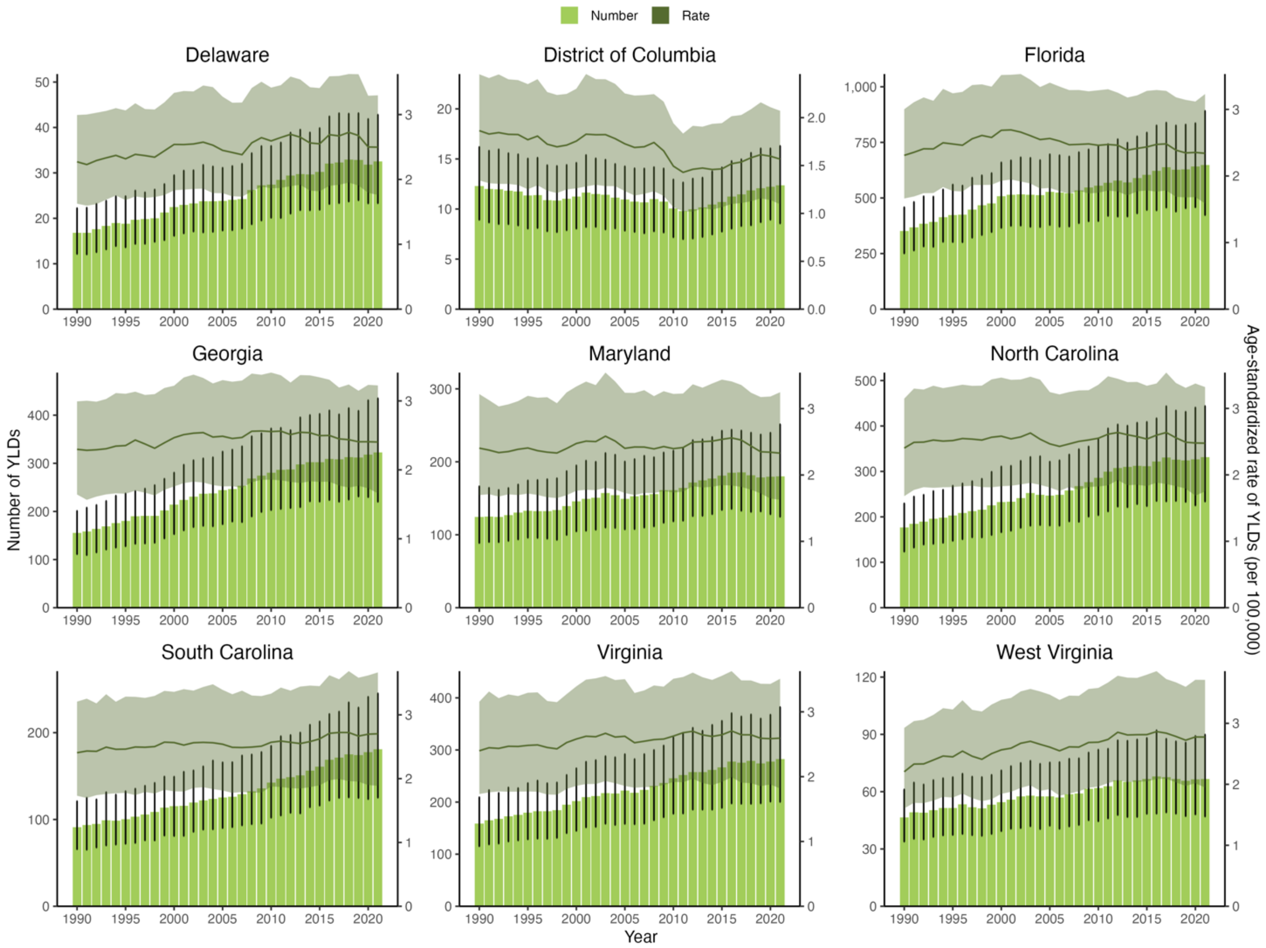

© 2025 GBD 2021 US CNS Cancer Collaborators. *JAMA Neurology*.

(F) East South Central

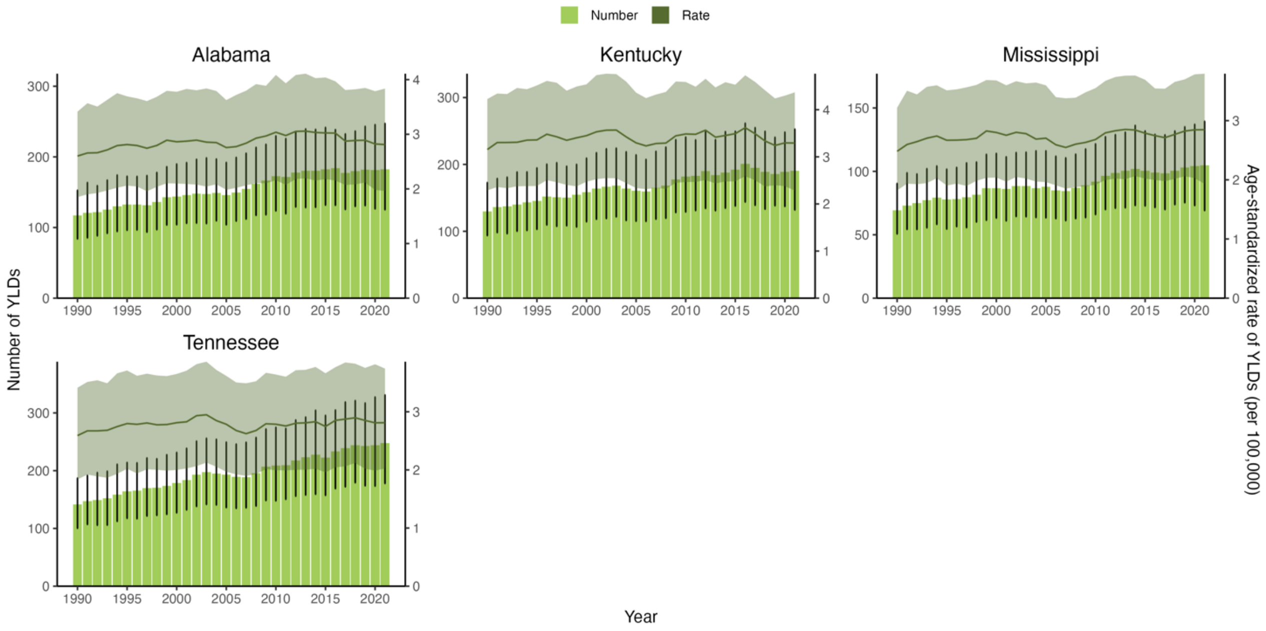

(G) West South Central

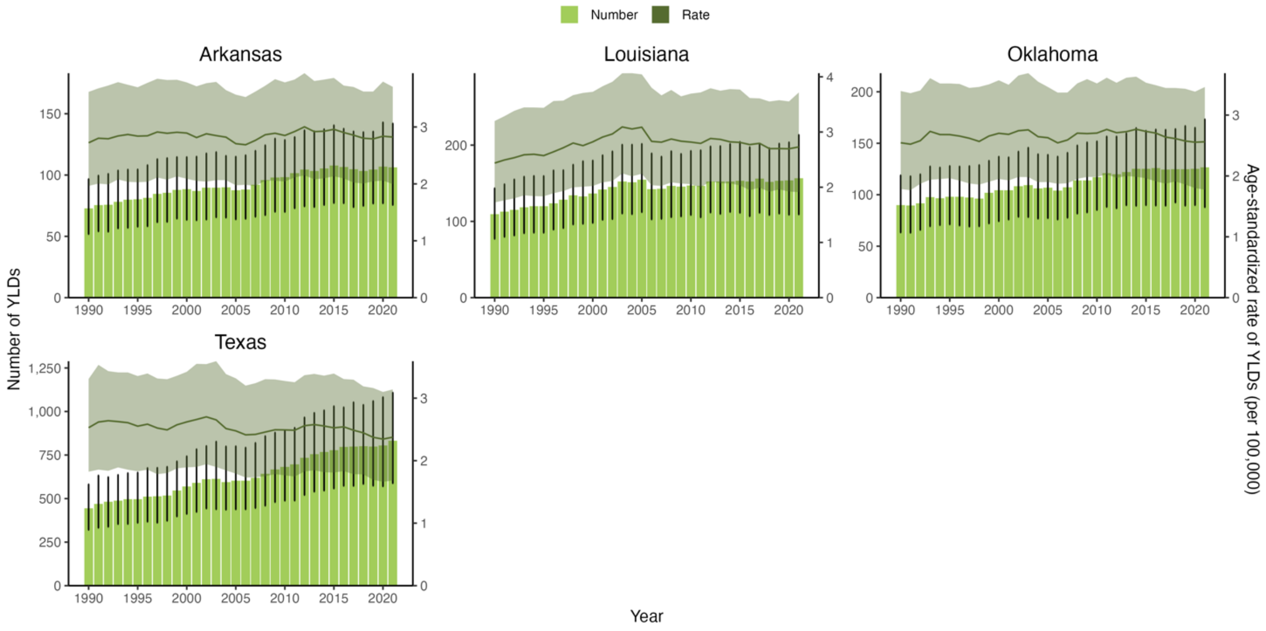

© 2025 GBD 2021 US CNS Cancer Collaborators. *JAMA Neurology*.

(H) Mountain

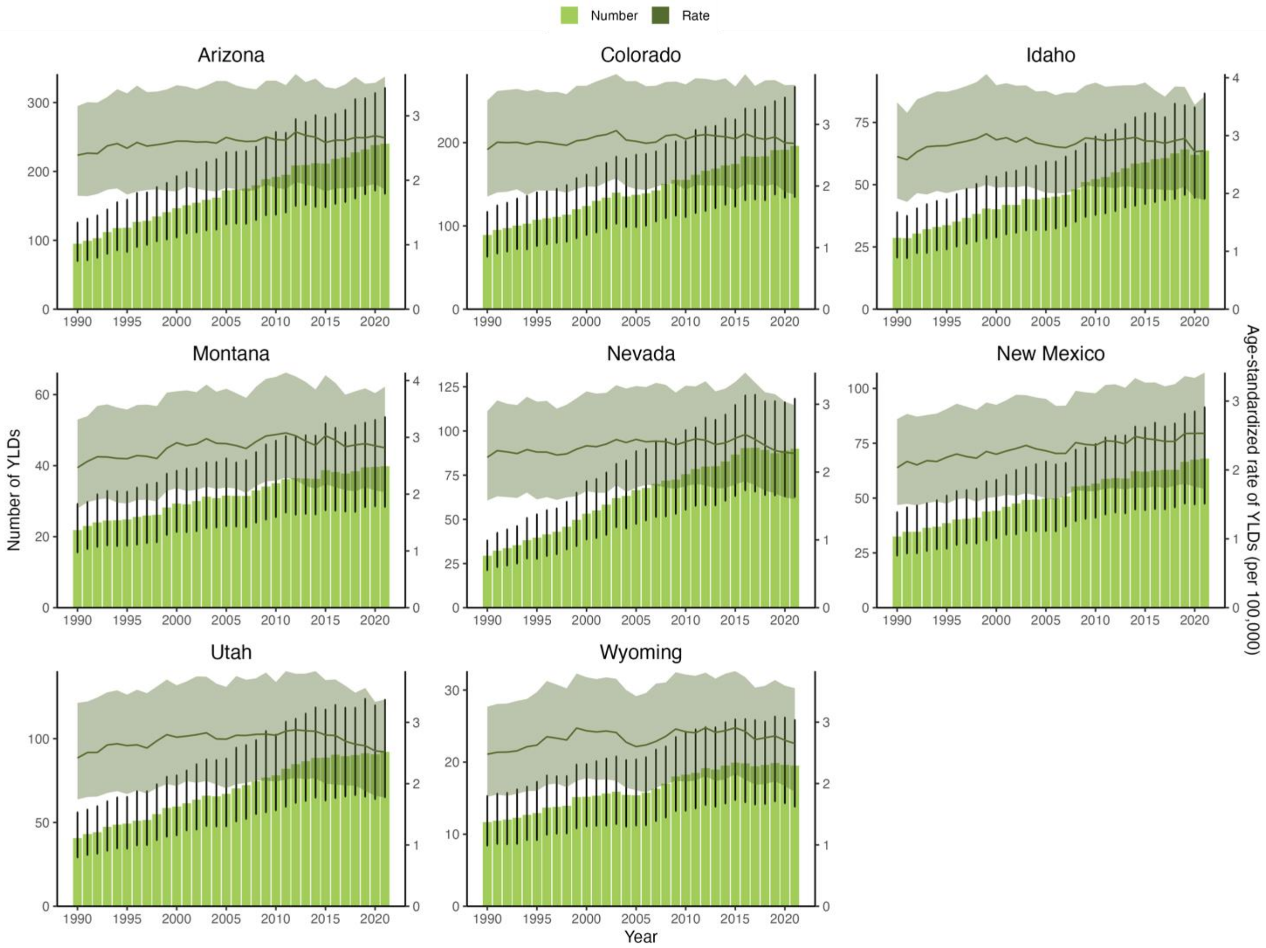

© 2025 GBD 2021 US CNS Cancer Collaborators. *JAMA Neurology*.

(I) Pacific

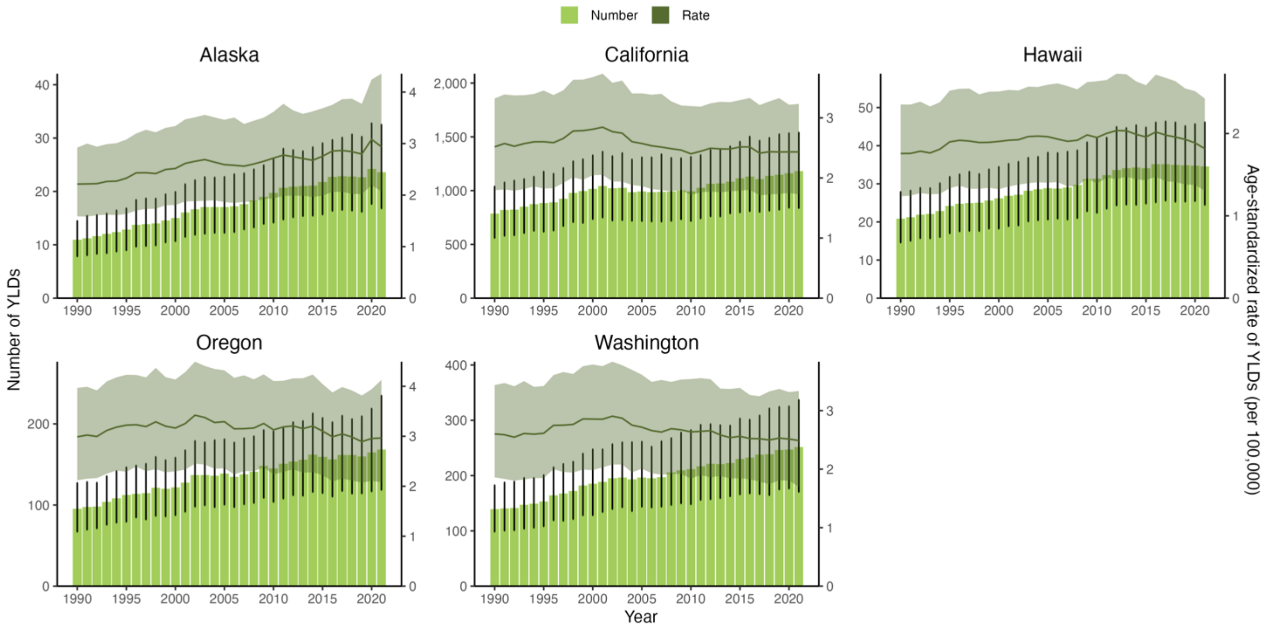

eFigure 1f: YLDs count and age-standardized rate of Brain and central nervous system cancer in the United States by division, (A) New England, (B) Middle Atlantic, (C) East North Central, (D) West North Central, (E) South Atlantic, (F) East South Central, (G) West South Central, (H) Mountain, (I) Pacific division, 1990 to 2021

© 2025 GBD 2021 US CNS Cancer Collaborators. *JAMA Neurology*.

(A) New England

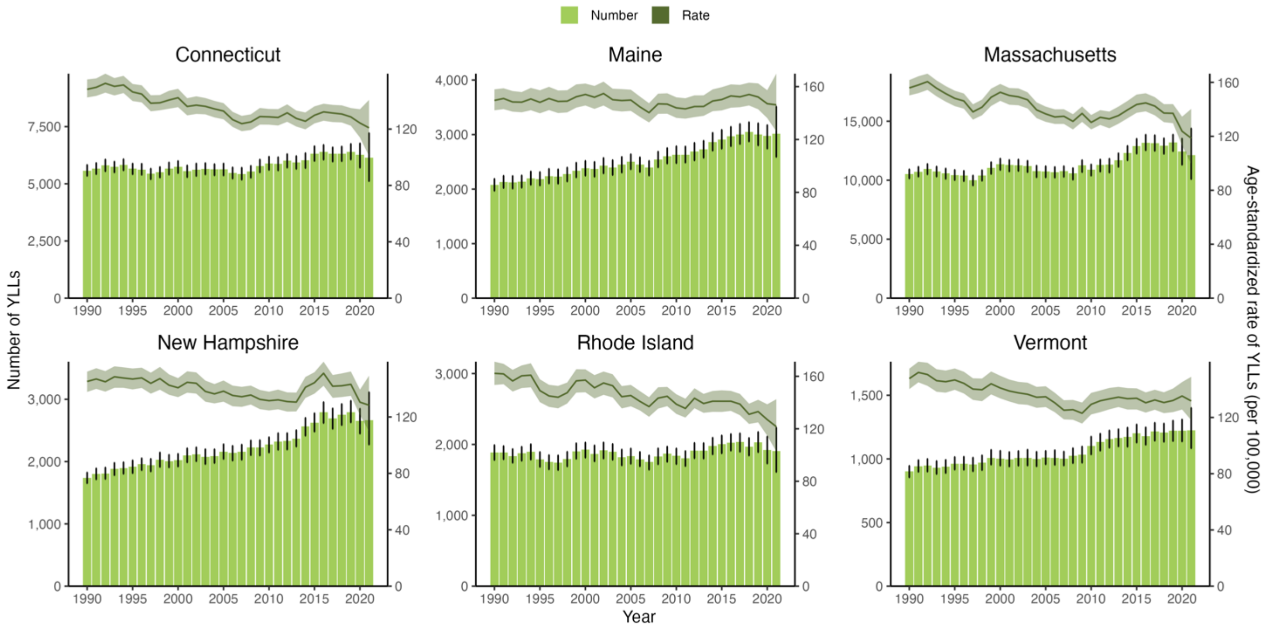

(B) Middle Atlantic

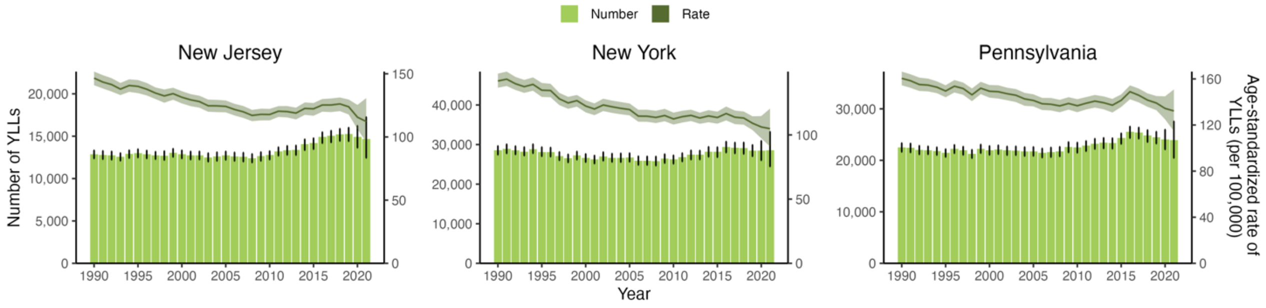

© 2025 GBD 2021 US CNS Cancer Collaborators. *JAMA Neurology*.

(C) East North Central

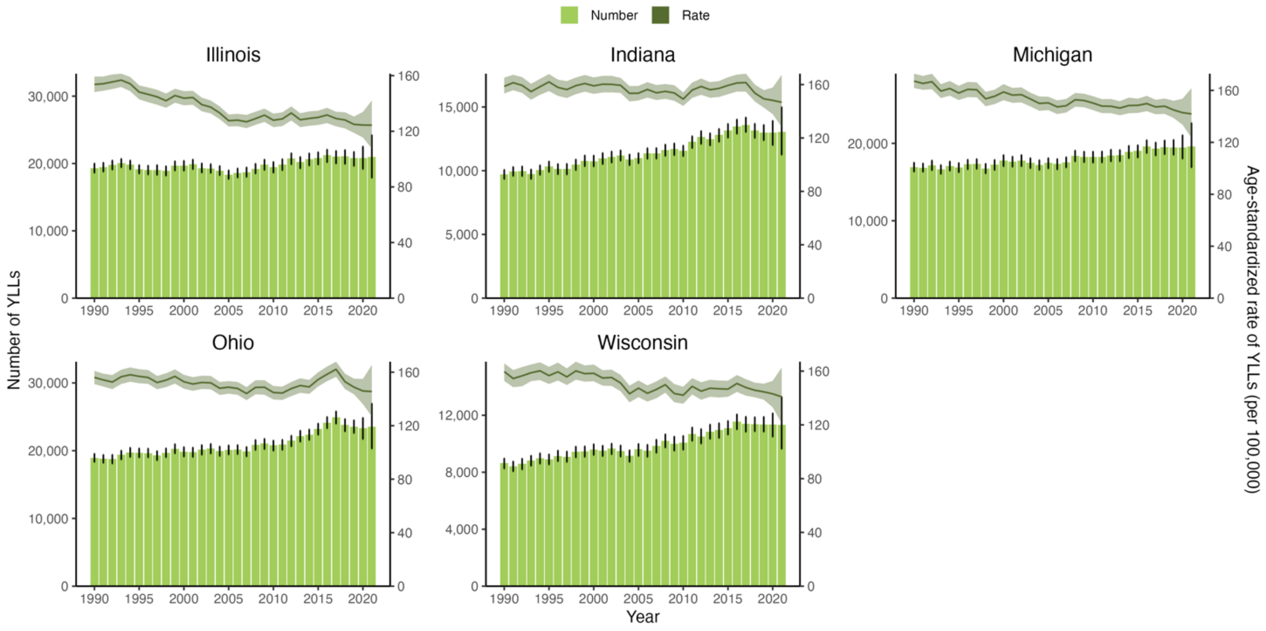

© 2025 GBD 2021 US CNS Cancer Collaborators. *JAMA Neurology*.

(D) West North Central

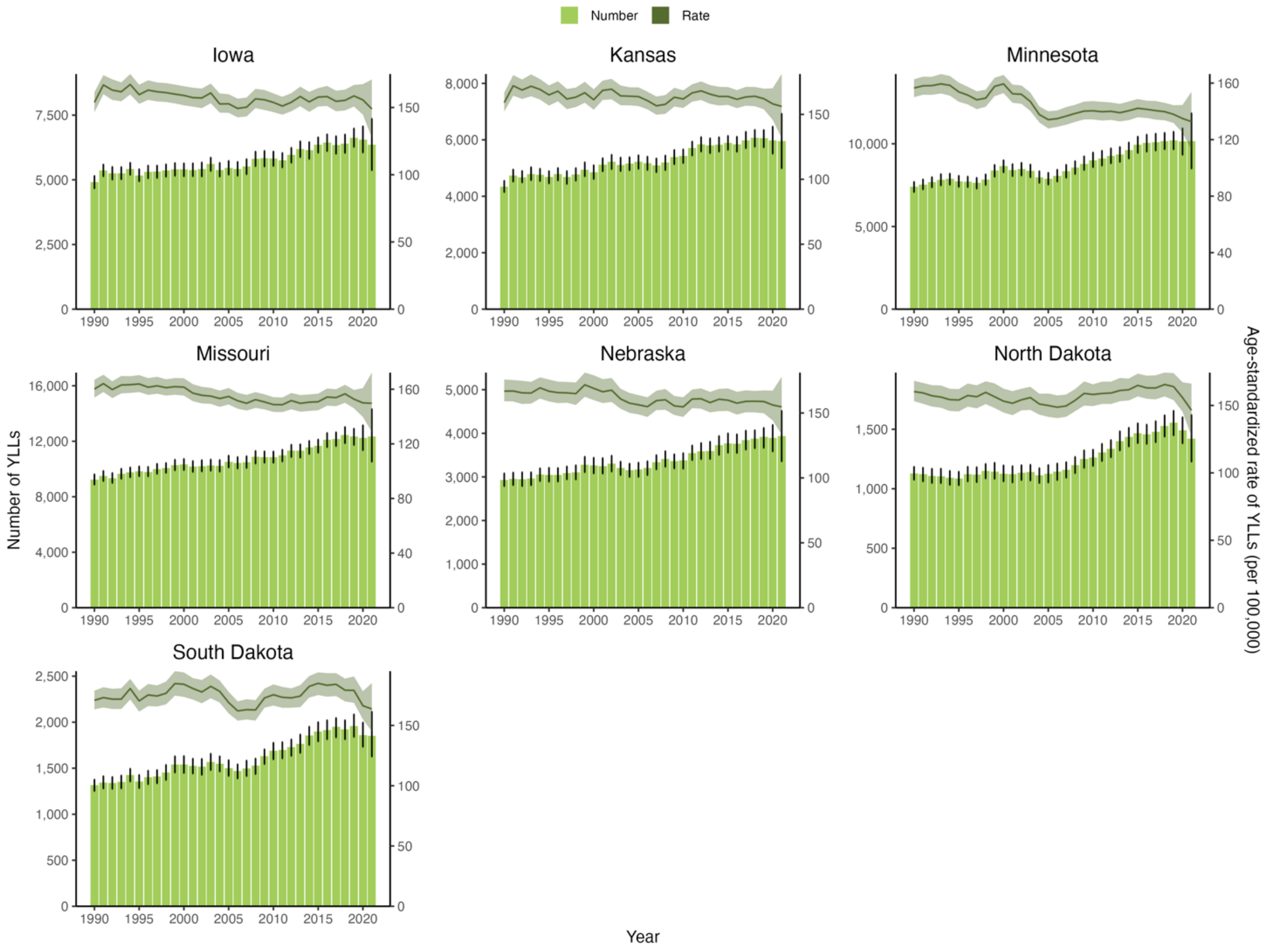

© 2025 GBD 2021 US CNS Cancer Collaborators. *JAMA Neurology*.

(E) South Atlantic

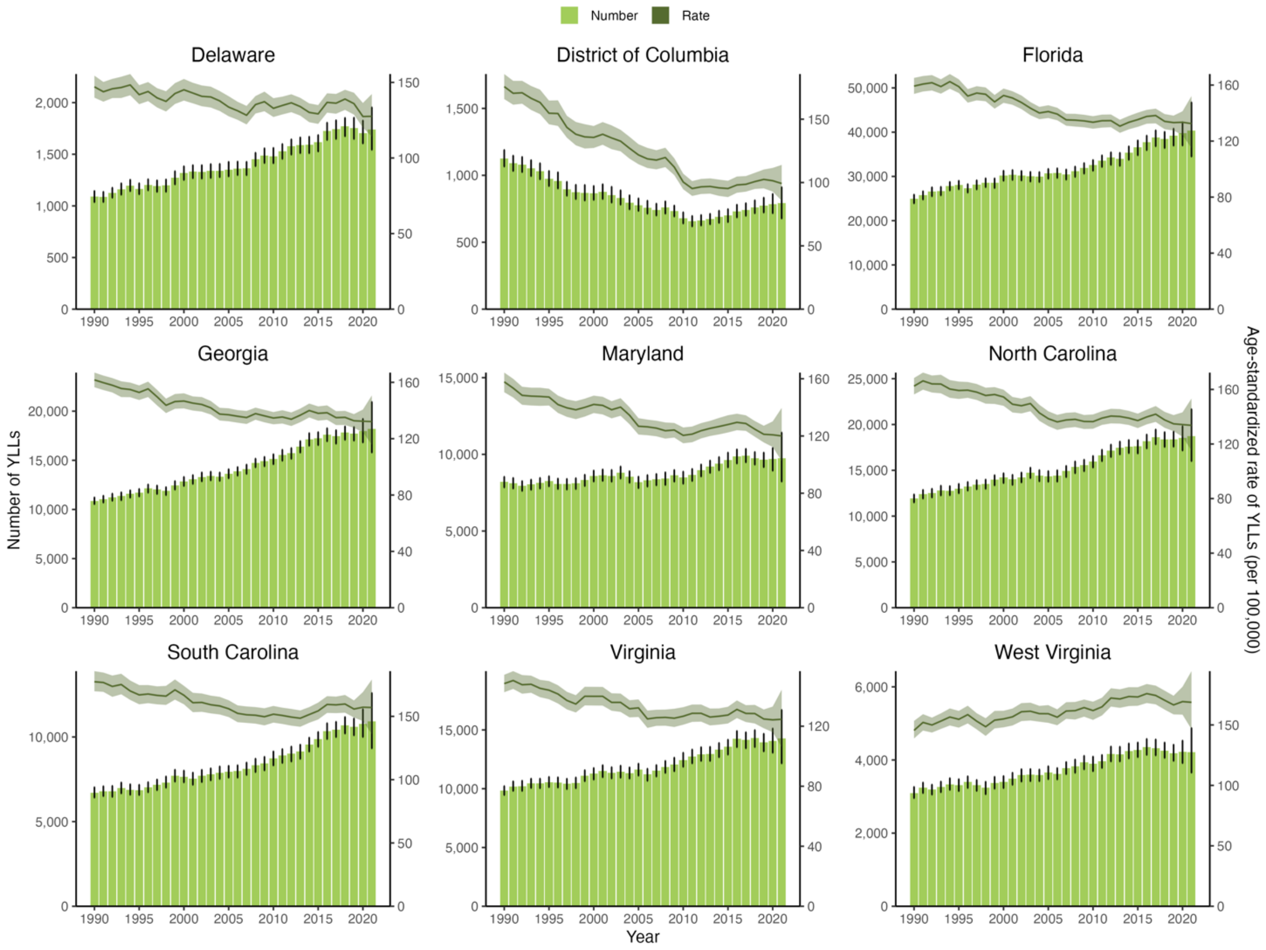

© 2025 GBD 2021 US CNS Cancer Collaborators. *JAMA Neurology*.

(F) East South Central

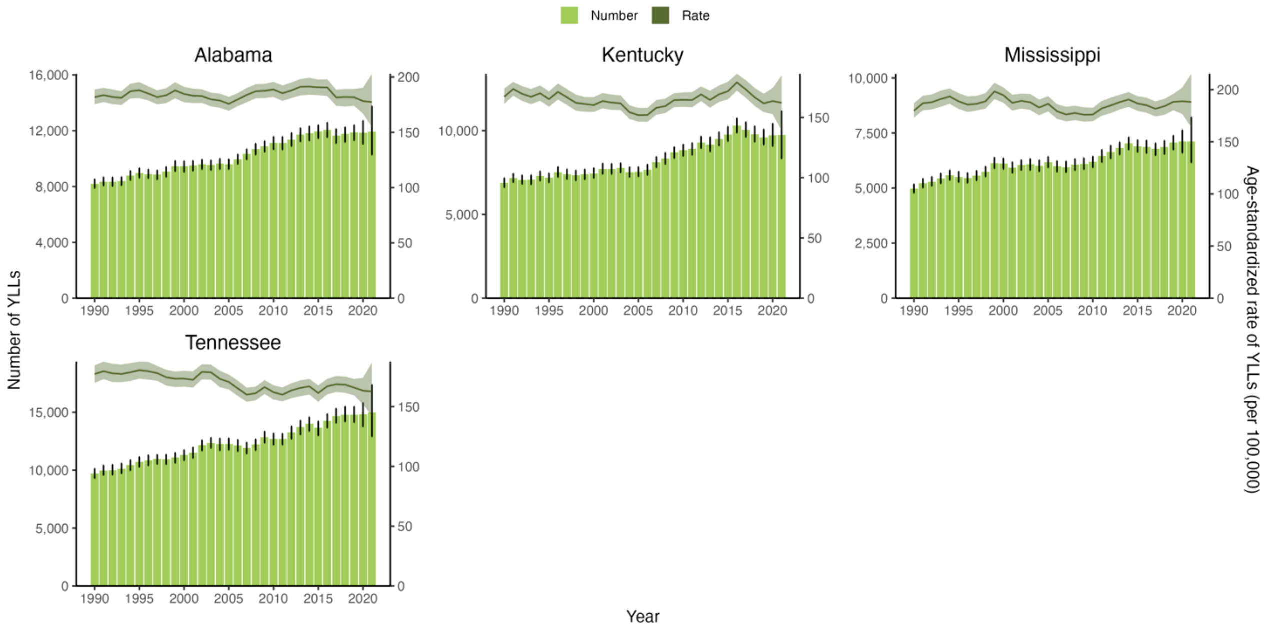

(G) West South Central

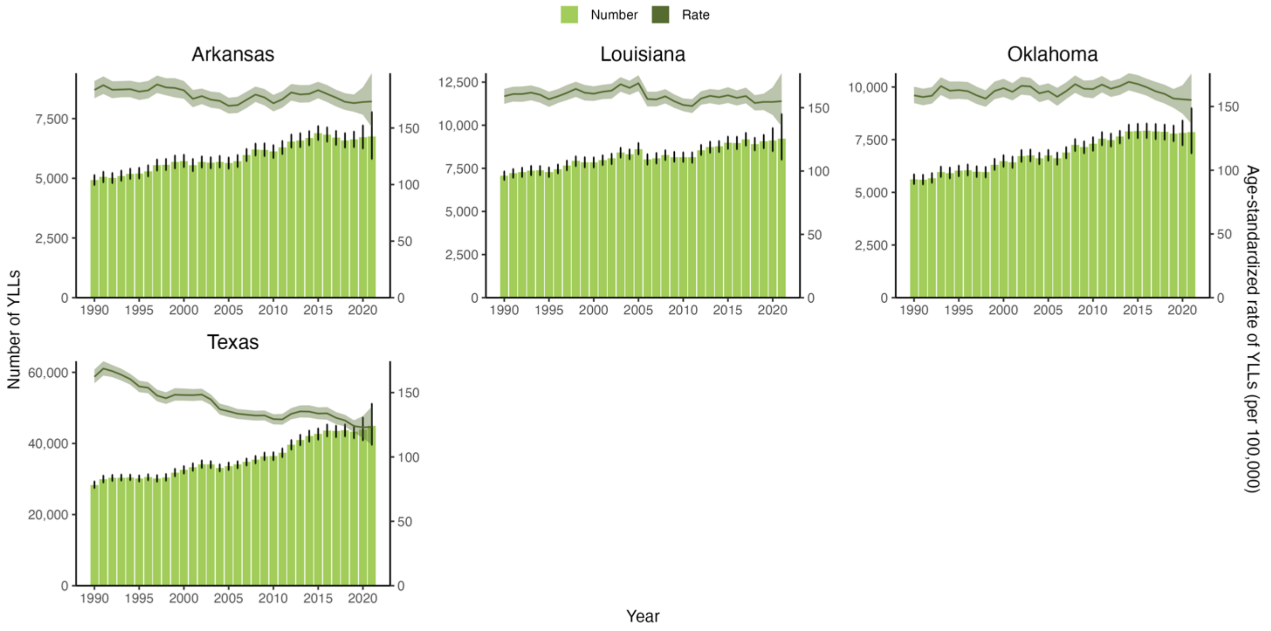

© 2025 GBD 2021 US CNS Cancer Collaborators. *JAMA Neurology*.

(H) Mountain

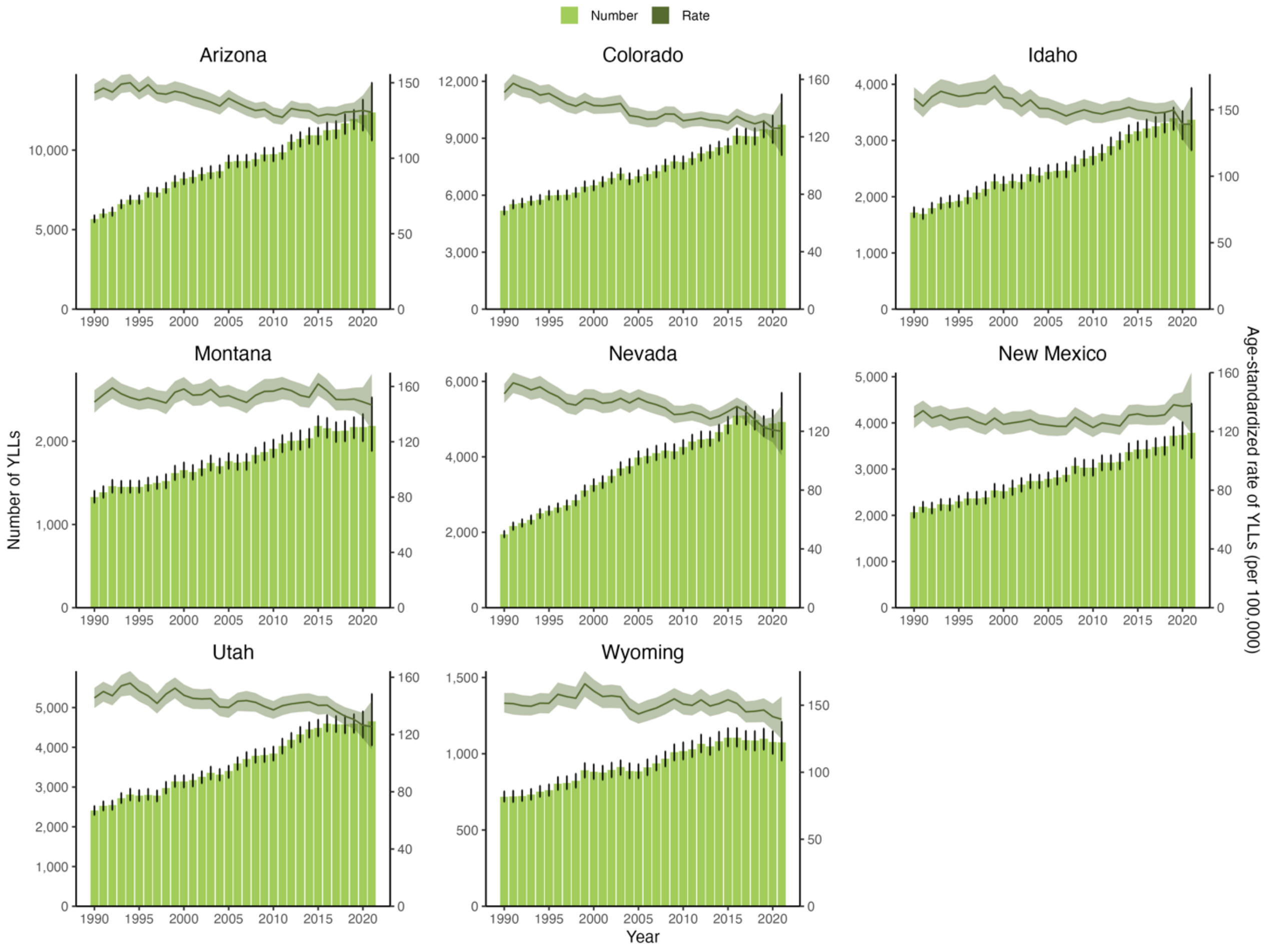

© 2025 GBD 2021 US CNS Cancer Collaborators. *JAMA Neurology*.

(I) Pacific

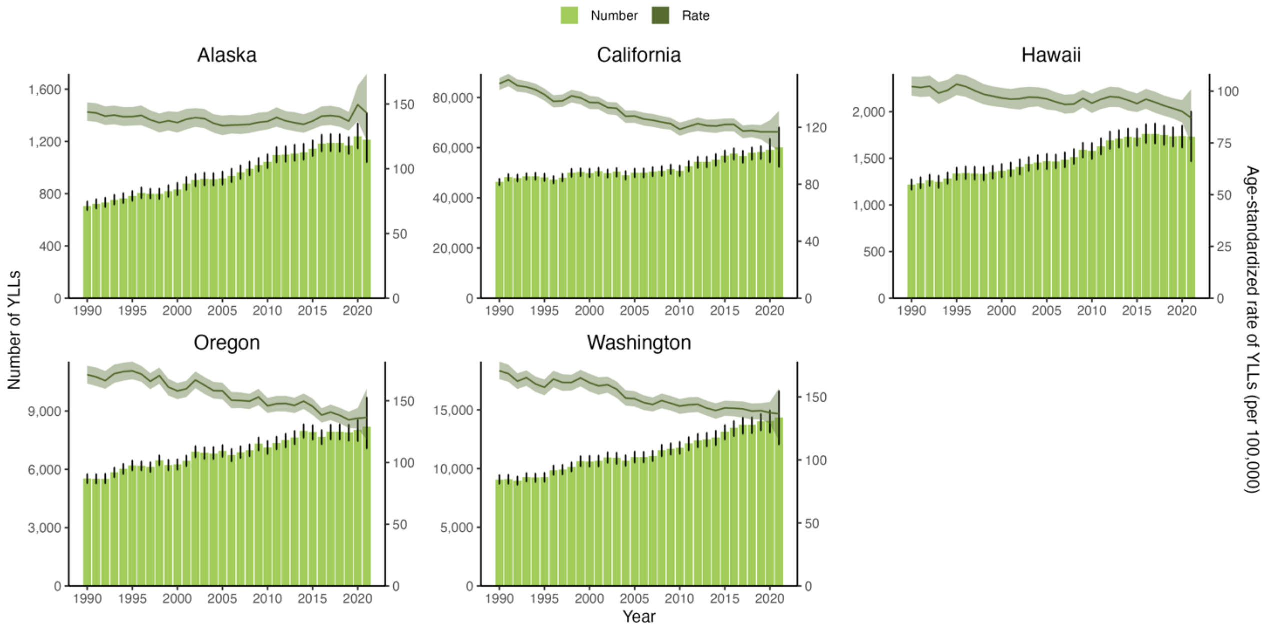

eFigure 1g: YLLs count and age-standardized rate of Brain and central nervous system cancer in the United States by division, (A) New England, (B) Middle Atlantic, (C) East North Central, (D) West North Central, (E) South Atlantic, (F) East South Central, (G) West South Central, (H) Mountain, (I) Pacific division, 1990 to 2021

© 2025 GBD 2021 US CNS Cancer Collaborators. *JAMA Neurology*.

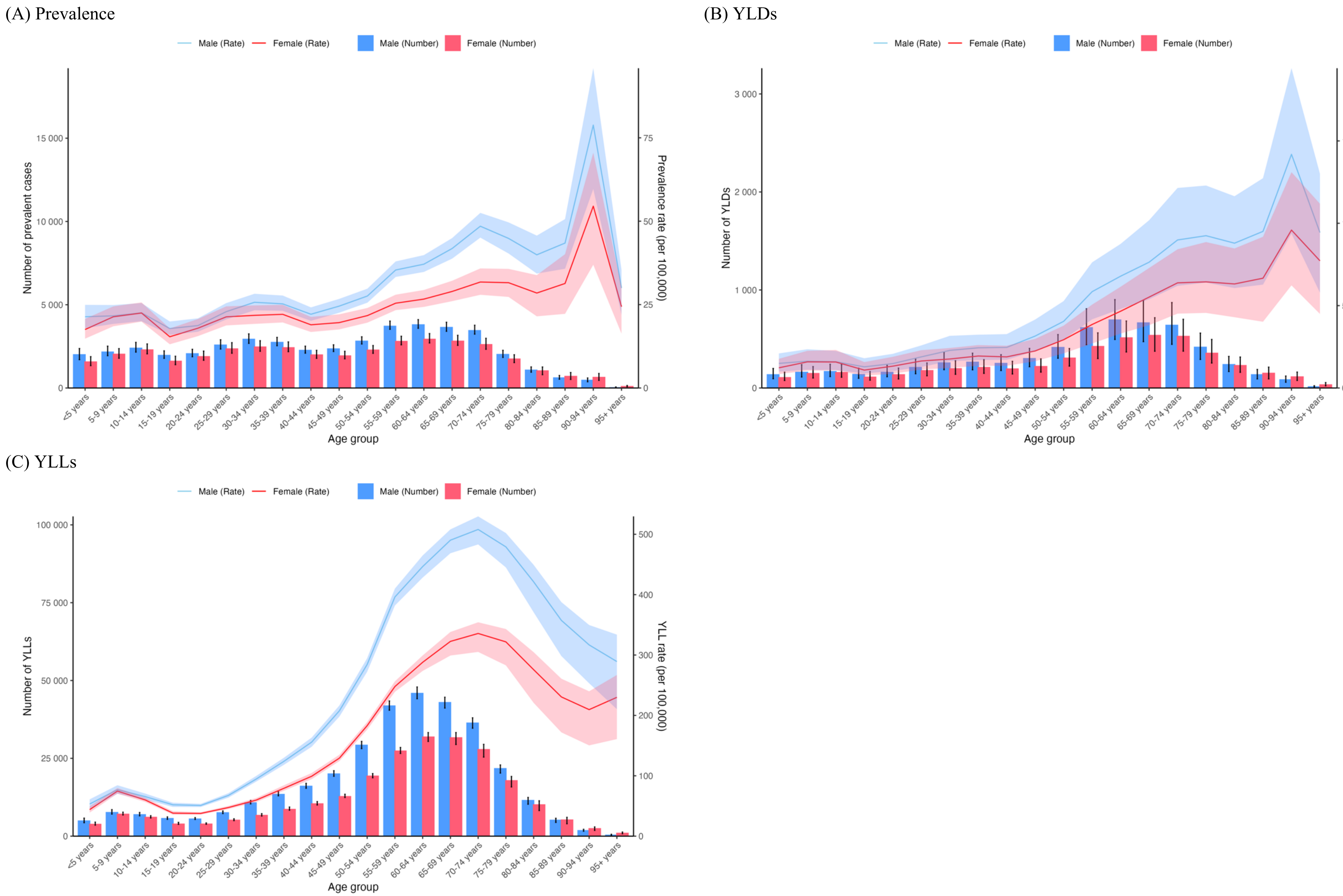

eFigure 2: (A) prevalence (B) YLDs (C) YLLs count and age-standardized rate of Brain and central nervous system cancer by sex, United States, 1990 to 2021

© 2025 GBD 2021 US CNS Cancer Collaborators. *JAMA Neurology*.

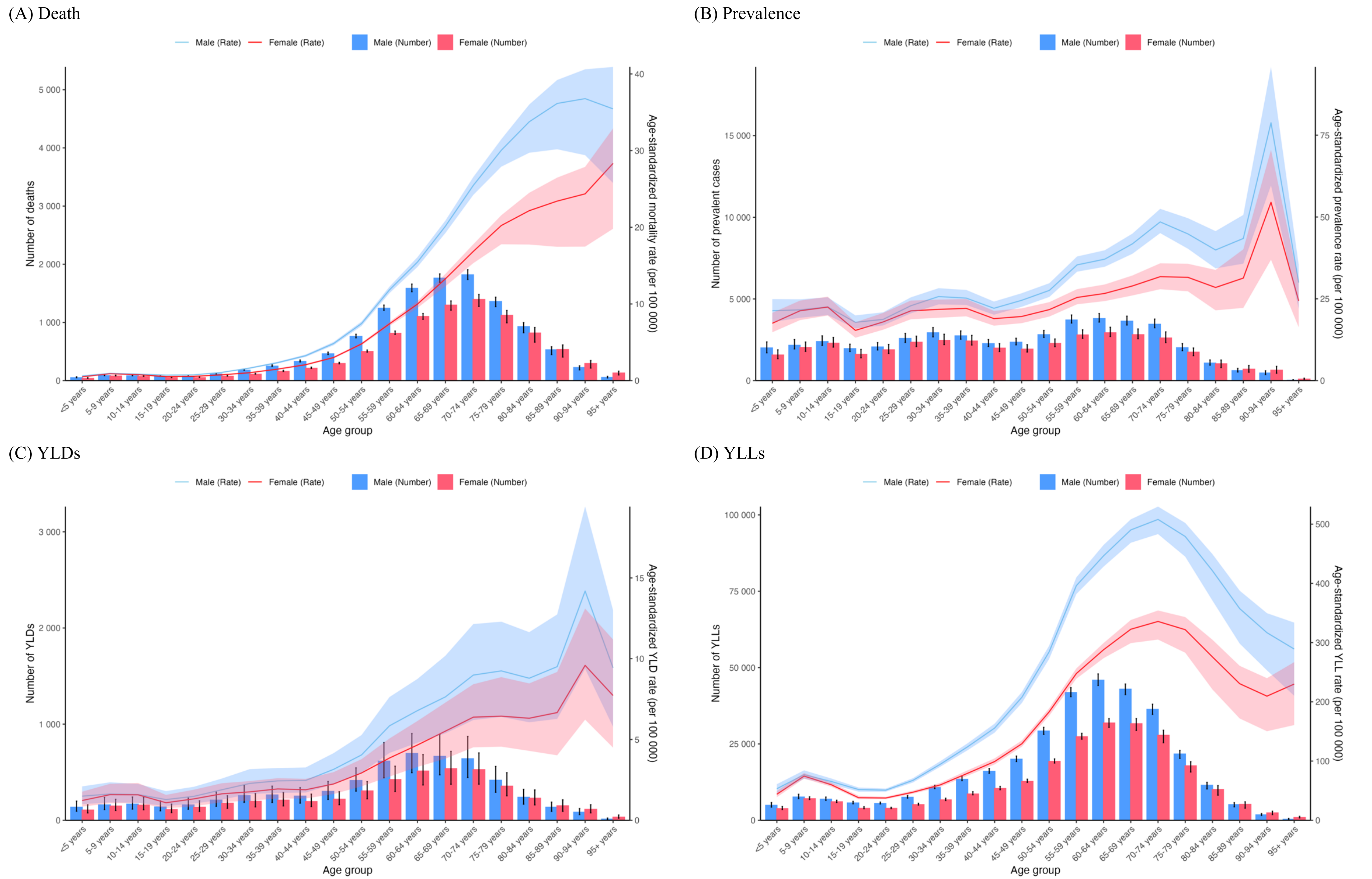

eFigure 3: (A) Death (B) prevalence (C) YLDs (D) YLLs count and rate of Brain and central nervous system cancer by sex and age, United States, 2021

© 2025 GBD 2021 US CNS Cancer Collaborators. *JAMA Neurology*.

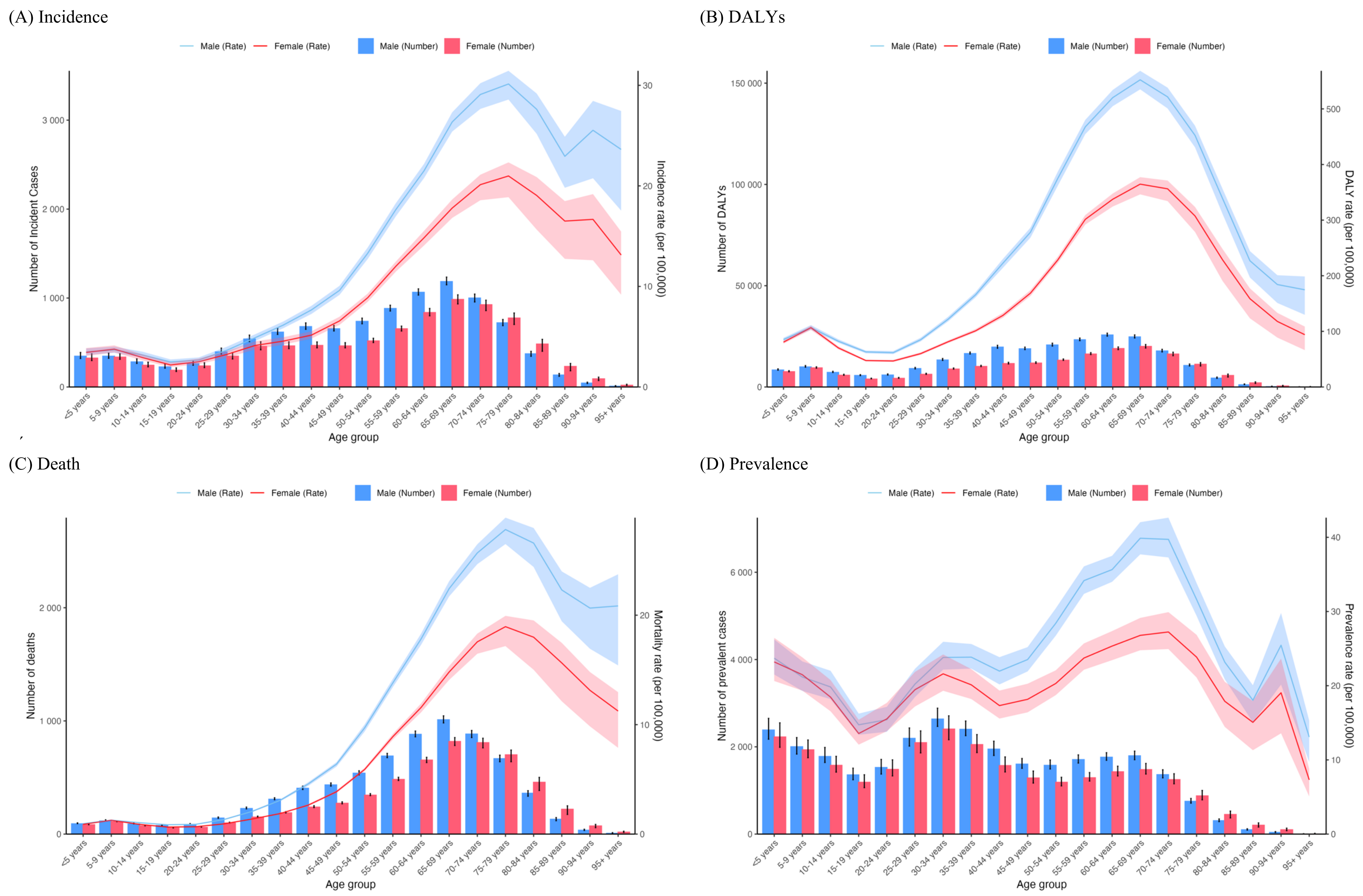

© 2025 GBD 2021 US CNS Cancer Collaborators. *JAMA Neurology*.

(E) YLDs

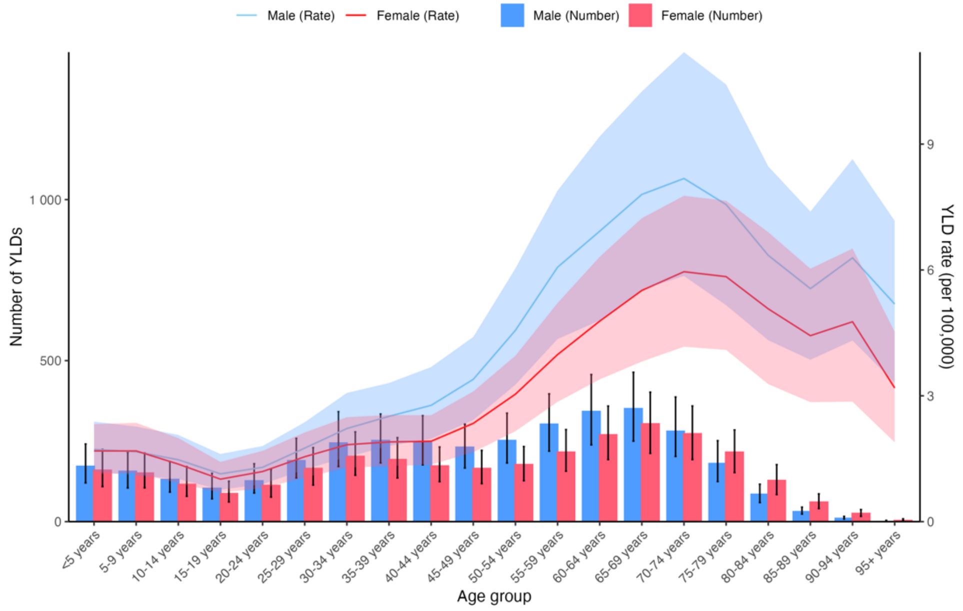

(F) YLLs

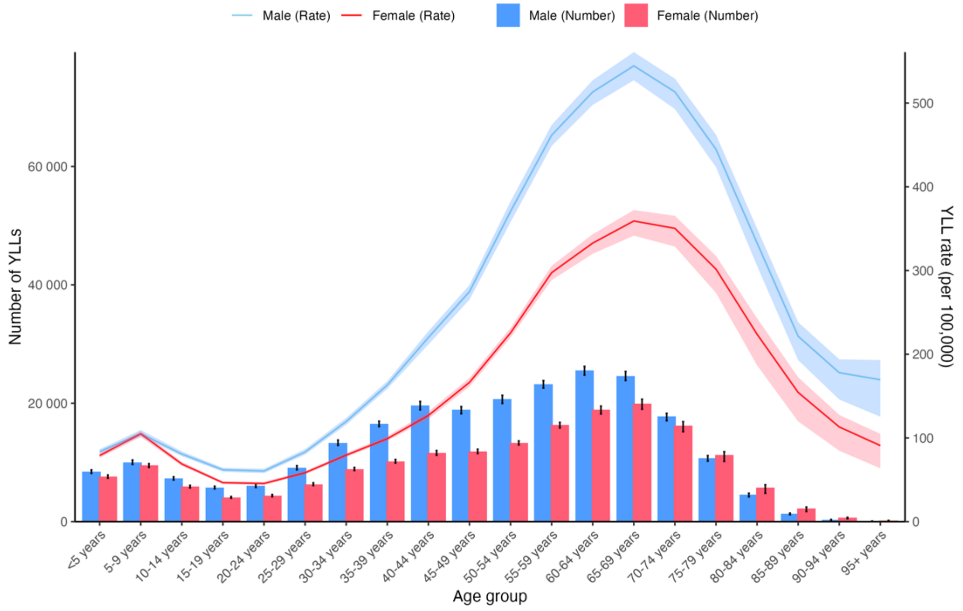

eFigure 4: (A) incidence (B) DALYs (C) death (D) prevalence (E) YLDs (F) YLLs count and rate of Brain and central nervous system cancer by sex and age, United States, 1990

© 2025 GBD 2021 US CNS Cancer Collaborators. *JAMA Neurology*.

(A) Incidence

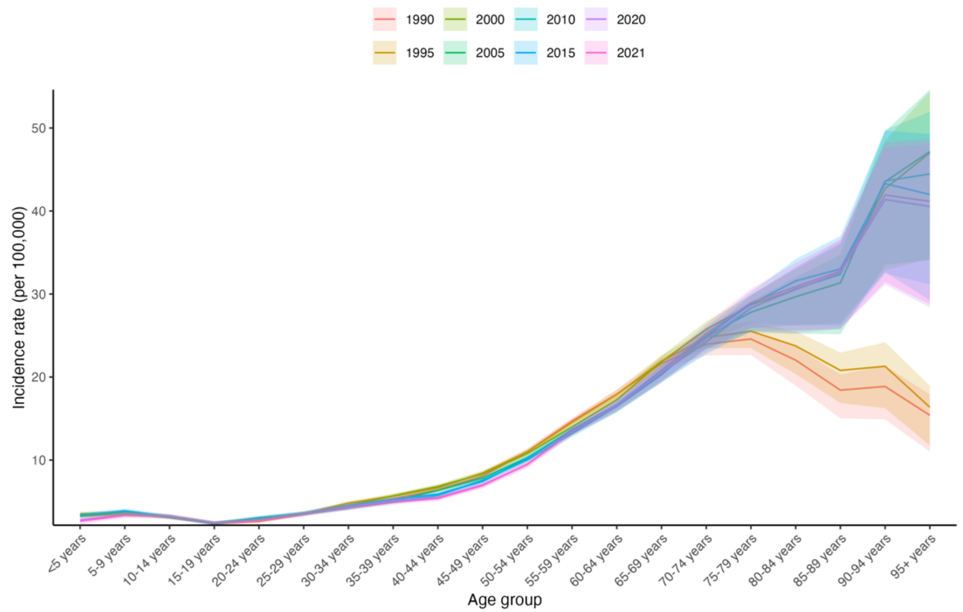

(B) DALYs

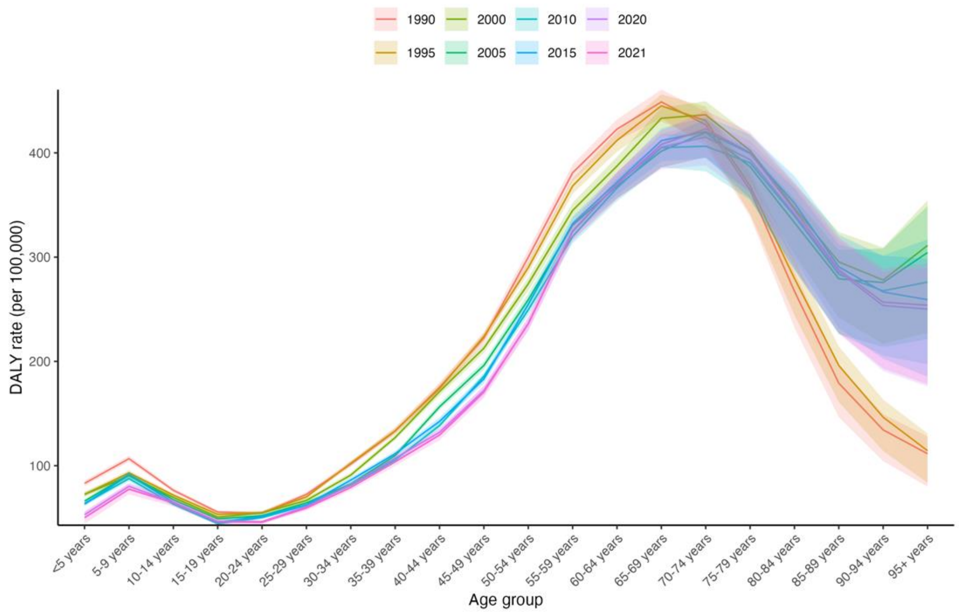

(C) Death

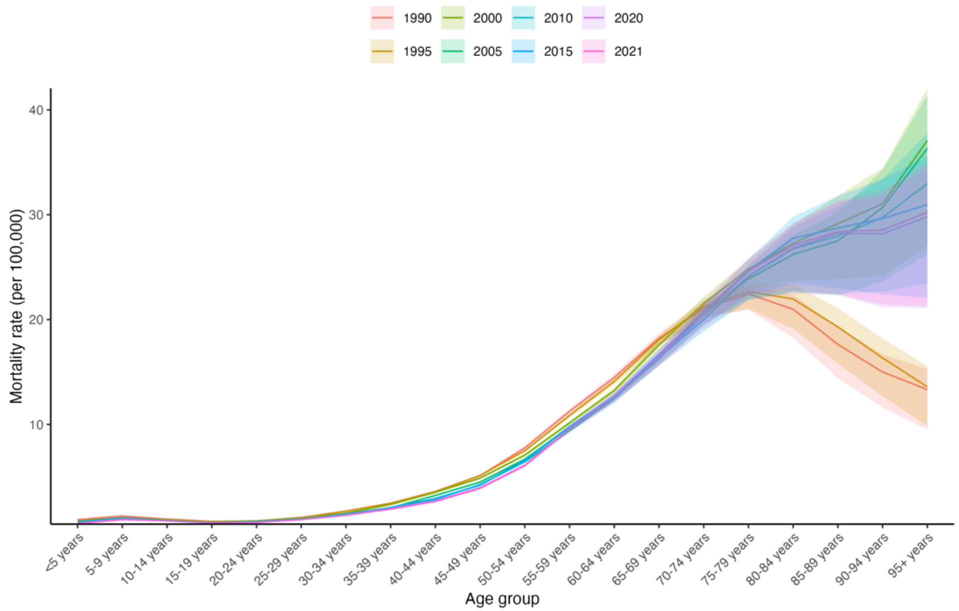

(D) Prevalence

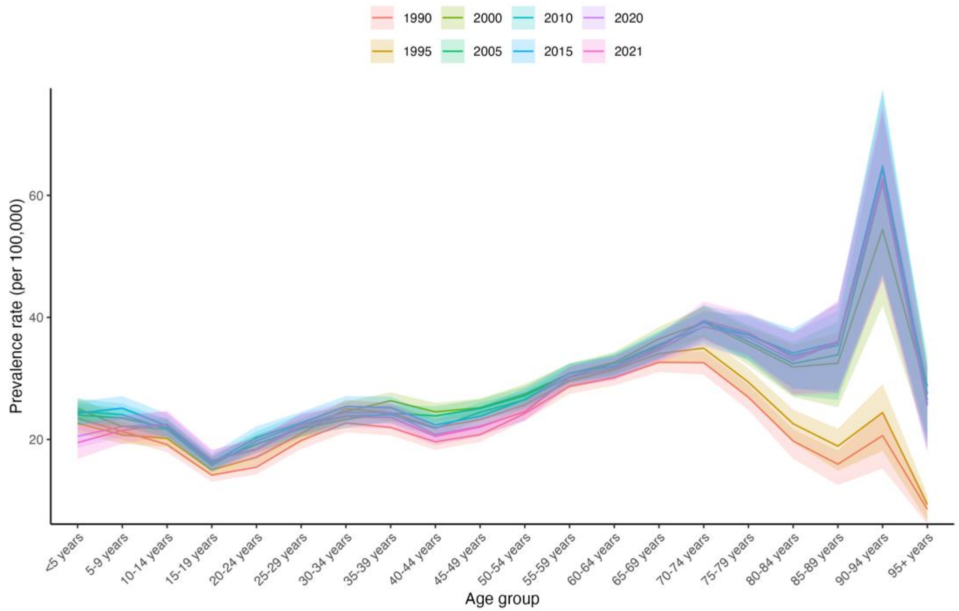

(E) YLDs

(F) YLLs

© 2025 GBD 2021 US CNS Cancer Collaborators. *JAMA Neurology*.

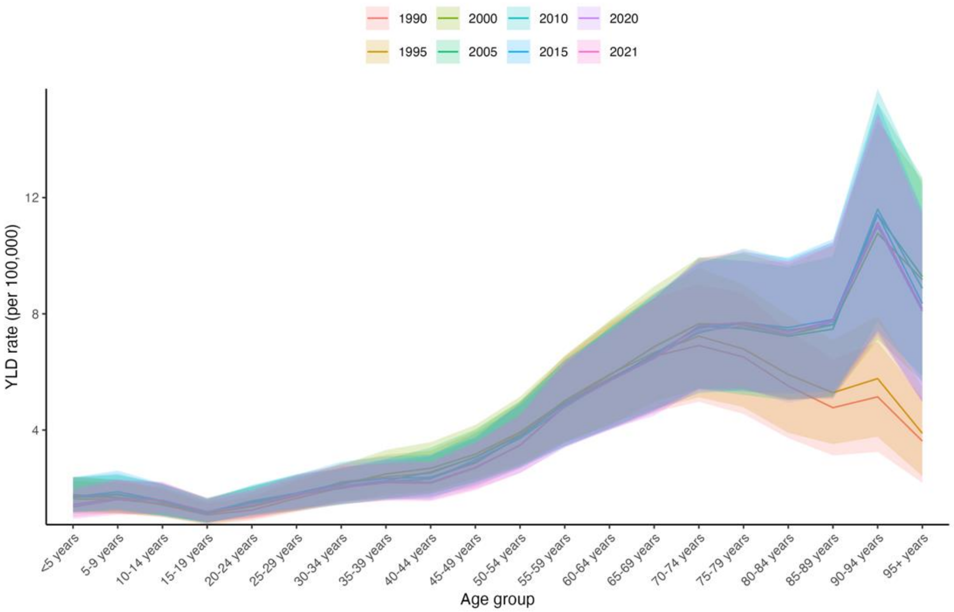

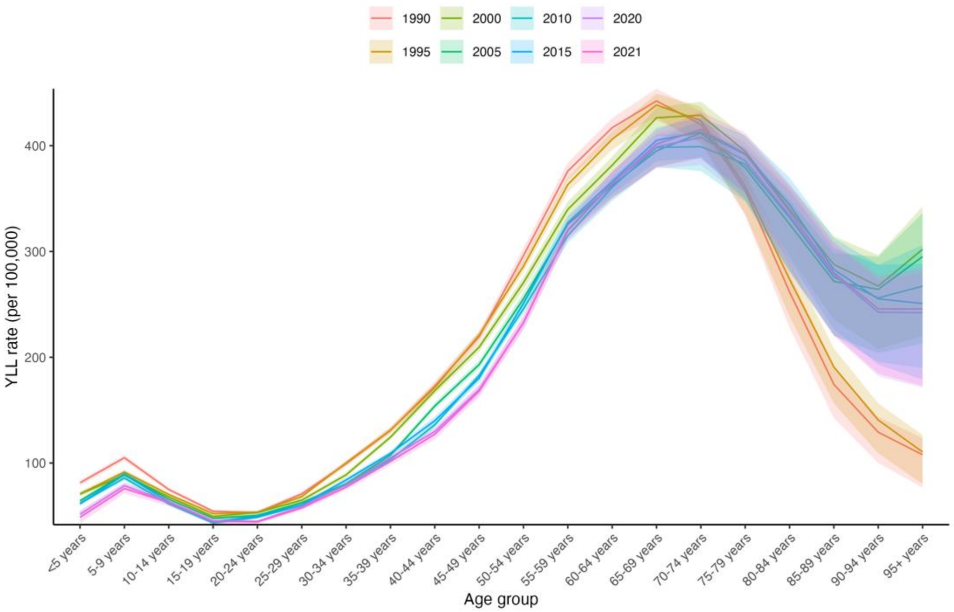

eFigure 5: (A) incidence (B) DALYs (C) death (D) prevalence (E) YLDs (F) YLLs count and rate of Brain and central nervous system cancer by age, United States, 1990~2021

© 2025 GBD 2021 US CNS Cancer Collaborators. *JAMA Neurology*.

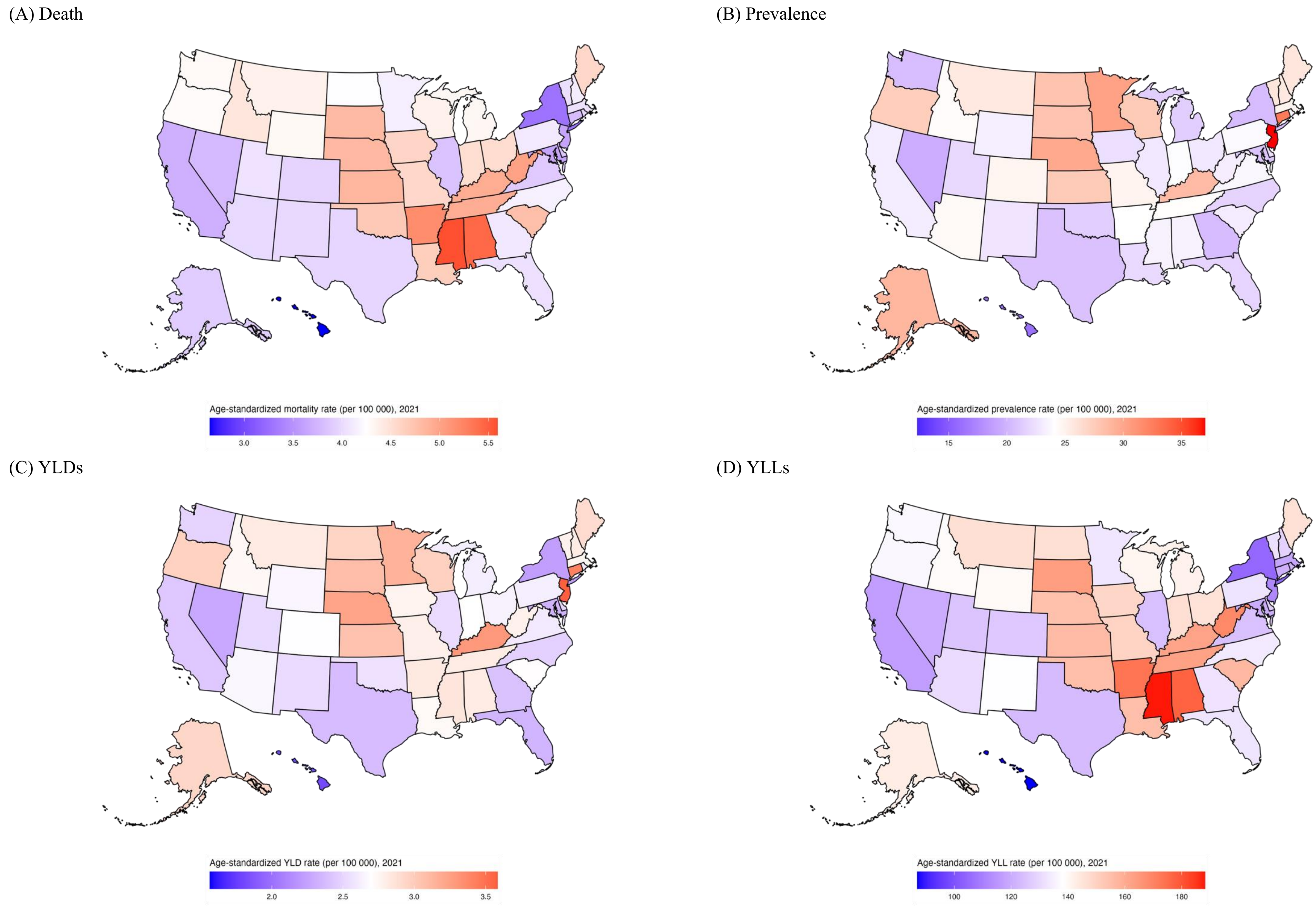

eFigure 6: Age-standardized (A) Death (B) prevalence (C) YLDs (D) YLLs rate of Brain and central nervous system cancer in the United States, 2021

© 2025 GBD 2021 US CNS Cancer Collaborators. *JAMA Neurology*.

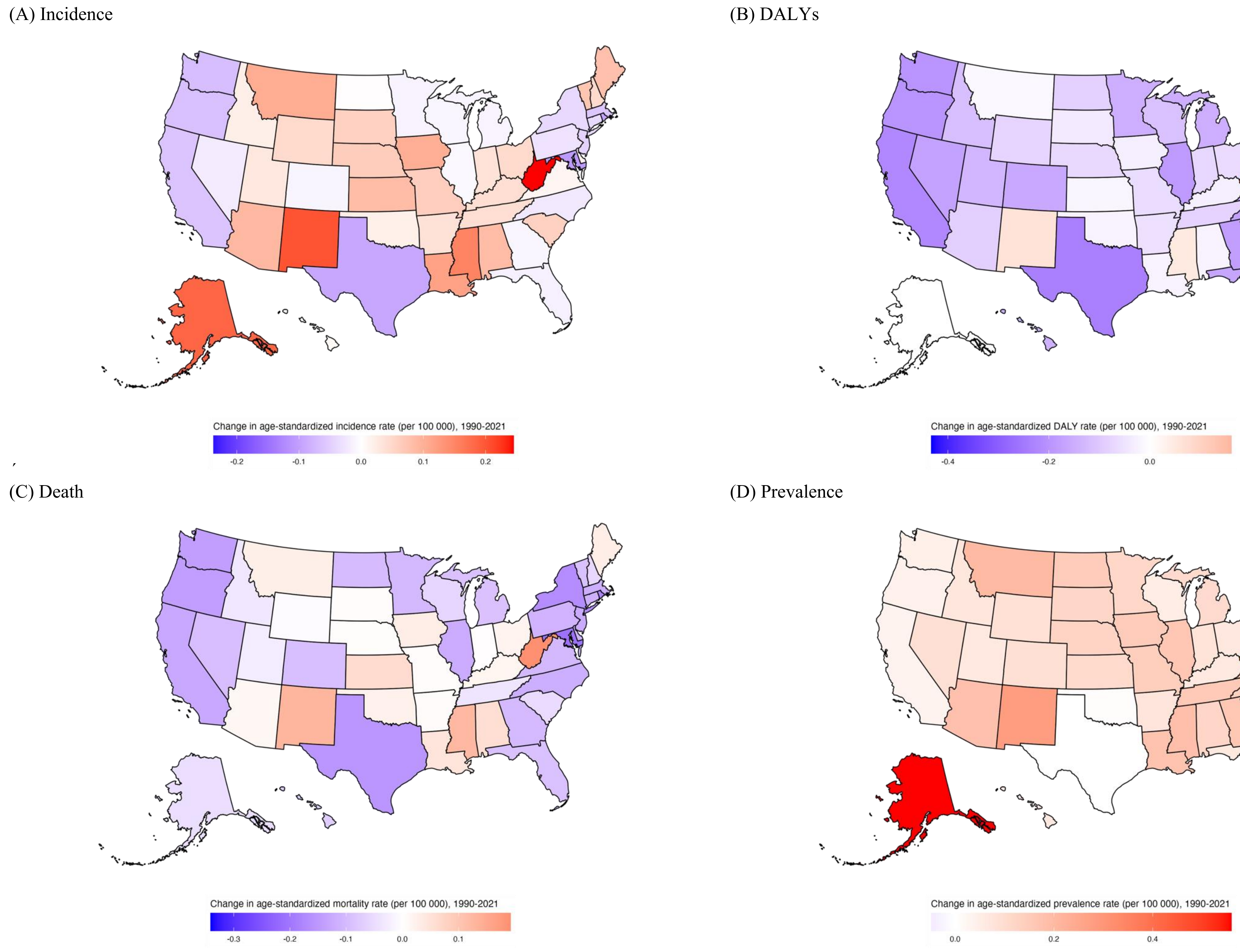

© 2025 GBD 2021 US CNS Cancer Collaborators. *JAMA Neurology*.

(E) YLDs

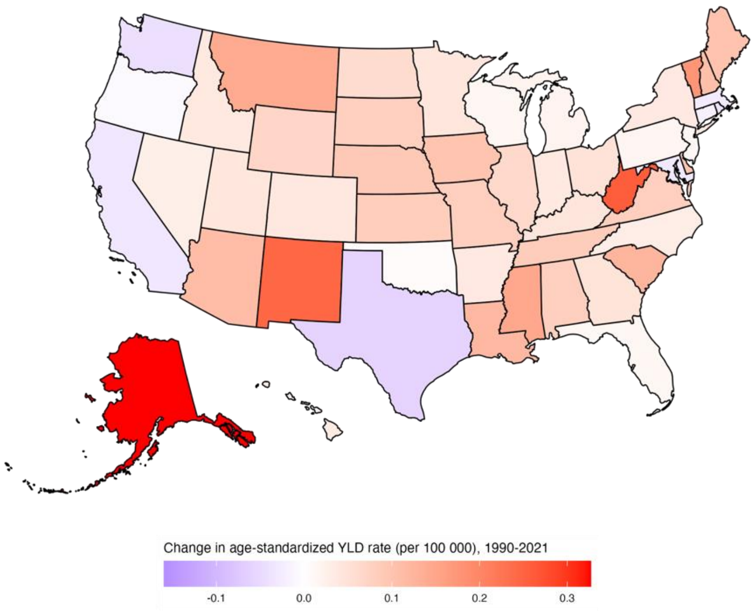

(F) YLLs

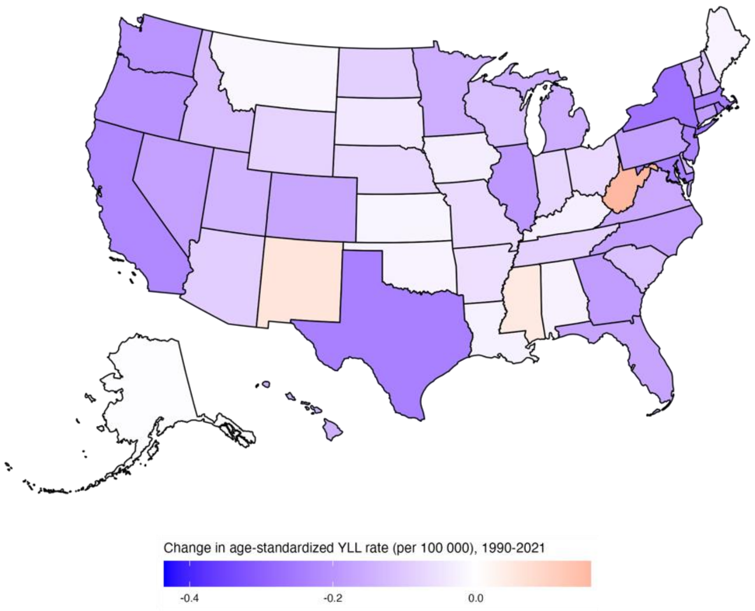

eFigure 7: Change in age-standardized (A) incidence (B) DALYs (C) death (D) prevalence (E) YLDs (F) YLLs rate of Brain and central nervous system cancer in the United States, 1990-2021

© 2025 GBD 2021 US CNS Cancer Collaborators. *JAMA Neurology*.

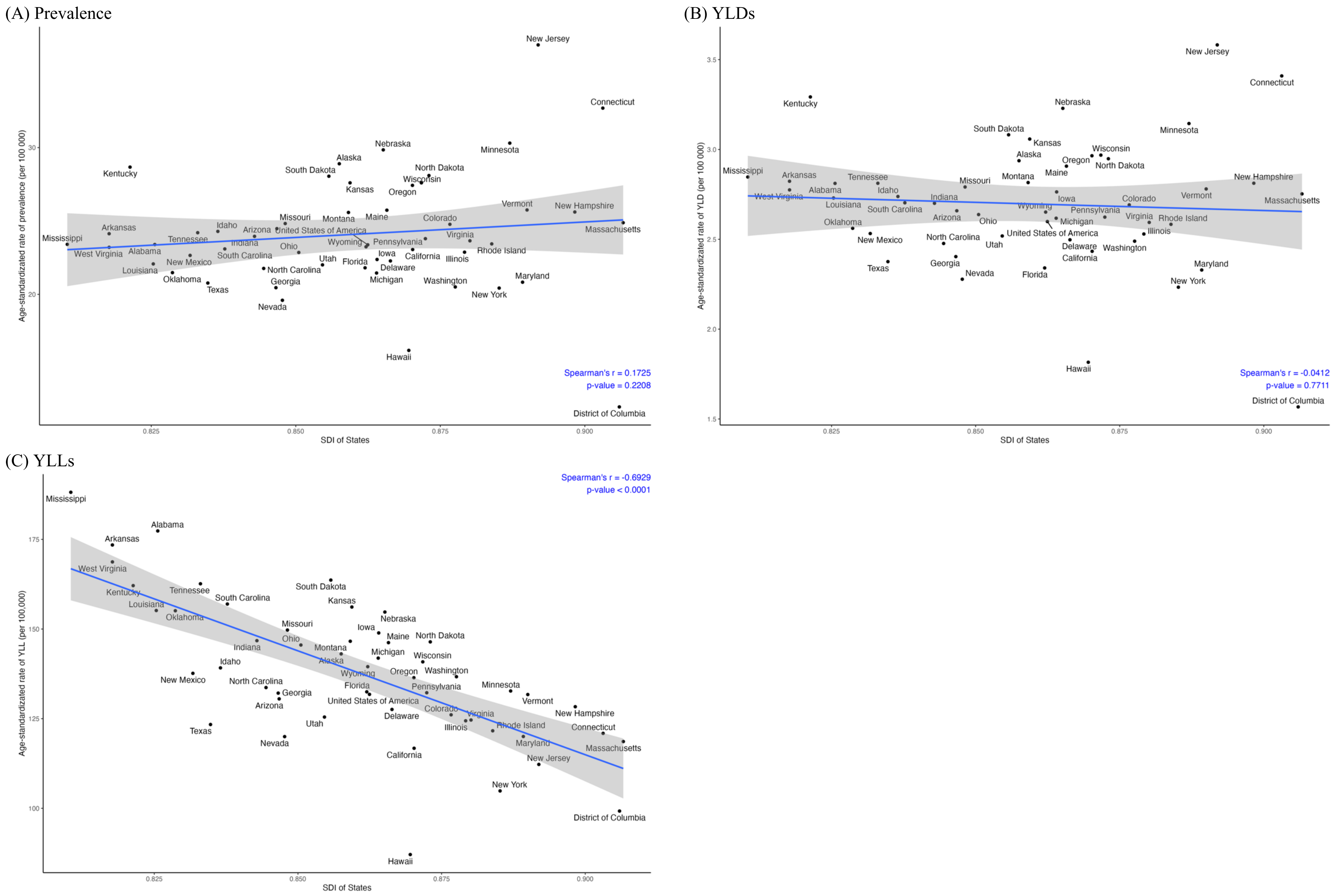

© 2025 GBD 2021 US CNS Cancer Collaborators. *JAMA Neurology*.

eFigure 8: Correlation between SDI and age-standardized (A) prevalence (B) YLDs (C) YLLs rate of Brain and central nervous system cancer in the United States by state, 2021

© 2025 GBD 2021 US CNS Cancer Collaborators. *JAMA Neurology*.

(A) Incidence

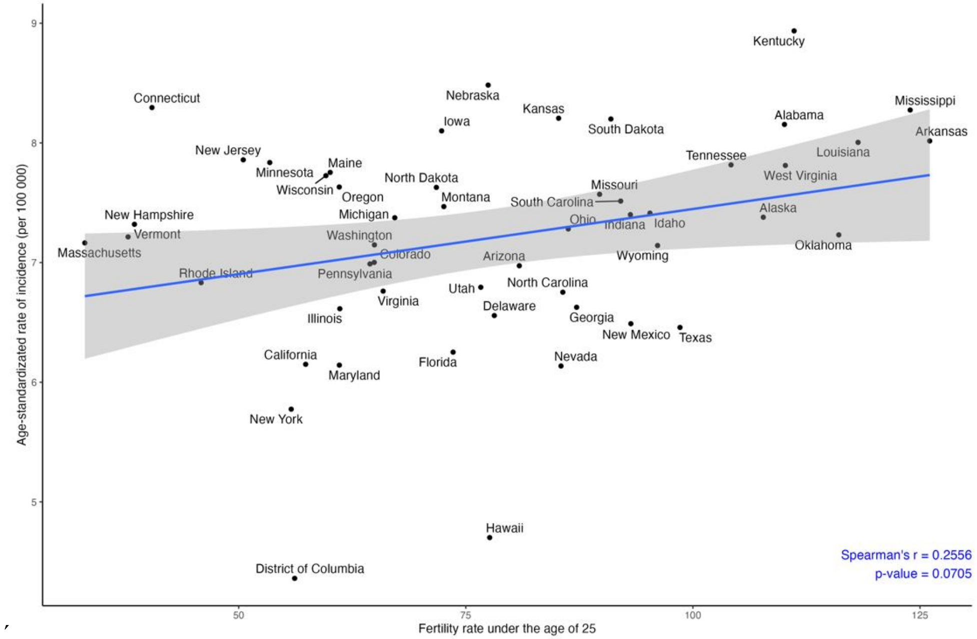

(B) DALYs

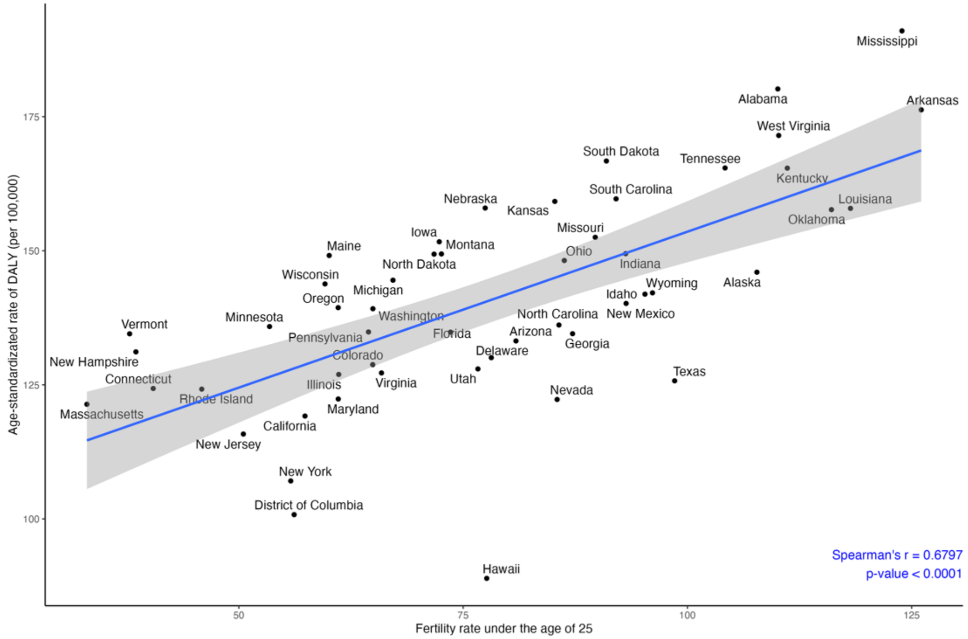

(C) Death

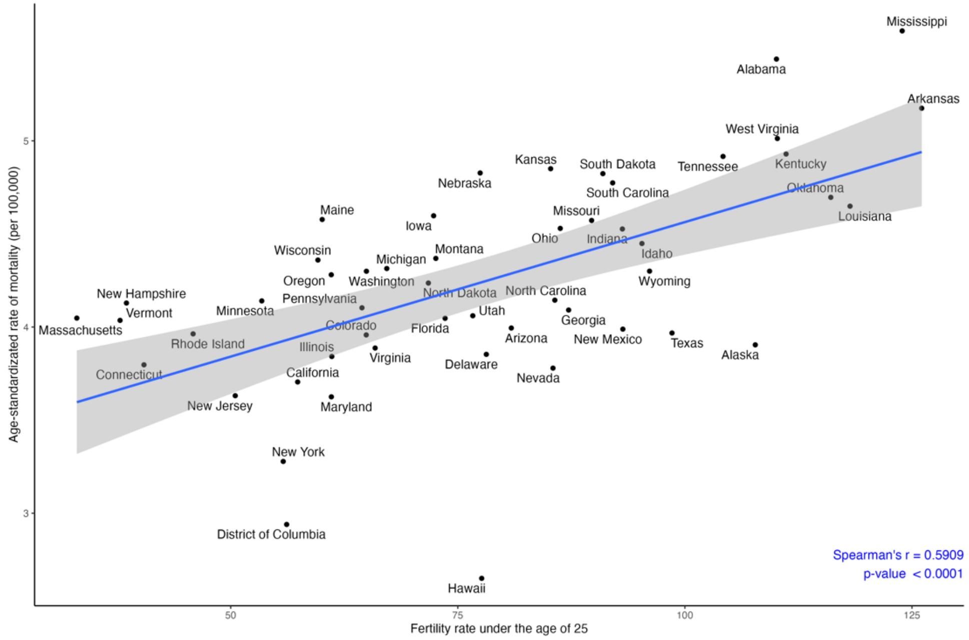

(D) Prevalence

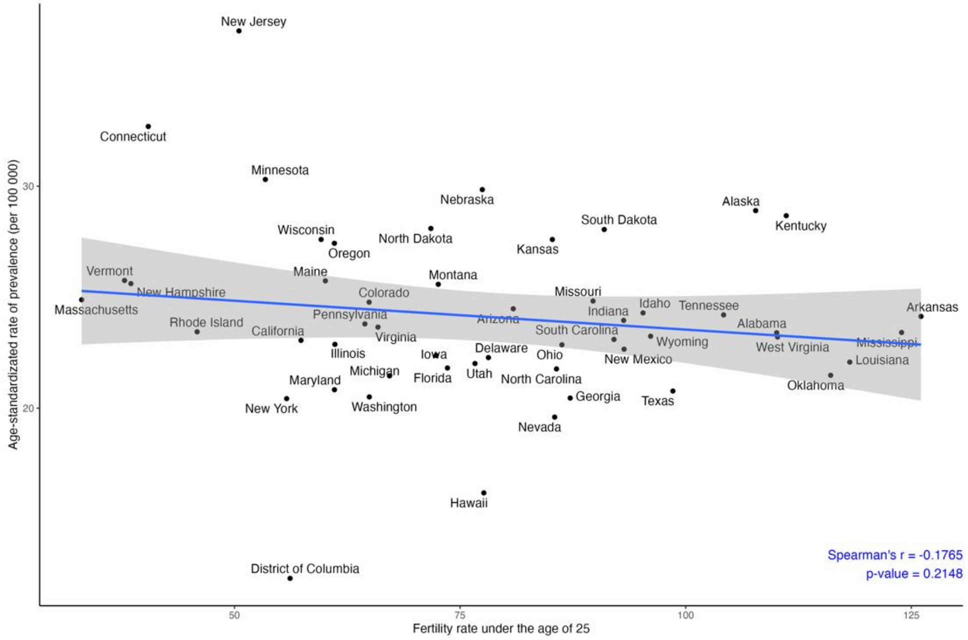

© 2025 GBD 2021 US CNS Cancer Collaborators. *JAMA Neurology*.

(E) YLDs

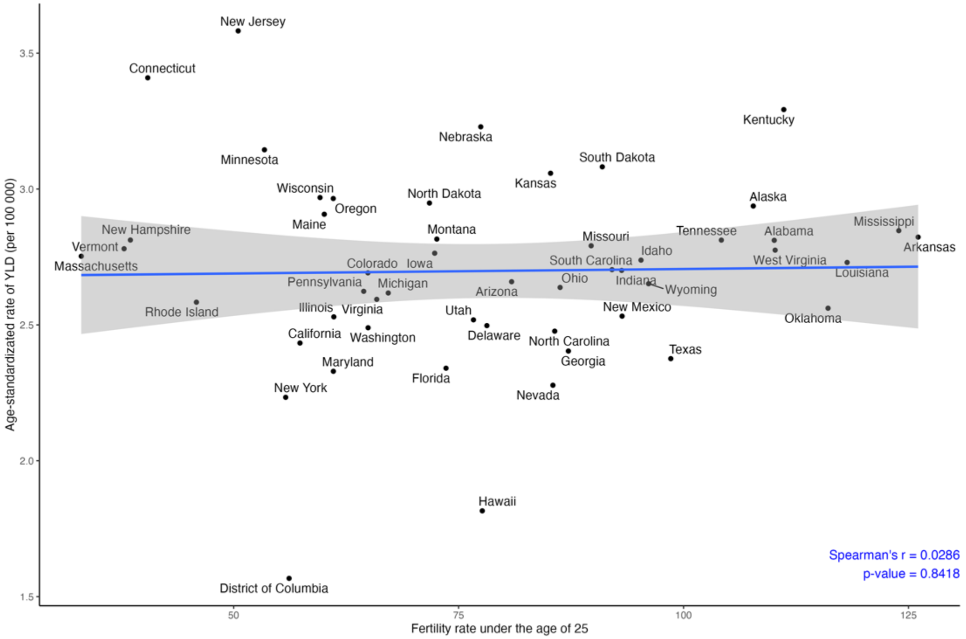

(F) YLLs

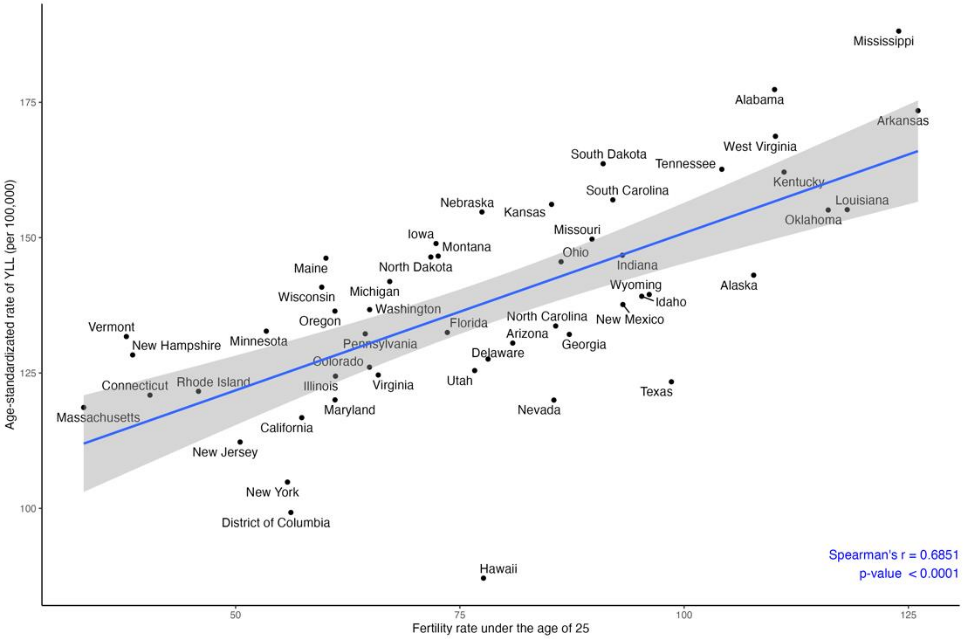

eFigure 9: Correlation between Fertility rate below 25 and age-standardized (A) incidence (B) DALYs (C) death (D) prevalence (E) YLDs (F) YLLs rate of Brain and central nervous system cancer in the United States by state, 2021

© 2025 GBD 2021 US CNS Cancer Collaborators. *JAMA Neurology*.

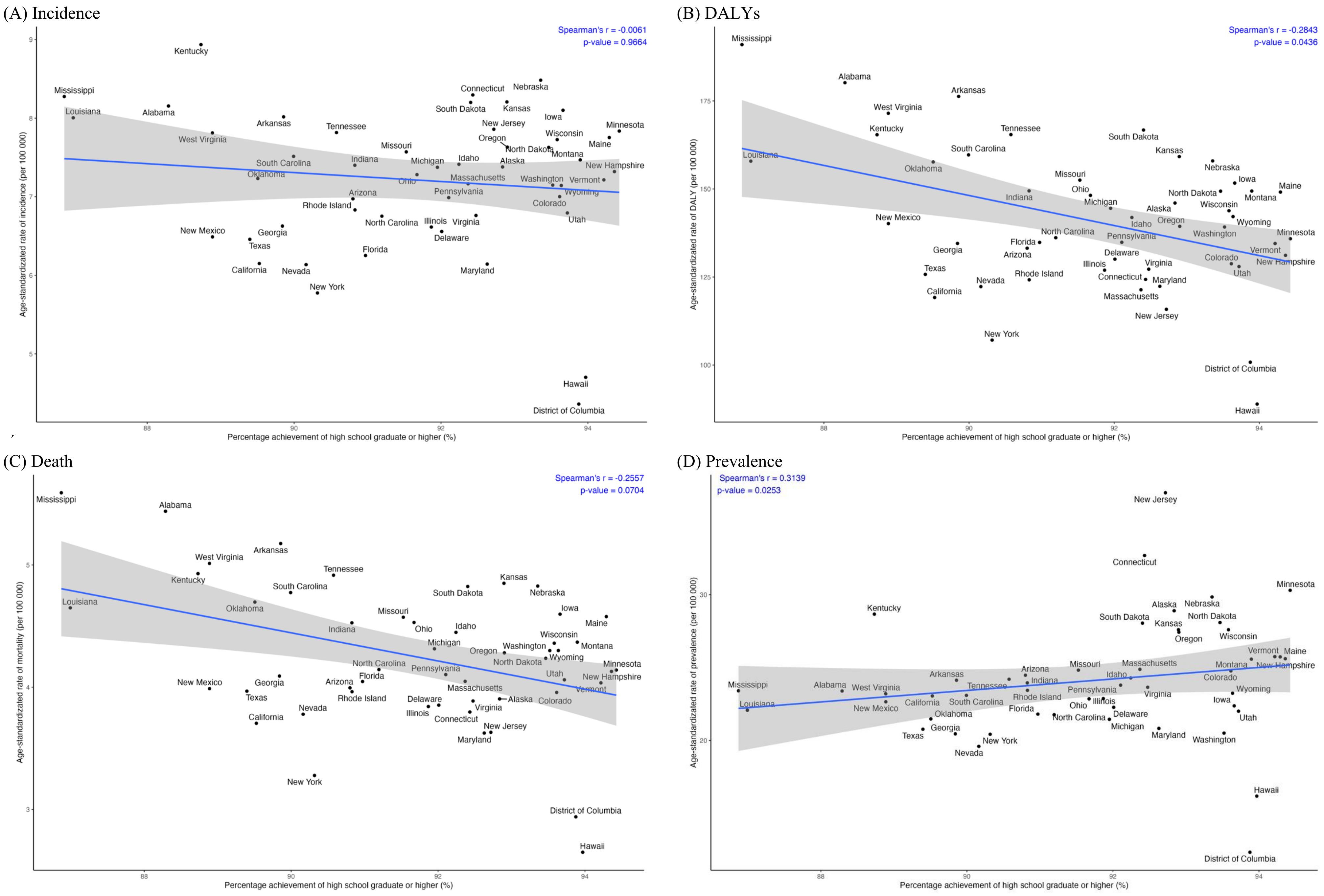

© 2025 GBD 2021 US CNS Cancer Collaborators. *JAMA Neurology*.

(E) YLDs

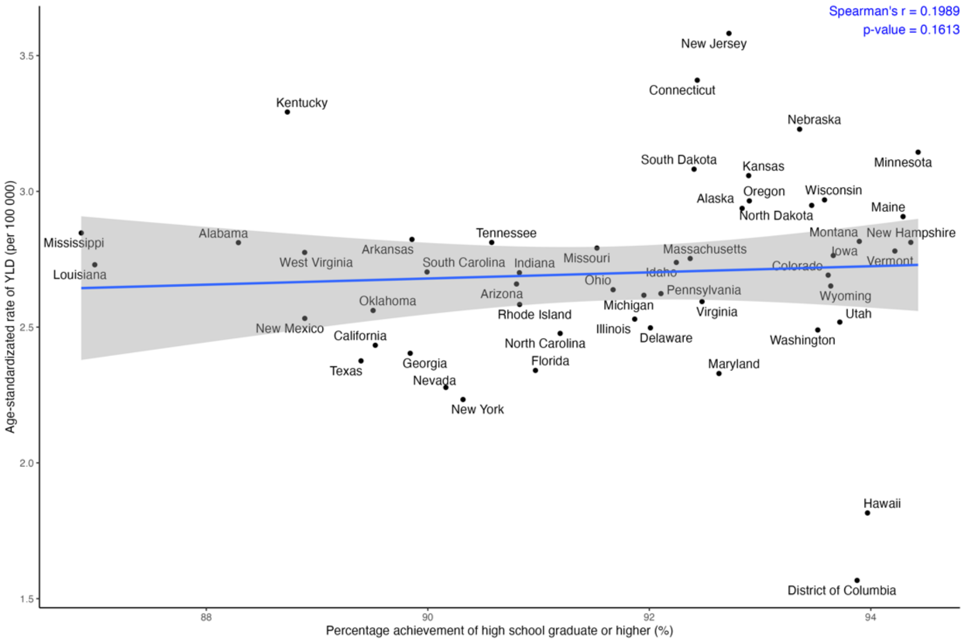

(F) YLLs

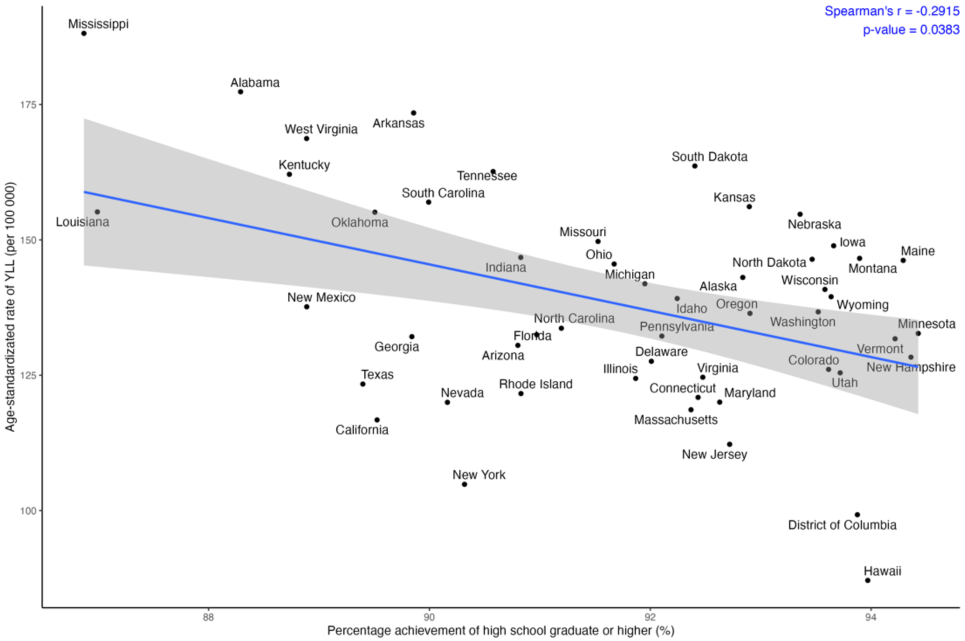

eFigure 10: Correlation between academic achievement and age-standardized (A) incidence (B) DALYs (C) death (D) prevalence (E) YLDs (F) YLLs rate of Brain and central nervous system cancer in the United States by state, 2021

© 2025 GBD 2021 US CNS Cancer Collaborators. *JAMA Neurology*.

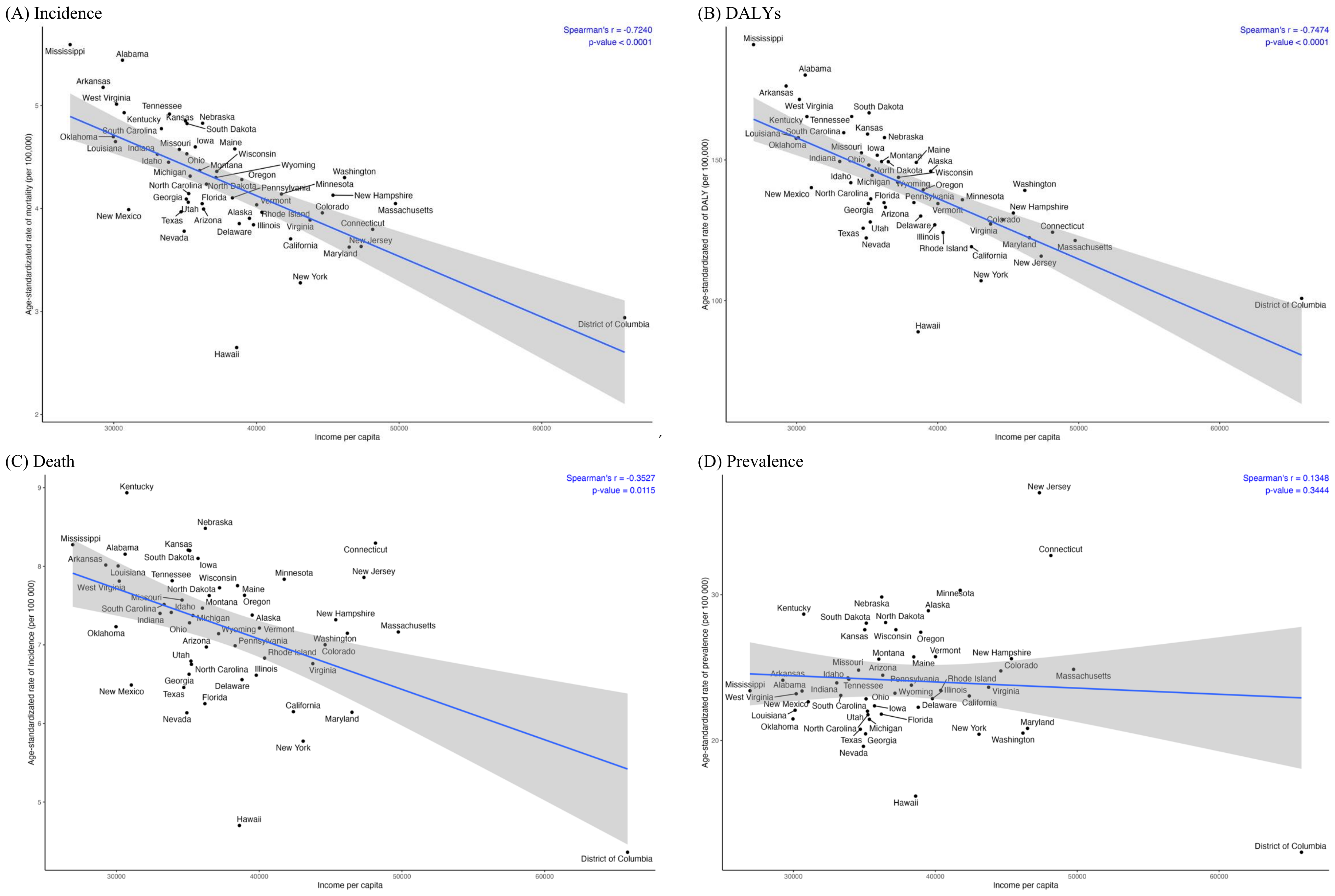

© 2025 GBD 2021 US CNS Cancer Collaborators. *JAMA Neurology*.

(E) YLDs

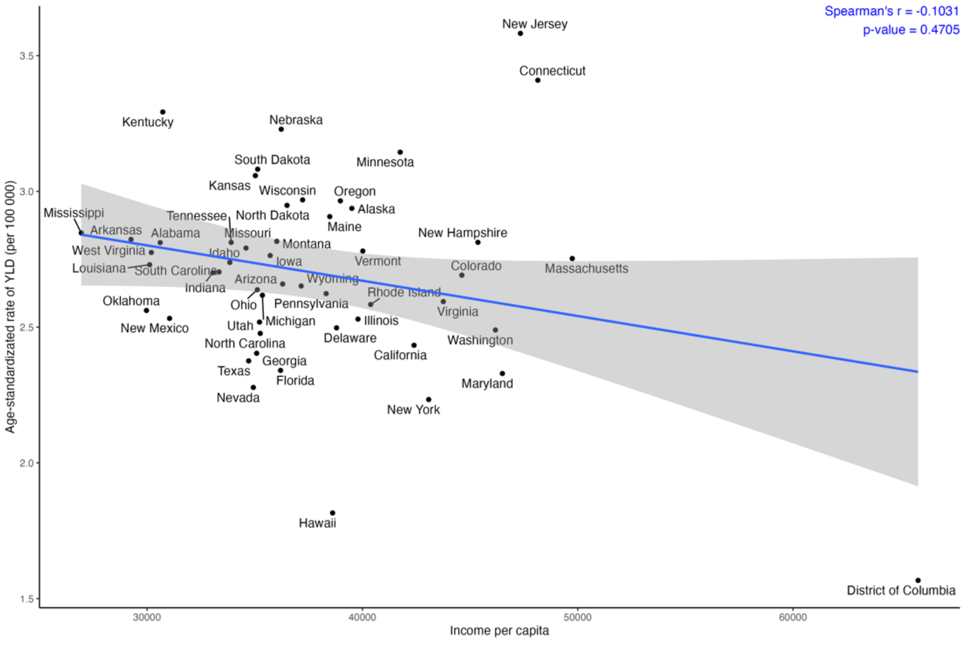

(F) YLLs

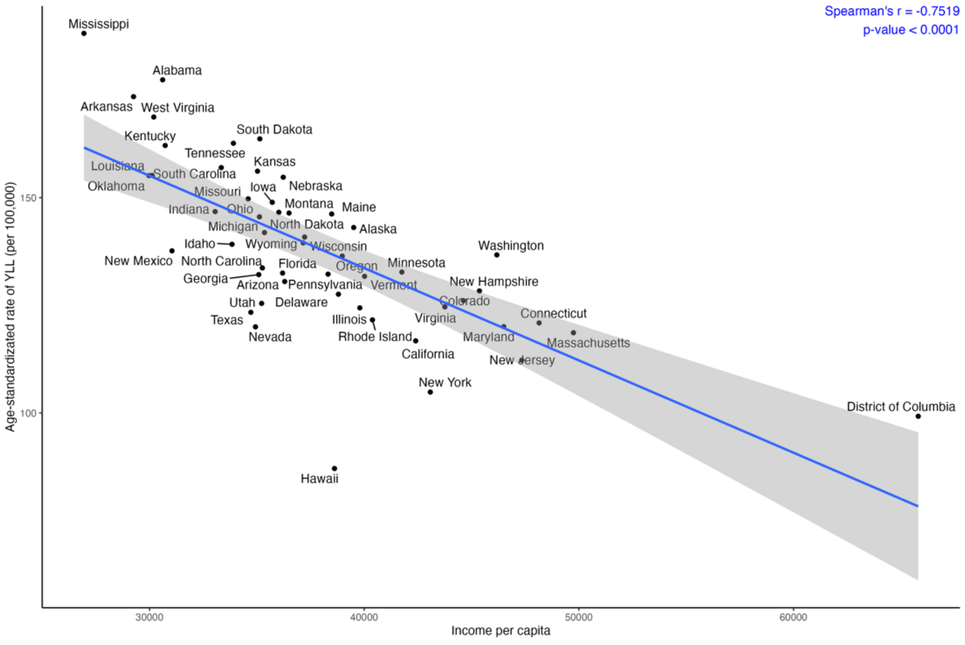

eFigure 11: Correlation between income per capita and age-standardized (A) incidence (B) DALYs (C) death (D) prevalence (E) YLDs (F) YLLs rate of Brain and central nervous system cancer in the United States by state, 2021

© 2025 GBD 2021 US CNS Cancer Collaborators. *JAMA Neurology*.

(A) Prevalence

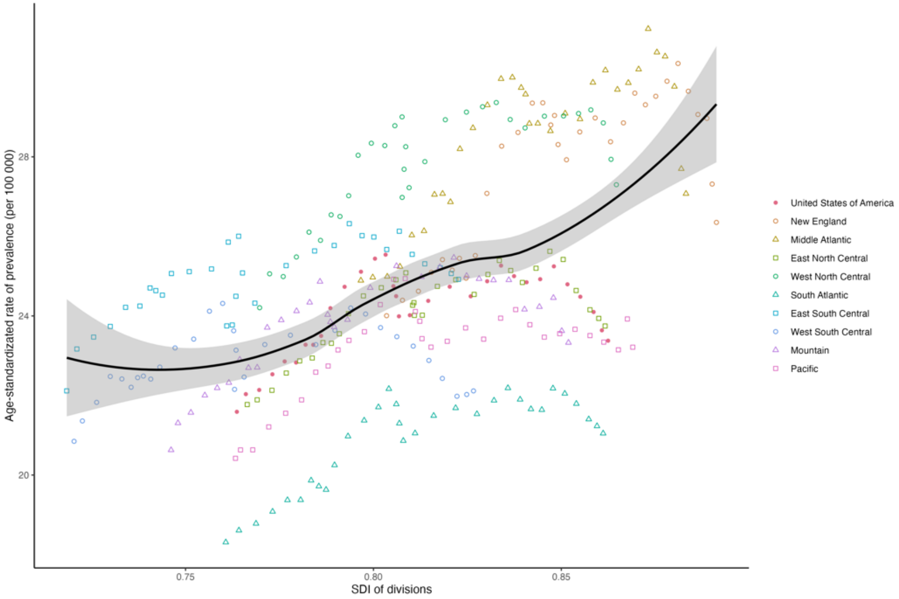

(B) YLDs

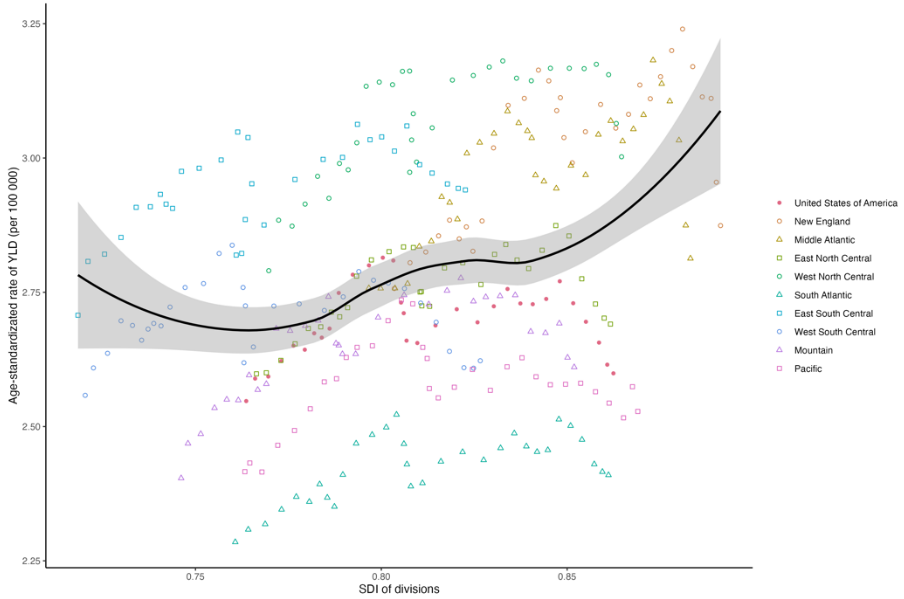

(C) YLLs

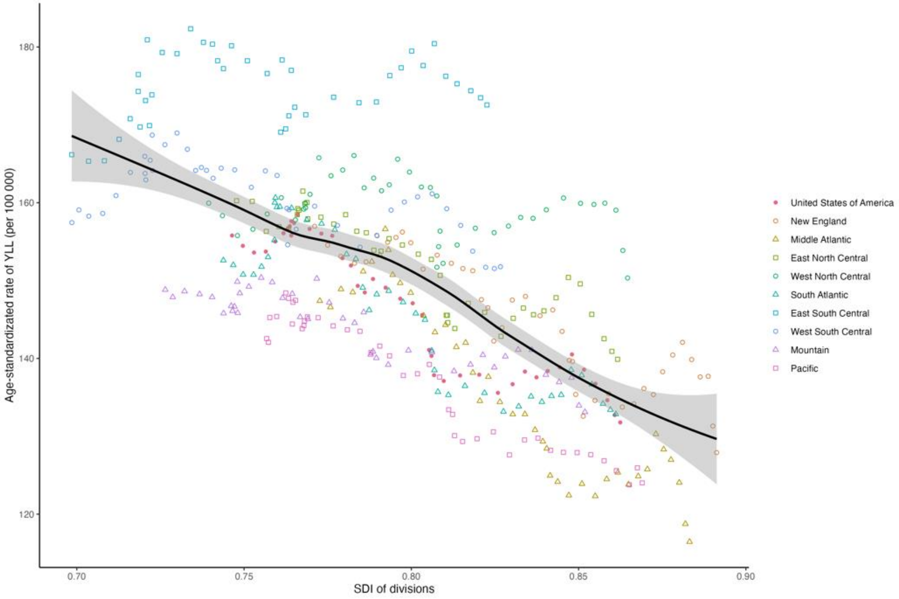

eFigure 12: Correlation between SDI and age-standardized (A) prevalence (B) YLDs (C) YLLs rate of Brain and central nervous system cancer in the United States by division, 2021

© 2025 GBD 2021 US CNS Cancer Collaborators. *JAMA Neurology*.

eAppendix VI: Comparison of GBD 2021 estimate and national report methodologies with plausible reasons behind the discrepancies between them.

The reported sex-specific incidence of total CNS cancers differs between the Central Brain Tumor Registry of the United States (CBTRUS), which shows higher rates in females, and our GBD 2021 analysis, which suggests higher rates in males. This discrepancy is likely multifactorial, and our assessment indicates that it might be attributed to three methodological differences. We propose that the estimates are complimentary rather than contradictory because of methodological differences rather than a reflection of error in either dataset.

- Age-standardization. CBTRUS applies the fixed 2000 US standard population, whereas GBD 2021 uses a global standard based on the average age structure of all countries with populations over 5 million. Because this global standard reflects lower female-to-male ratios than in the US, applying it to US data may lead to an underestimation of female-dominant cancer rates.

- Data processing. GBD estimates are derived through multi-step modeling intended to harmonize data across time and geography. The process includes standardization, mapping, age-sex splitting, corrections for violations, garbage code redistribution, noise reduction, and covariate adjustments (see GBD capstone methods). By contrast, CBTRUS reports are generated more directly from US cancer registry data, with less post-processing, which naturally produces different incidence figures.

-Case definition. While both sources include ICD-10 codes C70–C72, GBD additionally maps C75.1–C75.3 to CNS cancers. Even minor definitional differences can shift sex-specific incidence estimates.

It is neither feasible nor useful to judge one dataset as superior. Instead, we recommend using both sources together. CBTRUS offers detailed national estimates grounded in registry data, while GBD provides a global framework that enables international comparisons and incorporates broader measures such as DALYs. Considering both perspectives offers a more complete understanding of the US cancer burden and places it in a global context.

References:
1. Catalá-López F, Padron-Monedero A, Sarmiento Suárez R, Collaborators GCoD. Global burden of 288 causes of death and life expectancy decomposition in 204 countries and territories and 811 subnational locations, 1990-2021: a systematic analysis for the Global Burden of Disease Study 2021. 2024;

2. Ferrari AJ, Santomauro DF, Aali A, et al. Global incidence, prevalence, years lived with disability (YLDs), disability-adjusted life-years (DALYs), and healthy life expectancy (HALE) for 371 diseases and injuries in 204 countries and territories and 811 subnational locations, 1990–2021: a systematic analysis for the Global Burden of Disease Study 2021. The Lancet. 2024;403(10440):2133-2161.

3. Price M, Ballard C, Benedetti J, et al. CBTRUS statistical report: primary brain and other central nervous system tumors diagnosed in the United States in 2017-2021. *Neuro-oncology*. 2024;26(suppl_6):vi1-vi85.

© 2025 GBD 2021 US CNS Cancer Collaborators. *JAMA Neurology*.
