# Supplementary material for: Burden of Central Nervous System Cancer in the United States, 1990-2021
Source: JAMA Neurol. 2025 Nov 3;83(1):35–48. doi: 10.1001/jamaneurol.2025.4286 (PMC12584065; doi:10.1001/jamaneurol.2025.4286)
Supplement: Supplement 2. — Data sharing statement [file jamaneurol-e254286-s002.pdf]

Data Sharing Statement

Han. Burden of Central Nervous System Cancer in the United States, 1990-2021. *JAMA Neurol*. Published November 03, 2025. doi:10.1001/jamaneurol.2025.4286

Data
Data available: No

Additional Information
Explanation for why data not available: To download the data used in these analyses, please visit the Global Health Data Exchange GBD 2021 website at https://ghdx.healthdata.org/gbd-2021.
